# Differential microglia and macrophage profiles in human IDH-mutant and -wild type glioblastoma

**DOI:** 10.18632/oncotarget.26863

**Published:** 2019-05-03

**Authors:** Candice C. Poon, Paul M.K. Gordon, Katherine Liu, Runze Yang, Susobhan Sarkar, Reza Mirzaei, Shiekh Tanveer Ahmad, Martha L. Hughes, V. Wee Yong, John J.P. Kelly

**Affiliations:** ^1^ Department of Clinical Neurosciences, University of Calgary, Calgary, AB, Canada; ^2^ Centre for Health Genomics and Informatics, University of Calgary, Calgary, AB, Canada; ^3^ Department of Pathology and Laboratory Medicine, University of Calgary, Calgary, AB, Canada

**Keywords:** glioblastoma, microglia, macrophages, isocitrate dehydrogenase, single-cell RNA sequencing

## Abstract

Microglia and macrophages are the largest component of the inflammatory infiltrate in glioblastoma (GBM). However, whether there are differences in their representation and activity in the prognostically-favorable isocitrate dehydrogenase (IDH)-mutated compared to -wild type GBMs is unknown. Studies on human specimens of untreated IDH-mutant GBMs are rare given they comprise 10% of all GBMs and often present at lower grades, receiving treatments prior to dedifferentiation that can drastically alter microglia and macrophage phenotypes. We were able to obtain large samples of four previously untreated IDH-mutant GBM. Using flow cytometry, immunofluorescence techniques with automated segmentation protocols that quantify at the individual-cell level, and comparison between single-cell RNA-sequencing (scRNA-seq) databases of human GBM, we discerned dissimilarities between GBM-associated microglia and macrophages (GAMMs) in IDH-mutant and -wild type GBMs. We found there are significantly fewer GAMM in IDH-mutant GBMs, but they are more pro-inflammatory, suggesting this contributes to the better prognosis of these tumors. Our pro-inflammatory score which combines the expression of inflammatory markers (CD68/HLA-A, -B, -C/TNF/CD163/IL10/TGFB2), Iba1 intensity, and GAMM surface area also indicates that more pro-inflammatory GAMMs are associated with longer overall survival independent of IDH status. Interrogation of scRNA-seq databases demonstrates microglia in IDH-mutants are mainly pro-inflammatory, while anti-inflammatory macrophages that upregulate genes such as *FCER1G* and *TYROBP* predominate in IDH-wild type GBM. Taken together, these observations are the first head-to-head comparison of GAMMs in treatment-naïve IDH-mutant versus -wild type GBMs. Our findings highlight biological disparities in the innate immune microenvironment related to IDH prognosis that can be exploited for therapeutic purposes.

## INTRODUCTION

Glioblastoma (GBM) is the most common adult brain cancer with a median survival of 14.6 months despite aggressive surgery and chemoradiation [1]. Success with immunotherapies such as checkpoint inhibitors in melanoma [2] and lung cancer [3] have prompted researchers to investigate its promise in GBM. However, none have proven efficacious. One possible reason why immunotherapies have failed to prolong survival in GBM is because they focus on modulating T cells, but T cells are sparse in GBM [4] unlike in melanoma [5] and lung cancer [6]. In general, the immune response in GBM is poorly understood and must be elucidated to develop effective treatments.

Single-cell RNA sequencing (scRNA-seq) studies of human GBM, in agreement with more classical immunohistochemical studies [7, 8], have shown that the predominant immune cell type in the tumor microenvironment are myeloid cells comprised of microglia and macrophages [4, 9–11]. It has been suggested that microglia and macrophages within GBM initially participate in tumor surveillance, but are subverted by GBM to adopt grossly anti-inflammatory phenotypes and subsequently promote immunosuppression, tumor angiogenesis and invasion [12]. Nevertheless, it is still unknown whether there is variation in the degree of immunosuppression experienced by GAMMs.

The majority of GAMM research has been in isocitrate dehydrogenase-wild type (IDH-WT) GBMs. In 2016, the World Health Organization Classification of Tumors of the Central Nervous System was revamped to divide GBM into three major categories: IDH-WT, IDH-mutant (-MUT), and IDH not otherwise specified (when diagnostic procedures were lacking to determine IDH status)[13]. IDH-MUT GBMs have a better prognosis [14], are associated with different methylation patterns [15], and are present in younger patients compared to their wild type counterparts [16]. However, studies of untreated IDH-MUT GAMMs are rare because not only do IDH-MUT GBMs account for approximately 10% of GBMs [16], but they almost always present first as lower grade gliomas which are treated with surgery and chemoradiation [17], processes which can drastically alter the native phenotype of microglia and macrophages [18, 19].

Our present study directly compares previously untreated human IDH-WT and -MUT GAMMs, representing a crucial step towards addressing the natural state of microglia and macrophages in these two potentially different microenvironments. We hypothesized that not only will there be heterogeneity in the microglia and macrophage response between GBMs, but that microglia and macrophages in IDH-MUT tumors differ from those in wild type tumors. We found that innate immune cells are heterogeneously represented in wild type GBMs, while found in much smaller numbers in mutants. Notably, microglia and macrophages in mutants are of a more pro-inflammatory phenotype. Even wild type GBM patients with more pro-inflammatory microglia and macrophages had a longer overall survival. Furthermore, anti-inflammatory innate immune cells shared highly upregulated genes in common. These differences in innate immune biology can have important implications for development and selection of immunotherapy in GBM.

## RESULTS

### Microglia and macrophage content is highly variable across IDH-wild type GBM and is decreased in IDH-mutants

It is widely believed that microglia and macrophages make up one-third of all cells within GBM [20]. However, the origin of this estimate and applicability to all GBMs, particularly the newly diagnosed IDH-MUT GBMs, is unknown. Thus, we sought to determine GAMM content by using automated immunofluorescence segmentation techniques [21] with validation through flow cytometry. All GBM patients were previously untreated to allow characterization of the native microglia and macrophage state.

A surprisingly large range of CD11b+CD45+ cells was seen from approximately 0.0% to 65.4% of the parent population of IDH-WT GBM cells (Figure 2A). This substantial variation was again seen in matched GBMs (from 1.6±0.6% to 71.9±13.4%) using Iba1 as a pan-microglia and macrophage marker (Figure 2B and 2C). Different pan-microglia and macrophage markers (CD11b and CD45 double-positivity for flow cytometry, and Iba1 positivity for immunofluorescence) were used since dependence on only one microglia and macrophage marker for identification is insufficient. Furthermore, to ensure Iba1 did not co-label with astrocytic markers such as glial fibrillary acidic protein (GFAP), a double immunofluorescent stain of Iba1 and GFAP was performed in six GBMs (representative images are shown in Supplementary Figure 1). A positive correlation was observed between flow cytometry and immunofluorescence estimates of microglia and macrophage content (Pearson r = 0.73, p = 0.03; Figure 2D), thereby supporting the validity of our measurements.

**Figure 1:**
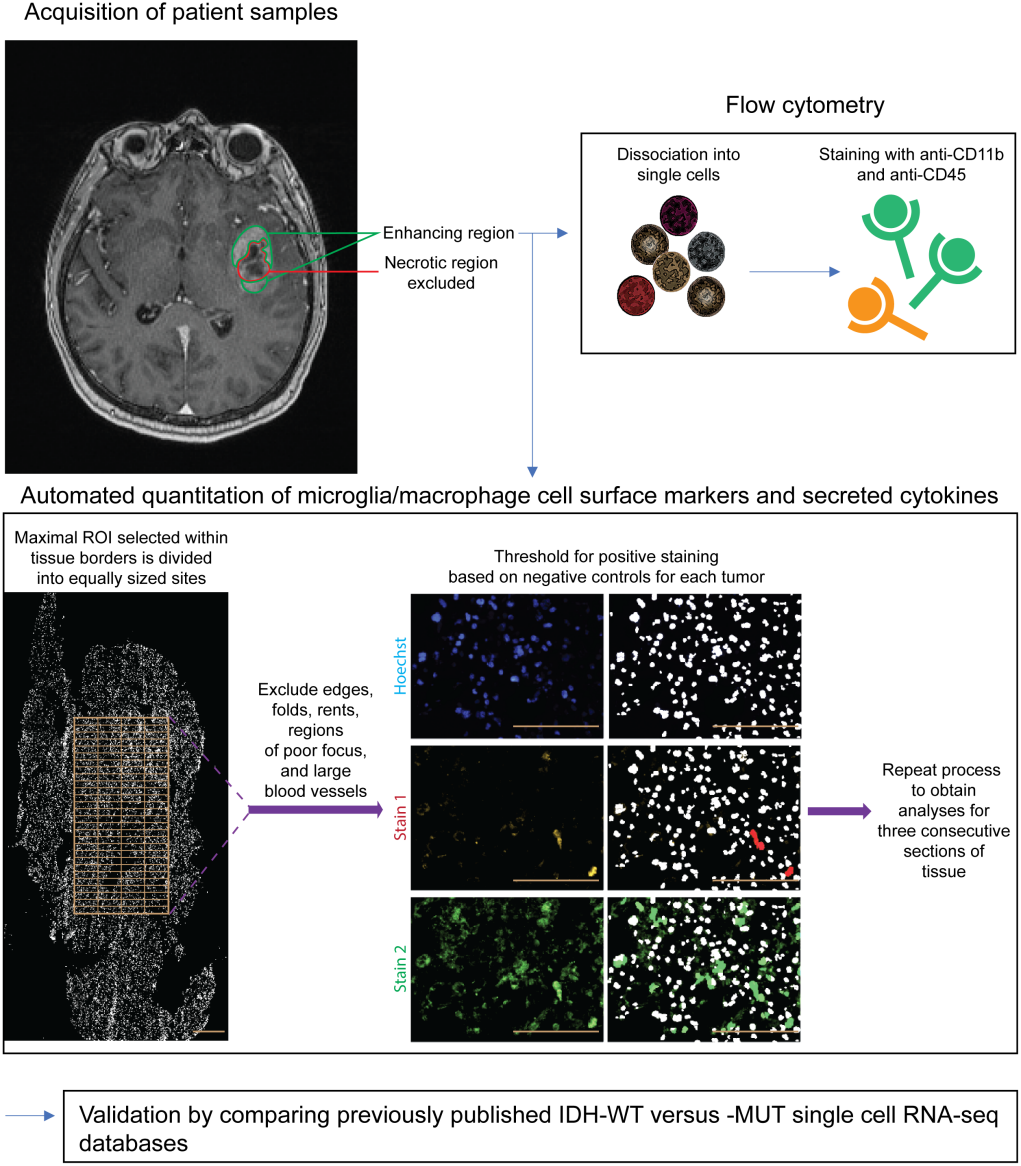
Schematic of methodology Only untreated IDH-WT and -MUT GBMs were included in this study. A representative T1-weighted post-gadolinium MRI shows the enhancing region from which tissue was obtained. Areas of gross necrosis were avoided. Matched GBM samples were then analyzed via flow cytometry and immunofluorescence techniques. An automated image acquisition and segmentation protocol (see Methods) was used to quantitate microglia and macrophages. Finally, results were independently validated and further refined using bioinformatics comparison of scRNA-seq databases of IDH-MUTand -WT astrocytomas, with inclusion of only those tumors that were GBMs. Scale bars are 100 μm.

**Figure 2:**
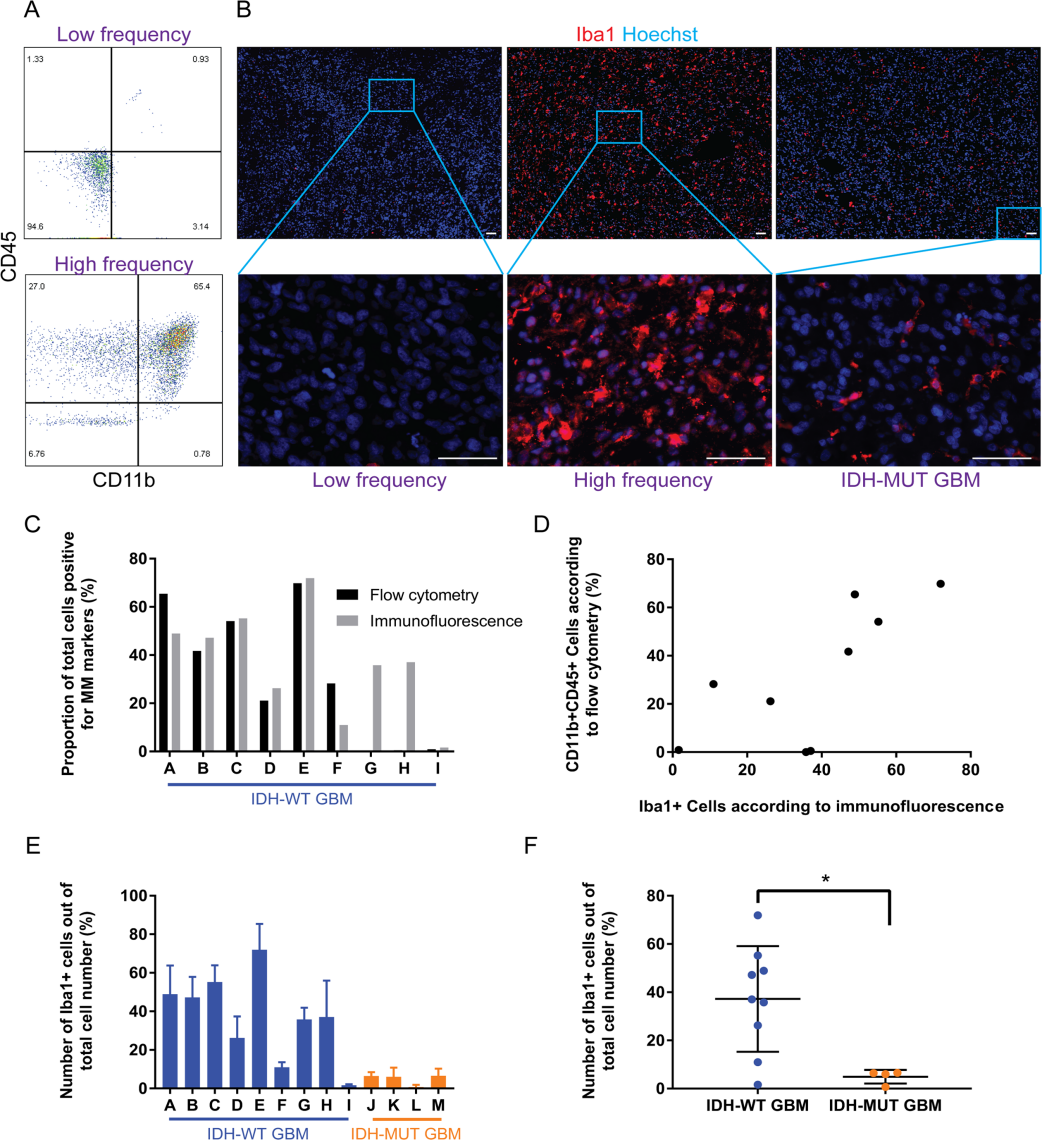
While the proportion of GAMM varies considerably across IDH-wild type GBMs, there is consistently less GAMM in IDH-mutant GBMs **(A)** Flow cytometry reveals that microglia and macrophage (CD11b+CD45+; upper right quadrant) content in GBMs can range from almost none (top panel) to approximately 65% of the tumor (bottom panel). **(B)** Immunofluorescence of tissue sections verifies the heterogeneity of microglia and macrophage (Iba1+) content in examples of low and high frequency in IDH-wild type GBMs; an image from an IDH-mutant is also displayed. Scale bars are 50 μm. **(C)** Graphical representation and comparison of GAMM counts using both flow cytometry and immunofluorescence/automated segmentation techniques. **(D)** Correlation analysis reveals a significantly positive correlation between microglia and macrophage numbers obtained using flow cytometry and immunofluorescence (Pearson r = 0.73, p = 0.03). **(E)** Proportion of GAMM across GBMs, displaying heterogeneity in IDH-wild type tumors and consistently low numbers in IDH-mutants. Values for each tumor are mean ± SD (n = 3 sections for each GBM). **(F)** Evaluated as a group, there is a statistically significant difference in the amount of GAMM between IDH-wild type and -mutant GBMs (p = 0.02).

A stark difference was observed between microglia and macrophage content in IDH-WT compared to –MUT GBMs; mean frequency of IDH-WT GAMMs was 37.2±7.3% while it was 4.9±1.4% in IDH-MUTs (p = 0.02; Figure 2E and 2F). Of note, as shown in the low magnification images in Figure 2B, microglia and macrophages were relatively evenly distributed across entire GBM sections instead of being in obvious clusters.

### Individual markers of GAMM activity are highly variable across specimens

Next, we sought to investigate the inflammatory profile of GAMMs by interrogating three pro- and anti-inflammatory markers each. While cell surface markers of inflammation are usually used, cytokines are one of the most salient indicators of inflammatory state. Thus, we incorporated a mixture of cell surface markers and cytokines in our inflammatory panels. In GBM, increased microglia and macrophage phagocytic ability, indicated by CD68, marks a more pro-inflammatory state [22]. Similarly, upregulation of HLA-A, -B, and -C results in more difficulty with immune evasion and is associated with a pro-inflammatory phenotype [23]. Lastly, we stained for tumor necrosis factor-alpha (TNF) because of its canonical pro-inflammatory nature and since it is the most widely used output of microglia and macrophage activation [24].

**Figure 3:**
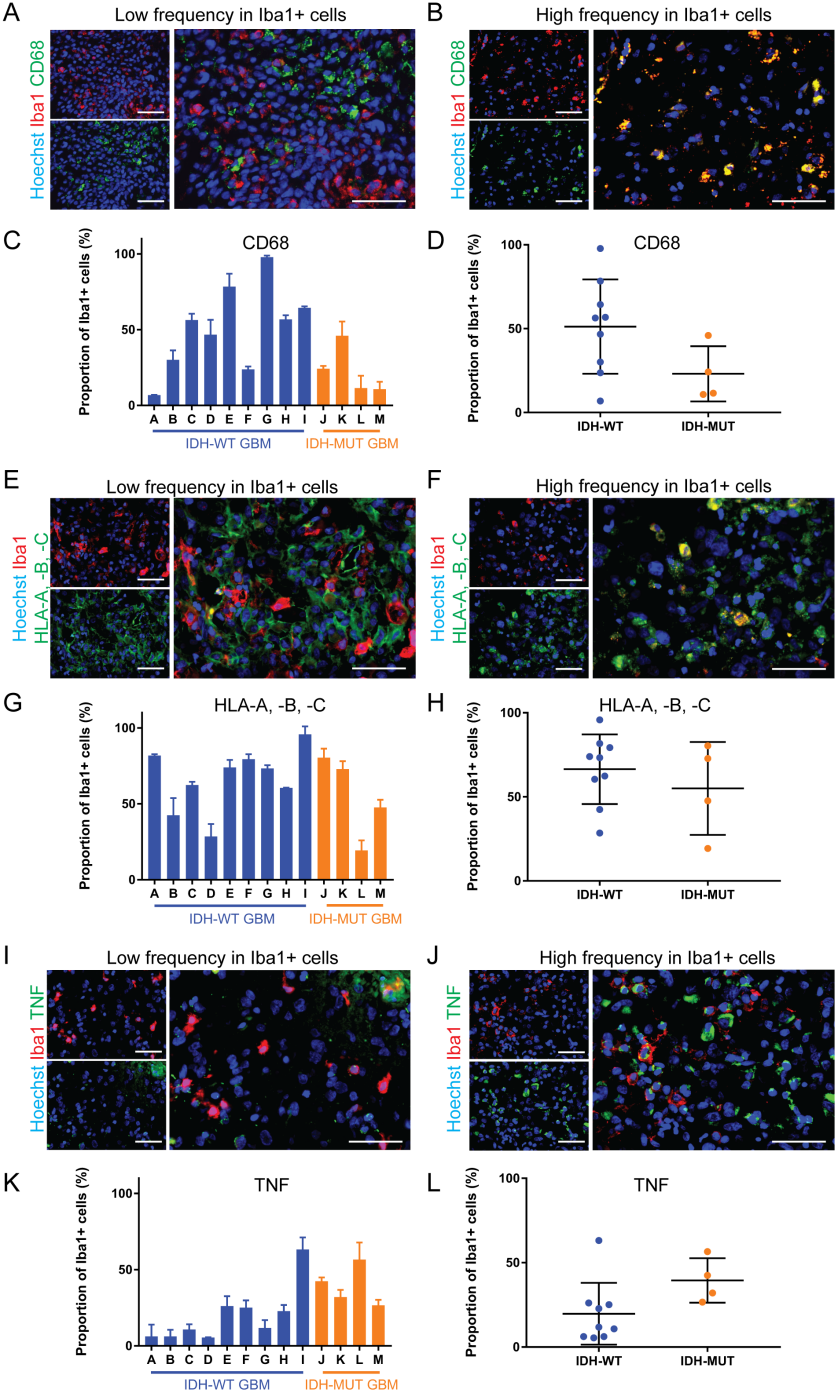
The expression of pro-inflammatory markers is highly variable in GAMMs **(A, B)** Representative images of low (A) and high (B) frequency of the pro-inflammatory marker CD68 in Iba1+ microglia and macrophages of different GBMs. **(C, D)** Graphical representation of the results in (A and B), p = 0.09. **(E, F)** Representative images of low (E) and high (F) frequency of the pro-inflammatory marker HLA-A, -B, -C in microglia and macrophages of different GBMs. **(G, H)** Graphical representation of the results in E and F (p = 0.42). **(I, J)** Representative images of low (I) and high (J) frequency of the pro-inflammatory marker TNF in microglia and macrophages of different GBMs. **(K, L)** Graphical representation of the results in (I and J), p = 0.08. Scale bars are 50 μm.

CD68 frequency in GAMMs ranged from 6.9±0.1% to 97.8±1.0% (Figure 3A-3D). A large range in expression was similarly seen with HLA-A, -B, -C (19.3±6.6% to 95.8±5.2%; Figure 3E-3H) and TNF (5.4±0.2% to 63.2±7.9%; Figure 3I-3L). While mean CD68 expression was approximately two-fold less frequent in IDH-MUT GAMMs (WT 51.2±9.4% versus MUT 23.1±8.2%), and TNF expression two-fold higher (WT 19.7±6.1% versus MUT 39.4±6.6%) than their wild type counterparts, these differences were not statistically significant (p = 0.09 and p = 0.08, respectively). The expression of HLA-A, -B, -C in microglia and macrophages was not statistically different between IDH-WT and IDH-MUT GAMMs (66.4±6.9% versus 55.01±13.8%, respectively, p = 0.21).

**Figure 4:**
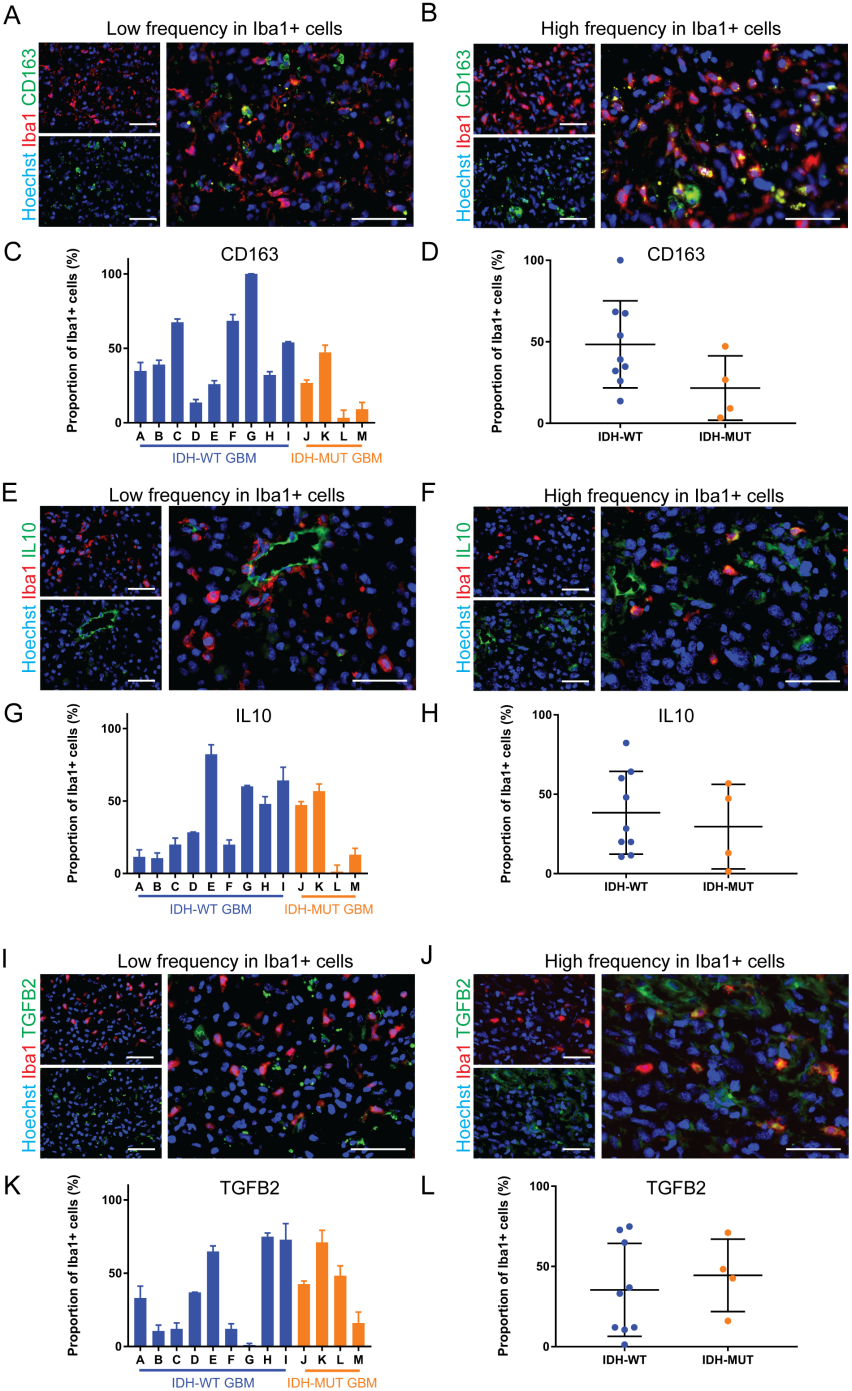
Anti-inflammatory markers are differentially expressed by GAMMs **(A, B)** Representative images of low (A) and high (B) frequency of the anti-inflammatory marker CD163 in microglia and macrophages of different GBMs. **(C, D)** Graphical representation of the results in A and B (p = 0.10). **(E, F)** Representative images of low (E) and high (F) frequency of the anti-inflammatory marker IL10 in microglia and macrophages of different GBMs. **(G, H)** Graphical representation of the results in E and F (p = 0.59). **(I, J)** Representative images of low (I) and high (J) frequency of the anti-inflammatory marker TGFB2 in microglia and macrophages of different GBMs. **(K, L)** Graphical representation of the results in I and J (p = 0.59). Scale bars are 50 μm.

For our anti-inflammatory marker panel, we chose CD163 because it is often used as an anti-inflammatory marker in microglia and macrophages [25, 26]. Interleukin-10 (IL10) and transforming growth factor-beta 2 (TGFB2) are major anti-inflammatory cytokines secreted by microglia and macrophages [27, 28] that are thought to heavily enforce the immunosuppressive microenvironment of GBM [29, 30]. Mean CD163+ GAMM frequency in IDH-MUT (21.6±9.9%) was half that of IDH-WT (48.4±8.9%) but this was not statistically significant (p = 0.10;) (Figure 4A-4D). IL10 (WT 38.4±8.7% versus MUT 29.6±13.3%) and TGFB2 (WT 35.4±9.7% versus MUT 44.5±11.3%, p = 0.59) did not differ between the genotypes (Figure 4E-4L). Once again, incredible heterogeneity in the expression of these markers in GAMMs was seen both within IDH-WT GBMs and between IDH-WT and -MUT GBMs; CD163+ microglia and macrophages ranged from 3.4±5.1% to 100±0.0%, IL10+ microglia and macrophages ranged from 1.4±4.5% to 82.3±6.5%, and TGFB2+ microglia and macrophages ranged from 1.3±1.1% to 74.9±2.5%.

**Figure 5:**
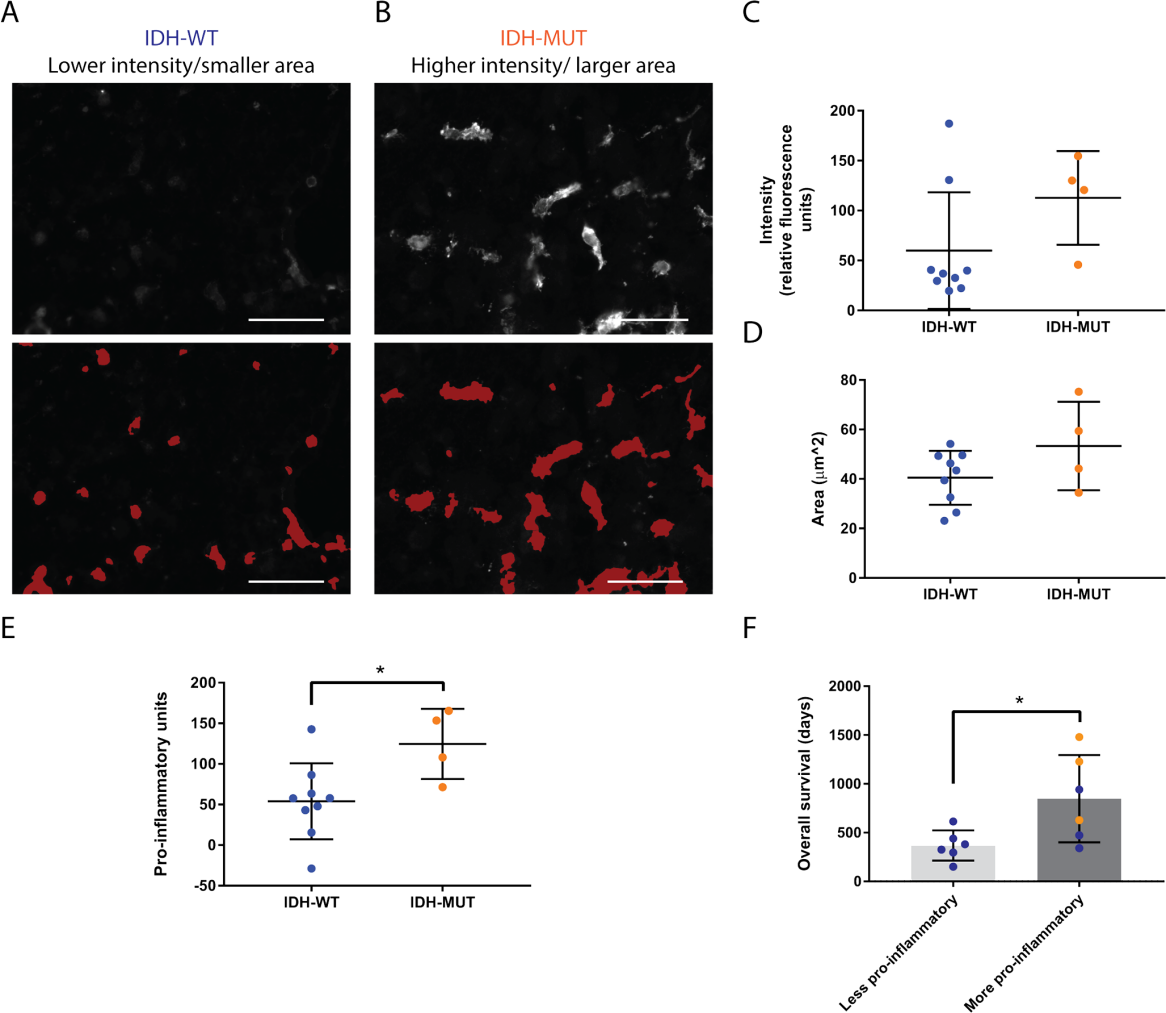
Microglia and macrophages are more pro-inflammatory in IDH-MUT compared to IDH-WT GBMs **(A, B)** Representative images and corresponding overlays generated from the automated segmentation protocol of Iba1 intensity (A) and surface area (B) differences between IDH-WT and -MUT glioblastomas. Scale bars are 50 μm. **(C, D)** Graphical representation of the statistical comparison between A and B (p = 0.14 and p = 0.13, respectively). **(E)** Graphical representation of the activation profile revealing that IDH-MUT glioblastoma-associated microglia and macrophages are more pro-inflammatory than IDH-WT GAMMs (p = 0.03). The activation profile was compiled by tabulating the overall inflammatory status, the Iba1 intensity, and the surface area of cells. **(F)** Overall survival is significantly greater in GBMs with higher activation profiles as determined by median split without regard to IDH status (p = 0.03). Nonetheless, values obtained from IDH-MUT glioblastomas (orange) fell into the higher pro-inflammatory and survival categories. One IDH-mutant GBM patient was lost to follow up leading to exclusion from this analysis.

In addition to inflammatory status, increased Iba1 intensity [31] and increased area [32] are also indicators of activation in microglia and macrophages. Hence, we used our automated segmentation protocol to quantify these parameters in GAMMs. IDH-MUT GAMMs reached an average of 112.7±23.5 relative fluorescence units versus 60.0±19.4 relative fluorescence units of IDH-WT GAMMs, but this was not statistically significant (p = 0.14; Figure 5A-5C). The mean area of IDH-MUT GAMMs was 53.3±8.9 μm^2^ compared with 40.5±3.6 μm^2^ of IDH-WT GAMMs (p = 0.13; Figure 5B-5D).

In summary, the use of single markers to inform on the activity of GAMMs was not instructive, contributed by the large range of expression of each marker across specimens. Thus, we sought to combine these individual markers in a pooled analysis to inform on the overall activity of GAMMs.

### IDH-mutant glioblastoma-associated microglia and macrophages are more pro-inflammatory than those in IDH-wild type glioblastomas and pro-inflammatory status is associated with overall survival

Many parameters have previously been associated with microglia and macrophage pro-inflammatory activation [33, 34]. While there is no one standard definition of activation, what is becoming clearer is that multiple parameters should be incorporated to determine the pro-inflammatory status of microglia and macrophages [35, 36]. Thus, we chose to use a combination of cell surface markers, cytokines, Iba1 intensity, and cell area. When all these characteristics are considered together, even though there are less GAMMs in IDH-MUT GBMs, they are more pro-inflammatory than in IDH-WT GBMs (124.5±21.6 pro-inflammatory units versus 54.0±15.6 pro-inflammatory units, p = 0.03; Figure 5E). Furthermore, when separating GBMs regardless of IDH-mutation status into those with higher and lower pro-inflammatory scores according to median split, patients with higher GAMM pro-inflammatory status had longer overall survival (847.8±182.4 days compared to 367.8±63.2 days, respectively, p = 0.03; Figure 5F). Notably, all IDH-mutant GBMs had a higher pro-inflammatory profile.

### The pro-inflammatory innate immune phenotype of IDH-MUT GBMs is driven by microglia

**Figure 6:**
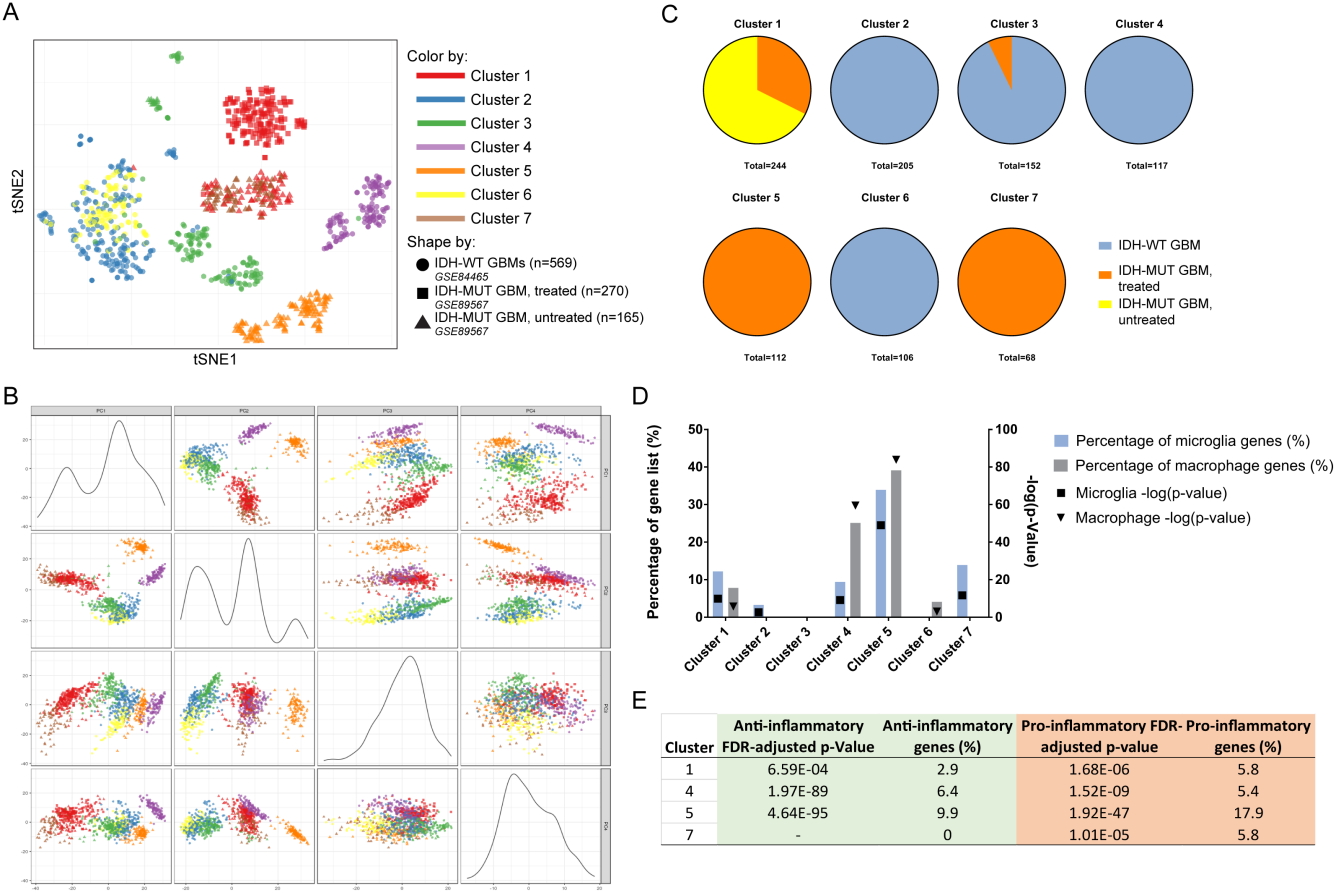
Comparison of scRNA-seq databases identifies microglia as the predominant source of pro-inflammatory milieu in IDH-MUT GBMs compared to their IDH-WT counterparts **(A)** tSNE plot showing individual cell clusters generated according to gene expression values. **(B)** Principal component plots demonstrating clustering based on gene expression values. **(C)** The origin of cells according to data source is shown. Notice in both A and B Clusters 1 and 7 (innate immune cells) and Clusters 2 and 6 (presumed neoplastic cells) congregate together. **(D)** The corrected FDR p-value and percentage of curated microglia and macrophage gene list is graphically represented for each cluster. Highly significant representation of GAMMs is found in Clusters 1, 4, 5, and 7. **(E)** Further analysis of innate immune clusters reveals the anti- or pro-inflammatory expression of each cluster. The most pro-inflammatory GAMMs are found in the untreated IDH-MUT GBM. The treated IDH-MUT has a complement of both pro- and anti-inflammatory GAMMs. The IDH-WT GBMs have a predominance of anti-inflammatory macrophages while the IDH-MUT GBMs have a preponderance of pro-inflammatory microglia.

To further understand the innate immune phenotype, we examined scRNA-seq databases. Since microglia and macrophages cannot be reliably distinguished at the protein level, we sought to determine their relative contribution to the GBM microenvironment by directly comparing the only available IDH-MUT GBM scRNA-seq database with another containing IDH-WT GBMs. A total of 1,004 cells were available for analysis (569 from untreated IDH-WT GBMs, 270 from a treated IDH-MUT GBM [identified as MGH45 in GEO accession number GSE89567], and 165 from an untreated IDH-MUT GBM [identified as MGH57 in GEO GSE89567]). We then separated scRNA-seq libraries via clustering by gene expression using both t-distributed stochastic neighbor embedding (tSNE; Figure 6A) and principal component analysis (PCA; Figure 6B and Supplementary Figure 2) techniques. Regardless of separation method, Clusters 1 and 7 segregated together, as well as Clusters 2 and 6. Absolute contributions of cells from each database are shown in Figure 6C.

Gene enrichment analyses based on the microglia and macrophage curated gene list revealed that Clusters 1 (79 cells from the treated IDH-MUT GBM, 165 cells from the untreated IDH-MUT GBM), 4 (117 cells from the IDH-WT GBMs), 5 (112 cells from the untreated IDH-MUT GBM), and 7 (68 cells from the treated IDH-MUT GBM) were highly enriched for microglia and/or macrophage genes (Figure 6D and Supplementary Table 4). Ingenuity pathway analysis (IPA) agreed with these designations by showing that the remaining clusters (Clusters 2, 3, and 6) had upregulation of canonical non-immune pathways involved in metastasis, neuronal processes, and neoplasia, respectively (Supplementary Table 5).

Cluster 1 had higher expression of microglia (p = 1.21 × 10^-10^, 12.2% of microglia gene list) than macrophage genes (p = 1.68 × 10^-6^, 7.8% of macrophage gene list,) that were mostly pro-inflammatory (p = 1.68 × 10^-6^, 5.8% of pro-inflammatory gene list; Supplementary Table 4). IPA revealed the most upregulated pathway was eukaryotic initiation factor 2 (*EIF2*) signaling (p = 5.01 × 10^-14^; Supplementary Table 5), a molecule shown to regulate pro-inflammatory cytokine expression [37]. This was similarly the most upregulated pathway in Cluster 7 (p = 1 × 10^-8^; Supplementary Table 5), which was enriched with pro-inflammatory (p = 1.01 × 10^-5^, 5.8% of gene list) microglia genes (p = 2.18 × 10^-12^, 13.9% of gene list).

Cluster 4 was very significantly enriched with anti-inflammatory (p = 1.97 × 10^-89^, 6.4% of anti-inflammatory gene list; Supplementary Table 4) macrophages (p = 3.18 × 10^-60^, 25.1% of gene list). Several pathways supporting this designation were strongly represented including those involved in communication between innate and adaptive immune cells (p = 1 × 10^-12^), the antigen presentation pathway (p = 2.14 × ^10-9^), and the most highly upregulated pathway dendritic cell maturation (p = 1.58 × 10^-17^; Supplementary Table 5). This pathway was linked to expression of genes such as *HLA-DRB1*, *HLA-DRA*, and *FCGR1B*, molecules all known to be highly expressed in macrophages [38, 39].

Lastly, Cluster 5 contained microglia (p = 9.98 × 10^-50^, 33.9% of gene list) and macrophages (p = 1.3 × 10^-84^, 39.1% of gene list) that were mostly anti-inflammatory (p = 4.64 × 10^-95^, 9.9% of gene list), although there was a smaller pro-inflammatory representation (p = 1.92 × 10^-47^, 9.9% of gene list; Supplementary Table 4). Again, the dendritic cell maturation pathway was highly upregulated (p = 1.58 × 10^-17^) as well as the *NFAT* pathway which is known to be important in microglial and macrophage toll-like receptor signaling (Supplementary Table 5)[40, 41]. Please refer to Figure 6E for summarized inflammatory data.

Due to rigorous normalization processes that allowed data from one scRNA-seq database to be directly compared with another (for example, analysis of only cells in G1 phase, cells with at least 3,000 genes, etc.)[42], some data was excluded from analyses. Thus, only 165 data points from the untreated IDH-MUT GBM were included and we cannot confidently make statements about the quantity of microglia and macrophages since we do not know the denominator.

Overall, strongly anti-inflammatory macrophages were only found in IDH-WT and treated IDH-MUT GBMs. The untreated IDH-MUT GBM was represented mostly by pro-inflammatory microglia, which were also found in the treated IDH-MUT GBM.

### *FCER1G*, *TYROBP*, *C1QA*, *C1QB*, and *CD74* were highly upregulated genes common to anti-inflammatory microglia and macrophages in Clusters 4 and 5

Anti-inflammatory microglia and macrophages in Clusters 4 and 5 shared 5 out of 10 of the most upregulated genes in each cluster (Supplementary Table 4). Interestingly, all five of these genes, *CD74*, *C1QB*, *FCER1G*, *TYROBP*, and *C1QA* are part of a protein-protein interaction network comprised of 20 proteins in total that are found only in head and neck squamous cell carcinomas but not in normal head and neck specimens [43]. Furthermore, *FCER1G* and *TYROBP* have been identified as two of three hub genes in a protein-protein interaction network positively correlated with the progression of clear cell renal cell carcinoma [44]. *FCER1G* and *TYROBP* encode for ITAM-containing adaptor proteins which may play major roles in CSF1R signaling [45], a receptor crucial to microglia and macrophage homeostasis and differentiation [46]. Similarly, *C1QA* and *C1QB* are involved in regulating IFNγ signaling [47], a pathway crucial to pro-inflammatory cytokine secretion in microglia and macrophages. Lastly, *CD74*, the migration inhibitory factor receptor that is expressed on GAMMs, has been described as a means by which IFNγ signaling is disrupted, thus resulting in the promotion of an anti-inflammatory environment [48]. Unlike the anti-inflammatory genes, different pro-inflammatory genes were expressed by microglia in Clusters 1, 5, and 7. Altogether this suggests that there are targetable anti-inflammatory genes commonly upregulated by GAMMs that has relevance to other cancer types.

## DISCUSSION

This is the first study to provide a head-to-head comparison between microglia and macrophages in human untreated IDH-MUT and -WT GBMs. In this report, we found that the innate immune microenvironment in these two categories of GBM was distinct. First, we showed that microglia and macrophage content was strikingly lower in IDH-MUT GBMs than their wild type counterparts. This is in line with a study in human IDH-mutated lower grade glioma and a syngeneic mouse model for IDH-MUT glioma which showed that downregulation of leukocyte chemotaxis contributed to less microglia and macrophage numbers in IDH-MUT tumors [49]. The lower amount of microglia and macrophages in IDH-MUT GBMs may either be a cause or consequence of GBM behavior. It would be important in the future to determine whether GAMM accumulation and associated activities promote malignancy, or whether their presence is a symptom of cancer-driven immunomodulation. The former would suggest immunotherapies targeting GAMM elimination or repulsion need to be developed, while the latter supports efforts to combat GBM-led immunomodulation for instance by re-stimulating GAMMs. Furthermore, decreased GAMM content in IDH-MUTs suggests immunotherapies aimed at activating innate immune cells would be more appropriately applied to IDH-WT GBMs.

In addition to differences in GAMM content, IDH-MUT GAMMs also displayed a disparate activation profile from IDH-WT GAMMs. Most of the literature suggests that GBM creates a potently immunosuppressive microenvironment that influences microglia and macrophages to adopt and perpetuate similarly anti-inflammatory profiles [12]. However, we show that the frequency of pro- and anti-inflammatory GAMMs varies drastically from GBM to GBM, especially in IDH-WTs, suggesting that levels of immunosuppression are also variable between tumors. The heterogeneity of our results makes the case for first profiling the innate immune milieu of a patient’s GBM prior to immunotherapy selection. For instance, administering immunostimulants to GAMMs which are already pro-inflammatory would likely result in no benefit. The informed selection of immunotherapies during clinical trials is particularly important given the expense of getting new drugs to market and dilution of treatment effects by giving candidate therapies to non-responders.

By considering multiple indicators of activation together, we find that IDH-MUT GAMMs possess more pro-inflammatory characteristics than IDH-WT GAMMs. Our scRNA-seq data mining of results from independent laboratories verifies this finding and suggests that the more pro-inflammatory GAMM in IDH-MUT results from microglia; conversely, the anti-inflammatory phenotype of GAMMs in IDH-WT GBMs appears to be driven by macrophages. Interestingly while different pro-inflammatory microglia clusters upregulated different pro-inflammatory genes, the anti-inflammatory genes that were the most highly upregulated were shared between clusters dominated by macrophages. Thus, these genes, *FCER1G*, *TYROBP*, *C1QA*, *C1QB*, and *CD74* represent actionable targets for future therapy development. Interestingly, several of them are involved in the CSF1R and IFNγ signaling pathways which play major roles in microglia and macrophage biology and activation. Overall, it appears that researching immunostimulants to promote pro-inflammatory phenotypes in macrophages specifically is a promising endeavor. The fact that these cells start in the periphery as monocytes also suggests that immunostimulants could be administered systemically and may not necessarily need to penetrate the blood brain barrier.

A limitation of this study is the relatively small sample size. However, the untreated IDH-MUT GBM tissue is rare so this limitation is difficult to overcome. Nonetheless, our quantitation methods are automated and absolute instead of semi-quantitative or manual. Lastly, while techniques such as high-dimensional single-cell analyses using mass cytometry are now available and allow the use of more markers than our comparatively simple immunofluorescence techniques, the processing required to run a sample through the mass cytometer including enzymatic, chemical, and mechanical dissociation into single-cell suspensions intrinsically alters the phenotype of microglia and macrophages prior to data capture [50], which would obviate our goal of determining the naïve GAMM activation state.

In conclusion, the phenotype of GAMM differs between IDH-WT and -MUT GBMs. Our results show this novel distinction at the protein level in rare untreated human IDH-MUT GBMs and at the single-cell RNA level through public databases. Not only are substantially less microglia and macrophages found in mutants, but they are also more pro-inflammatory, a generally sought-after state in GBM treatment because of its association with immunostimulatory and tumor-suppressing properties [51, 52]. Increased microglia and macrophage pro-inflammatory activation may help explain why GBM patients with IDH mutations fare better than those without. Indeed, a high pro-inflammatory score is associated with a better overall survival regardless of IDH mutation status. Lastly, another surprising conclusion is that the heterogeneity in GAMM number as well as inflammatory phenotype is diverse in IDH-WT GBM where microglia and macrophage content can range from approximately 0 to 70%. This makes a strong argument for precision medicine in GBM, particularly with regards to immunotherapy development and selection. This incredible GAMM variation highlights the importance of future research elucidating what host-tumor interactions lead to this marked diversity, and what mechanisms underlying the difference in MM biology between IDH-MUTs and -WTs can be exploited for therapeutic gain.

## MATERIALS AND METHODS

### Collection of glioblastoma samples

GBM tissue was obtained only from the gadolinium-enhancing region on pre-operative MRIs to preclude areas of gross necrosis (Figure 1) using Medtronic StealthStation Surgical Navigation. This was conducted in 13 patients with previously untreated GBM (9 IDH-WT and 4 IDH-MUT). Patient demographics are detailed in Supplementary Table 1. Surgical excision was performed in all cases by the same neurosurgeon (JJK). Diagnosis of GBM and determination of IDH status was made through histopathological review by board-certified neuropathologists. Tumor specimens and clinical data were obtained as per protocol approved by the local institutional review board and ethics committee and conducted in accordance with national regulations. All patients provided written informed consent.

### Flow cytometry

GBM specimens fresh from the operating room were mechanically and enzymatically digested for one hour using collagenase (4 mg), DNAse (10 mg), and kynurenic acid (4 mg). The resultant single-cell suspensions were passed through a 40 μm strainer and stained with Fixable Viability Stain 510 (1:1000; BD Horizon, #564406), APC-Cy7-CD11b (1:250; BD Pharmingen, #557657) and PE-Cy5-CD45 antibodies (1:250; BD Pharmingen, #555484) or isotype controls (APC-Cy7-Mouse IgG1κ or PE-Cy5-IgG1κ; 1:250; BD Pharmingen, #557873 and #555750, respectively). Samples were analyzed using either the BD FACSAria Fusion Cell Sorter or the Sony SH800. Data was processed using Flowjo software (Treestar). Debris, doublets, and dead cells were excluded using forward and side scatter parameters, and live cell gates. IDH-MUT GBMs were acquired after flow cytometry protocol development, thus microglia and macrophage quantitation of these tumors was only with immunofluorescence.

### Tissue fixation and immunofluorescence

GBM tissue from the operating room was immediately fixed in 4% paraformaldehyde for 15min, embedded in optimal cutting temperature compound, flash frozen and stored at -80°C until use. Cryostat sections were cut at 10μm thickness and slide-mounted. Slides were permeabilized with 0.25% Triton X-100, blocked with 3% bovine serum albumin, and incubated with primary antibodies (Supplementary Table 2) at 4°C overnight. The next day, slides were incubated with secondary antibodies (Supplementary Table 2) and counterstained with Hoechst at room temperature in the dark.

### Imaging and analysis

Immunofluorescent images were obtained with the ImageXpress Micro XLS widefield high-content analysis system and analyzed with MetaXpress 5 software using an in-house developed previously published protocol [21]. Briefly, a rectangular maximal region of interest on slide-mounted sections was selected within tissue borders for imaging (Figure 1). Individual sections of 10μm thickness ranged from approximately 3000–8000 μm in diameter (relatively large sections compared to those available in the literature). Each region of interest was divided into equally-sized rectangular sites of 225 × 168 μm. After image acquisition, sites that included tissue folds, rents, regions of poor focus, and large blood vessels defined as > 25% of the site area were manually excluded (this typically represented < 10-15% of all sites). A no primary antibody control customized to individual GBMs was used to define the threshold above which staining was positive to account for highly variable levels of autofluorescence between GBM specimens. The threshold was set to allow no more than 5% false-positive staining for each fluorophore used. Automated segmentation which allows for single-cell quantitation was performed using the MetaXpress Multiwavelength Cell Scoring Module according to user-defined parameters which included setting the minimum and maximum nuclear and cell diameter as well as the minimum area stained. Microglia and macrophage content was calculated with the formula:

number of Iba1+cells/total nuclei×100%.

The microglia and macrophage inflammatory phenotype was calculated with the formula:

number of inflammatory marker and Iba1double-positive cells/number of Iba1+cells×100%.

Iba1 intensity and surface area were also quantitated in an automated fashion with MetaXpress using the same user-defined parameters. Image acquisition and analysis was performed for three consecutive sections of each GBM.

### Pro-inflammatory profile calculation

The overall inflammatory profile (derived from subtracting the frequency of GAMM anti-inflammatory markers from pro-inflammatory markers), Iba1 intensity, and surface area of microglia and macrophages were chosen *a priori* as indicators of pro-inflammatory activation. To combine these parameters, each dataset was scaled by setting the minimum measured value to “0” and the maximum value to “100”, then summated. Patients that were still alive were given an overall survival as of May 23, 2018. One IDH-mutant patient was lost to follow up and this data was not included in the analysis.

### Statistical analysis

All statistical analyses were performed with Microsoft Excel and Graphpad Prism. Results are shown as mean values ± standard deviations. Statistical significance was calculated by a two-tailed Student’s t-test. Correlation analysis was performed with Pearson’s Correlation Formula. Statistical significance was defined at p < 0.05.

### Analysis of scRNA-seq databases

Treated and untreated IDH-MUT GBM [10] single-cell gene expression levels were downloaded from the NCBI GEO repository under accession GSE89567. Single-cell gene expression levels for untreated IDH-WT GBMs [4] were generated from the NCBI SRA repository under accession SRP079058 by truncating the raw reads to 38bp pair-end data, then applying the same mapping, normalization and filtering procedures as described in Venteicher et al., 2017 [10]. All cells were assessed in the R statistical programming language for cell cycle stage using cyclone [42], and only G1 phase cells were used in subsequent analysis. Each subject’s G1-phase cells were subjected to its own principal component analysis denoising procedure based on variance trends [53]. Denoised data from all three subjects were combined into a master dataset, and clustered based on Ward D2 criterion [54] for genes’ log read counts. Dynamic Tree Cutting in R[55] was used with default parameters to generate the final cluster assignments. The “findMarkersˮ method of Scialdone et al. (2015)[42], was used to identify significantly differentially expressed genes (FDR < 1 × 10-20) in each cluster. Genes from each cluster that were upregulated at least 2-fold were submitted to Ingenuity Pathway Analysis (IPA; Qiagen, Redwood City, CA, USA) for canonical pathway and bespoke gene list enrichment analysis. FDR p-value correction was applied to all IPA results.

### Generation of gene lists and gene enrichment analysis

Lists of genes that were differentially expressed between microglia and macrophages, and pro-versus anti-inflammatory genes, were collected from all available human GBM single-cell RNA sequencing studies as of June 18, 2018 (Supplementary Table 3). Four manuscripts were included using these search criteria [4, 9, 11]. Duplicates were removed. If not already present, genes representing the six inflammatory markers investigated in this manuscript were added (*CD68*, *HLA-A*, *-B*, *-C*, *TNF*, *CD163*, *IL10*, *TGFB2*). Differentially expressed genes generated in the aforementioned scRNA-seq were then cross-referenced with the curated gene lists and compared amongst clusters.

## SUPPLEMENTARY MATERIALS FIGURES AND TABLES









## Abbreviations

GBM: glioblastoma
IDH: isocitrate dehydrogenase
WT: wildtype
MUT: mutant
scRNA-seq: single-cell RNA-sequencing
GAMM: glioblastoma-associated microglia and macrophages
