# Supplementary material for: Differential microglia and macrophage profiles in human IDH-mutant and -wild type glioblastoma
Source: Oncotarget. 2019 May 3;10(33):3129–43. doi: 10.18632/oncotarget.26863 (PMC6517100; doi:10.18632/oncotarget.26863)
Supplement: Supplementary file 1 [file oncotarget-10-3129-s001.pdf]

Differential microglia and macrophage profiles in human IDH-mutant and -wildtype glioblastoma

SUPPLEMENTARY MATERIALS

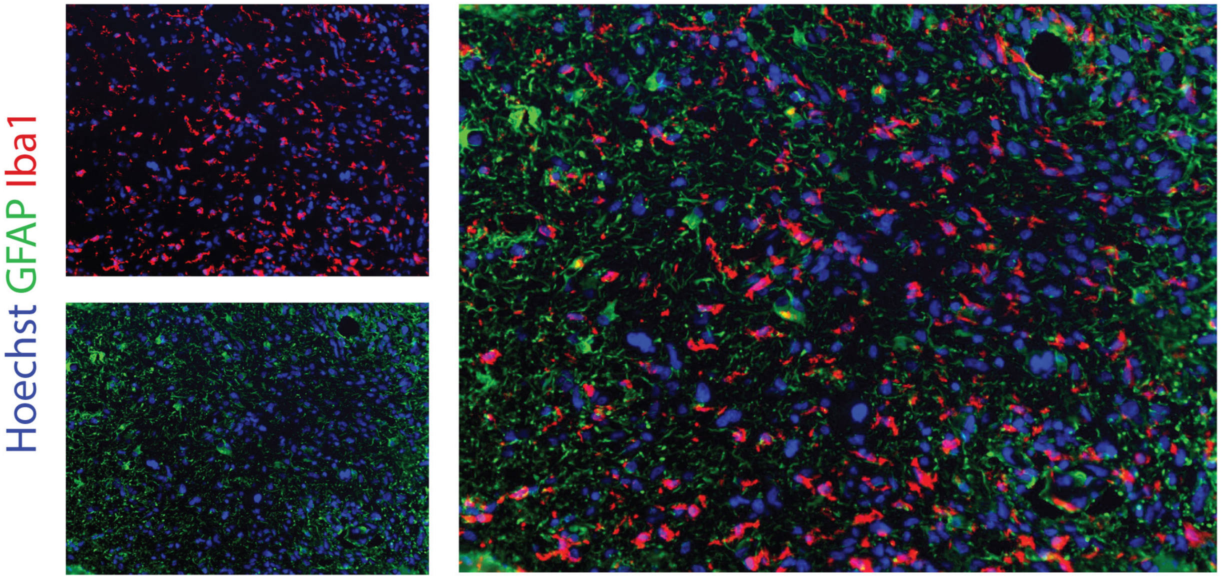

Supplementary Figure 1: A representative image of a human GBM specimen double-stained with the astrocytic marker, GFAP, and the pan-microglia/macrophage marker, Iba1. GFAP and Iba1 do not co-label. GFAP = glial fibrillary acidic protein; Iba1 = ionized calcium-binding adaptor molecule 1.

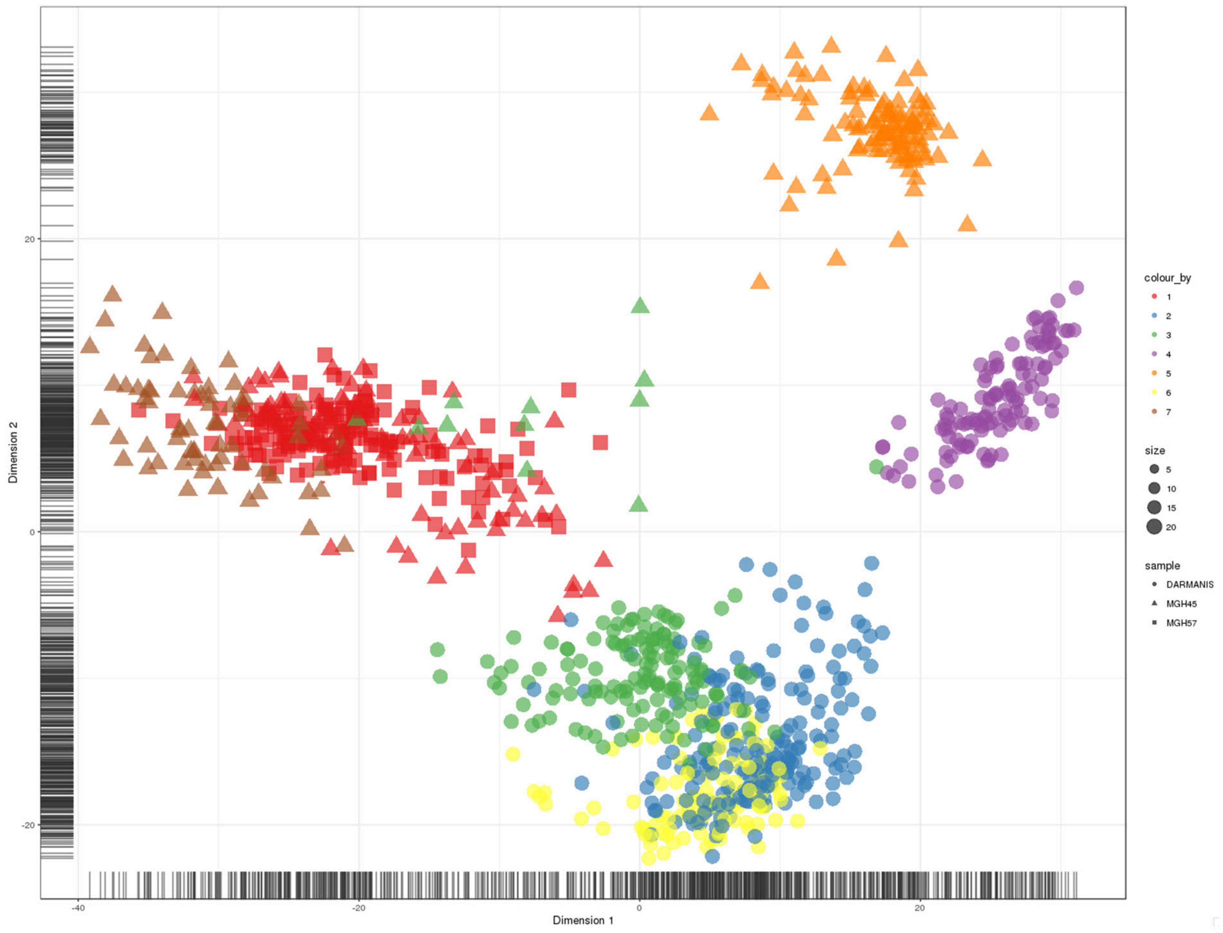

Supplementary Figure 2: PCA dot plot demonstrating cell clusters. Similar to the cell clusters generated using tSNE, Clusters 2, 3, and 6 clustered together as well as Clusters 1 and 7. Interestingly, in IPA analysis the former group represented non-immune, neoplastic cells while Clusters 1 and 7 were associated with GAMM.

Supplementary Table 1: Clinical demographics for newly diagnosed, previously untreated glioblastoma patients in this study. IDH, isocitrate dehydrogenase; GBM, glioblastoma; WT, wild type; MUT, mutant.

Tumor ID	Patient's sex	Patient's age	Diagnosis	IDH status	ATRX status	Time to progression (days)	Overall survival (days)
A	M	34	GBM	WT	WT	131	296
B	M	68	GBM	WT	WT	45	151
C	M	58	GBM	WT	WT	257	614
D	M	61	GBM	WT	WT	239	Still alive
E	M	63	GBM	WT	WT	151	381
F	F	60	GBM	WT	WT	264	340
G	F	60	GBM	WT	WT	105	439
H	M	49	GBM	WT	WT	92	326
I	M	65	GBM	WT	WT	62	Still alive
J	M	33	GBM	MUT	MUT	No progression	Still alive
K	F	39	GBM	MUT	MUT	990	Still alive
L	M	37	GBM	MUT	MUT	305	628
M	M	49	GBM	MUT	MUT	Lost to follow up	Lost to follow up

Supplementary Table 2: Primary and secondary antibodies

Type of antibody	Antigen/fluorophore	Company	Catalog number	Concentration
Primary	Iba1	Wako	019-19741	1:500
Primary	CD68	Dako	M 0876	1:100
Primary	HLA-A, -B, -C	abcam	ab70328	1:100
Primary	TNF	abcam	ab1793	1:50
Primary	CD163	Novus Biologicals	NB1 10-59935	1:50
Primary	IL10	Santa Cruz Biotechnology	sc-8438	1:50
Primary	TGFB2	abcam	ab36495	1:50
Secondary	FITC	Jackson ImmunoResearch	115-096-003	1:200
Secondary	Rhodamine	Jackson ImmunoResearch	111-025-045	1:100
Secondary	Alexa-647	Jackson ImmunoResearch	711-605-152	1:200

Supplementary Table 3: Curated microglia, macrophage, pro- and anti-inflammatory gene lists.

See Supplementary File 1

Supplementary Table 4: Gene enrichment lists for GAMM clusters.

See Supplementary File 2

Supplementary Table 5: Bespoke IPA analysis for Clusters 1 to 7. The canonical pathways differentially regulated in each cluster are shown.

See Supplementary File 3
