# Supplementary material for: Differential microglia and macrophage profiles in human IDH-mutant and -wild type glioblastoma
Source: Oncotarget. 2019 May 3;10(33):3129–43. doi: 10.18632/oncotarget.26863 (PMC6517100; doi:10.18632/oncotarget.26863)
Supplement: Supplementary file 2 [file oncotarget-10-3129-s002.doc]

| Microglia |
| --- |
| A2M |
| ABCC9 |
| ACY3 |
| ADAM28 |
| ADRB2 |
| ANKRD44 |
| APPL2 |
| ARGLU1 |
| ARHGAP17 |
| ARHGEF26-AS1 |
| ASTN2 |
| C6orf62 |
| CCDC144B |
| CCL5 |
| CD84 |
| CH25H |
| CSF1R |
| CX3CR1 |
| DAGLB |
| DDX17 |
| DDX5 |
| DHRS9 |
| DIP2A |
| DOCK8 |
| DPY19L2P2 |
| EGR1 |
| EGR2 |
| EGR3 |
| ENTPD1 |
| FBLIM1 |
| FCGR1B |
| FGD2 |
| FMNL3 |
| FOS |
| FRMD4A |
| GLDN |
| GLIPR1L2 |
| GOSR1 |
| GSTM2 |
| HERC2P4 |
| HIST2H2BF |
| HNRNPH1 |
| IFNGR1 |
| IL6ST |
| IPCEF1 |
| ITGAX |
| ITM2B |
| ITPR2 |
| JUN |
| KCNQ1OT1 |
| KIAA1598 |
| L2HGDH |
| LILRA4 |
| LOC284454 |
| LOC286437 |
| LOC643406 |
| LOC646214 |
| LOC731275 |
| LOC91948 |
| LRTOMT |
| MAF |
| MALAT1 |
| MDM4 |
| METTL21A |
| MTFMT |
| NAV3 |
| ODF2L |
| OPHN1 |
| ORC4 |
| P2RY12 |
| P2RY13 |
| PDK4 |
| PLXDC2 |
| POU5F1 |
| PTPRJ |
| RAPGEF6 |
| RASGEF1C |
| RBM6 |
| RHOB |
| RIN2 |
| RTTN |
| SELENBP1 |
| SELPLG |
| SEMA6D |
| SFMBT2 |
| SH3TC1 |
| SHISA9 |
| SLC15A2 |
| SLC1A3 |
| SLC44A2 |
| SNX29 |
| SOCS6 |
| SON |
| SORL1 |
| SPC25 |
| SRGAP2 |
| SRGAP2C |
| SRRM2 |
| SRSF5 |
| ST6GAL1 |
| SYNDIG1 |
| TMEM212 |
| TNFAIP8L1 |
| TRA2A |
| TRAPPC2 |
| ABHD3 |
| ADORA3 |
| ADORA3 |
| ADORA3 |
| ADRB2 |
| ADRB2 |
| AIF1 |
| ARSB |
| ASPH |
| BHLHE41 |
| BHLHE41 |
| BIN1 |
| BIN1 |
| C2 |
| C4orf19 |
| C4orf48 |
| CCL4 |
| CCL4 |
| CD37 |
| CD81 |
| CD9 |
| CDH23 |
| CHST11 |
| CKB |
| CRYBB1 |
| CST3 |
| CTSD |
| CTSF |
| DDAH2 |
| DNAJA4 |
| DNAJB4 |
| DST |
| DYRK4 |
| FAM135A |
| FAM149A |
| FHIT |
| FKBP14 |
| GEM |
| GFOD2 |
| GLUL |
| GPR155 |
| GPR34 |
| GPR34 |
| GPR84 |
| HEXB |
| IFFO1 |
| ITGAM |
| JAM2 |
| KCNMA1 |
| KHDRBS3 |
| KLF2 |
| KLF2 |
| LDHB |
| MAGED1 |
| MAGED2 |
| MAGEH1 |
| MEF2C |
| MLXIPL |
| MRC2 |
| NAV3 |
| NAV3 |
| NFIA |
| OLFML2B |
| OLFML3 |
| OLFML3 |
| OTUD1 |
| P2RY12 |
| P2RY12 |
| P2RY12 |
| PAG1 |
| PDE3B |
| PDGFB |
| PHACTR1 |
| PHYHD1 |
| PLXDC2 |
| PROS1 |
| RAB34 |
| RASGRP3 |
| RGL3 |
| RHOB |
| RHOB |
| SALL1 |
| SALL1 |
| SALL1 |
| SCCPDH |
| SCN1B |
| SCOC |
| SERPINE1 |
| SIGLEC8 |
| SIGLEC8 |
| SIPA1L2 |
| SLC1A3 |
| SLC1A3 |
| SLC2A5 |
| SLC2A5 |
| SORT1 |
| SPNS2 |
| SPRY1 |
| SPRY1 |
| ST3GAL6 |
| SUSD3 |
| SYNGR1 |
| TAL1 |
| TAL1 |
| TCEAL1 |
| TIAM1 |
| TLN2 |
| TMEM119 |
| TMEM119 |
| TMEM63A |
| TNF |
| TSC22D2 |
| TSC22D3 |
| TSPAN7 |
| XRCC5 |

| Macrophage |
| --- |
| ACSL1 |
| BCL2A1 |
| BHLHE40 |
| C1orf162 |
| CD163 |
| CD74 |
| COTL1 |
| DPYD |
| DUSP2 |
| EEF1A1 |
| EEF1G |
| EMR2 |
| ENO1 |
| F13A1 |
| FAM26F |
| FCER1G |
| FCGBP |
| FCGR3A |
| FOSL2 |
| FTH1 |
| GAPDH |
| GNG10 |
| GPR84 |
| GSTO1 |
| HAMP |
| HLA-A |
| HLA-B |
| HLA-C |
| HLA-DPA1 |
| HLA-DPB1 |
| HLA-DQA1 |
| HLA-DQA2 |
| HLA-DQB1 |
| HLA-DRA |
| HLA-DRB1 |
| HLA-DRB5 |
| IFI30 |
| IFITM2 |
| IFITM3 |
| LDHA |
| LITAF |
| LMNA |
| METRNL |
| MIF |
| MS4A4A |
| MS4A6A |
| MTHFD2 |
| NAMPT |
| NME2 |
| NPC2 |
| PDE4B |
| PLAUR |
| PLEK |
| PLTP |
| PPA1 |
| PTPN1 |
| RAB20 |
| RNASE2 |
| RPL10 |
| RPL10A |
| RPL12 |
| RPL17 |
| RPL18A |
| RPL19 |
| RPL23 |
| RPL27 |
| RPL29 |
| RPL30 |
| RPL37A |
| RPL38 |
| RPL5 |
| RPL7 |
| RPL7A |
| RPL8 |
| RPL9 |
| RPLP0 |
| RPLP1 |
| RPLP2 |
| RPS10 |
| RPS12 |
| RPS13 |
| RPS18 |
| RPS2 |
| RPS23 |
| RPS24 |
| RPS27A |
| RPS3 |
| RPS3A |
| RPS4X |
| RPS5 |
| RPS8 |
| RPS9 |
| RPSA |
| S100A9 |
| SERPINA1 |
| SLC7A5 |
| SMIM3 |
| SOCS3 |
| SOD2 |
| TAGLN2 |
| TGFBI |
| THBD |
| TMEM176B |
| TMSB10 |
| TPT1 |
| TSPO |
| TYMP |
| VAMP8 |
| ADAMTSL4 |
| ALPK1 |
| ANXA1 |
| ANXA11 |
| AOAH |
| AP2A2 |
| APH1B |
| ARG2 |
| ARG2 |
| ARHGAP15 |
| ATP8B4 |
| BEX4 |
| BHLHE40 |
| BHLHE40 |
| CARD11 |
| CCDC125 |
| CCL22 |
| CCR1 |
| CD14 |
| CD300C |
| CD40 |
| CD74 |
| CD74 |
| CD93 |
| CD93 |
| CDK2AP2 |
| CIB1 |
| CIB1 |
| CIITA |
| CIITA |
| CLEC4A |
| CLEC9A |
| COQ10B |
| CREM |
| CREM |
| CRIP1 |
| CSF2RB |
| CTDSP1 |
| CWC25 |
| CXCL16 |
| CXCR4 |
| CYBB |
| CYBB |
| CYP27A1 |
| CYTH1 |
| CYTH1 |
| CYTIP |
| DCK |
| DEPTOR |
| DNASE1L1 |
| DOK3 |
| DOK3 |
| DSE |
| DSE |
| EMB |
| EMB |
| ESYT1 |
| EVI2B |
| FAM49A |
| FAM49A |
| FAS |
| FEM1C |
| FGR |
| FGR |
| FOSL2 |
| FOSL2 |
| FPR3 |
| FPR3 |
| FXYD5 |
| FXYD5 |
| GAB3 |
| GPR132 |
| GPR132 |
| GPR65 |
| GPR65 |
| GSN |
| GYPC |
| HLA-DMB |
| HLA-DMB |
| HLA-DQA1 |
| HLA-DQA1 |
| HLA-DRB5 |
| HLA-DRB5 |
| HPCAL1 |
| IFITM2 |
| IFITM2 |
| IL10 |
| IL10 |
| IL1RN |
| IL1RN |
| IQGAP1 |
| IQGAP1 |
| IRS2 |
| ISY1 |
| ITGA4 |
| ITGA4 |
| JAK2 |
| KLRD1 |
| KYNU |
| KYNU |
| LRRC8C |
| LYZ |
| LYZ |
| MALT1 |
| MAP4K1 |
| MAPKAPK3 |
| MEFV |
| METRNL |
| METRNL |
| MKL1 |
| MNDA |
| MS4A6A |
| MS4A6A |
| MS4A7 |
| MS4A7 |
| MTHFR |
| MTSS1 |
| MXD1 |
| MXD1 |
| MYOF |
| NCOA7 |
| NET1 |
| NFIL3 |
| NFIL3 |
| NFKBIE |
| NR4A2 |
| NR4A3 |
| NUP98 |
| OSBPL3 |
| P2RX5-TAX1BP3 |
| PDE4B |
| PDE4B |
| PDE8A |
| PDGFC |
| PILRB |
| PIM1 |
| PIM1 |
| PLA2G7 |
| PLAC8 |
| PLAC8 |
| PLBD1 |
| PLBD1 |
| PLEKHM1 |
| PLTP |
| PLTP |
| PLXND1 |
| PMAIP1 |
| PMVK |
| PQLC1 |
| PQLC3 |
| PQLC3 |
| PRDM1 |
| PRKCH |
| PTPN7 |
| PTPN7 |
| RASGRP4 |
| RBMS1 |
| RELL1 |
| RGS1 |
| RGS18 |
| RIN3 |
| RUNX2 |
| S100A11 |
| S100A11 |
| S100A8 |
| S100A9 |
| SAMHD1 |
| SAMHD1 |
| SELL |
| SERPINB9 |
| SH3BGRL |
| SH3BGRL |
| SIK1 |
| SIRPB1 |
| SPINT2 |
| SPINT2 |
| SPTY2D1 |
| ST3GAL1 |
| ST8SIA4 |
| STK17B |
| STOM |
| SYNGR2 |
| SYNGR2 |
| SYTL3 |
| TEP1 |
| TFEC |
| TGFBI |
| TGFBI |
| THBD |
| THBD |
| TINF2 |
| TIPARP |
| TLR5 |
| TMEM123 |
| TMEM123 |
| TNFAIP2 |
| TNFSF13 |
| TNFSF13 |
| TREM1 |
| TREM1 |
| UNC119 |
| VOPP1 |
| VOPP1 |
| VPS37B |
| ZC3H12D |
