# Supplementary material for: Differential microglia and macrophage profiles in human IDH-mutant and -wild type glioblastoma
Source: Oncotarget. 2019 May 3;10(33):3129–43. doi: 10.18632/oncotarget.26863 (PMC6517100; doi:10.18632/oncotarget.26863)
Supplement: Supplementary file 3 [file oncotarget-10-3129-s003.doc]

Cluster 1 microglia

| © 2000-2018 QIAGEN. All rights reserved. |  |  |  |  |  |  |  |  |  |  |  |  |
| --- | --- | --- | --- | --- | --- | --- | --- | --- | --- | --- | --- | --- |
| Symbol | Entrez Gene Name | Gene Symbol - human (HUGO / HGNC / Entrez Gene)/Gene Symbol - mouse (Entrez Gene)/Gene Symbol - rat (Entrez Gene) | Expr Log Ratio | Expr False Discovery Rate (q-value) | Networks | Location | Type(s) | Biomarker Application(s) | Drug(s) | Entrez Gene ID for Human | Entrez Gene ID for Mouse | Entrez Gene ID for Rat |
| CKB | creatine kinase B | CKB | 2.97 | 1.94E-121 | 5 | Cytoplasm | kinase | safety |  | 1152 | 12709 | 24264 |
| HNRNPH1 | heterogeneous nuclear ribonucleoprotein H1 | HNRNPH1 | 2.011 | 3.28E-64 | 1 | Nucleus | other | diagnosis |  | 3187 | 59013 |  |
| ORC4 | origin recognition complex subunit 4 | ORC4 | 1.884 | 4.12E-115 | 9 | Nucleus | other |  |  | 5000 | 26428 | 295596 |
| SHISA9 | shisa family member 9 | SHISA9 | 1.792 | 4.93E-151 | 18 | Plasma Membrane | other |  |  | 729993 | 72555 | 100361134 |
| OPHN1 | oligophrenin 1 | OPHN1 | 1.722 | 2.22E-21 | 7 | Cytoplasm | other |  |  | 4983 | 94190 | 312108 |
| TMEM212 | transmembrane protein 212 | TMEM212 | 1.703 | 4.26E-170 |  | Other | other |  |  | 389177 | 208613 | 499586 |
| TRA2A | transformer 2 alpha homolog | TRA2A | 1.697 | 2.77E-54 | 1 | Nucleus | other |  |  | 29896 | 101214 | 500116 |
| RIN2 | Ras and Rab interactor 2 | RIN2 | 1.675 | 9.44E-58 | 19 | Cytoplasm | other |  |  | 54453 | 74030 | 311494 |
| ASTN2 | astrotactin 2 | ASTN2 | 1.655 | 1.45E-58 | 16 | Cytoplasm | other |  |  | 23245 | 56079 | 100361323 |
| MAGEH1 | MAGE family member H1 | MAGEH1 | 1.391 | 2.22E-34 | 2 | Cytoplasm | other |  |  | 28986 | 75625 | 367767 |
| DDX17 | DEAD-box helicase 17 | DDX17 | 1.332 | 1.75E-52 | 1 | Nucleus | enzyme | unspecified application |  | 10521 | 67040 |  |
| ODF2L | outer dense fiber of sperm tails 2 like | ODF2L | 1.323 | 7.88E-82 | 2,17 | Cytoplasm | other |  |  | 57489 | 52184 | 685425 |
| NFIA | nuclear factor I A | NFIA | 1.292 | 1.66E-73 | 11 | Nucleus | transcription regulator |  |  | 4774 | 18027 | 25492 |
| KCNQ1OT1 | KCNQ1 opposite strand/antisense transcript 1 (non-protein coding) | KCNQ1OT1 | 1.283 | 3.8E-149 | 17 | Other | other |  |  | 10984 | 63830 |  |
| SPC25 | SPC25, NDC80 kinetochore complex component | SPC25 | 1.28 | 3.16E-76 | 1 | Cytoplasm | other |  |  | 57405 | 66442 | 295661 |
| JAM2 | junctional adhesion molecule 2 | JAM2 | 1.273 | 5.51E-27 | 6 | Plasma Membrane | other |  |  | 58494 | 67374 | 619374 |
| C6orf62 | chromosome 6 open reading frame 62 | C6orf62 | 1.257 | 2.74E-44 | 12 | Other | other |  |  | 81688 | 79555 |  |
| L2HGDH | L-2-hydroxyglutarate dehydrogenase | L2HGDH | 1.175 | 2.75E-86 | 14 | Cytoplasm | enzyme |  |  | 79944 | 217666 | 314196 |
| TSPAN7 | tetraspanin 7 | TSPAN7 | 1.056 | 1.85E-44 | 13 | Plasma Membrane | other |  |  | 7102 | 21912 | 363447 |
| ABCC9 | ATP binding cassette subfamily C member 9 | ABCC9 | 1.03 | 4.5E-88 | 7 | Plasma Membrane | ion channel |  |  | 10060 | 20928 | 25560 |
| SRRM2 | serine/arginine repetitive matrix 2 | SRRM2 | 1.03 | 6.24E-51 | 1 | Nucleus | other |  |  | 23524 |  |  |
| GLIPR1L2 | GLIPR1 like 2 | GLIPR1L2 | 1.015 | 4.51E-109 | 16 | Extracellular Space | other |  |  | 144321 | 67537 | 366890 |

Cluster 1 macrophage

| © 2000-2018 QIAGEN. All rights reserved. |  |  |  |  |  |  |  |  |  |  |  |  |
| --- | --- | --- | --- | --- | --- | --- | --- | --- | --- | --- | --- | --- |
| Symbol | Entrez Gene Name | Gene Symbol - human (HUGO / HGNC / Entrez Gene)/Gene Symbol - mouse (Entrez Gene)/Gene Symbol - rat (Entrez Gene) | Expr Log Ratio | Expr False Discovery Rate (q-value) | Networks | Location | Type(s) | Biomarker Application(s) | Drug(s) | Entrez Gene ID for Human | Entrez Gene ID for Mouse | Entrez Gene ID for Rat |
| RPLP0 | ribosomal protein lateral stalk subunit P0 | RPLP0 | 2.047 | 2.51E-130 | 3 | Cytoplasm | other |  |  | 6175 | 11837 | 64205 |
| RPSA | ribosomal protein SA | RPSA | 2.037 | 5.07E-32 | 3 | Cytoplasm | translation regulator |  |  | 3921 | 16785 | 29236 |
| RPS4X | ribosomal protein S4, X-linked | RPS4X | 1.958 | 6.73E-29 | 3 | Cytoplasm | other | diagnosis |  | 6191 |  |  |
| RPLP1 | ribosomal protein lateral stalk subunit P1 | RPLP1 | 1.885 | 8.49E-92 | 3 | Cytoplasm | other |  |  | 6176 |  | 140661 |
| EEF1G | eukaryotic translation elongation factor 1 gamma | EEF1G | 1.734 | 5.65E-95 | 8 | Cytoplasm | translation regulator |  |  | 1937 | 67160 | 293725 |
| RPL9 | ribosomal protein L9 | RPL9 | 1.729 | 1.92E-91 | 3 | Nucleus | other | diagnosis |  | 6133 | 20005 | 100360449|29257|100364457 |
| TMEM123 | transmembrane protein 123 | TMEM123 | 1.64 | 4.67E-49 | 12 | Plasma Membrane | other |  |  | 114908 | 71929 | 363013 |
| RPL7A | ribosomal protein L7a | RPL7A | 1.601 | 7.08E-71 | 3 | Cytoplasm | other |  |  | 6130 | 27176 | 296596 |
| RPL5 | ribosomal protein L5 | RPL5 | 1.595 | 2.7E-66 | 3 | Cytoplasm | other |  |  | 6125 | 100503670 | 81763 |
| BEX4 | brain expressed X-linked 4 | BEX4 | 1.462 | 6.1E-35 | 16 | Cytoplasm | other |  |  | 56271 | 406217 | 501624 |
| RPS3A | ribosomal protein S3A | RPS3A | 1.448 | 2.09E-77 | 3 | Nucleus | other |  |  | 6189 |  | 100365839 |
| PPA1 | pyrophosphatase (inorganic) 1 | PPA1 | 1.43 | 8.16E-35 | 9,16 | Cytoplasm | enzyme |  |  | 5464 | 67895 | 294504 |
| RPL19 | ribosomal protein L19 | RPL19 | 1.117 | 4.3E-51 | 3 | Cytoplasm | other |  |  | 6143 | 19921 | 81767 |
| RPL23 | ribosomal protein L23 | RPL23 | 1.092 | 2.17E-63 | 3 | Cytoplasm | other |  |  | 9349 | 65019 | 29282 |
| RPS27A | ribosomal protein S27a | RPS27A | 1.078 | 4.29E-64 | 3 | Cytoplasm | other |  |  | 6233 | 78294 | 100912032 |
| RPL10 | ribosomal protein L10 | RPL10 | 1.073 | 6.34E-74 | 3 | Cytoplasm | translation regulator |  |  | 6134 | 110954 | 81764 |
| RPS3 | ribosomal protein S3 | RPS3 | 1.063 | 1.62E-55 | 3 | Cytoplasm | enzyme | unspecified application |  | 6188 | 27050 | 140654 |
| EEF1A1 | eukaryotic translation elongation factor 1 alpha 1 | EEF1A1 | 1.049 | 1.35E-87 | 9 | Cytoplasm | translation regulator |  |  | 1915 | 13627 | 171361 |
| RPS23 | ribosomal protein S23 | RPS23 | 1.038 | 2.78E-31 | 3 | Cytoplasm | translation regulator |  |  | 6228 | 66475 | 124323 |

Cluster 1 pro-inflammatory

| © 2000-2018 QIAGEN. All rights reserved. |  |  |  |  |  |  |  |  |  |  |  |  |
| --- | --- | --- | --- | --- | --- | --- | --- | --- | --- | --- | --- | --- |
| Symbol | Entrez Gene Name | Gene Symbol - human (HUGO / HGNC / Entrez Gene)/Gene Symbol - mouse (Entrez Gene)/Gene Symbol - rat (Entrez Gene) | Expr Log Ratio | Expr False Discovery Rate (q-value) | Networks | Location | Type(s) | Biomarker Application(s) | Drug(s) | Entrez Gene ID for Human | Entrez Gene ID for Mouse | Entrez Gene ID for Rat |
| CKB | creatine kinase B | CKB | 2.97 | 1.94E-121 | 5 | Cytoplasm | kinase | safety |  | 1152 | 12709 | 24264 |
| SOX2-OT | SOX2 overlapping transcript | SOX2-OT | 2.847 | 2.22E-87 |  | Other | other |  |  | 347689 |  |  |
| GPM6A | glycoprotein M6A | GPM6A | 2.768 | 2.29E-147 | 12 | Plasma Membrane | ion channel |  |  | 2823 | 234267 | 306439 |
| RPS4Y1 | ribosomal protein S4, Y-linked 1 | RPS4Y1 | 2.015 | 3.53E-26 | 3 | Cytoplasm | other | diagnosis,prognosis |  | 6192 | 20102 | 100362640 |
| HNRNPH1 | heterogeneous nuclear ribonucleoprotein H1 | HNRNPH1 | 2.011 | 3.28E-64 | 1 | Nucleus | other | diagnosis |  | 3187 | 59013 |  |
| VIPR2 | vasoactive intestinal peptide receptor 2 | VIPR2 | 1.984 | 2.06E-100 | 13 | Plasma Membrane | G-protein coupled receptor |  |  | 7434 | 22355 | 29555 |
| SHISA9 | shisa family member 9 | SHISA9 | 1.792 | 4.93E-151 | 18 | Plasma Membrane | other |  |  | 729993 | 72555 | 100361134 |
| MTPAP | mitochondrial poly(A) polymerase | MTPAP | 1.741 | 4.28E-73 | 3 | Cytoplasm | enzyme |  |  | 55149 | 67440 | 307050 |
| OPHN1 | oligophrenin 1 | OPHN1 | 1.722 | 2.22E-21 | 7 | Cytoplasm | other |  |  | 4983 | 94190 | 312108 |
| TRA2A | transformer 2 alpha homolog | TRA2A | 1.697 | 2.77E-54 | 1 | Nucleus | other |  |  | 29896 | 101214 | 500116 |
| RIN2 | Ras and Rab interactor 2 | RIN2 | 1.675 | 9.44E-58 | 19 | Cytoplasm | other |  |  | 54453 | 74030 | 311494 |
| MARCKS | myristoylated alanine rich protein kinase C substrate | MARCKS | 1.659 | 1.52E-24 | 5 | Plasma Membrane | other |  |  | 4082 |  |  |
| CTTNBP2 | cortactin binding protein 2 | CTTNBP2 | 1.546 | 4.74E-41 | 7 | Cytoplasm | other |  |  | 83992 | 30785 | 282587 |
| ELAVL4 | ELAV like RNA binding protein 4 | ELAVL4 | 1.518 | 9.91E-40 | 18 | Cytoplasm | other |  |  | 1996 | 15572 | 432358 |
| SRSF6 | serine and arginine rich splicing factor 6 | SRSF6 | 1.453 | 2.44E-31 | 1 | Nucleus | other |  |  | 6431 | 67996 | 362264 |
| CHD7 | chromodomain helicase DNA binding protein 7 | CHD7 | 1.398 | 6E-64 | 11 | Nucleus | enzyme |  |  | 55636 | 320790 | 312974 |
| UGT8 | UDP glycosyltransferase 8 | UGT8 | 1.396 | 2.99E-82 | 4 | Cytoplasm | enzyme |  |  | 7368 | 22239 | 50555 |
| SGCB | sarcoglycan beta | SGCB | 1.306 | 1.36E-45 | 15 | Plasma Membrane | other |  |  | 6443 | 24051 | 680229 |
| KCNQ1OT1 | KCNQ1 opposite strand/antisense transcript 1 (non-protein coding) | KCNQ1OT1 | 1.283 | 3.8E-149 | 17 | Other | other |  |  | 10984 | 63830 |  |
| MAML2 | mastermind like transcriptional coactivator 2 | MAML2 | 1.213 | 1.62E-58 | 11 | Nucleus | transcription regulator |  |  | 84441 |  | 689844 |
| GPR37L1 | G protein-coupled receptor 37 like 1 | GPR37L1 | 1.194 | 3.03E-21 | 16 | Plasma Membrane | G-protein coupled receptor |  |  | 9283 | 171469 | 252939 |
| ZNF248 | zinc finger protein 248 | ZNF248 | 1.125 | 1.04E-36 | 14 | Nucleus | other |  |  | 57209 | 72720 | 500304 |
| ASTN1 | astrotactin 1 | ASTN1 | 1.066 | 1.18E-39 | 16 | Other | other |  |  | 460 | 11899 | 304900 |
| TSPAN7 | tetraspanin 7 | TSPAN7 | 1.056 | 1.85E-44 | 13 | Plasma Membrane | other |  |  | 7102 | 21912 | 363447 |
| NBPF10 (includes others) | NBPF member 20 | NBPF24 | 1.045 | 3.83E-65 | 8 | Other | other |  |  | 55672|101060226|101060684|284565|200030|100132406|400818|100288142|728841|102724250|149013|25832 |  |  |
| PTK2 | protein tyrosine kinase 2 | PTK2 | 1.035 | 1.71E-23 | 9 | Cytoplasm | kinase | diagnosis,disease progression,efficacy,prognosis | BI 853520, CT-707, TPX-0005 | 5747 | 14083 | 25614 |
| ABCC9 | ATP binding cassette subfamily C member 9 | ABCC9 | 1.03 | 4.5E-88 | 7 | Plasma Membrane | ion channel |  |  | 10060 | 20928 | 25560 |
| SRRM2 | serine/arginine repetitive matrix 2 | SRRM2 | 1.03 | 6.24E-51 | 1 | Nucleus | other |  |  | 23524 |  |  |
| DIP2B | disco interacting protein 2 homolog B | DIP2B | 1.011 | 1.88E-30 | 10,12 | Cytoplasm | other |  |  | 57609 | 239667 | 300231 |

Cluster 4 macrophage

| © 2000-2018 QIAGEN. All rights reserved. |  |  |  |  |  |  |  |  |  |  |  |  |
| --- | --- | --- | --- | --- | --- | --- | --- | --- | --- | --- | --- | --- |
| Symbol | Entrez Gene Name | Gene Symbol - human (HUGO / HGNC / Entrez Gene)/Gene Symbol - mouse (Entrez Gene)/Gene Symbol - rat (Entrez Gene) | Expr Log Ratio | Expr False Discovery Rate (q-value) | Networks | Location | Type(s) | Biomarker Application(s) | Drug(s) | Entrez Gene ID for Human | Entrez Gene ID for Mouse | Entrez Gene ID for Rat |
| FCER1G | Fc fragment of IgE receptor Ig | FCER1G | 6.204 | 0 | 5 | Plasma Membrane | transmembrane receptor |  |  | 2207 | 14127 | 25441 |
| CD74 | CD74 molecule | CD74 | 6.174 | 4.15E-229 | 2 | Plasma Membrane | transmembrane receptor |  | milatuzumab | 972 | 16149 | 25599 |
| IFI30 | IFI30, lysosomal thiol reductase | IFI30 | 5.516 | 0 | 3 | Cytoplasm | enzyme |  |  | 10437 | 65972 | 290644 |
| CD14 | CD14 molecule | CD14 | 5.408 | 3.65E-235 | 4 | Plasma Membrane | transmembrane receptor | efficacy,unspecified application |  | 929 | 12475 | 60350 |
| HLA-DRB1 | major histocompatibility complex, class II, DR beta 1 | HLA-DRB1 | 5.25 | 9.38E-294 | 2 | Plasma Membrane | transmembrane receptor |  | apolizumab | 3123 |  |  |
| HLA-DRA | major histocompatibility complex, class II, DR alpha | HLA-DRA | 4.976 | 2.91E-193 | 2 | Plasma Membrane | transmembrane receptor |  |  | 3122 | 100504404 | 294269 |
| TGFBI | transforming growth factor beta induced | TGFBI | 4.658 | 6.91E-144 | 7 | Extracellular Space | other |  |  | 7045 | 21810 | 116487 |
| S100A11 | S100 calcium binding protein A11 | S100A11 | 4.652 | 1.2E-115 | 1 | Cytoplasm | other |  |  | 6282 | 277089 | 445415 |
| S100A9 | S100 calcium binding protein A9 | S100A9 | 4.493 | 7.58E-164 | 1 | Cytoplasm | other | diagnosis,unspecified application |  | 6280 | 20202 | 94195 |
| VAMP8 | vesicle associated membrane protein 8 | VAMP8 | 4.303 | 2.24E-245 | 17 | Plasma Membrane | transporter |  |  | 8673 | 22320 | 83730 |
| NPC2 | NPC intracellular cholesterol transporter 2 | NPC2 | 4.282 | 2.96E-111 | 2 | Extracellular Space | transporter |  |  | 10577 | 67963 | 286898 |
| RGS1 | regulator of G protein signaling 1 | RGS1 | 4.019 | 5.38E-270 | 12 | Plasma Membrane | other |  |  | 5996 | 50778 | 54289 |
| TYMP | thymidine phosphorylase | TYMP | 3.972 | 6.15E-169 | 8 | Extracellular Space | growth factor | diagnosis,efficacy,prognosis,response to therapy |  | 1890 | 72962 | 315219 |
| SYNGR2 | synaptogyrin 2 | SYNGR2 | 3.326 | 1.54E-81 | 3 | Cytoplasm | other |  |  | 9144 | 20973 | 89815 |
| TSPO | translocator protein | TSPO | 3.083 | 2.91E-25 | 17 | Cytoplasm | transmembrane receptor |  | CGS-8216, diazepinomicin, dexamethasone/olanzapine, fluoxetine/olanzapine, estazolam, clorazepate, eszopiclone, temazepam, zolpidem, chlordiazepoxide, lorazepam, olanzapine, triazolam, flumazenil, clonazepam, flurazepam, midazolam, flunitrazepam, alprazolam, zaleplon, SSR180575, PK 11195 | 706 | 12257 | 24230 |
| C1orf162 | chromosome 1 open reading frame 162 | C1orf162 | 3.057 | 1.82E-116 | 17 | Other | transporter |  |  | 128346 | 433638 | 100911379|100363228 |
| S100A8 | S100 calcium binding protein A8 | S100A8 | 3.032 | 9.71E-109 | 1 | Cytoplasm | other | diagnosis,efficacy,unspecified application |  | 6279 | 20201 | 116547 |
| MS4A4A | membrane spanning 4-domains A4A | MS4A4A | 2.896 | 9.84E-145 | 11 | Cytoplasm | other |  |  | 51338 | 666907 |  |
| TMSB10/TMSB4X | thymosin beta 4, X-linked | TMSB4X | 2.894 | 3.8E-80 | 6 | Cytoplasm | other | diagnosis |  | 9168|7114 | 100042318 | 81814|50665 |
| FXYD5 | FXYD domain containing ion transport regulator 5 | FXYD5 | 2.885 | 5.38E-133 | 19 | Plasma Membrane | ion channel |  |  | 53827 | 18301 | 60338 |
| PLAUR | plasminogen activator, urokinase receptor | PLAUR | 2.787 | 2.19E-72 | 8 | Plasma Membrane | transmembrane receptor | diagnosis,disease progression |  | 5329 | 18793 | 50692 |
| FCGR3A/FCGR3B | Fc fragment of IgG receptor IIIa | FCGR3A | 2.779 | 1.05E-154 | 11 | Plasma Membrane | transmembrane receptor |  | IgG, AFM13 | 2214|2215 | 246256 | 304966 |
| MS4A7 | membrane spanning 4-domains A7 | MS4A7 | 2.735 | 2.19E-180 | 21 | Other | other |  |  | 58475 | 109225 | 293744 |
| CD163 | CD163 molecule | CD163 | 2.655 | 5.82E-101 | 5 | Plasma Membrane | transmembrane receptor |  |  | 9332 | 93671 | 312701 |
| RNASE2 | ribonuclease A family member 2 | RNASE2 | 2.642 | 6.79E-72 | 5 | Cytoplasm | enzyme | diagnosis |  | 6036 | 93726|54159 |  |
| SERPINA1 | serpin family A member 1 | SERPINA1 | 2.6 | 1.56E-160 | 8 | Extracellular Space | other | diagnosis,unspecified application |  | 5265 | 20704|20703|20702|20701|20700 | 24648 |
| HLA-DQB1 | major histocompatibility complex, class II, DQ beta 1 | HLA-DQB1 | 2.526 | 6.06E-94 | 2 | Plasma Membrane | other |  |  | 3119 | 14961 | 309622 |
| HAMP | hepcidin antimicrobial peptide | HAMP | 2.516 | 3.8E-47 | 19 | Extracellular Space | other | efficacy | NOX-H94 | 57817 |  |  |
| COTL1 | coactosin like F-actin binding protein 1 | COTL1 | 2.487 | 4.71E-72 | 1 | Cytoplasm | other |  |  | 23406 | 72042 | 361422 |
| MS4A6A | membrane spanning 4-domains A6A | MS4A6A | 2.47 | 8E-135 | 15 | Other | other |  |  | 64231 | 68774 | 361735 |
| TREM1 | triggering receptor expressed on myeloid cells 1 | TREM1 | 2.308 | 2.92E-50 | 4 | Plasma Membrane | transmembrane receptor | efficacy |  | 54210 | 58217 | 301229 |
| GSTO1 | glutathione S-transferase omega 1 | GSTO1 | 2.238 | 1.78E-32 | 7 | Cytoplasm | enzyme | unspecified application |  | 9446 | 14873 | 114846 |
| BCL2A1 | BCL2 related protein A1 | BCL2A1 | 2.231 | 1.61E-71 | 15 | Cytoplasm | other |  |  | 597 | 12044|12045|12047 | 170929 |
| CXCL16 | C-X-C motif chemokine ligand 16 | CXCL16 | 2.224 | 6.75E-44 | 9 | Extracellular Space | cytokine | diagnosis,disease progression,unspecified application |  | 58191 | 66102 | 497942 |
| METRNL | meteorin like, glial cell differentiation regulator | METRNL | 2.11 | 7.48E-89 | 7 | Cytoplasm | other |  |  | 284207 | 210029 | 316842 |
| ACSL1 | acyl-CoA synthetase long chain family member 1 | ACSL1 | 2.048 | 1.49E-43 | 14 | Cytoplasm | enzyme | unspecified application |  | 2180 | 14081 | 25288 |
| FCGBP | Fc fragment of IgG binding protein | FCGBP | 2.047 | 6.23E-67 | 4 | Extracellular Space | other |  |  | 8857 | 215384 | 100303643 |
| HLA-B | major histocompatibility complex, class I, B | HLA-B | 2.034 | 1.28E-93 | 10 | Plasma Membrane | transmembrane receptor | safety |  | 3106 |  |  |
| GSN | gelsolin | GSN | 2.014 | 1.04E-28 | 6 | Extracellular Space | other | disease progression,efficacy |  | 2934 | 227753 | 296654 |
| PLEK | pleckstrin | PLEK | 1.902 | 1.1E-97 | 8 | Cytoplasm | other |  |  | 5341 | 56193 | 364206 |
| FTH1 | ferritin heavy chain 1 | FTH1 | 1.895 | 2.3E-25 | 19 | Cytoplasm | enzyme | unspecified application |  | 2495 | 14319 | 25319|689130 |
| HLA-C | major histocompatibility complex, class I, C | HLA-C | 1.807 | 3.82E-110 | 10 | Plasma Membrane | other | response to therapy |  | 3107 |  |  |
| HLA-DQA1 | major histocompatibility complex, class II, DQ alpha 1 | HLA-DQA1 | 1.773 | 4.31E-90 | 2 | Plasma Membrane | transmembrane receptor |  |  | 3117 | 14960 | 309621 |
| GYPC | glycophorin C (Gerbich blood group) | GYPC | 1.753 | 4.89E-86 | 10 | Plasma Membrane | other | unspecified application |  | 2995 | 71683 | 364837 |
| HLA-DPB1 | major histocompatibility complex, class II, DP beta 1 | HLA-DPB1 | 1.75 | 2.4E-110 | 2 | Plasma Membrane | transmembrane receptor |  |  | 3115 |  |  |
| NR4A2 | nuclear receptor subfamily 4 group A member 2 | NR4A2 | 1.641 | 1.71E-72 | 19 | Nucleus | ligand-dependent nuclear receptor |  |  | 4929 | 18227 | 54278 |
| IL1RN | interleukin 1 receptor antagonist | IL1RN | 1.547 | 8.11E-56 | 7 | Extracellular Space | cytokine | efficacy |  | 3557 | 16181 | 60582 |
| TNFSF13 | TNF superfamily member 13 | TNFSF13 | 1.409 | 8.56E-35 | 9 | Extracellular Space | cytokine |  |  | 8741 | 69583 | 287437 |
| IFITM2 | interferon induced transmembrane protein 2 | IFITM2 | 1.381 | 1.37E-43 | 12 | Cytoplasm | other | prognosis |  | 10581 | 80876 | 114709 |
| RAB20 | RAB20, member RAS oncogene family | RAB20 | 1.352 | 1.21E-42 | 1 | Cytoplasm | enzyme |  |  | 55647 | 19332 | 689377 |
| CRIP1 | cysteine rich protein 1 | CRIP1 | 1.345 | 4.19E-36 | 12 | Cytoplasm | other | unspecified application |  | 1396 | 12925 | 691657 |
| RPS18 | ribosomal protein S18 | RPS18 | 1.328 | 3.64E-29 | 16 | Cytoplasm | other |  |  | 6222 | 20084 | 100360679|294282 |
| CALHM6 | calcium homeostasis modulator family member 6 | FAM26F | 1.266 | 4.7E-51 | 3 | Other | other |  |  | 441168 | 215900 | 294430 |
| DOK3 | docking protein 3 | DOK3 | 1.241 | 1.95E-50 | 9 | Cytoplasm | other |  |  | 79930 | 27261 | 306760 |
| ANXA1 | annexin A1 | ANXA1 | 1.24 | 1.38E-34 | 8 | Plasma Membrane | enzyme | diagnosis,prognosis,unspecified application | hydrocortisone, hydrocortisone/prednisone, hydrocortisone/mitoxantrone | 301 | 16952 | 25380 |
| SPINT2 | serine peptidase inhibitor, Kunitz type 2 | SPINT2 | 1.177 | 3.75E-21 | 18 | Extracellular Space | other | diagnosis |  | 10653 | 20733 | 292770 |
| ANXA11 | annexin A11 | ANXA11 | 1.175 | 1.75E-52 | 1 | Nucleus | other |  |  | 311 | 11744 | 290527 |
| HLA-DRB5 | major histocompatibility complex, class II, DR beta 5 | HLA-DRB5 | 1.157 | 9.03E-67 | 2 | Plasma Membrane | transmembrane receptor |  |  | 3127 | 14969 | 294270 |
| SERPINB9 | serpin family B member 9 | SERPINB9 | 1.153 | 3.95E-81 | 5 | Cytoplasm | other |  |  | 5272 | 20723 | 361241 |
| LITAF | lipopolysaccharide induced TNF factor | LITAF | 1.065 | 5.31E-35 | 4 | Nucleus | transcription regulator |  |  | 9516 | 56722 | 65161 |
| MNDA | myeloid cell nuclear differentiation antigen | MNDA | 1.023 | 2.19E-35 | 14 | Nucleus | other |  |  | 4332 |  |  |

Cluster 4 anti-inflammatory

| © 2000-2018 QIAGEN. All rights reserved. |  |  |  |  |  |  |  |  |  |  |  |  |
| --- | --- | --- | --- | --- | --- | --- | --- | --- | --- | --- | --- | --- |
| Symbol | Entrez Gene Name | Gene Symbol - human (HUGO / HGNC / Entrez Gene)/Gene Symbol - mouse (Entrez Gene)/Gene Symbol - rat (Entrez Gene) | Expr Log Ratio | Expr False Discovery Rate (q-value) | Networks | Location | Type(s) | Biomarker Application(s) | Drug(s) | Entrez Gene ID for Human | Entrez Gene ID for Mouse | Entrez Gene ID for Rat |
| FCER1G | Fc fragment of IgE receptor Ig | FCER1G | 6.204 | 0 | 5 | Plasma Membrane | transmembrane receptor |  |  | 2207 | 14127 | 25441 |
| CD74 | CD74 molecule | CD74 | 6.174 | 4.15E-229 | 2 | Plasma Membrane | transmembrane receptor |  | milatuzumab | 972 | 16149 | 25599 |
| TYROBP | TYRO protein tyrosine kinase binding protein | TYROBP | 6.03 | 0 | 2 | Plasma Membrane | transmembrane receptor |  |  | 7305 | 22177 | 361537 |
| C1QB | complement C1q B chain | C1QB | 5.667 | 0 | 1 | Extracellular Space | peptidase |  |  | 713 | 12260 | 29687 |
| IFI30 | IFI30, lysosomal thiol reductase | IFI30 | 5.516 | 0 | 3 | Cytoplasm | enzyme |  |  | 10437 | 65972 | 290644 |
| ALOX5AP | arachidonate 5-lipoxygenase activating protein | ALOX5AP | 5.472 | 0 | 1 | Plasma Membrane | other |  |  | 241 | 11690 | 29624 |
| CD14 | CD14 molecule | CD14 | 5.408 | 3.65E-235 | 4 | Plasma Membrane | transmembrane receptor | efficacy,unspecified application |  | 929 | 12475 | 60350 |
| APOC1 | apolipoprotein C1 | APOC1 | 5.293 | 1.15E-262 | 7 | Extracellular Space | transporter | prognosis,unspecified application |  | 341 |  |  |
| HLA-DRB1 | major histocompatibility complex, class II, DR beta 1 | HLA-DRB1 | 5.25 | 9.38E-294 | 2 | Plasma Membrane | transmembrane receptor |  | apolizumab | 3123 |  |  |
| C1QA | complement C1q A chain | C1QA | 5.173 | 0 | 1 | Extracellular Space | peptidase |  |  | 712 | 12259 | 298566 |
| CYBA | cytochrome b-245 alpha chain | CYBA | 5.033 | 0 | 1 | Cytoplasm | enzyme |  |  | 1535 | 13057 | 79129 |
| HLA-DRA | major histocompatibility complex, class II, DR alpha | HLA-DRA | 4.976 | 2.91E-193 | 2 | Plasma Membrane | transmembrane receptor |  |  | 3122 | 100504404 | 294269 |
| C1QC | complement C1q C chain | C1QC | 4.727 | 0 | 1 | Extracellular Space | peptidase |  |  | 714 | 12262 | 362634 |
| TGFBI | transforming growth factor beta induced | TGFBI | 4.658 | 6.91E-144 | 7 | Extracellular Space | other |  |  | 7045 | 21810 | 116487 |
| S100A11 | S100 calcium binding protein A11 | S100A11 | 4.652 | 1.2E-115 | 1 | Cytoplasm | other |  |  | 6282 | 277089 | 445415 |
| S100A9 | S100 calcium binding protein A9 | S100A9 | 4.493 | 7.58E-164 | 1 | Cytoplasm | other | diagnosis,unspecified application |  | 6280 | 20202 | 94195 |
| VSIG4 | V-set and immunoglobulin domain containing 4 | VSIG4 | 4.435 | 6.92E-265 | 16 | Plasma Membrane | other |  |  | 11326 | 278180 | 312102 |
| VAMP8 | vesicle associated membrane protein 8 | VAMP8 | 4.303 | 2.24E-245 | 17 | Plasma Membrane | transporter |  |  | 8673 | 22320 | 83730 |
| NPC2 | NPC intracellular cholesterol transporter 2 | NPC2 | 4.282 | 2.96E-111 | 2 | Extracellular Space | transporter |  |  | 10577 | 67963 | 286898 |
| CAPG | capping actin protein, gelsolin like | CAPG | 4.213 | 4.75E-145 | 6 | Nucleus | other |  |  | 822 | 12332 | 297339 |
| TYMP | thymidine phosphorylase | TYMP | 3.972 | 6.15E-169 | 8 | Extracellular Space | growth factor | diagnosis,efficacy,prognosis,response to therapy |  | 1890 | 72962 | 315219 |
| SLC11A1 | solute carrier family 11 member 1 | SLC11A1 | 3.968 | 4.78E-231 | 2,3 | Plasma Membrane | transporter |  |  | 6556 | 18173 | 316519 |
| AIF1 | allograft inflammatory factor 1 | AIF1 | 3.861 | 0 | 3 | Nucleus | other |  |  | 199 | 11629 | 29427 |
| LGALS1 | galectin 1 | LGALS1 | 3.621 | 5.56E-92 | 4 | Extracellular Space | other | diagnosis,prognosis | OTX008 | 3956 | 16852 | 56646 |
| FPR1 | formyl peptide receptor 1 | FPR1 | 3.548 | 9E-139 | 12,20 | Plasma Membrane | G-protein coupled receptor |  |  | 2357 | 14293 | 292409 |
| UCP2 | uncoupling protein 2 | UCP2 | 3.389 | 2.1E-132 | 14 | Cytoplasm | transporter |  |  | 7351 | 22228 | 54315 |
| SYNGR2 | synaptogyrin 2 | SYNGR2 | 3.326 | 1.54E-81 | 3 | Cytoplasm | other |  |  | 9144 | 20973 | 89815 |
| S100A4 | S100 calcium binding protein A4 | S100A4 | 3.24 | 1.91E-145 | 1 | Cytoplasm | other | disease progression |  | 6275 | 20198 | 24615 |
| GPR183 | G protein-coupled receptor 183 | GPR183 | 3.205 | 1.74E-171 | 3 | Plasma Membrane | G-protein coupled receptor |  |  | 1880 | 321019 | 679975 |
| STAB1 | stabilin 1 | STAB1 | 3.132 | 2.38E-231 | 9 | Plasma Membrane | transporter |  |  | 23166 | 192187 | 100363145 |
| ITGB2 | integrin subunit beta 2 | ITGB2 | 3.101 | 7.25E-225 | 12 | Plasma Membrane | transmembrane receptor |  |  | 3689 | 16414 | 309684 |
| HCST | hematopoietic cell signal transducer | HCST | 3.088 | 4.58E-157 | 2,20 | Plasma Membrane | transmembrane receptor |  |  | 10870 | 23900 | 474146 |
| TSPO | translocator protein | TSPO | 3.083 | 2.91E-25 | 17 | Cytoplasm | transmembrane receptor |  | CGS-8216, diazepinomicin, dexamethasone/olanzapine, fluoxetine/olanzapine, estazolam, clorazepate, eszopiclone, temazepam, zolpidem, chlordiazepoxide, lorazepam, olanzapine, triazolam, flumazenil, clonazepam, flurazepam, midazolam, flunitrazepam, alprazolam, zaleplon, SSR180575, PK 11195 | 706 | 12257 | 24230 |
| C1orf162 | chromosome 1 open reading frame 162 | C1orf162 | 3.057 | 1.82E-116 | 17 | Other | transporter |  |  | 128346 | 433638 | 100911379|100363228 |
| S100A8 | S100 calcium binding protein A8 | S100A8 | 3.032 | 9.71E-109 | 1 | Cytoplasm | other | diagnosis,efficacy,unspecified application |  | 6279 | 20201 | 116547 |
| PYCARD | PYD and CARD domain containing | PYCARD | 2.996 | 1.01E-127 | 12 | Cytoplasm | transcription regulator | diagnosis |  | 29108 | 66824 | 282817 |
| GMFG | glia maturation factor gamma | GMFG | 2.981 | 2.89E-146 | 2 | Cytoplasm | growth factor |  |  | 9535 | 100040018|63986 | 113940 |
| MS4A4A | membrane spanning 4-domains A4A | MS4A4A | 2.896 | 9.84E-145 | 11 | Cytoplasm | other |  |  | 51338 | 666907 |  |
| TMSB10/TMSB4X | thymosin beta 4, X-linked | TMSB4X | 2.894 | 3.8E-80 | 6 | Cytoplasm | other | diagnosis |  | 9168|7114 | 100042318 | 81814|50665 |
| FXYD5 | FXYD domain containing ion transport regulator 5 | FXYD5 | 2.885 | 5.38E-133 | 19 | Plasma Membrane | ion channel |  |  | 53827 | 18301 | 60338 |
| ARPC1B | actin related protein 2/3 complex subunit 1B | ARPC1B | 2.874 | 5.35E-104 | 13,14 | Cytoplasm | other |  |  | 10095 | 11867 | 54227 |
| CORO1A | coronin 1A | CORO1A | 2.83 | 3.42E-127 | 1 | Cytoplasm | other |  |  | 11151 | 12721 | 155151 |
| HLA-DMA | major histocompatibility complex, class II, DM alpha | HLA-DMA | 2.794 | 3.13E-167 | 2 | Plasma Membrane | transmembrane receptor |  |  | 3108 | 14998 |  |
| PLAUR | plasminogen activator, urokinase receptor | PLAUR | 2.787 | 2.19E-72 | 8 | Plasma Membrane | transmembrane receptor | diagnosis,disease progression |  | 5329 | 18793 | 50692 |
| FCGR3A/FCGR3B | Fc fragment of IgG receptor IIIa | FCGR3A | 2.779 | 1.05E-154 | 11 | Plasma Membrane | transmembrane receptor |  | IgG, AFM13 | 2214|2215 | 246256 | 304966 |
| PLIN2 | perilipin 2 | PLIN2 | 2.775 | 1.59E-56 | 7 | Plasma Membrane | other | disease progression |  | 123 | 11520 | 298199 |
| CLIC1 | chloride intracellular channel 1 | CLIC1 | 2.766 | 3.91E-95 | 1 | Nucleus | ion channel |  |  | 1192 | 114584 | 406864 |
| MS4A7 | membrane spanning 4-domains A7 | MS4A7 | 2.735 | 2.19E-180 | 21 | Other | other |  |  | 58475 | 109225 | 293744 |
| NINJ1 | ninjurin 1 | NINJ1 | 2.67 | 3E-49 | 3 | Plasma Membrane | other | disease progression |  | 4814 | 18081 | 25338 |
| CD163 | CD163 molecule | CD163 | 2.655 | 5.82E-101 | 5 | Plasma Membrane | transmembrane receptor |  |  | 9332 | 93671 | 312701 |
| GRN | granulin precursor | GRN | 2.653 | 5.87E-48 | 9 | Extracellular Space | growth factor |  |  | 2896 | 14824 | 29143 |
| RNASE2 | ribonuclease A family member 2 | RNASE2 | 2.642 | 6.79E-72 | 5 | Cytoplasm | enzyme | diagnosis |  | 6036 | 93726|54159 |  |
| SERPINA1 | serpin family A member 1 | SERPINA1 | 2.6 | 1.56E-160 | 8 | Extracellular Space | other | diagnosis,unspecified application |  | 5265 | 20704|20703|20702|20701|20700 | 24648 |
| HLA-DQB1 | major histocompatibility complex, class II, DQ beta 1 | HLA-DQB1 | 2.526 | 6.06E-94 | 2 | Plasma Membrane | other |  |  | 3119 | 14961 | 309622 |
| HAMP | hepcidin antimicrobial peptide | HAMP | 2.516 | 3.8E-47 | 19 | Extracellular Space | other | efficacy | NOX-H94 | 57817 |  |  |
| THEMIS2 | thymocyte selection associated family member 2 | THEMIS2 | 2.516 | 2.44E-103 | 1 | Other | other |  |  | 9473 | 230787 | 500561 |
| HCLS1 | hematopoietic cell-specific Lyn substrate 1 | HCLS1 | 2.494 | 2.42E-239 | 11 | Nucleus | other |  |  | 3059 | 15163 | 288077 |
| COTL1 | coactosin like F-actin binding protein 1 | COTL1 | 2.487 | 4.71E-72 | 1 | Cytoplasm | other |  |  | 23406 | 72042 | 361422 |
| MS4A6A | membrane spanning 4-domains A6A | MS4A6A | 2.47 | 8E-135 | 15 | Other | other |  |  | 64231 | 68774 | 361735 |
| TCIRG1 | T cell immune regulator 1, ATPase H+ transporting V0 subunit a3 | TCIRG1 | 2.454 | 4.94E-71 | 4 | Plasma Membrane | enzyme |  |  | 10312 | 27060 | 293650 |
| G0S2 | G0/G1 switch 2 | G0S2 | 2.411 | 7.28E-52 | 16 | Cytoplasm | other |  |  | 50486 | 14373 | 289388 |
| FCGR2A | Fc fragment of IgG receptor IIa | FCGR2A | 2.406 | 6.26E-194 | 11 | Plasma Membrane | transmembrane receptor |  | IgG | 2212 | 14131 | 116591|103693683|103694908|100911825|498276 |
| MYL12A | myosin light chain 12A | MYL12A | 2.395 | 8.88E-48 | 6 | Cytoplasm | other | unspecified application |  | 10627 | 67938 | 50685 |
| ANXA2 | annexin A2 | ANXA2 | 2.365 | 4.29E-44 | 8 | Plasma Membrane | other | diagnosis,unspecified application |  | 302 | 12306 | 56611 |
| TREM1 | triggering receptor expressed on myeloid cells 1 | TREM1 | 2.308 | 2.92E-50 | 4 | Plasma Membrane | transmembrane receptor | efficacy |  | 54210 | 58217 | 301229 |
| LAIR1 | leukocyte associated immunoglobulin like receptor 1 | LAIR1 | 2.28 | 7.27E-170 | 5 | Plasma Membrane | transmembrane receptor |  |  | 3903 | 52855 | 574531 |
| SH3BGRL3 | SH3 domain binding glutamate rich protein like 3 | SH3BGRL3 | 2.28 | 4.79E-53 | 18 | Nucleus | other |  |  | 83442 | 73723 | 298544 |
| GSTO1 | glutathione S-transferase omega 1 | GSTO1 | 2.238 | 1.78E-32 | 7 | Cytoplasm | enzyme | unspecified application |  | 9446 | 14873 | 114846 |
| BCL2A1 | BCL2 related protein A1 | BCL2A1 | 2.231 | 1.61E-71 | 15 | Cytoplasm | other |  |  | 597 | 12044|12045|12047 | 170929 |
| RGCC | regulator of cell cycle | RGCC | 2.22 | 2.14E-22 | 5 | Cytoplasm | other |  |  | 28984 | 66214 | 117183 |
| CTSH | cathepsin H | CTSH | 2.152 | 1.29E-72 | 10 | Cytoplasm | peptidase |  |  | 1512 | 13036 | 25425 |
| MPP1 | membrane palmitoylated protein 1 | MPP1 | 2.151 | 7.93E-56 | 10 | Plasma Membrane | kinase |  |  | 4354 | 17524 |  |
| CTSD | cathepsin D | CTSD | 2.131 | 1.26E-23 | 10 | Cytoplasm | peptidase | diagnosis,unspecified application |  | 1509 | 13033 | 171293 |
| CTSL | cathepsin L | CTSL1 | 2.123 | 4.34E-30 | 2 | Cytoplasm | peptidase |  | cathepsin L inhibitor | 1514 |  |  |
| ENG | endoglin | ENG | 2.121 | 5.31E-104 | 6 | Plasma Membrane | transmembrane receptor | disease progression,efficacy | TRC105 | 2022 | 13805 | 497010 |
| RNASE1 | ribonuclease A family member 1, pancreatic | RNASE1 | 2.111 | 2.66E-24 | 5 | Extracellular Space | enzyme |  |  | 6035 | 19752 | 364303|305844|364304 |
| CD81 | CD81 molecule | CD81 | 2.11 | 4.01E-29 | 12 | Plasma Membrane | other |  |  | 975 | 12520 | 25621 |
| METRNL | meteorin like, glial cell differentiation regulator | METRNL | 2.11 | 7.48E-89 | 7 | Cytoplasm | other |  |  | 284207 | 210029 | 316842 |
| CD4 | CD4 molecule | CD4 | 2.075 | 7.4E-187 | 12 | Plasma Membrane | transmembrane receptor | diagnosis,efficacy,unspecified application | zanolimumab, ibalizumab | 920 | 12504 | 24932 |
| ACSL1 | acyl-CoA synthetase long chain family member 1 | ACSL1 | 2.048 | 1.49E-43 | 14 | Cytoplasm | enzyme | unspecified application |  | 2180 | 14081 | 25288 |
| FCGBP | Fc fragment of IgG binding protein | FCGBP | 2.047 | 6.23E-67 | 4 | Extracellular Space | other |  |  | 8857 | 215384 | 100303643 |
| RAC2 | Rac family small GTPase 2 | RAC2 | 2.034 | 4.08E-62 | 12 | Cytoplasm | enzyme |  |  | 5880 | 19354 | 366957 |
| SLC16A3 | solute carrier family 16 member 3 | SLC16A3 | 2.017 | 3.11E-74 | 5 | Plasma Membrane | transporter |  |  | 9123 | 80879 | 80878 |
| CD300A | CD300a molecule | CD300A | 2.007 | 3.78E-108 | 15 | Plasma Membrane | transmembrane receptor |  |  | 11314 | 217303 | 501736 |
| SAMSN1 | SAM domain, SH3 domain and nuclear localization signals 1 | SAMSN1 | 2 | 6.68E-104 | 15 | Nucleus | other |  |  | 64092 | 67742 | 170637 |
| ALOX5 | arachidonate 5-lipoxygenase | ALOX5 | 1.928 | 1.64E-78 | 7 | Cytoplasm | enzyme | diagnosis,efficacy | TA 270, benoxaprofen, diclofenac/omeprazole, diclofenac/misoprostol, diclofenac, diethylcarbamazine, meclofenamic acid, zileuton, sulfasalazine, balsalazide, mesalamine, nordihydroguaiaretic acid, masoprocol | 240 | 11689 | 25290 |
| PLEK | pleckstrin | PLEK | 1.902 | 1.1E-97 | 8 | Cytoplasm | other |  |  | 5341 | 56193 | 364206 |
| RNF149 | ring finger protein 149 | RNF149 | 1.888 | 3.79E-77 | 4 | Cytoplasm | enzyme |  |  | 284996 | 67702 | 363222 |
| PFN1 | profilin 1 | PFN1 | 1.871 | 5.3E-50 | 6 | Cytoplasm | other | unspecified application |  | 5216 | 18643 | 64303 |
| LGMN | legumain | LGMN | 1.861 | 1.69E-25 | 19 | Cytoplasm | peptidase |  |  | 5641 | 19141 | 63865 |
| FABP5 | fatty acid binding protein 5 | FABP5 | 1.856 | 5.12E-23 | 17 | Cytoplasm | transporter | disease progression |  | 2171 | 16592 | 140868 |
| MSR1 | macrophage scavenger receptor 1 | MSR1 | 1.848 | 2.06E-96 | 1 | Plasma Membrane | transmembrane receptor |  |  | 4481 | 20288 | 498638 |
| ST14 | suppression of tumorigenicity 14 | ST14 | 1.839 | 4.85E-154 | 8 | Plasma Membrane | peptidase |  |  | 6768 | 19143 | 114093 |
| EFHD2 | EF-hand domain family member D2 | EFHD2 | 1.823 | 3.89E-62 | 15 | Other | other |  |  | 79180 | 27984 | 298609 |
| MAFB | MAF bZIP transcription factor B | MAFB | 1.799 | 2.91E-68 | 4 | Nucleus | transcription regulator |  |  | 9935 | 16658 | 54264 |
| S100A10 | S100 calcium binding protein A10 | S100A10 | 1.779 | 4.35E-35 | 1 | Cytoplasm | other |  |  | 6281 | 20194 | 81778 |
| HLA-DQA1 | major histocompatibility complex, class II, DQ alpha 1 | HLA-DQA1 | 1.773 | 4.31E-90 | 2 | Plasma Membrane | transmembrane receptor |  |  | 3117 | 14960 | 309621 |
| HLA-DPB1 | major histocompatibility complex, class II, DP beta 1 | HLA-DPB1 | 1.75 | 2.4E-110 | 2 | Plasma Membrane | transmembrane receptor |  |  | 3115 |  |  |
| HMOX1 | heme oxygenase 1 | HMOX1 | 1.749 | 7.66E-29 | 19 | Cytoplasm | enzyme | efficacy,safety,unspecified application | tin mesoporphyrin | 3162 | 15368 | 24451 |
| CMTM7 | CKLF like MARVEL transmembrane domain containing 7 | CMTM7 | 1.727 | 1.28E-68 | 13 | Extracellular Space | cytokine |  |  | 112616 | 102545 | 501065 |
| DUSP23 | dual specificity phosphatase 23 | DUSP23 | 1.706 | 7.96E-39 | 15 | Cytoplasm | phosphatase |  |  | 54935 | 68440 | 360881 |
| BST2 | bone marrow stromal cell antigen 2 | BST2 | 1.694 | 2.23E-57 | 4 | Plasma Membrane | other |  |  | 684 |  |  |
| SLA | Src like adaptor | SLA | 1.674 | 6.41E-95 | 19 | Plasma Membrane | other |  |  | 6503 | 20491 | 338477 |
| ATP5F1E | ATP synthase F1 subunit epsilon | ATP5E | 1.664 | 2.9E-43 | 12 | Cytoplasm | other |  |  | 514 |  |  |
| HBEGF | heparin binding EGF like growth factor | HBEGF | 1.652 | 4.27E-31 | 16 | Extracellular Space | growth factor | diagnosis,unspecified application |  | 1839 | 15200 | 25433 |
| CTSC | cathepsin C | CTSC | 1.648 | 4.4E-55 | 10 | Cytoplasm | peptidase | unspecified application |  | 1075 | 13032 | 25423 |
| OSTF1 | osteoclast stimulating factor 1 | OSTF1 | 1.641 | 2.04E-31 | 6 | Nucleus | transcription regulator |  |  | 26578 | 20409 | 259275 |
| RNASE6 | ribonuclease A family member k6 | RNASE6 | 1.639 | 8.94E-74 | 1 | Extracellular Space | enzyme |  |  | 6039 | 78416 | 305842 |
| CPVL | carboxypeptidase, vitellogenic like | CPVL | 1.626 | 3E-61 | 18 | Cytoplasm | peptidase |  |  | 54504 | 71287 | 502774 |
| NOP10 | NOP10 ribonucleoprotein | NOP10 | 1.608 | 3.05E-21 | 18 | Nucleus | other |  |  | 55505 | 66181 | 691534 |
| CD86 | CD86 molecule | CD86 | 1.589 | 2.77E-109 | 4 | Plasma Membrane | transmembrane receptor | efficacy,prognosis | abatacept, belatacept, abatacept/methotrexate | 942 | 12524 | 56822 |
| FERMT3 | fermitin family member 3 | FERMT3 | 1.576 | 2.97E-79 | 11 | Cytoplasm | enzyme |  |  | 83706 | 108101 | 309186 |
| TNFRSF1B | TNF receptor superfamily member 1B | TNFRSF1B | 1.559 | 4.2E-94 | 4 | Plasma Membrane | transmembrane receptor | efficacy,safety |  | 7133 | 21938 | 156767 |
| IL1RN | interleukin 1 receptor antagonist | IL1RN | 1.547 | 8.11E-56 | 7 | Extracellular Space | cytokine | efficacy |  | 3557 | 16181 | 60582 |
| ARHGAP9 | Rho GTPase activating protein 9 | ARHGAP9 | 1.545 | 3.98E-67 | 16 | Cytoplasm | other |  |  | 64333 | 216445 | 362893 |
| GNAI2 | G protein subunit alpha i2 | GNAI2 | 1.536 | 1.9E-65 | 12 | Plasma Membrane | enzyme |  |  | 2771 | 14678 | 81664 |
| PRR13 | proline rich 13 | PRR13 | 1.53 | 2.74E-35 | 20 | Nucleus | other |  |  | 54458 |  |  |
| TLR2 | toll like receptor 2 | TLR2 | 1.503 | 1.87E-101 | 4 | Plasma Membrane | transmembrane receptor | diagnosis,efficacy,unspecified application | OM 174 lipid | 7097 | 24088 | 310553 |
| SLC31A2 | solute carrier family 31 member 2 | SLC31A2 | 1.48 | 1.31E-25 | 20 | Plasma Membrane | transporter |  |  | 1318 | 20530 | 298091 |
| STXBP2 | syntaxin binding protein 2 | STXBP2 | 1.462 | 1.15E-62 | 17 | Plasma Membrane | transporter |  |  | 6813 | 20911 | 81804 |
| SERPINB1 | serpin family B member 1 | SERPINB1 | 1.46 | 1.79E-55 | 5 | Cytoplasm | other |  |  | 1992 | 66222 | 291091 |
| TBXAS1 | thromboxane A synthase 1 | TBXAS1 | 1.451 | 3.64E-117 | 3 | Plasma Membrane | enzyme |  | ridogrel | 6916 | 21391 | 24886 |
| PARVG | parvin gamma | PARVG | 1.444 | 6.54E-74 | 6 | Cytoplasm | other |  |  | 64098 | 64099 | 689069 |
| NCF4 | neutrophil cytosolic factor 4 | NCF4 | 1.442 | 2.95E-69 | 1 | Cytoplasm | enzyme |  |  | 4689 | 17972 | 500904 |
| MFSD1 | major facilitator superfamily domain containing 1 | MFSD1 | 1.427 | 1.32E-32 | 3 | Other | transporter |  |  | 64747 | 66868 | 361957 |
| FBP1 | fructose-bisphosphatase 1 | FBP1 | 1.424 | 2.54E-46 | 4 | Cytoplasm | phosphatase |  |  | 2203 | 14121 | 24362 |
| LYN | LYN proto-oncogene, Src family tyrosine kinase | LYN | 1.41 | 5.65E-58 | 15 | Cytoplasm | kinase |  | bafetinib, nintedanib, JNJ-26483327, rebastinib, docetaxel/nintedanib, bosutinib, tolimidone | 4067 | 17096 | 81515 |
| IL18 | interleukin 18 | IL18 | 1.409 | 2.95E-47 | 16 | Extracellular Space | cytokine | efficacy,unspecified application |  | 3606 | 16173 | 29197 |
| TNFSF13 | TNF superfamily member 13 | TNFSF13 | 1.409 | 8.56E-35 | 9 | Extracellular Space | cytokine |  |  | 8741 | 69583 | 287437 |
| IFITM2 | interferon induced transmembrane protein 2 | IFITM2 | 1.381 | 1.37E-43 | 12 | Cytoplasm | other | prognosis |  | 10581 | 80876 | 114709 |
| TK1 | thymidine kinase 1 | TK1 | 1.369 | 3.24E-26 | 17 | Cytoplasm | kinase | diagnosis,prognosis |  | 7083 | 21877 | 24834 |
| CKLF | chemokine like factor | CKLF | 1.352 | 1.66E-22 | 12 | Extracellular Space | cytokine |  |  | 51192 | 75458 | 245978 |
| RAB20 | RAB20, member RAS oncogene family | RAB20 | 1.352 | 1.21E-42 | 1 | Cytoplasm | enzyme |  |  | 55647 | 19332 | 689377 |
| TMC6 | transmembrane channel like 6 | TMC6 | 1.351 | 3.25E-38 | 3 | Cytoplasm | transporter |  |  | 11322 | 217353 |  |
| CRIP1 | cysteine rich protein 1 | CRIP1 | 1.345 | 4.19E-36 | 12 | Cytoplasm | other | unspecified application |  | 1396 | 12925 | 691657 |
| VMO1 | vitelline membrane outer layer 1 homolog | VMO1 | 1.327 | 9.66E-22 |  | Extracellular Space | other |  |  | 284013 | 327956 | 360553 |
| COX14 | COX14, cytochrome c oxidase assembly factor | COX14 | 1.318 | 2.04E-22 | 13 | Cytoplasm | other |  |  | 84987 | 66379 | 681219 |
| MGST2 | microsomal glutathione S-transferase 2 | MGST2 | 1.315 | 1.83E-33 | 7 | Cytoplasm | enzyme |  |  | 4258 | 211666 | 295037 |
| CTSS | cathepsin S | CTSS | 1.313 | 1.53E-106 | 10 | Cytoplasm | peptidase |  |  | 1520 | 13040 | 50654 |
| ATP5ME | ATP synthase membrane subunit e | ATP5I | 1.309 | 1.29E-35 | 18 | Cytoplasm | transporter |  |  | 521 |  |  |
| SCIN | scinderin | SCIN | 1.303 | 4.61E-66 | 6 | Cytoplasm | other |  |  | 85477 | 20259 | 298975 |
| SH2B3 | SH2B adaptor protein 3 | SH2B3 | 1.294 | 1.2E-55 | 2 | Plasma Membrane | other |  |  | 10019 | 16923 |  |
| ARHGAP4 | Rho GTPase activating protein 4 | ARHGAP4 | 1.29 | 3.7E-61 | 2 | Cytoplasm | other |  |  | 393 | 171207 | 246249 |
| GAS6 | growth arrest specific 6 | GAS6 | 1.268 | 1.17E-28 | 1 | Extracellular Space | growth factor |  |  | 2621 | 14456 | 58935 |
| CALHM6 | calcium homeostasis modulator family member 6 | FAM26F | 1.266 | 4.7E-51 | 3 | Other | other |  |  | 441168 | 215900 | 294430 |
| LTBR | lymphotoxin beta receptor | LTBR | 1.261 | 1.61E-29 | 16 | Plasma Membrane | transmembrane receptor |  |  | 4055 | 17000 | 297604 |
| VIM | vimentin | VIM | 1.256 | 3.06E-25 | 14 | Cytoplasm | other | diagnosis,efficacy,prognosis,unspecified application |  | 7431 | 22352 | 81818 |
| NAPSB | napsin B aspartic peptidase, pseudogene | NAPSB | 1.25 | 2.97E-40 |  | Other | other |  |  | 256236 |  |  |
| SLC25A19 | solute carrier family 25 member 19 | SLC25A19 | 1.246 | 3.46E-22 | 3 | Cytoplasm | transporter |  |  | 60386 | 67283 | 303676 |
| CD63 | CD63 molecule | CD63 | 1.241 | 7.32E-72 | 2 | Plasma Membrane | other |  |  | 967 | 12512 | 29186 |
| DOK3 | docking protein 3 | DOK3 | 1.241 | 1.95E-50 | 9 | Cytoplasm | other |  |  | 79930 | 27261 | 306760 |
| ANXA1 | annexin A1 | ANXA1 | 1.24 | 1.38E-34 | 8 | Plasma Membrane | enzyme | diagnosis,prognosis,unspecified application | hydrocortisone, hydrocortisone/prednisone, hydrocortisone/mitoxantrone | 301 | 16952 | 25380 |
| RGS19 | regulator of G protein signaling 19 | RGS19 | 1.236 | 2.51E-30 | 10 | Cytoplasm | other |  |  | 10287 | 56470 | 59293 |
| IL1B | interleukin 1 beta | IL1B | 1.233 | 2.24E-27 | 5 | Extracellular Space | cytokine | diagnosis,efficacy,prognosis | canakinumab, gevokizumab, canakinumab/INS, gallium nitrate | 3553 | 16176 | 24494 |
| AVPI1 | arginine vasopressin induced 1 | AVPI1 | 1.232 | 2.8E-23 | 3 | Other | other |  |  | 60370 | 69534 | 171386 |
| SASH3 | SAM and SH3 domain containing 3 | SASH3 | 1.229 | 1.74E-50 | 4 | Cytoplasm | other |  |  | 54440 | 74131 | 317578 |
| SLC15A3 | solute carrier family 15 member 3 | SLC15A3 | 1.214 | 2.89E-42 | 7 | Cytoplasm | transporter |  |  | 51296 | 65221 | 246239 |
| SAP30 | Sin3A associated protein 30 | SAP30 | 1.204 | 3.83E-38 | 14 | Nucleus | transcription regulator | unspecified application |  | 8819 | 60406 | 680122 |
| LY96 | lymphocyte antigen 96 | LY96 | 1.201 | 1.5E-28 | 7 | Plasma Membrane | transmembrane receptor |  |  | 23643 | 17087 | 448830 |
| TPM3 | tropomyosin 3 | TPM3 | 1.183 | 4.66E-36 | 6 | Cytoplasm | other | diagnosis,unspecified application | AZD-7451 | 7170 | 59069 |  |
| CALM1 (includes others) | calmodulin 1 | CALM3 | 1.179 | 3.49E-97 | 17 | Cytoplasm | other |  |  | 801|805|808 |  |  |
| SPINT2 | serine peptidase inhibitor, Kunitz type 2 | SPINT2 | 1.177 | 3.75E-21 | 18 | Extracellular Space | other | diagnosis |  | 10653 | 20733 | 292770 |
| MYL6 | myosin light chain 6 | MYL6 | 1.176 | 1.13E-31 | 6 | Cytoplasm | enzyme |  |  | 4637 | 17904 | 685867 |
| ANXA11 | annexin A11 | ANXA11 | 1.175 | 1.75E-52 | 1 | Nucleus | other |  |  | 311 | 11744 | 290527 |
| HCK | HCK proto-oncogene, Src family tyrosine kinase | HCK | 1.167 | 1.09E-52 | 16 | Cytoplasm | kinase |  | rebastinib, bosutinib | 3055 | 15162 | 25734 |
| SPI1 | Spi-1 proto-oncogene | SPI1 | 1.164 | 7.92E-85 | 14 | Nucleus | transcription regulator |  |  | 6688 | 20375 | 366126 |
| IBSP | integrin binding sialoprotein | IBSP | 1.162 | 4.98E-30 | 5 | Extracellular Space | other | efficacy |  | 3381 | 15891 | 24477 |
| HLA-DRB5 | major histocompatibility complex, class II, DR beta 5 | HLA-DRB5 | 1.157 | 9.03E-67 | 2 | Plasma Membrane | transmembrane receptor |  |  | 3127 | 14969 | 294270 |
| SIGLEC10 | sialic acid binding Ig like lectin 10 | SIGLEC10 | 1.156 | 7.8E-60 | 15 | Plasma Membrane | other |  |  | 89790 | 243958 | 292844 |
| C1orf54 | chromosome 1 open reading frame 54 | C1orf54 | 1.155 | 1.21E-28 | 13 | Other | other |  |  | 79630 | 229600 |  |
| NCF1 | neutrophil cytosolic factor 1 | NCF1 | 1.117 | 3.15E-26 | 1 | Cytoplasm | enzyme | efficacy |  | 653361 | 17969 | 114553 |
| LSP1 | lymphocyte-specific protein 1 | LSP1 | 1.107 | 1.38E-27 | 6 | Cytoplasm | other |  |  | 4046 | 16985 | 361680 |
| CLEC5A | C-type lectin domain containing 5A | CLEC5A | 1.104 | 1.91E-62 | 2 | Plasma Membrane | other |  |  | 23601 | 23845 | 679787 |
| COX6B1 | cytochrome c oxidase subunit 6B1 | COX6B1 | 1.097 | 1.8E-40 | 8 | Cytoplasm | enzyme |  |  | 1340 | 110323 | 688869 |
| ZNF331 | zinc finger protein 331 | ZNF331 | 1.08 | 5.05E-33 | 16 | Nucleus | other |  |  | 55422 |  |  |
| AKAP13 | A-kinase anchoring protein 13 | AKAP13 | 1.078 | 3.81E-28 | 8 | Cytoplasm | other |  |  | 11214 | 75547 | 293024 |
| LITAF | lipopolysaccharide induced TNF factor | LITAF | 1.065 | 5.31E-35 | 4 | Nucleus | transcription regulator |  |  | 9516 | 56722 | 65161 |
| MYO1F | myosin IF | MYO1F | 1.065 | 6.03E-58 | 6 | Cytoplasm | other |  |  | 4542 | 17916 | 314654 |
| S100A6 | S100 calcium binding protein A6 | S100A6 | 1.065 | 4.97E-29 | 1 | Cytoplasm | transporter | diagnosis,unspecified application |  | 6277 | 20200 | 85247 |
| GRB2 | growth factor receptor bound protein 2 | GRB2 | 1.049 | 3.39E-36 | 16 | Cytoplasm | kinase |  | liposome-incorporated Grb2 antisense oligodeoxynucleotide | 2885 | 14784 | 81504 |
| TIMP1 | TIMP metallopeptidase inhibitor 1 | TIMP1 | 1.039 | 3.41E-55 | 8 | Extracellular Space | cytokine | diagnosis,disease progression,efficacy,prognosis,unspecified application |  | 7076 | 21857 | 116510 |
| MNDA | myeloid cell nuclear differentiation antigen | MNDA | 1.023 | 2.19E-35 | 14 | Nucleus | other |  |  | 4332 |  | C |

Cluster 5 microglia

| © 2000-2018 QIAGEN. All rights reserved. |  |  |  |  |  |  |  |  |  |  |  |  |
| --- | --- | --- | --- | --- | --- | --- | --- | --- | --- | --- | --- | --- |
| Symbol | Entrez Gene Name | Gene Symbol - human (HUGO / HGNC / Entrez Gene)/Gene Symbol - mouse (Entrez Gene)/Gene Symbol - rat (Entrez Gene) | Expr Log Ratio | Expr False Discovery Rate (q-value) | Networks | Location | Type(s) | Biomarker Application(s) | Drug(s) | Entrez Gene ID for Human | Entrez Gene ID for Mouse | Entrez Gene ID for Rat |
| CSF1R | colony stimulating factor 1 receptor | CSF1R | 4.879 | 0 | 14 | Plasma Membrane | kinase | prognosis | nilotinib, sunitinib, pazopanib, quizartinib, pexidartinib, IMC-CS4, PLX7486, ARRY-382, JNJ-40346527, BLZ-945, FPA008, AMG 820, DCC-3014, emactuzumab, bosutinib, imatinib, SNDX-6352, imatinib/sunitinib, imatinib/nilotinib, dasatinib/nilotinib, crizotinib/pazopanib, decitabine/imatinib | 1436 | 12978 | 307403 |
| A2M | alpha-2-macroglobulin | A2M | 4.601 | 1.5E-175 | 3 | Extracellular Space | transporter |  |  | 2 | 232345 | 100911545|24153 |
| CH25H | cholesterol 25-hydroxylase | CH25H | 4.196 | 3.04E-161 | 15 | Cytoplasm | enzyme | unspecified application |  | 9023 | 12642 | 309527 |
| GPR34 | G protein-coupled receptor 34 | GPR34 | 4.065 | 4.81E-246 | 17 | Plasma Membrane | G-protein coupled receptor |  |  | 2857 | 23890 | 554353 |
| AIF1 | allograft inflammatory factor 1 | AIF1 | 3.94 | 6.66E-286 | 12,21 | Nucleus | other |  |  | 199 | 11629 | 29427 |
| CCL4 | C-C motif chemokine ligand 4 | CCL4 | 3.591 | 1.62E-82 | 10 | Extracellular Space | cytokine | diagnosis,efficacy,prognosis,unspecified application |  | 6351 | 20303 | 116637 |
| PLXDC2 | plexin domain containing 2 | PLXDC2 | 3.471 | 5.9E-172 | 9 | Extracellular Space | other |  |  | 84898 | 67448 | 361282 |
| CD37 | CD37 molecule | CD37 | 3.45 | 3.2E-191 | 22 | Plasma Membrane | other |  | otlertuzumab, BI 836826, IMGN529 | 951 | 12493 | 29185 |
| SLC2A5 | solute carrier family 2 member 5 | SLC2A5 | 3.368 | 4.81E-246 | 23 | Plasma Membrane | transporter |  |  | 6518 | 56485 | 65197 |
| P2RY12 | purinergic receptor P2Y12 | P2RY12 | 3.252 | 2.76E-145 |  | Plasma Membrane | G-protein coupled receptor |  | treprostinil, epoprostenol, cangrelor, prasugrel, ticagrelor, clopidogrel/telmisartan, (2Z)-2-[1-[(S)-(2-chlorophenyl)-methoxycarbonyl-methyl]-4-sulfanyl-3-piperidylidene]acetic acid, aspirin/clopidogrel, ticlopidine, clopidogrel | 64805 | 70839 | 64803 |
| ADORA3 | adenosine A3 receptor | ADORA3 | 3.128 | 4.07E-196 |  | Plasma Membrane | G-protein coupled receptor | efficacy | CF102, adenosine, dyphylline, aminothiadiazole, clofarabine, theophylline, caffeine | 140 | 11542 |  |
| ADAM28 | ADAM metallopeptidase domain 28 | ADAM28 | 3.088 | 3.17E-197 | 25 | Plasma Membrane | peptidase |  |  | 10863 | 13522 | 290344 |
| IFNGR1 | interferon gamma receptor 1 | IFNGR1 | 3.059 | 1.69E-40 |  | Plasma Membrane | transmembrane receptor |  | interferon gamma-1b | 3459 | 15979 | 116465 |
| ST6GAL1 | ST6 beta-galactoside alpha-2,6-sialyltransferase 1 | ST6GAL1 | 3.048 | 9.3E-65 |  | Cytoplasm | enzyme |  |  | 6480 | 20440 | 25197 |
| SELPLG | selectin P ligand | SELPLG | 2.801 | 2.04E-150 | 24 | Plasma Membrane | other |  |  | 6404 | 20345 | 363930 |
| ITGAX | integrin subunit alpha X | ITGAX | 2.781 | 8.32E-210 | 24 | Plasma Membrane | transmembrane receptor | unspecified application |  | 3687 | 16411 | 499271 |
| PDK4 | pyruvate dehydrogenase kinase 4 | PDK4 | 2.607 | 1.1E-71 |  | Cytoplasm | kinase |  |  | 5166 | 27273 | 89813 |
| BIN1 | bridging integrator 1 | BIN1 | 2.585 | 1.53E-66 | 20 | Nucleus | other |  |  | 274 | 30948 | 117028 |
| SIGLEC8 | sialic acid binding Ig like lectin 8 | SIGLEC8 | 2.576 | 2.33E-159 | 15 | Plasma Membrane | transmembrane receptor |  |  | 27181 | 233186 | 292843 |
| CX3CR1 | C-X3-C motif chemokine receptor 1 | CX3CR1 | 2.534 | 1.36E-87 | 25 | Plasma Membrane | G-protein coupled receptor | unspecified application |  | 1524 | 13051 | 171056 |
| TMEM119 | transmembrane protein 119 | TMEM119 | 2.45 | 2.07E-112 | 16 | Cytoplasm | other |  |  | 338773 | 231633 | 304581 |
| SH3TC1 | SH3 domain and tetratricopeptide repeats 1 | SH3TC1 | 2.427 | 2.34E-170 | 3 | Extracellular Space | other |  |  | 54436 | 231147 | 305441 |
| FGD2 | FYVE, RhoGEF and PH domain containing 2 | FGD2 | 2.327 | 7.08E-210 | 15 | Cytoplasm | other |  |  | 221472 | 26382 | 309653 |
| TSC22D3 | TSC22 domain family member 3 | TSC22D3 | 2.289 | 4.11E-38 | 3 | Nucleus | transcription regulator |  |  | 1831 | 14605 |  |
| FCGR1B | Fc fragment of IgG receptor Ib | FCGR1B | 2.25 | 2.3E-134 | 7 | Plasma Membrane | transmembrane receptor |  | IgG | 2210 |  |  |
| SORL1 | sortilin related receptor 1 | SORL1 | 2.241 | 1.28E-71 | 19 | Cytoplasm | transporter |  |  | 6653 | 20660 | 300652 |
| ITGAM | integrin subunit alpha M | ITGAM | 2.134 | 1.44E-156 | 3 | Plasma Membrane | transmembrane receptor | efficacy,unspecified application |  | 3684 | 16409 | 25021 |
| C6orf62 | chromosome 6 open reading frame 62 | C6orf62 | 2.123 | 2.79E-69 | 18 | Other | other |  |  | 81688 | 79555 |  |
| SRGAP2 | SLIT-ROBO Rho GTPase activating protein 2 | SRGAP2 | 2.015 | 1.19E-97 | 18 | Cytoplasm | other |  |  | 23380 | 14270 | 360840 |
| LOC284454 | uncharacterized LOC284454 | LOC284454 | 2.002 | 9.3E-65 |  | Other | other |  |  | 284454 |  |  |
| DOCK8 | dedicator of cytokinesis 8 | DOCK8 | 1.969 | 5.7E-154 | 2 | Cytoplasm | other |  |  | 81704 | 76088 | 499337 |
| DAGLB | diacylglycerol lipase beta | DAGLB | 1.951 | 8.69E-28 |  | Plasma Membrane | enzyme |  |  | 221955 | 231871 | 304289 |
| MEF2C | myocyte enhancer factor 2C | MEF2C | 1.942 | 3.17E-124 | 11 | Nucleus | transcription regulator | unspecified application |  | 4208 | 17260 | 499497 |
| SLC1A3 | solute carrier family 1 member 3 | SLC1A3 | 1.933 | 1.37E-48 |  | Plasma Membrane | transporter |  | riluzole | 6507 | 20512 | 29483 |
| ITM2B | integral membrane protein 2B | ITM2B | 1.906 | 3.64E-21 | 9 | Plasma Membrane | other |  |  | 9445 | 16432 | 290364 |
| EGR3 | early growth response 3 | EGR3 | 1.902 | 1.1E-69 | 3 | Nucleus | transcription regulator |  |  | 1960 | 13655 | 25148 |
| BHLHE41 | basic helix-loop-helix family member e41 | BHLHE41 | 1.847 | 2E-118 | 20 | Nucleus | transcription regulator |  |  | 79365 |  |  |
| OLFML3 | olfactomedin like 3 | OLFML3 | 1.808 | 8E-50 |  | Extracellular Space | other |  |  | 56944 | 99543 | 310743 |
| ADRB2 | adrenoceptor beta 2 | ADRB2 | 1.72 | 5.59E-48 |  | Plasma Membrane | G-protein coupled receptor | diagnosis,efficacy,unspecified application | articaine/epinephrine, bupivacaine/epinephrine, carteolol, dipivefrin, meluadrine, epinephrine/prilocaine, epinephrine/lidocaine, bedoradrine, KUL 7211, celiprolol, arformoterol, indacaterol, myogane, budesonide/formoterol, nebivolol, vilanterol, olodaterol, formoterol/mometasone furoate, glycopyrrolate/indacaterol, fluticasone furoate/vilanterol, latanoprost/timolol, umeclidinium/vilanterol, epinephrine/methotrexate, olodaterol/tiotropium, indacaterol/tiotropium, nebivolol/valsartan, formoterol/glycopyrrolate, glycopyrrolate/indacaterol/mometasone furoate, indacaterol/mometasone furoate, fluticasone/salmeterol, albuterol/ipratropium, carvedilol, ephedrine, guanethidine, levalbuterol, propranolol, dexamethasone/olanzapine, pindolol, esmolol, metoprolol, alprenolol, salmeterol, dorzolamide/timolol, fluoxetine/olanzapine, guanadrel, bendroflumethiazide/nadolol, isoxsuprine, hydrochlorothiazide/propranolol, hydrochlorothiazide/timolol, fluticasone furoate/umeclidinium/vilanterol, isoproterenol, sotalol, bambuterol, nadolol, timolol, isoetharine, ritodrine, olanzapine, venlafaxine, labetalol, formoterol, bitolterol, albuterol, terbutaline, procaterol, pirbuterol, clenbuterol, fenoterol, norepinephrine, metaproterenol sulfate, epinephrine, dobutamine, droxidopa, arbutamine | 154 | 11555 | 24176 |
| ACY3 | aminoacylase 3 | ACY3 | 1.71 | 4.07E-76 |  | Cytoplasm | enzyme |  |  | 91703 | 71670 | 293653 |
| PDGFB | platelet derived growth factor subunit B | PDGFB | 1.695 | 2E-102 |  | Extracellular Space | growth factor | disease progression |  | 5155 | 18591 | 24628 |
| GPR84 | G protein-coupled receptor 84 | GPR84 | 1.67 | 3.31E-46 |  | Plasma Membrane | G-protein coupled receptor |  |  | 53831 | 80910 | 688730 |
| CD84 | CD84 molecule | CD84 | 1.624 | 1.93E-113 | 23 | Plasma Membrane | other |  |  | 8832 | 12523 | 501872 |
| P2RY13 | purinergic receptor P2Y13 | P2RY13 | 1.611 | 3.53E-87 |  | Plasma Membrane | G-protein coupled receptor |  |  | 53829 | 74191 | 310444 |
| FMNL3 | formin like 3 | FMNL3 | 1.59 | 2.9E-77 | 18 | Cytoplasm | other |  |  | 91010 | 22379 | 300225 |
| IL6ST | interleukin 6 signal transducer | IL6ST | 1.416 | 1.6E-27 | 14 | Plasma Membrane | transmembrane receptor | unspecified application |  | 3572 | 16195 | 25205 |
| SHISA9 | shisa family member 9 | SHISA9 | 1.316 | 1.82E-113 |  | Plasma Membrane | other |  |  | 729993 | 72555 | 100361134 |
| TMEM212 | transmembrane protein 212 | TMEM212 | 1.307 | 1.62E-102 |  | Other | other |  |  | 389177 | 208613 | 499586 |
| SFMBT2 | Scm like with four mbt domains 2 | SFMBT2 | 1.276 | 1.37E-98 | 10 | Nucleus | other |  |  | 57713 | 353282 | 307106 |
| C2 | complement C2 | C2 | 1.241 | 1.55E-50 | 25 | Extracellular Space | peptidase |  |  | 717 | 12263 | 24231 |
| ENTPD1 | ectonucleoside triphosphate diphosphohydrolase 1 | ENTPD1 | 1.23 | 7.02E-37 | 25 | Plasma Membrane | enzyme |  |  | 953 | 12495 | 64519 |
| TSC22D2 | TSC22 domain family member 2 | TSC22D2 | 1.225 | 5.61E-33 | 3 | Extracellular Space | other |  |  | 9819 | 72033 | 499624 |
| SUSD3 | sushi domain containing 3 | SUSD3 | 1.204 | 1.01E-24 | 4 | Plasma Membrane | other |  |  | 203328 | 66329 | 306810 |
| ANKRD44 | ankyrin repeat domain 44 | ANKRD44 | 1.199 | 7.06E-35 | 16 | Other | other |  |  | 91526 | 329154 | 301415 |
| CCL5 | C-C motif chemokine ligand 5 | CCL5 | 1.19 | 1.17E-77 | 4 | Extracellular Space | cytokine | diagnosis,efficacy,unspecified application |  | 6352 | 20304 | 81780 |
| ORC4 | origin recognition complex subunit 4 | ORC4 | 1.185 | 5.56E-42 | 5 | Nucleus | other |  |  | 5000 | 26428 | 295596 |
| DHRS9 | dehydrogenase/reductase 9 | DHRS9 | 1.136 | 1.04E-40 | 11 | Cytoplasm | enzyme |  |  | 10170 | 241452 | 170635 |
| ASTN2 | astrotactin 2 | ASTN2 | 1.083 | 1.33E-75 |  | Cytoplasm | other |  |  | 23245 | 56079 | 100361323 |
| SHTN1 | shootin 1 | KIAA1598 | 1.064 | 4.35E-41 | 18 | Plasma Membrane | other |  |  | 57698 | 71653 | 292139 |
| TNF | tumor necrosis factor | TNF | 1.062 | 2.75E-27 | 6 | Extracellular Space | cytokine | diagnosis,disease progression,efficacy,prognosis,response to therapy,safety,unspecified application | adalimumab, etanercept, infliximab, certolizumab, golimumab, tumor necrosis factor receptor antagonist, infliximab/methotrexate, dexamethasone/thalidomide, dexamethasone/pomalidomide, cyclophosphamide/dexamethasone/thalidomide, golimumab/methotrexate, bortezomib/dexamethasone/thalidomide, rituximab/thalidomide, bortezomib/thalidomide, prednisone/thalidomide, adalimumab/methotrexate, etanercept/methotrexate, pomalidomide, bortezomib/dexamethasone/pomalidomide, thalidomide | 7124 | 21926 | 24835 |
| DDX5 | DEAD-box helicase 5 | DDX5 | 1.048 | 1.13E-46 | 5 | Nucleus | enzyme | unspecified application |  | 1655 | 13207 | 287765 |

Cluster 5 macrophage

| © 2000-2018 QIAGEN. All rights reserved. |  |  |  |  |  |  |  |  |  |  |  |  |
| --- | --- | --- | --- | --- | --- | --- | --- | --- | --- | --- | --- | --- |
| Symbol | Entrez Gene Name | Gene Symbol - human (HUGO / HGNC / Entrez Gene)/Gene Symbol - mouse (Entrez Gene)/Gene Symbol - rat (Entrez Gene) | Expr Log Ratio | Expr False Discovery Rate (q-value) | Networks | Location | Type(s) | Biomarker Application(s) | Drug(s) | Entrez Gene ID for Human | Entrez Gene ID for Mouse | Entrez Gene ID for Rat |
| CD74 | CD74 molecule | CD74 | 6.364 | 0 | 22 | Plasma Membrane | transmembrane receptor |  | milatuzumab | 972 | 16149 | 25599 |
| RGS1 | regulator of G protein signaling 1 | RGS1 | 6.34 | 0 |  | Plasma Membrane | other |  |  | 5996 | 50778 | 54289 |
| HLA-DPA1 | major histocompatibility complex, class II, DP alpha 1 | HLA-DPA1 | 6.21 | 0 | 22 | Plasma Membrane | transmembrane receptor |  |  | 3113 |  |  |
| HLA-DRA | major histocompatibility complex, class II, DR alpha | HLA-DRA | 6.041 | 8.89E-242 | 22 | Plasma Membrane | transmembrane receptor |  |  | 3122 | 100504404 | 294269 |
| FCER1G | Fc fragment of IgE receptor Ig | FCER1G | 4.802 | 0 | 13 | Plasma Membrane | transmembrane receptor |  |  | 2207 | 14127 | 25441 |
| HLA-DMB | major histocompatibility complex, class II, DM beta | HLA-DMB | 4.316 | 0 | 6 | Plasma Membrane | transmembrane receptor |  |  | 3109 | 15000|14999 |  |
| FCGR3A/FCGR3B | Fc fragment of IgG receptor IIIa | FCGR3A | 4.29 | 7.73E-278 | 13 | Plasma Membrane | transmembrane receptor |  | IgG, AFM13 | 2214|2215 | 246256 | 304966 |
| HLA-DRB1 | major histocompatibility complex, class II, DR beta 1 | HLA-DRB1 | 4.149 | 1.13E-202 | 6 | Plasma Membrane | transmembrane receptor |  | apolizumab | 3123 |  |  |
| IFI30 | IFI30, lysosomal thiol reductase | IFI30 | 3.914 | 2.2E-245 |  | Cytoplasm | enzyme |  |  | 10437 | 65972 | 290644 |
| CXCR4 | C-X-C motif chemokine receptor 4 | CXCR4 | 3.601 | 1.25E-108 | 25 | Plasma Membrane | G-protein coupled receptor | diagnosis | AMD 11070, ulocuplumab, cladribine/cytarabine/filgrastim/idarubicin/plerixafor, POL6326, BL-8040, LY-2510924, burixafor, USL311, PF-06747143, plerixafor, filgrastim/plerixafor | 7852 | 12767 | 60628 |
| CD14 | CD14 molecule | CD14 | 3.593 | 3.09E-232 | 10 | Plasma Membrane | transmembrane receptor | efficacy,unspecified application |  | 929 | 12475 | 60350 |
| HLA-DRB5 | major histocompatibility complex, class II, DR beta 5 | HLA-DRB5 | 3.512 | 1.23E-194 | 6,21 | Plasma Membrane | transmembrane receptor |  |  | 3127 | 14969 | 294270 |
| SERPINB9 | serpin family B member 9 | SERPINB9 | 3.484 | 2.57E-286 | 9 | Cytoplasm | other |  |  | 5272 | 20723 | 361241 |
| CYBB | cytochrome b-245 beta chain | CYBB | 3.476 | 4.47E-202 | 7 | Cytoplasm | enzyme |  |  | 1536 | 13058 | 66021 |
| CXCL16 | C-X-C motif chemokine ligand 16 | CXCL16 | 3.435 | 2.47E-149 | 10 | Extracellular Space | cytokine | diagnosis,disease progression,unspecified application |  | 58191 | 66102 | 497942 |
| HLA-DQB1 | major histocompatibility complex, class II, DQ beta 1 | HLA-DQB1 | 3.348 | 3.6E-208 | 22 | Plasma Membrane | other |  |  | 3119 | 14961 | 309622 |
| HLA-DQA1 | major histocompatibility complex, class II, DQ alpha 1 | HLA-DQA1 | 3.343 | 1.22E-199 | 22 | Plasma Membrane | transmembrane receptor |  |  | 3117 | 14960 | 309621 |
| ACSL1 | acyl-CoA synthetase long chain family member 1 | ACSL1 | 3.279 | 2.43E-128 |  | Cytoplasm | enzyme | unspecified application |  | 2180 | 14081 | 25288 |
| NPC2 | NPC intracellular cholesterol transporter 2 | NPC2 | 3.275 | 1.16E-86 | 24 | Extracellular Space | transporter |  |  | 10577 | 67963 | 286898 |
| HLA-DPB1 | major histocompatibility complex, class II, DP beta 1 | HLA-DPB1 | 3.265 | 1.07E-256 | 22 | Plasma Membrane | transmembrane receptor |  |  | 3115 |  |  |
| MS4A6A | membrane spanning 4-domains A6A | MS4A6A | 3.235 | 5.66E-188 | 2 | Other | other |  |  | 64231 | 68774 | 361735 |
| MS4A7 | membrane spanning 4-domains A7 | MS4A7 | 3.098 | 3.44E-194 | 21 | Other | other |  |  | 58475 | 109225 | 293744 |
| PLEK | pleckstrin | PLEK | 3.087 | 2.61E-175 | 12 | Cytoplasm | other |  |  | 5341 | 56193 | 364206 |
| PLAUR | plasminogen activator, urokinase receptor | PLAUR | 2.982 | 8.97E-112 | 14 | Plasma Membrane | transmembrane receptor | diagnosis,disease progression |  | 5329 | 18793 | 50692 |
| HLA-B | major histocompatibility complex, class I, B | HLA-B | 2.869 | 3.07E-25 | 22 | Plasma Membrane | transmembrane receptor | safety |  | 3106 |  |  |
| GSN | gelsolin | GSN | 2.786 | 7.73E-44 |  | Extracellular Space | other | disease progression,efficacy |  | 2934 | 227753 | 296654 |
| RAB20 | RAB20, member RAS oncogene family | RAB20 | 2.781 | 1.36E-117 | 20 | Cytoplasm | enzyme |  |  | 55647 | 19332 | 689377 |
| TPT1 | tumor protein, translationally-controlled 1 | TPT1 | 2.652 | 3.93E-71 | 1 | Cytoplasm | other |  |  | 7178 | 22070 | 116646 |
| BCL2A1 | BCL2 related protein A1 | BCL2A1 | 2.649 | 3.97E-121 | 24 | Cytoplasm | other |  |  | 597 | 12044|12045|12047 | 170929 |
| SYNGR2 | synaptogyrin 2 | SYNGR2 | 2.618 | 1.86E-86 | 14 | Cytoplasm | other |  |  | 9144 | 20973 | 89815 |
| NR4A2 | nuclear receptor subfamily 4 group A member 2 | NR4A2 | 2.61 | 1.46E-101 | 11 | Nucleus | ligand-dependent nuclear receptor |  |  | 4929 | 18227 | 54278 |
| STOM | stomatin | STOM | 2.606 | 1.37E-50 | 5 | Plasma Membrane | other |  |  | 2040 | 13830 | 296655 |
| PLTP | phospholipid transfer protein | PLTP | 2.551 | 7.7E-46 | 20 | Extracellular Space | enzyme |  |  | 5360 | 18830 | 296371 |
| AOAH | acyloxyacyl hydrolase | AOAH | 2.429 | 4.6E-123 | 18 | Extracellular Space | enzyme |  |  | 313 | 27052 | 498757 |
| TMEM176B | transmembrane protein 176B | TMEM176B | 2.301 | 1.62E-37 | 6 | Other | other |  |  | 28959 | 65963 | 171411 |
| MS4A4A | membrane spanning 4-domains A4A | MS4A4A | 2.255 | 3.98E-142 | 12 | Cytoplasm | other |  |  | 51338 | 666907 |  |
| SERPINA1 | serpin family A member 1 | SERPINA1 | 2.239 | 5.46E-109 | 24 | Extracellular Space | other | diagnosis,unspecified application |  | 5265 | 20704|20703|20702|20701|20700 | 24648 |
| HLA-C | major histocompatibility complex, class I, C | HLA-C | 2.23 | 1.13E-131 | 22 | Plasma Membrane | other | response to therapy |  | 3107 |  |  |
| CCR1 | C-C motif chemokine receptor 1 | CCR1 | 2.2 | 2.07E-138 |  | Plasma Membrane | G-protein coupled receptor |  |  | 1230 | 12768 | 57301 |
| MTHFD2 | methylenetetrahydrofolate dehydrogenase (NADP+ dependent) 2, methenyltetrahydrofolate cyclohydrolase | MTHFD2 | 2.164 | 1.57E-33 | 3 | Cytoplasm | enzyme |  |  | 10797 | 17768 | 680308 |
| DUSP2 | dual specificity phosphatase 2 | DUSP2 | 2.157 | 1.42E-69 | 4 | Nucleus | phosphatase |  |  | 1844 | 13537 | 311406 |
| IFITM2 | interferon induced transmembrane protein 2 | IFITM2 | 2.091 | 4.61E-68 | 5 | Cytoplasm | other | prognosis |  | 10581 | 80876 | 114709 |
| C1orf162 | chromosome 1 open reading frame 162 | C1orf162 | 2.071 | 4.78E-94 |  | Other | transporter |  |  | 128346 | 433638 | 100911379|100363228 |
| RPS4X | ribosomal protein S4, X-linked | RPS4X | 2.055 | 5.15E-38 | 8 | Cytoplasm | other | diagnosis |  | 6191 |  |  |
| TNFSF13 | TNF superfamily member 13 | TNFSF13 | 2.032 | 8.32E-56 | 10 | Extracellular Space | cytokine |  |  | 8741 | 69583 | 287437 |
| RPLP1 | ribosomal protein lateral stalk subunit P1 | RPLP1 | 2.009 | 7.69E-54 | 1 | Cytoplasm | other |  |  | 6176 |  | 140661 |
| RIN3 | Ras and Rab interactor 3 | RIN3 | 1.939 | 2.65E-128 | 8 | Cytoplasm | other |  |  | 79890 | 217835 | 314397 |
| EVI2B | ecotropic viral integration site 2B | EVI2B | 1.931 | 1.52E-63 |  | Plasma Membrane | other |  |  | 2124 | 101488212|216984 | 100910940 |
| HLA-DQA2 | major histocompatibility complex, class II, DQ alpha 2 | HLA-DQA2 | 1.855 | 5.95E-184 | 6 | Plasma Membrane | transmembrane receptor |  |  | 3118 |  |  |
| VAMP8 | vesicle associated membrane protein 8 | VAMP8 | 1.83 | 2.58E-74 | 24 | Plasma Membrane | transporter |  |  | 8673 | 22320 | 83730 |
| FXYD5 | FXYD domain containing ion transport regulator 5 | FXYD5 | 1.799 | 4E-56 | 14 | Plasma Membrane | ion channel |  |  | 53827 | 18301 | 60338 |
| ARHGAP15 | Rho GTPase activating protein 15 | ARHGAP15 | 1.783 | 6.56E-88 | 19 | Cytoplasm | other |  |  | 55843 | 76117 | 295635 |
| TYMP | thymidine phosphorylase | TYMP | 1.697 | 2.51E-112 | 4 | Extracellular Space | growth factor | diagnosis,efficacy,prognosis,response to therapy |  | 1890 | 72962 | 315219 |
| GPR84 | G protein-coupled receptor 84 | GPR84 | 1.67 | 3.31E-46 |  | Plasma Membrane | G-protein coupled receptor |  |  | 53831 | 80910 | 688730 |
| SLC7A5 | solute carrier family 7 member 5 | SLC7A5 | 1.617 | 5.01E-21 | 9 | Plasma Membrane | transporter | diagnosis |  | 8140 | 20539 | 50719 |
| PLAC8 | placenta specific 8 | PLAC8 | 1.609 | 2.17E-78 | 12 | Nucleus | other |  |  | 51316 | 231507 | 360914 |
| THBD | thrombomodulin | THBD | 1.534 | 5E-52 | 10 | Plasma Membrane | transmembrane receptor | efficacy |  | 7056 | 21824 | 83580 |
| SPINT2 | serine peptidase inhibitor, Kunitz type 2 | SPINT2 | 1.519 | 2.87E-42 | 3 | Extracellular Space | other | diagnosis |  | 10653 | 20733 | 292770 |
| RPL9 | ribosomal protein L9 | RPL9 | 1.51 | 1.32E-32 | 1 | Nucleus | other | diagnosis |  | 6133 | 20005 | 100360449|29257|100364457 |
| PRDM1 | PR/SET domain 1 | PRDM1 | 1.499 | 4.12E-57 | 5 | Nucleus | transcription regulator |  |  | 639 | 12142 | 309871 |
| PRKCH | protein kinase C eta | PRKCH | 1.473 | 1.19E-73 |  | Cytoplasm | kinase | diagnosis | ingenol mebutate | 5583 | 18755 | 81749 |
| FGR | FGR proto-oncogene, Src family tyrosine kinase | FGR | 1.472 | 4.41E-60 | 12 | Nucleus | kinase |  | vemurafenib, rebastinib, cobimetinib/vemurafenib, dabrafenib/trametinib/vemurafenib, cetuximab/vemurafenib, trametinib/vemurafenib | 2268 | 14191 | 79113 |
| CD163 | CD163 molecule | CD163 | 1.457 | 1.77E-50 | 20 | Plasma Membrane | transmembrane receptor |  |  | 9332 | 93671 | 312701 |
| GYPC | glycophorin C (Gerbich blood group) | GYPC | 1.449 | 6.28E-58 | 8 | Plasma Membrane | other | unspecified application |  | 2995 | 71683 | 364837 |
| EMB | embigin | EMB | 1.442 | 4.76E-80 | 11 | Plasma Membrane | transporter |  |  | 133418 | 13723 | 114511 |
| RPL10 | ribosomal protein L10 | RPL10 | 1.407 | 4.1E-46 | 1 | Cytoplasm | translation regulator |  |  | 6134 | 110954 | 81764 |
| RPS3A | ribosomal protein S3A | RPS3A | 1.397 | 6.59E-34 | 1 | Nucleus | other |  |  | 6189 |  | 100365839 |
| LYZ | lysozyme | LYZ | 1.348 | 4.92E-32 | 25 | Extracellular Space | enzyme | unspecified application |  | 4069 | 17110|17105 | 25211 |
| SAMHD1 | SAM and HD domain containing deoxynucleoside triphosphate triphosphohydrolase 1 | SAMHD1 | 1.339 | 1.7E-30 | 17 | Nucleus | enzyme |  |  | 25939 | 56045 | 311580 |
| ANXA11 | annexin A11 | ANXA11 | 1.322 | 6.96E-54 | 19 | Nucleus | other |  |  | 311 | 11744 | 290527 |
| SYTL3 | synaptotagmin like 3 | SYTL3 | 1.319 | 2.55E-42 | 13 | Cytoplasm | other |  |  | 94120 | 83672 | 499017 |
| DOK3 | docking protein 3 | DOK3 | 1.306 | 8.59E-29 | 23 | Cytoplasm | other |  |  | 79930 | 27261 | 306760 |
| RPS27A | ribosomal protein S27a | RPS27A | 1.28 | 6.13E-64 | 8 | Cytoplasm | other |  |  | 6233 | 78294 | 100912032 |
| SIK1/SIK1B | salt inducible kinase 1 | SIK1 | 1.28 | 3.81E-46 | 11 | Nucleus | kinase |  | dabrafenib, dabrafenib/trametinib, dabrafenib/panitumumab, dabrafenib/trametinib/vemurafenib, dabrafenib/panitumumab/trametinib | 150094|102724428 |  |  |
| RPL23 | ribosomal protein L23 | RPL23 | 1.278 | 9.56E-45 | 1 | Cytoplasm | other |  |  | 9349 | 65019 | 29282 |
| SELL | selectin L | SELL | 1.275 | 8.49E-59 | 14 | Plasma Membrane | transmembrane receptor | efficacy |  | 6402 | 20343 | 29259 |
| RPSA | ribosomal protein SA | RPSA | 1.262 | 5.56E-49 | 1 | Cytoplasm | translation regulator |  |  | 3921 | 16785 | 29236 |
| FCGBP | Fc fragment of IgG binding protein | FCGBP | 1.237 | 5.04E-47 | 13 | Extracellular Space | other |  |  | 8857 | 215384 | 100303643 |
| PLBD1 | phospholipase B domain containing 1 | PLBD1 | 1.182 | 7.86E-34 |  | Extracellular Space | enzyme |  |  | 79887 | 66857 | 297694 |
| EEF1G | eukaryotic translation elongation factor 1 gamma | EEF1G | 1.176 | 9.04E-30 | 1 | Cytoplasm | translation regulator |  |  | 1937 | 67160 | 293725 |
| SOD2 | superoxide dismutase 2 | SOD2 | 1.175 | 1.91E-33 | 3 | Cytoplasm | enzyme | diagnosis,unspecified application |  | 6648 | 20656 | 24787 |
| F13A1 | coagulation factor XIII A chain | F13A1 | 1.169 | 3.18E-35 |  | Extracellular Space | enzyme |  |  | 2162 | 74145 | 60327 |
| ADGRE2 | adhesion G protein-coupled receptor E2 | EMR2 | 1.153 | 4.36E-59 |  | Plasma Membrane | other |  |  | 30817 |  |  |
| PTPN1 | protein tyrosine phosphatase, non-receptor type 1 | PTPN1 | 1.144 | 6.99E-29 | 4 | Cytoplasm | phosphatase | prognosis | protein tyrosine phosphatase 1B inhibitor, trodusquemine | 5770 | 19246 | 24697 |
| CD300C | CD300c molecule | CD300C | 1.141 | 2.66E-44 | 6 | Plasma Membrane | transmembrane receptor |  |  | 10871 | 387565|140497 | 287813|501742|498022|100909671|303666|501745 |
| RPS23 | ribosomal protein S23 | RPS23 | 1.14 | 5.74E-34 | 8 | Cytoplasm | translation regulator |  |  | 6228 | 66475 | 124323 |
| ST8SIA4 | ST8 alpha-N-acetyl-neuraminide alpha-2,8-sialyltransferase 4 | ST8SIA4 | 1.121 | 8.64E-25 |  | Cytoplasm | enzyme |  |  | 7903 | 20452 | 116696 |
| ATP8B4 | ATPase phospholipid transporting 8B4 (putative) | ATP8B4 | 1.094 | 7.38E-97 | 18 | Plasma Membrane | transporter |  |  | 79895 | 241633 | 311396 |
| CALHM6 | calcium homeostasis modulator family member 6 | FAM26F | 1.093 | 3.87E-25 | 17 | Other | other |  |  | 441168 | 215900 | 294430 |
| NUP98 | nucleoporin 98 | NUP98 | 1.086 | 9.83E-25 | 21 | Nucleus | transporter |  |  | 4928 | 269966 | 81738 |
| EEF1A1 | eukaryotic translation elongation factor 1 alpha 1 | EEF1A1 | 1.082 | 3.77E-50 | 1 | Cytoplasm | translation regulator |  |  | 1915 | 13627 | 171361 |
| TREM1 | triggering receptor expressed on myeloid cells 1 | TREM1 | 1.082 | 8.15E-38 | 10 | Plasma Membrane | transmembrane receptor | efficacy |  | 54210 | 58217 | 301229 |
| MAPKAPK3 | mitogen-activated protein kinase-activated protein kinase 3 | MAPKAPK3 | 1.068 | 2.92E-29 | 11 | Nucleus | kinase | unspecified application |  | 7867 | 102626 | 315994 |
| RPL5 | ribosomal protein L5 | RPL5 | 1.043 | 9.79E-41 | 1 | Cytoplasm | other |  |  | 6125 | 100503670 | 81763 |
| RPS3 | ribosomal protein S3 | RPS3 | 1.017 | 1.85E-46 | 1 | Cytoplasm | enzyme | unspecified application |  | 6188 | 27050 | 140654 |

Cluster 5 pro-inflammatory

| © 2000-2018 QIAGEN. All rights reserved. |  |  |  |  |  |  |  |  |  |  |  |  |
| --- | --- | --- | --- | --- | --- | --- | --- | --- | --- | --- | --- | --- |
| Symbol | Entrez Gene Name | Gene Symbol - human (HUGO / HGNC / Entrez Gene)/Gene Symbol - mouse (Entrez Gene)/Gene Symbol - rat (Entrez Gene) | Expr Log Ratio | Expr False Discovery Rate (q-value) | Networks | Location | Type(s) | Biomarker Application(s) | Drug(s) | Entrez Gene ID for Human | Entrez Gene ID for Mouse | Entrez Gene ID for Rat |
| RGS1 | regulator of G protein signaling 1 | RGS1 | 6.34 | 0 |  | Plasma Membrane | other |  |  | 5996 | 50778 | 54289 |
| CSF1R | colony stimulating factor 1 receptor | CSF1R | 4.879 | 0 | 14 | Plasma Membrane | kinase | prognosis | nilotinib, sunitinib, pazopanib, quizartinib, pexidartinib, IMC-CS4, PLX7486, ARRY-382, JNJ-40346527, BLZ-945, FPA008, AMG 820, DCC-3014, emactuzumab, bosutinib, imatinib, SNDX-6352, imatinib/sunitinib, imatinib/nilotinib, dasatinib/nilotinib, crizotinib/pazopanib, decitabine/imatinib | 1436 | 12978 | 307403 |
| A2M | alpha-2-macroglobulin | A2M | 4.601 | 1.5E-175 | 3 | Extracellular Space | transporter |  |  | 2 | 232345 | 100911545|24153 |
| CH25H | cholesterol 25-hydroxylase | CH25H | 4.196 | 3.04E-161 | 15 | Cytoplasm | enzyme | unspecified application |  | 9023 | 12642 | 309527 |
| CCL3 | C-C motif chemokine ligand 3 | CCL3 | 4.107 | 1.88E-127 | 24 | Extracellular Space | cytokine | diagnosis,efficacy,prognosis |  | 6348 |  |  |
| GPR34 | G protein-coupled receptor 34 | GPR34 | 4.065 | 4.81E-246 | 17 | Plasma Membrane | G-protein coupled receptor |  |  | 2857 | 23890 | 554353 |
| IL1B | interleukin 1 beta | IL1B | 4.06 | 1.92E-135 | 9 | Extracellular Space | cytokine | diagnosis,efficacy,prognosis | canakinumab, gevokizumab, canakinumab/INS, gallium nitrate | 3553 | 16176 | 24494 |
| PLD4 | phospholipase D family member 4 | PLD4 | 3.653 | 8.33E-202 |  | Extracellular Space | enzyme |  |  | 122618 | 104759 | 362792 |
| CCL4 | C-C motif chemokine ligand 4 | CCL4 | 3.591 | 1.62E-82 | 10 | Extracellular Space | cytokine | diagnosis,efficacy,prognosis,unspecified application |  | 6351 | 20303 | 116637 |
| CD68 | CD68 molecule | CD68 | 3.422 | 2.27E-205 |  | Plasma Membrane | other | diagnosis,efficacy |  | 968 | 12514 | 287435 |
| CD83 | CD83 molecule | CD83 | 3.399 | 7.98E-118 | 10 | Plasma Membrane | transmembrane receptor |  |  | 9308 | 12522 | 361226 |
| RGS10 | regulator of G protein signaling 10 | RGS10 | 3.285 | 6.3E-218 | 11 | Cytoplasm | other |  |  | 6001 | 67865 | 54290 |
| TREM2 | triggering receptor expressed on myeloid cells 2 | TREM2 | 3.282 | 1.84E-223 | 7 | Plasma Membrane | transmembrane receptor |  |  | 54209 | 83433 |  |
| P2RY12 | purinergic receptor P2Y12 | P2RY12 | 3.252 | 2.76E-145 |  | Plasma Membrane | G-protein coupled receptor |  | treprostinil, epoprostenol, cangrelor, prasugrel, ticagrelor, clopidogrel/telmisartan, (2Z)-2-[1-[(S)-(2-chlorophenyl)-methoxycarbonyl-methyl]-4-sulfanyl-3-piperidylidene]acetic acid, aspirin/clopidogrel, ticlopidine, clopidogrel | 64805 | 70839 | 64803 |
| ADORA3 | adenosine A3 receptor | ADORA3 | 3.128 | 4.07E-196 |  | Plasma Membrane | G-protein coupled receptor | efficacy | CF102, adenosine, dyphylline, aminothiadiazole, clofarabine, theophylline, caffeine | 140 | 11542 |  |
| ADAM28 | ADAM metallopeptidase domain 28 | ADAM28 | 3.088 | 3.17E-197 | 25 | Plasma Membrane | peptidase |  |  | 10863 | 13522 | 290344 |
| DUSP1 | dual specificity phosphatase 1 | DUSP1 | 3.063 | 1.27E-43 | 4 | Nucleus | phosphatase | diagnosis,efficacy |  | 1843 | 19252 | 114856 |
| IFNGR1 | interferon gamma receptor 1 | IFNGR1 | 3.059 | 1.69E-40 |  | Plasma Membrane | transmembrane receptor |  | interferon gamma-1b | 3459 | 15979 | 116465 |
| NLRP3 | NLR family pyrin domain containing 3 | NLRP3 | 2.982 | 3.3E-165 | 9 | Cytoplasm | other |  |  | 114548 | 216799 | 287362 |
| NR4A1 | nuclear receptor subfamily 4 group A member 1 | NR4A1 | 2.963 | 2.97E-52 | 11 | Nucleus | ligand-dependent nuclear receptor | unspecified application |  | 3164 | 15370 | 79240 |
| B4GALT1 | beta-1,4-galactosyltransferase 1 | B4GALT1 | 2.902 | 1.83E-195 |  | Cytoplasm | enzyme | diagnosis |  | 2683 | 14595 | 24390 |
| HLA-B | major histocompatibility complex, class I, B | HLA-B | 2.869 | 3.07E-25 | 22 | Plasma Membrane | transmembrane receptor | safety |  | 3106 |  |  |
| SELPLG | selectin P ligand | SELPLG | 2.801 | 2.04E-150 | 24 | Plasma Membrane | other |  |  | 6404 | 20345 | 363930 |
| IRF8 | interferon regulatory factor 8 | IRF8 | 2.781 | 5.79E-142 | 7 | Nucleus | transcription regulator |  |  | 3394 | 15900 | 292060 |
| ITGAX | integrin subunit alpha X | ITGAX | 2.781 | 8.32E-210 | 24 | Plasma Membrane | transmembrane receptor | unspecified application |  | 3687 | 16411 | 499271 |
| CCL3L3 | C-C motif chemokine ligand 3 like 3 | CCL3L3 | 2.752 | 4.67E-54 | 14 | Extracellular Space | cytokine | unspecified application |  | 414062 | 20302 | 25542 |
| SGK1 | serum/glucocorticoid regulated kinase 1 | SGK1 | 2.719 | 1.69E-22 | 5 | Cytoplasm | kinase |  |  | 6446 | 20393 | 29517 |
| PDK4 | pyruvate dehydrogenase kinase 4 | PDK4 | 2.607 | 1.1E-71 |  | Cytoplasm | kinase |  |  | 5166 | 27273 | 89813 |
| BIN1 | bridging integrator 1 | BIN1 | 2.585 | 1.53E-66 | 20 | Nucleus | other |  |  | 274 | 30948 | 117028 |
| SIGLEC8 | sialic acid binding Ig like lectin 8 | SIGLEC8 | 2.576 | 2.33E-159 | 15 | Plasma Membrane | transmembrane receptor |  |  | 27181 | 233186 | 292843 |
| CX3CR1 | C-X3-C motif chemokine receptor 1 | CX3CR1 | 2.534 | 1.36E-87 | 25 | Plasma Membrane | G-protein coupled receptor | unspecified application |  | 1524 | 13051 | 171056 |
| FGD2 | FYVE, RhoGEF and PH domain containing 2 | FGD2 | 2.327 | 7.08E-210 | 15 | Cytoplasm | other |  |  | 221472 | 26382 | 309653 |
| TSC22D3 | TSC22 domain family member 3 | TSC22D3 | 2.289 | 4.11E-38 | 3 | Nucleus | transcription regulator |  |  | 1831 | 14605 |  |
| FCGR1B | Fc fragment of IgG receptor Ib | FCGR1B | 2.25 | 2.3E-134 | 7 | Plasma Membrane | transmembrane receptor |  | IgG | 2210 |  |  |
| SORL1 | sortilin related receptor 1 | SORL1 | 2.241 | 1.28E-71 | 19 | Cytoplasm | transporter |  |  | 6653 | 20660 | 300652 |
| BTG2 | BTG anti-proliferation factor 2 | BTG2 | 2.236 | 1.34E-29 |  | Nucleus | transcription regulator |  |  | 7832 | 12227 | 29619 |
| HLA-C | major histocompatibility complex, class I, C | HLA-C | 2.23 | 1.13E-131 | 22 | Plasma Membrane | other | response to therapy |  | 3107 |  |  |
| CCR1 | C-C motif chemokine receptor 1 | CCR1 | 2.2 | 2.07E-138 |  | Plasma Membrane | G-protein coupled receptor |  |  | 1230 | 12768 | 57301 |
| PADI2 | peptidyl arginine deiminase 2 | PADI2 | 2.176 | 3.99E-47 | 23 | Cytoplasm | enzyme |  |  | 11240 | 18600 | 29511 |
| MEF2A | myocyte enhancer factor 2A | MEF2A | 2.104 | 4.24E-126 | 11 | Nucleus | transcription regulator |  |  | 4205 | 17258 | 309957 |
| SEMA4D | semaphorin 4D | SEMA4D | 2.1 | 4.75E-115 |  | Plasma Membrane | transmembrane receptor |  | VX15/2503 | 10507 | 20354 | 306790 |
| SPTLC2 | serine palmitoyltransferase long chain base subunit 2 | SPTLC2 | 2.06 | 2.21E-95 | 3 | Cytoplasm | enzyme |  |  | 9517 | 20773 | 366697 |
| SRGAP2 | SLIT-ROBO Rho GTPase activating protein 2 | SRGAP2 | 2.015 | 1.19E-97 | 18 | Cytoplasm | other |  |  | 23380 | 14270 | 360840 |
| LOC284454 | uncharacterized LOC284454 | LOC284454 | 2.002 | 9.3E-65 |  | Other | other |  |  | 284454 |  |  |
| DOCK8 | dedicator of cytokinesis 8 | DOCK8 | 1.969 | 5.7E-154 | 2 | Cytoplasm | other |  |  | 81704 | 76088 | 499337 |
| B3GNT5 | UDP-GlcNAc:betaGal beta-1,3-N-acetylglucosaminyltransferase 5 | B3GNT5 | 1.961 | 1.13E-84 | 23 | Cytoplasm | enzyme |  |  | 84002 | 108105 | 116740 |
| DAGLB | diacylglycerol lipase beta | DAGLB | 1.951 | 8.69E-28 |  | Plasma Membrane | enzyme |  |  | 221955 | 231871 | 304289 |
| MEF2C | myocyte enhancer factor 2C | MEF2C | 1.942 | 3.17E-124 | 11 | Nucleus | transcription regulator | unspecified application |  | 4208 | 17260 | 499497 |
| SLC1A3 | solute carrier family 1 member 3 | SLC1A3 | 1.933 | 1.37E-48 |  | Plasma Membrane | transporter |  | riluzole | 6507 | 20512 | 29483 |
| ITM2B | integral membrane protein 2B | ITM2B | 1.906 | 3.64E-21 | 9 | Plasma Membrane | other |  |  | 9445 | 16432 | 290364 |
| EGR3 | early growth response 3 | EGR3 | 1.902 | 1.1E-69 | 3 | Nucleus | transcription regulator |  |  | 1960 | 13655 | 25148 |
| BHLHE41 | basic helix-loop-helix family member e41 | BHLHE41 | 1.847 | 2E-118 | 20 | Nucleus | transcription regulator |  |  | 79365 |  |  |
| APBB1IP | amyloid beta precursor protein binding family B member 1 interacting protein | APBB1IP | 1.844 | 4.57E-121 | 12 | Cytoplasm | other |  |  | 54518 | 54519 | 307171 |
| LPAR5 | lysophosphatidic acid receptor 5 | LPAR5 | 1.818 | 5.27E-88 |  | Plasma Membrane | G-protein coupled receptor |  |  | 57121 | 381810 | 500317 |
| SKIL | SKI like proto-oncogene | SKIL | 1.771 | 1.24E-71 | 16 | Nucleus | transcription regulator |  |  | 6498 | 20482 | 114208 |
| MKNK1 | MAP kinase interacting serine/threonine kinase 1 | MKNK1 | 1.743 | 1.29E-39 | 11 | Cytoplasm | kinase |  | BAY1143269, eFT508 | 8569 | 17346 | 500526 |
| ADRB2 | adrenoceptor beta 2 | ADRB2 | 1.72 | 5.59E-48 |  | Plasma Membrane | G-protein coupled receptor | diagnosis,efficacy,unspecified application | articaine/epinephrine, bupivacaine/epinephrine, carteolol, dipivefrin, meluadrine, epinephrine/prilocaine, epinephrine/lidocaine, bedoradrine, KUL 7211, celiprolol, arformoterol, indacaterol, myogane, budesonide/formoterol, nebivolol, vilanterol, olodaterol, formoterol/mometasone furoate, glycopyrrolate/indacaterol, fluticasone furoate/vilanterol, latanoprost/timolol, umeclidinium/vilanterol, epinephrine/methotrexate, olodaterol/tiotropium, indacaterol/tiotropium, nebivolol/valsartan, formoterol/glycopyrrolate, glycopyrrolate/indacaterol/mometasone furoate, indacaterol/mometasone furoate, fluticasone/salmeterol, albuterol/ipratropium, carvedilol, ephedrine, guanethidine, levalbuterol, propranolol, dexamethasone/olanzapine, pindolol, esmolol, metoprolol, alprenolol, salmeterol, dorzolamide/timolol, fluoxetine/olanzapine, guanadrel, bendroflumethiazide/nadolol, isoxsuprine, hydrochlorothiazide/propranolol, hydrochlorothiazide/timolol, fluticasone furoate/umeclidinium/vilanterol, isoproterenol, sotalol, bambuterol, nadolol, timolol, isoetharine, ritodrine, olanzapine, venlafaxine, labetalol, formoterol, bitolterol, albuterol, terbutaline, procaterol, pirbuterol, clenbuterol, fenoterol, norepinephrine, metaproterenol sulfate, epinephrine, dobutamine, droxidopa, arbutamine | 154 | 11555 | 24176 |
| ACY3 | aminoacylase 3 | ACY3 | 1.71 | 4.07E-76 |  | Cytoplasm | enzyme |  |  | 91703 | 71670 | 293653 |
| WASF2 | WAS protein family member 2 | WASF2 | 1.677 | 2.33E-34 | 12 | Plasma Membrane | other |  |  | 10163 | 242687 |  |
| CD84 | CD84 molecule | CD84 | 1.624 | 1.93E-113 | 23 | Plasma Membrane | other |  |  | 8832 | 12523 | 501872 |
| P2RY13 | purinergic receptor P2Y13 | P2RY13 | 1.611 | 3.53E-87 |  | Plasma Membrane | G-protein coupled receptor |  |  | 53829 | 74191 | 310444 |
| FMNL3 | formin like 3 | FMNL3 | 1.59 | 2.9E-77 | 18 | Cytoplasm | other |  |  | 91010 | 22379 | 300225 |
| SMAP2 | small ArfGAP2 | SMAP2 | 1.557 | 5.26E-36 |  | Cytoplasm | other |  |  | 64744 | 69780 | 298500 |
| HK2 | hexokinase 2 | HK2 | 1.514 | 3.49E-56 | 20 | Cytoplasm | kinase |  |  | 3099 | 15277 | 25059 |
| FOSB | FosB proto-oncogene, AP-1 transcription factor subunit | FOSB | 1.491 | 8.08E-51 | 11 | Nucleus | transcription regulator |  |  | 2354 | 14282 | 100360880 |
| GYPC | glycophorin C (Gerbich blood group) | GYPC | 1.449 | 6.28E-58 | 8 | Plasma Membrane | other | unspecified application |  | 2995 | 71683 | 364837 |
| IL6ST | interleukin 6 signal transducer | IL6ST | 1.416 | 1.6E-27 | 14 | Plasma Membrane | transmembrane receptor | unspecified application |  | 3572 | 16195 | 25205 |
| PLVAP | plasmalemma vesicle associated protein | PLVAP | 1.372 | 2.98E-99 | 6 | Plasma Membrane | other |  |  | 83483 | 84094 | 56765 |
| IL1A | interleukin 1 alpha | IL1A | 1.35 | 9.83E-48 | 25 | Extracellular Space | cytokine | efficacy,prognosis,unspecified application | MABp1 | 3552 | 16175 | 24493 |
| SHISA9 | shisa family member 9 | SHISA9 | 1.316 | 1.82E-113 |  | Plasma Membrane | other |  |  | 729993 | 72555 | 100361134 |
| TMEM156 | transmembrane protein 156 | TMEM156 | 1.285 | 3.23E-72 |  | Other | other |  |  | 80008 | 243025 | 498365 |
| SFMBT2 | Scm like with four mbt domains 2 | SFMBT2 | 1.276 | 1.37E-98 | 10 | Nucleus | other |  |  | 57713 | 353282 | 307106 |
| ENTPD1 | ectonucleoside triphosphate diphosphohydrolase 1 | ENTPD1 | 1.23 | 7.02E-37 | 25 | Plasma Membrane | enzyme |  |  | 953 | 12495 | 64519 |
| TSC22D2 | TSC22 domain family member 2 | TSC22D2 | 1.225 | 5.61E-33 | 3 | Extracellular Space | other |  |  | 9819 | 72033 | 499624 |
| SLC25A37 | solute carrier family 25 member 37 | SLC25A37 | 1.209 | 3.54E-23 | 14,18 | Cytoplasm | transporter |  |  | 51312 | 67712 | 306000 |
| GAL3ST4 | galactose-3-O-sulfotransferase 4 | GAL3ST4 | 1.201 | 2.54E-25 | 9 | Cytoplasm | enzyme |  |  | 79690 | 330217 | 498166 |
| ANKRD44 | ankyrin repeat domain 44 | ANKRD44 | 1.199 | 7.06E-35 | 16 | Other | other |  |  | 91526 | 329154 | 301415 |
| CCL5 | C-C motif chemokine ligand 5 | CCL5 | 1.19 | 1.17E-77 | 4 | Extracellular Space | cytokine | diagnosis,efficacy,unspecified application |  | 6352 | 20304 | 81780 |
| ARRDC2 | arrestin domain containing 2 | ARRDC2 | 1.189 | 7.02E-22 | 16 | Other | other |  |  | 27106 | 70807 |  |
| ARHGAP25 | Rho GTPase activating protein 25 | ARHGAP25 | 1.185 | 7.24E-61 |  | Cytoplasm | other |  |  | 9938 | 232201 | 500246 |
| USP4 | ubiquitin specific peptidase 4 | USP4 | 1.183 | 1.34E-30 | 16 | Nucleus | peptidase |  |  | 7375 | 22258 | 290864 |
| DHRS9 | dehydrogenase/reductase 9 | DHRS9 | 1.136 | 1.04E-40 | 11 | Cytoplasm | enzyme |  |  | 10170 | 241452 | 170635 |
| SPHK1 | sphingosine kinase 1 | SPHK1 | 1.129 | 1.39E-21 | 5 | Cytoplasm | kinase | diagnosis,disease progression,prognosis,unspecified application |  | 8877 | 20698 | 170897 |
| CPED1 | cadherin like and PC-esterase domain containing 1 | CPED1 | 1.113 | 2.05E-79 |  | Cytoplasm | other |  |  | 79974 | 214642 | 500046 |
| BIN2 | bridging integrator 2 | BIN2 | 1.11 | 5.69E-52 | 8 | Plasma Membrane | other |  |  | 51411 | 668218 | 366988 |
| BLNK | B cell linker | BLNK | 1.108 | 2.87E-36 | 11 | Cytoplasm | other |  |  | 29760 | 17060 | 499356 |
| SHTN1 | shootin 1 | KIAA1598 | 1.064 | 4.35E-41 | 18 | Plasma Membrane | other |  |  | 57698 | 71653 | 292139 |
| TNF | tumor necrosis factor | TNF | 1.062 | 2.75E-27 | 6 | Extracellular Space | cytokine | diagnosis,disease progression,efficacy,prognosis,response to therapy,safety,unspecified application | adalimumab, etanercept, infliximab, certolizumab, golimumab, tumor necrosis factor receptor antagonist, infliximab/methotrexate, dexamethasone/thalidomide, dexamethasone/pomalidomide, cyclophosphamide/dexamethasone/thalidomide, golimumab/methotrexate, bortezomib/dexamethasone/thalidomide, rituximab/thalidomide, bortezomib/thalidomide, prednisone/thalidomide, adalimumab/methotrexate, etanercept/methotrexate, pomalidomide, bortezomib/dexamethasone/pomalidomide, thalidomide | 7124 | 21926 | 24835 |
| DDX5 | DEAD-box helicase 5 | DDX5 | 1.048 | 1.13E-46 | 5 | Nucleus | enzyme | unspecified application |  | 1655 | 13207 | 287765 |
| VASH1 | vasohibin 1 | VASH1 | 1.029 | 1.02E-38 | 6 | Extracellular Space | other |  |  | 22846 | 238328 | 503052 |

Cluster 5 anti-inflammatory

| © 2000-2018 QIAGEN. All rights reserved. |  |  |  |  |  |  |  |  |  |  |  |  |
| --- | --- | --- | --- | --- | --- | --- | --- | --- | --- | --- | --- | --- |
| Symbol | Entrez Gene Name | Gene Symbol - human (HUGO / HGNC / Entrez Gene)/Gene Symbol - mouse (Entrez Gene)/Gene Symbol - rat (Entrez Gene) | Expr Log Ratio | Expr False Discovery Rate (q-value) | Networks | Location | Type(s) | Biomarker Application(s) | Drug(s) | Entrez Gene ID for Human | Entrez Gene ID for Mouse | Entrez Gene ID for Rat |
| CD74 | CD74 molecule | CD74 | 6.364 | 0 | 22 | Plasma Membrane | transmembrane receptor |  | milatuzumab | 972 | 16149 | 25599 |
| HLA-DPA1 | major histocompatibility complex, class II, DP alpha 1 | HLA-DPA1 | 6.21 | 0 | 22 | Plasma Membrane | transmembrane receptor |  |  | 3113 |  |  |
| HLA-DRA | major histocompatibility complex, class II, DR alpha | HLA-DRA | 6.041 | 8.89E-242 | 22 | Plasma Membrane | transmembrane receptor |  |  | 3122 | 100504404 | 294269 |
| C1QB | complement C1q B chain | C1QB | 5.631 | 0 | 13 | Extracellular Space | peptidase |  |  | 713 | 12260 | 29687 |
| C1QC | complement C1q C chain | C1QC | 4.946 | 0 | 13 | Extracellular Space | peptidase |  |  | 714 | 12262 | 362634 |
| FCER1G | Fc fragment of IgE receptor Ig | FCER1G | 4.802 | 0 | 13 | Plasma Membrane | transmembrane receptor |  |  | 2207 | 14127 | 25441 |
| TYROBP | TYRO protein tyrosine kinase binding protein | TYROBP | 4.733 | 0 | 7 | Plasma Membrane | transmembrane receptor |  |  | 7305 | 22177 | 361537 |
| HLA-E | major histocompatibility complex, class I, E | HLA-E | 4.731 | 6.99E-187 | 22 | Plasma Membrane | transmembrane receptor | prognosis |  | 3133 | 667803|15040 | 294228 |
| C1QA | complement C1q A chain | C1QA | 4.657 | 0 | 13 | Extracellular Space | peptidase |  |  | 712 | 12259 | 298566 |
| HLA-DMB | major histocompatibility complex, class II, DM beta | HLA-DMB | 4.316 | 0 | 6 | Plasma Membrane | transmembrane receptor |  |  | 3109 | 15000|14999 |  |
| FCGR3A/FCGR3B | Fc fragment of IgG receptor IIIa | FCGR3A | 4.29 | 7.73E-278 | 13 | Plasma Membrane | transmembrane receptor |  | IgG, AFM13 | 2214|2215 | 246256 | 304966 |
| HLA-DRB1 | major histocompatibility complex, class II, DR beta 1 | HLA-DRB1 | 4.149 | 1.13E-202 | 6 | Plasma Membrane | transmembrane receptor |  | apolizumab | 3123 |  |  |
| IL1B | interleukin 1 beta | IL1B | 4.06 | 1.92E-135 | 9 | Extracellular Space | cytokine | diagnosis,efficacy,prognosis | canakinumab, gevokizumab, canakinumab/INS, gallium nitrate | 3553 | 16176 | 24494 |
| AIF1 | allograft inflammatory factor 1 | AIF1 | 3.94 | 6.66E-286 | 12,21 | Nucleus | other |  |  | 199 | 11629 | 29427 |
| IFI30 | IFI30, lysosomal thiol reductase | IFI30 | 3.914 | 2.2E-245 |  | Cytoplasm | enzyme |  |  | 10437 | 65972 | 290644 |
| ALOX5AP | arachidonate 5-lipoxygenase activating protein | ALOX5AP | 3.867 | 6.19E-272 | 8 | Plasma Membrane | other |  |  | 241 | 11690 | 29624 |
| FPR1 | formyl peptide receptor 1 | FPR1 | 3.815 | 3.93E-158 |  | Plasma Membrane | G-protein coupled receptor |  |  | 2357 | 14293 | 292409 |
| HLA-DMA | major histocompatibility complex, class II, DM alpha | HLA-DMA | 3.763 | 2.8E-180 | 6 | Plasma Membrane | transmembrane receptor |  |  | 3108 | 14998 |  |
| GPR183 | G protein-coupled receptor 183 | GPR183 | 3.741 | 4.07E-172 |  | Plasma Membrane | G-protein coupled receptor |  |  | 1880 | 321019 | 679975 |
| FCGR2A | Fc fragment of IgG receptor IIa | FCGR2A | 3.72 | 7E-296 | 12 | Plasma Membrane | transmembrane receptor |  | IgG | 2212 | 14131 | 116591|103693683|103694908|100911825|498276 |
| ITGB2 | integrin subunit beta 2 | ITGB2 | 3.694 | 0 | 17 | Plasma Membrane | transmembrane receptor |  |  | 3689 | 16414 | 309684 |
| C5AR1 | complement C5a receptor 1 | C5AR1 | 3.612 | 4.25E-191 |  | Plasma Membrane | G-protein coupled receptor |  |  | 728 | 12273 | 113959 |
| CXCR4 | C-X-C motif chemokine receptor 4 | CXCR4 | 3.601 | 1.25E-108 | 25 | Plasma Membrane | G-protein coupled receptor | diagnosis | AMD 11070, ulocuplumab, cladribine/cytarabine/filgrastim/idarubicin/plerixafor, POL6326, BL-8040, LY-2510924, burixafor, USL311, PF-06747143, plerixafor, filgrastim/plerixafor | 7852 | 12767 | 60628 |
| CD14 | CD14 molecule | CD14 | 3.593 | 3.09E-232 | 10 | Plasma Membrane | transmembrane receptor | efficacy,unspecified application |  | 929 | 12475 | 60350 |
| VSIG4 | V-set and immunoglobulin domain containing 4 | VSIG4 | 3.582 | 1.02E-246 |  | Plasma Membrane | other |  |  | 11326 | 278180 | 312102 |
| TBXAS1 | thromboxane A synthase 1 | TBXAS1 | 3.535 | 4.86E-289 | 6 | Plasma Membrane | enzyme |  | ridogrel | 6916 | 21391 | 24886 |
| HLA-DRB5 | major histocompatibility complex, class II, DR beta 5 | HLA-DRB5 | 3.512 | 1.23E-194 | 6,21 | Plasma Membrane | transmembrane receptor |  |  | 3127 | 14969 | 294270 |
| CYBB | cytochrome b-245 beta chain | CYBB | 3.476 | 4.47E-202 | 7 | Cytoplasm | enzyme |  |  | 1536 | 13058 | 66021 |
| HCLS1 | hematopoietic cell-specific Lyn substrate 1 | HCLS1 | 3.431 | 6E-300 | 12 | Nucleus | other |  |  | 3059 | 15163 | 288077 |
| CORO1A | coronin 1A | CORO1A | 3.416 | 1.62E-192 | 2 | Cytoplasm | other |  |  | 11151 | 12721 | 155151 |
| HLA-DQB1 | major histocompatibility complex, class II, DQ beta 1 | HLA-DQB1 | 3.348 | 3.6E-208 | 22 | Plasma Membrane | other |  |  | 3119 | 14961 | 309622 |
| HLA-DQA1 | major histocompatibility complex, class II, DQ alpha 1 | HLA-DQA1 | 3.343 | 1.22E-199 | 22 | Plasma Membrane | transmembrane receptor |  |  | 3117 | 14960 | 309621 |
| SLA | Src like adaptor | SLA | 3.293 | 3.1E-207 |  | Plasma Membrane | other |  |  | 6503 | 20491 | 338477 |
| ACSL1 | acyl-CoA synthetase long chain family member 1 | ACSL1 | 3.279 | 2.43E-128 |  | Cytoplasm | enzyme | unspecified application |  | 2180 | 14081 | 25288 |
| NPC2 | NPC intracellular cholesterol transporter 2 | NPC2 | 3.275 | 1.16E-86 | 24 | Extracellular Space | transporter |  |  | 10577 | 67963 | 286898 |
| HLA-DPB1 | major histocompatibility complex, class II, DP beta 1 | HLA-DPB1 | 3.265 | 1.07E-256 | 22 | Plasma Membrane | transmembrane receptor |  |  | 3115 |  |  |
| APOC1 | apolipoprotein C1 | APOC1 | 3.261 | 1.3E-143 | 20 | Extracellular Space | transporter | prognosis,unspecified application |  | 341 |  |  |
| MS4A6A | membrane spanning 4-domains A6A | MS4A6A | 3.235 | 5.66E-188 | 2 | Other | other |  |  | 64231 | 68774 | 361735 |
| SCIN | scinderin | SCIN | 3.195 | 1.08E-188 | 4 | Cytoplasm | other |  |  | 85477 | 20259 | 298975 |
| CD4 | CD4 molecule | CD4 | 3.191 | 4.1E-286 | 5 | Plasma Membrane | transmembrane receptor | diagnosis,efficacy,unspecified application | zanolimumab, ibalizumab | 920 | 12504 | 24932 |
| TNFRSF1B | TNF receptor superfamily member 1B | TNFRSF1B | 3.19 | 5.29E-189 | 1 | Plasma Membrane | transmembrane receptor | efficacy,safety |  | 7133 | 21938 | 156767 |
| CYBA | cytochrome b-245 alpha chain | CYBA | 3.107 | 7.09E-266 | 7 | Cytoplasm | enzyme |  |  | 1535 | 13057 | 79129 |
| MS4A7 | membrane spanning 4-domains A7 | MS4A7 | 3.098 | 3.44E-194 | 21 | Other | other |  |  | 58475 | 109225 | 293744 |
| PLEK | pleckstrin | PLEK | 3.087 | 2.61E-175 | 12 | Cytoplasm | other |  |  | 5341 | 56193 | 364206 |
| CTSS | cathepsin S | CTSS | 3.005 | 1.77E-260 |  | Cytoplasm | peptidase |  |  | 1520 | 13040 | 50654 |
| PLAUR | plasminogen activator, urokinase receptor | PLAUR | 2.982 | 8.97E-112 | 14 | Plasma Membrane | transmembrane receptor | diagnosis,disease progression |  | 5329 | 18793 | 50692 |
| LAIR1 | leukocyte associated immunoglobulin like receptor 1 | LAIR1 | 2.924 | 4.85E-226 |  | Plasma Membrane | transmembrane receptor |  |  | 3903 | 52855 | 574531 |
| THEMIS2 | thymocyte selection associated family member 2 | THEMIS2 | 2.911 | 3.9E-146 | 7 | Other | other |  |  | 9473 | 230787 | 500561 |
| CTSH | cathepsin H | CTSH | 2.834 | 6.72E-127 |  | Cytoplasm | peptidase |  |  | 1512 | 13036 | 25425 |
| REL | REL proto-oncogene, NF-kB subunit | REL | 2.831 | 7.18E-144 | 17 | Nucleus | transcription regulator |  |  | 5966 | 19696 | 305584 |
| MSR1 | macrophage scavenger receptor 1 | MSR1 | 2.796 | 3.83E-143 | 8 | Plasma Membrane | transmembrane receptor |  |  | 4481 | 20288 | 498638 |
| RAB20 | RAB20, member RAS oncogene family | RAB20 | 2.781 | 1.36E-117 | 20 | Cytoplasm | enzyme |  |  | 55647 | 19332 | 689377 |
| SIGLEC10 | sialic acid binding Ig like lectin 10 | SIGLEC10 | 2.778 | 3.28E-154 | 15 | Plasma Membrane | other |  |  | 89790 | 243958 | 292844 |
| TLR2 | toll like receptor 2 | TLR2 | 2.767 | 1.3E-158 | 10 | Plasma Membrane | transmembrane receptor | diagnosis,efficacy,unspecified application | OM 174 lipid | 7097 | 24088 | 310553 |
| UCP2 | uncoupling protein 2 | UCP2 | 2.698 | 6.44E-131 | 3 | Cytoplasm | transporter |  |  | 7351 | 22228 | 54315 |
| RNASE6 | ribonuclease A family member k6 | RNASE6 | 2.69 | 4.54E-107 |  | Extracellular Space | enzyme |  |  | 6039 | 78416 | 305842 |
| BCL2A1 | BCL2 related protein A1 | BCL2A1 | 2.649 | 3.97E-121 | 24 | Cytoplasm | other |  |  | 597 | 12044|12045|12047 | 170929 |
| SYNGR2 | synaptogyrin 2 | SYNGR2 | 2.618 | 1.86E-86 | 14 | Cytoplasm | other |  |  | 9144 | 20973 | 89815 |
| STOM | stomatin | STOM | 2.606 | 1.37E-50 | 5 | Plasma Membrane | other |  |  | 2040 | 13830 | 296655 |
| CTSC | cathepsin C | CTSC | 2.584 | 1.15E-102 |  | Cytoplasm | peptidase | unspecified application |  | 1075 | 13032 | 25423 |
| PLTP | phospholipid transfer protein | PLTP | 2.551 | 7.7E-46 | 20 | Extracellular Space | enzyme |  |  | 5360 | 18830 | 296371 |
| IL18 | interleukin 18 | IL18 | 2.522 | 8.77E-132 | 14 | Extracellular Space | cytokine | efficacy,unspecified application |  | 3606 | 16173 | 29197 |
| MGAT1 | mannosyl (alpha-1,3-)-glycoprotein beta-1,2-N-acetylglucosaminyltransferase | MGAT1 | 2.518 | 2.3E-42 | 3 | Cytoplasm | enzyme |  |  | 4245 | 17308 | 81519 |
| CD300A | CD300a molecule | CD300A | 2.509 | 7.25E-120 | 19 | Plasma Membrane | transmembrane receptor |  |  | 11314 | 217303 | 501736 |
| PPP1R15A | protein phosphatase 1 regulatory subunit 15A | PPP1R15A | 2.507 | 5.06E-22 | 3 | Cytoplasm | other |  |  | 23645 | 17872 |  |
| PLIN2 | perilipin 2 | PLIN2 | 2.502 | 3.7E-90 | 20 | Plasma Membrane | other | disease progression |  | 123 | 11520 | 298199 |
| NCKAP1L | NCK associated protein 1 like | NCKAP1L | 2.5 | 3.93E-183 | 7 | Plasma Membrane | other |  |  | 3071 | 105855 | 315348 |
| LIPA | lipase A, lysosomal acid type | LIPA | 2.475 | 1.12E-40 |  | Cytoplasm | enzyme | unspecified application |  | 3988 | 16889 | 25055 |
| SLC11A1 | solute carrier family 11 member 1 | SLC11A1 | 2.463 | 8.45E-202 | 6 | Plasma Membrane | transporter |  |  | 6556 | 18173 | 316519 |
| TNFAIP3 | TNF alpha induced protein 3 | TNFAIP3 | 2.463 | 2.15E-66 | 10 | Nucleus | enzyme |  |  | 7128 | 21929 | 683206 |
| NAPSB | napsin B aspartic peptidase, pseudogene | NAPSB | 2.442 | 3.46E-61 |  | Other | other |  |  | 256236 |  |  |
| AOAH | acyloxyacyl hydrolase | AOAH | 2.429 | 4.6E-123 | 18 | Extracellular Space | enzyme |  |  | 313 | 27052 | 498757 |
| OSM | oncostatin M | OSM | 2.412 | 2.39E-59 | 4 | Extracellular Space | cytokine | unspecified application |  | 5008 | 18413 | 289747 |
| CD86 | CD86 molecule | CD86 | 2.408 | 2.81E-122 | 10 | Plasma Membrane | transmembrane receptor | efficacy,prognosis | abatacept, belatacept, abatacept/methotrexate | 942 | 12524 | 56822 |
| MFSD1 | major facilitator superfamily domain containing 1 | MFSD1 | 2.407 | 2.61E-66 |  | Other | transporter |  |  | 64747 | 66868 | 361957 |
| DENND3 | DENN domain containing 3 | DENND3 | 2.404 | 9.19E-97 |  | Cytoplasm | other |  |  | 22898 | 105841 | 315055 |
| ZNF331 | zinc finger protein 331 | ZNF331 | 2.398 | 8.01E-91 | 19 | Nucleus | other |  |  | 55422 |  |  |
| MAN2B1 | mannosidase alpha class 2B member 1 | MAN2B1 | 2.397 | 6.38E-76 | 19 | Cytoplasm | enzyme |  |  | 4125 | 17159 | 361378 |
| SCPEP1 | serine carboxypeptidase 1 | SCPEP1 | 2.397 | 4.57E-58 | 17 | Cytoplasm | peptidase |  |  | 59342 | 74617 | 114861 |
| ARHGAP4 | Rho GTPase activating protein 4 | ARHGAP4 | 2.393 | 7.39E-183 | 22 | Cytoplasm | other |  |  | 393 | 171207 | 246249 |
| ARPC1B | actin related protein 2/3 complex subunit 1B | ARPC1B | 2.387 | 9.31E-90 | 2 | Cytoplasm | other |  |  | 10095 | 11867 | 54227 |
| GRN | granulin precursor | GRN | 2.386 | 1.83E-44 | 10 | Extracellular Space | growth factor |  |  | 2896 | 14824 | 29143 |
| ETS2 | ETS proto-oncogene 2, transcription factor | ETS2 | 2.384 | 1.65E-79 | 25 | Nucleus | transcription regulator | efficacy |  | 2114 | 23872 | 304063 |
| ICAM1 | intercellular adhesion molecule 1 | ICAM1 | 2.362 | 5.69E-53 | 1 | Plasma Membrane | transmembrane receptor | diagnosis,efficacy,prognosis,unspecified application |  | 3383 | 15894 | 25464 |
| CD55 | CD55 molecule (Cromer blood group) | CD55 | 2.345 | 1.04E-105 |  | Plasma Membrane | other |  |  | 1604 | 13136|13137 | 64036 |
| PARVG | parvin gamma | PARVG | 2.331 | 1.61E-174 | 17 | Cytoplasm | other |  |  | 64098 | 64099 | 689069 |
| RGS2 | regulator of G protein signaling 2 | RGS2 | 2.315 | 1.04E-41 | 8 | Nucleus | other |  |  | 5997 | 19735 | 84583 |
| FERMT3 | fermitin family member 3 | FERMT3 | 2.309 | 2.36E-133 | 12 | Cytoplasm | enzyme |  |  | 83706 | 108101 | 309186 |
| TMEM176B | transmembrane protein 176B | TMEM176B | 2.301 | 1.62E-37 | 6 | Other | other |  |  | 28959 | 65963 | 171411 |
| PFKFB3 | 6-phosphofructo-2-kinase/fructose-2,6-biphosphatase 3 | PFKFB3 | 2.295 | 7.25E-43 | 4 | Cytoplasm | kinase |  | PFK-158 | 5209 | 170768 | 117276 |
| LST1 | leukocyte specific transcript 1 | LST1 | 2.255 | 6.27E-163 | 14 | Plasma Membrane | other |  |  | 7940 |  |  |
| MS4A4A | membrane spanning 4-domains A4A | MS4A4A | 2.255 | 3.98E-142 | 12 | Cytoplasm | other |  |  | 51338 | 666907 |  |
| IL10RA | interleukin 10 receptor subunit alpha | IL10RA | 2.248 | 6.02E-112 | 17 | Plasma Membrane | transmembrane receptor |  |  | 3587 | 16154 | 117539 |
| KLHL6 | kelch like family member 6 | KLHL6 | 2.245 | 2.49E-142 |  | Other | other |  |  | 89857 | 239743 | 287974 |
| STAB1 | stabilin 1 | STAB1 | 2.243 | 2.03E-117 | 14,21 | Plasma Membrane | transporter |  |  | 23166 | 192187 | 100363145 |
| SERPINA1 | serpin family A member 1 | SERPINA1 | 2.239 | 5.46E-109 | 24 | Extracellular Space | other | diagnosis,unspecified application |  | 5265 | 20704|20703|20702|20701|20700 | 24648 |
| CCR1 | C-C motif chemokine receptor 1 | CCR1 | 2.2 | 2.07E-138 |  | Plasma Membrane | G-protein coupled receptor |  |  | 1230 | 12768 | 57301 |
| PYCARD | PYD and CARD domain containing | PYCARD | 2.187 | 1.45E-114 | 9 | Cytoplasm | transcription regulator | diagnosis |  | 29108 | 66824 | 282817 |
| MTHFD2 | methylenetetrahydrofolate dehydrogenase (NADP+ dependent) 2, methenyltetrahydrofolate cyclohydrolase | MTHFD2 | 2.164 | 1.57E-33 | 3 | Cytoplasm | enzyme |  |  | 10797 | 17768 | 680308 |
| RHOG | ras homolog family member G | RHOG | 2.163 | 2.89E-32 | 19 | Cytoplasm | enzyme |  |  | 391 | 56212 | 308875 |
| DUSP2 | dual specificity phosphatase 2 | DUSP2 | 2.157 | 1.42E-69 | 4 | Nucleus | phosphatase |  |  | 1844 | 13537 | 311406 |
| SERPINB1 | serpin family B member 1 | SERPINB1 | 2.15 | 1.08E-128 | 4 | Cytoplasm | other |  |  | 1992 | 66222 | 291091 |
| HCK | HCK proto-oncogene, Src family tyrosine kinase | HCK | 2.146 | 2.4E-133 | 14 | Cytoplasm | kinase |  | rebastinib, bosutinib | 3055 | 15162 | 25734 |
| NINJ1 | ninjurin 1 | NINJ1 | 2.125 | 2.56E-50 | 6 | Plasma Membrane | other | disease progression |  | 4814 | 18081 | 25338 |
| NAGA | alpha-N-acetylgalactosaminidase | NAGA | 2.115 | 2.25E-35 |  | Cytoplasm | enzyme |  |  | 4668 | 17939 | 315165 |
| MOB1A | MOB kinase activator 1A | MOB1A | 2.095 | 1.18E-56 | 16 | Plasma Membrane | other |  |  | 55233 | 232157 | 297387 |
| IFITM2 | interferon induced transmembrane protein 2 | IFITM2 | 2.091 | 4.61E-68 | 5 | Cytoplasm | other | prognosis |  | 10581 | 80876 | 114709 |
| C1orf162 | chromosome 1 open reading frame 162 | C1orf162 | 2.071 | 4.78E-94 |  | Other | transporter |  |  | 128346 | 433638 | 100911379|100363228 |
| RPS4X | ribosomal protein S4, X-linked | RPS4X | 2.055 | 5.15E-38 | 8 | Cytoplasm | other | diagnosis |  | 6191 |  |  |
| ALOX5 | arachidonate 5-lipoxygenase | ALOX5 | 2.05 | 3.12E-135 | 8 | Cytoplasm | enzyme | diagnosis,efficacy | TA 270, benoxaprofen, diclofenac/omeprazole, diclofenac/misoprostol, diclofenac, diethylcarbamazine, meclofenamic acid, zileuton, sulfasalazine, balsalazide, mesalamine, nordihydroguaiaretic acid, masoprocol | 240 | 11689 | 25290 |
| FGL2 | fibrinogen like 2 | FGL2 | 2.039 | 8.27E-145 | 2 | Extracellular Space | peptidase |  |  | 10875 | 14190 | 84586 |
| CPVL | carboxypeptidase, vitellogenic like | CPVL | 2.035 | 1.26E-40 | 16 | Cytoplasm | peptidase |  |  | 54504 | 71287 | 502774 |
| TNFSF13 | TNF superfamily member 13 | TNFSF13 | 2.032 | 8.32E-56 | 10 | Extracellular Space | cytokine |  |  | 8741 | 69583 | 287437 |
| RGS19 | regulator of G protein signaling 19 | RGS19 | 2.022 | 4.09E-49 | 8 | Cytoplasm | other |  |  | 10287 | 56470 | 59293 |
| HMOX1 | heme oxygenase 1 | HMOX1 | 2.021 | 3.35E-60 | 25 | Cytoplasm | enzyme | efficacy,safety,unspecified application | tin mesoporphyrin | 3162 | 15368 | 24451 |
| GGTA1P | glycoprotein, alpha-galactosyltransferase 1 pseudogene | GGTA1P | 1.999 | 1.2E-87 |  | Cytoplasm | other |  |  | 2681 |  |  |
| MSN | moesin | MSN | 1.99 | 4.65E-34 | 4 | Plasma Membrane | other |  |  | 4478 | 17698 | 81521 |
| RASSF5 | Ras association domain family member 5 | RASSF5 | 1.985 | 6.74E-111 | 16 | Plasma Membrane | other |  |  | 83593 | 54354 | 54355 |
| PILRA | paired immunoglobin like type 2 receptor alpha | PILRA | 1.978 | 1.69E-84 | 9 | Plasma Membrane | other |  |  | 29992 | 231805 | 100910669|100910497 |
| SAMSN1 | SAM domain, SH3 domain and nuclear localization signals 1 | SAMSN1 | 1.978 | 9.78E-82 | 2 | Nucleus | other |  |  | 64092 | 67742 | 170637 |
| GAA | glucosidase alpha, acid | GAA | 1.97 | 1.27E-25 | 19 | Cytoplasm | enzyme | diagnosis | miglitol, acarbose | 2548 | 14387 | 367562 |
| DOCK2 | dedicator of cytokinesis 2 | DOCK2 | 1.953 | 9.57E-110 | 19 | Cytoplasm | other |  |  | 1794 | 94176 | 360509 |
| RIN3 | Ras and Rab interactor 3 | RIN3 | 1.939 | 2.65E-128 | 8 | Cytoplasm | other |  |  | 79890 | 217835 | 314397 |
| ADA2 | adenosine deaminase 2 | CECR1 | 1.908 | 2.99E-77 | 14 | Extracellular Space | enzyme |  |  | 51816 |  |  |
| RASGEF1B | RasGEF domain family member 1B | RASGEF1B | 1.905 | 5.78E-81 |  | Other | other |  |  | 153020 | 320292 | 100361238 |
| TAGAP | T cell activation RhoGTPase activating protein | TAGAP | 1.875 | 1.03E-88 | 4 | Cytoplasm | other |  |  | 117289 | 72536 | 308097 |
| CMTM7 | CKLF like MARVEL transmembrane domain containing 7 | CMTM7 | 1.868 | 9.39E-84 |  | Extracellular Space | cytokine |  |  | 112616 | 102545 | 501065 |
| CLIC1 | chloride intracellular channel 1 | CLIC1 | 1.861 | 2.75E-119 | 8 | Nucleus | ion channel |  |  | 1192 | 114584 | 406864 |
| FAM49B | family with sequence similarity 49 member B | FAM49B | 1.859 | 1.36E-51 | 17 | Extracellular Space | other |  |  | 51571 | 223601 | 299909 |
| TAPBP | TAP binding protein | TAPBP | 1.851 | 8.25E-43 | 22 | Cytoplasm | transporter |  |  | 6892 | 21356 | 25217 |
| VAMP8 | vesicle associated membrane protein 8 | VAMP8 | 1.83 | 2.58E-74 | 24 | Plasma Membrane | transporter |  |  | 8673 | 22320 | 83730 |
| NFKBIZ | NFKB inhibitor zeta | NFKBIZ | 1.826 | 6.28E-40 | 23 | Nucleus | transcription regulator |  |  | 64332 | 80859 | 304005 |
| LTBR | lymphotoxin beta receptor | LTBR | 1.814 | 1.27E-49 | 24 | Plasma Membrane | transmembrane receptor |  |  | 4055 | 17000 | 297604 |
| CYTH4 | cytohesin 4 | CYTH4 | 1.811 | 2.86E-92 | 21 | Cytoplasm | other |  |  | 27128 | 72318 | 500906 |
| SP100 | SP100 nuclear antigen | SP100 | 1.81 | 3.7E-36 | 16 | Nucleus | transcription regulator |  |  | 6672 |  |  |
| RNF149 | ring finger protein 149 | RNF149 | 1.809 | 5.15E-31 | 6 | Cytoplasm | enzyme |  |  | 284996 | 67702 | 363222 |
| FXYD5 | FXYD domain containing ion transport regulator 5 | FXYD5 | 1.799 | 4E-56 | 14 | Plasma Membrane | ion channel |  |  | 53827 | 18301 | 60338 |
| STAT6 | signal transducer and activator of transcription 6 | STAT6 | 1.787 | 4.1E-124 | 14 | Nucleus | transcription regulator | prognosis,response to therapy |  | 6778 | 20852 | 362896 |
| HLA-F | major histocompatibility complex, class I, F | HLA-F | 1.775 | 9.53E-75 | 22 | Plasma Membrane | transmembrane receptor |  |  | 3134 | 100529082|630294 |  |
| NCOA4 | nuclear receptor coactivator 4 | NCOA4 | 1.774 | 2.42E-52 | 20 | Nucleus | transcription regulator |  |  | 8031 | 27057 | 619385 |
| RCSD1 | RCSD domain containing 1 | RCSD1 | 1.772 | 1.33E-88 |  | Other | other |  |  | 92241 | 226594 | 360872 |
| GNA13 | G protein subunit alpha 13 | GNA13 | 1.767 | 2.91E-64 | 11 | Plasma Membrane | enzyme |  |  | 10672 | 14674 | 303634 |
| RB1 | RB transcriptional corepressor 1 | RB1 | 1.754 | 9.13E-49 |  | Nucleus | transcription regulator | diagnosis,efficacy,prognosis,unspecified application |  | 5925 | 19645 | 24708 |
| LILRB1 | leukocyte immunoglobulin like receptor B1 | LILRB1 | 1.74 | 1.69E-118 | 22 | Plasma Membrane | transmembrane receptor |  |  | 10859 |  |  |
| SEC14L1 | SEC14 like lipid binding 1 | SEC14L1 | 1.735 | 2.66E-34 | 21 | Cytoplasm | transporter |  |  | 6397 | 74136 | 360668 |
| SLC31A2 | solute carrier family 31 member 2 | SLC31A2 | 1.735 | 1.32E-29 |  | Plasma Membrane | transporter |  |  | 1318 | 20530 | 298091 |
| ITPRIP | inositol 1,4,5-trisphosphate receptor interacting protein | ITPRIP | 1.734 | 4.17E-87 | 9 | Extracellular Space | other |  |  | 85450 | 414801 | 100912218 |
| SIGLEC9 | sialic acid binding Ig like lectin 9 | SIGLEC9 | 1.721 | 1.6E-90 | 13 | Plasma Membrane | other |  |  | 27180 | 83382 |  |
| LGMN | legumain | LGMN | 1.715 | 1.72E-21 | 5 | Cytoplasm | peptidase |  |  | 5641 | 19141 | 63865 |
| CMTM6 | CKLF like MARVEL transmembrane domain containing 6 | CMTM6 | 1.701 | 4.8E-29 | 3 | Extracellular Space | cytokine |  |  | 54918 | 67213 | 316035 |
| TYMP | thymidine phosphorylase | TYMP | 1.697 | 2.51E-112 | 4 | Extracellular Space | growth factor | diagnosis,efficacy,prognosis,response to therapy |  | 1890 | 72962 | 315219 |
| SKAP2 | src kinase associated phosphoprotein 2 | SKAP2 | 1.671 | 1.54E-49 | 12 | Cytoplasm | other |  |  | 8935 | 54353 | 155183 |
| AKAP13 | A-kinase anchoring protein 13 | AKAP13 | 1.67 | 7.65E-46 | 11 | Cytoplasm | other |  |  | 11214 | 75547 | 293024 |
| GPR84 | G protein-coupled receptor 84 | GPR84 | 1.67 | 3.31E-46 |  | Plasma Membrane | G-protein coupled receptor |  |  | 53831 | 80910 | 688730 |
| VAMP3 | vesicle associated membrane protein 3 | VAMP3 | 1.65 | 5.54E-28 | 24 | Plasma Membrane | other |  |  | 9341 | 22319 | 29528 |
| EBI3 | Epstein-Barr virus induced 3 | EBI3 | 1.633 | 1.17E-46 | 22 | Extracellular Space | cytokine |  |  | 10148 | 50498 | 680609 |
| TCIRG1 | T cell immune regulator 1, ATPase H+ transporting V0 subunit a3 | TCIRG1 | 1.632 | 1.02E-41 | 2 | Plasma Membrane | enzyme |  |  | 10312 | 27060 | 293650 |
| ACTR2 | ARP2 actin related protein 2 homolog | ACTR2 | 1.626 | 4.13E-38 | 2 | Plasma Membrane | other |  |  | 10097 | 66713 | 289820 |
| ENG | endoglin | ENG | 1.622 | 4.18E-43 |  | Plasma Membrane | transmembrane receptor | disease progression,efficacy | TRC105 | 2022 | 13805 | 497010 |
| SLC7A5 | solute carrier family 7 member 5 | SLC7A5 | 1.617 | 5.01E-21 | 9 | Plasma Membrane | transporter | diagnosis |  | 8140 | 20539 | 50719 |
| CD164 | CD164 molecule | CD164 | 1.616 | 1.16E-27 | 2 | Plasma Membrane | other |  |  | 8763 | 53599 | 83689 |
| PLAC8 | placenta specific 8 | PLAC8 | 1.609 | 2.17E-78 | 12 | Nucleus | other |  |  | 51316 | 231507 | 360914 |
| NABP1 | nucleic acid binding protein 1 | NABP1 | 1.603 | 3.06E-100 | 5 | Nucleus | other |  |  | 64859 | 109019 | 363227 |
| JAK3 | Janus kinase 3 | JAK3 | 1.599 | 2.59E-139 | 4 | Cytoplasm | kinase | efficacy | tofacitinib, R-348, methotrexate/tofacitinib | 3718 | 16453 | 25326 |
| HLA-DOA | major histocompatibility complex, class II, DO alpha | HLA-DOA | 1.591 | 2.98E-113 | 6 | Plasma Membrane | transmembrane receptor |  |  | 3111 | 15001 | 24984 |
| CPM | carboxypeptidase M | CPM | 1.56 | 4.93E-59 | 2 | Plasma Membrane | peptidase |  |  | 1368 | 70574 | 314855 |
| RPL28 | ribosomal protein L28 | RPL28 | 1.559 | 2.48E-32 | 1 | Cytoplasm | other |  |  | 6158 | 19943 | 64638 |
| SLC16A3 | solute carrier family 16 member 3 | SLC16A3 | 1.559 | 7.68E-57 | 9 | Plasma Membrane | transporter |  |  | 9123 | 80879 | 80878 |
| MTCH1 | mitochondrial carrier 1 | MTCH1 | 1.553 | 2.42E-40 | 9 | Cytoplasm | other |  |  | 23787 | 56462 | 294313 |
| CD58 | CD58 molecule | CD58 | 1.537 | 3.19E-25 | 19 | Plasma Membrane | transmembrane receptor |  |  | 965 |  |  |
| THBD | thrombomodulin | THBD | 1.534 | 5E-52 | 10 | Plasma Membrane | transmembrane receptor | efficacy |  | 7056 | 21824 | 83580 |
| TMBIM1 | transmembrane BAX inhibitor motif containing 1 | TMBIM1 | 1.534 | 9.09E-26 |  | Cytoplasm | other |  |  | 64114 | 69660 | 316516 |
| MAFB | MAF bZIP transcription factor B | MAFB | 1.531 | 1.96E-59 | 7 | Nucleus | transcription regulator |  |  | 9935 | 16658 | 54264 |
| MPP1 | membrane palmitoylated protein 1 | MPP1 | 1.52 | 1.65E-27 | 11 | Plasma Membrane | kinase |  |  | 4354 | 17524 |  |
| SPINT2 | serine peptidase inhibitor, Kunitz type 2 | SPINT2 | 1.519 | 2.87E-42 | 3 | Extracellular Space | other | diagnosis |  | 10653 | 20733 | 292770 |
| MYL12A | myosin light chain 12A | MYL12A | 1.501 | 1.85E-82 |  | Cytoplasm | other | unspecified application |  | 10627 | 67938 | 50685 |
| EIF4B | eukaryotic translation initiation factor 4B | EIF4B | 1.488 | 1.77E-33 | 8 | Cytoplasm | translation regulator | unspecified application |  | 1975 | 75705 | 300253 |
| STX11 | syntaxin 11 | STX11 | 1.487 | 8.94E-50 | 24 | Plasma Membrane | transporter |  |  | 8676 | 74732 | 292483 |
| MYO1F | myosin IF | MYO1F | 1.476 | 8.06E-76 | 13 | Cytoplasm | other |  |  | 4542 | 17916 | 314654 |
| RNF130 | ring finger protein 130 | RNF130 | 1.476 | 4.15E-30 | 2 | Cytoplasm | peptidase |  |  | 55819 | 59044 | 652955 |
| FGR | FGR proto-oncogene, Src family tyrosine kinase | FGR | 1.472 | 4.41E-60 | 12 | Nucleus | kinase |  | vemurafenib, rebastinib, cobimetinib/vemurafenib, dabrafenib/trametinib/vemurafenib, cetuximab/vemurafenib, trametinib/vemurafenib | 2268 | 14191 | 79113 |
| CTSL | cathepsin L | CTSL1 | 1.468 | 7.51E-21 |  | Cytoplasm | peptidase |  | cathepsin L inhibitor | 1514 |  |  |
| CD163 | CD163 molecule | CD163 | 1.457 | 1.77E-50 | 20 | Plasma Membrane | transmembrane receptor |  |  | 9332 | 93671 | 312701 |
| RBM47 | RNA binding motif protein 47 | RBM47 | 1.448 | 9.5E-62 | 7,18 | Nucleus | other |  |  | 54502 | 245945 | 305340 |
| EMB | embigin | EMB | 1.442 | 4.76E-80 | 11 | Plasma Membrane | transporter |  |  | 133418 | 13723 | 114511 |
| TMEM176A | transmembrane protein 176A | TMEM176A | 1.43 | 2.58E-36 |  | Other | other |  |  | 55365 | 66058 | 297077 |
| TUBB | tubulin beta class I | TUBB | 1.423 | 1.03E-66 | 1 | Cytoplasm | other | efficacy | ixabepilone | 203068 | 22154 | 29214 |
| CD33 | CD33 molecule | CD33 | 1.417 | 5.05E-62 | 13 | Plasma Membrane | other | efficacy,unspecified application | lintuzumab, arsenic trioxide/gemtuzumab ozogamicin/tretinoin, anti-CD33 monoclonal antibody, BI 836858, vadastuximab talirine, actinium Ac 225 lintuzumab, cytarabine/daunorubicin/etoposide/gemtuzumab ozogamicin, gemtuzumab ozogamicin | 945 |  |  |
| FGD4 | FYVE, RhoGEF and PH domain containing 4 | FGD4 | 1.417 | 3.94E-61 | 15 | Cytoplasm | other |  |  | 121512 | 224014 | 246174 |
| PIK3IP1 | phosphoinositide-3-kinase interacting protein 1 | PIK3IP1 | 1.417 | 2.03E-30 | 12 | Cytoplasm | other |  |  | 113791 | 216505 | 305472 |
| BST2 | bone marrow stromal cell antigen 2 | BST2 | 1.414 | 3.56E-52 | 16 | Plasma Membrane | other |  |  | 684 |  |  |
| HCST | hematopoietic cell signal transducer | HCST | 1.412 | 3.19E-42 | 2,21 | Plasma Membrane | transmembrane receptor |  |  | 10870 | 23900 | 474146 |
| GRB2 | growth factor receptor bound protein 2 | GRB2 | 1.397 | 4.03E-46 | 2 | Cytoplasm | kinase |  | liposome-incorporated Grb2 antisense oligodeoxynucleotide | 2885 | 14784 | 81504 |
| OSTF1 | osteoclast stimulating factor 1 | OSTF1 | 1.395 | 4.87E-26 | 13 | Nucleus | transcription regulator |  |  | 26578 | 20409 | 259275 |
| SASH3 | SAM and SH3 domain containing 3 | SASH3 | 1.388 | 5.57E-53 | 15 | Cytoplasm | other |  |  | 54440 | 74131 | 317578 |
| STXBP2 | syntaxin binding protein 2 | STXBP2 | 1.386 | 4.09E-76 | 24 | Plasma Membrane | transporter |  |  | 6813 | 20911 | 81804 |
| ANKRD22 | ankyrin repeat domain 22 | ANKRD22 | 1.384 | 3.55E-73 |  | Nucleus | transcription regulator |  |  | 118932 | 52024 | 294093 |
| EIF2S3 | eukaryotic translation initiation factor 2 subunit gamma | EIF2S3 | 1.381 | 3.27E-46 | 1 | Cytoplasm | translation regulator |  |  | 1968 | 26905 | 299027 |
| TMEM109 | transmembrane protein 109 | TMEM109 | 1.369 | 5.22E-26 | 9 | Cytoplasm | other |  |  | 79073 | 68539 | 361732 |
| MANBA | mannosidase beta | MANBA | 1.357 | 5.88E-53 | 18 | Cytoplasm | enzyme |  |  | 4126 | 110173 | 310864 |
| ACTR3 | ARP3 actin related protein 3 homolog | ACTR3 | 1.356 | 6.32E-33 | 2 | Plasma Membrane | other | diagnosis |  | 10096 | 74117 | 81732 |
| PAPOLG | poly(A) polymerase gamma | PAPOLG | 1.351 | 2.21E-50 | 3 | Nucleus | enzyme |  |  | 64895 | 216578 | 305586 |
| LYZ | lysozyme | LYZ | 1.348 | 4.92E-32 | 25 | Extracellular Space | enzyme | unspecified application |  | 4069 | 17110|17105 | 25211 |
| ZC3H12A | zinc finger CCCH-type containing 12A | ZC3H12A | 1.348 | 3.91E-32 | 15 | Cytoplasm | enzyme |  |  | 80149 | 230738 | 313587 |
| HSD17B11 | hydroxysteroid 17-beta dehydrogenase 11 | HSD17B11 | 1.341 | 8.71E-30 | 17 | Cytoplasm | enzyme |  |  | 51170 | 114664 | 289456 |
| SAMHD1 | SAM and HD domain containing deoxynucleoside triphosphate triphosphohydrolase 1 | SAMHD1 | 1.339 | 1.7E-30 | 17 | Nucleus | enzyme |  |  | 25939 | 56045 | 311580 |
| SLC29A1 | solute carrier family 29 member 1 (Augustine blood group) | SLC29A1 | 1.337 | 9.21E-22 | 19 | Plasma Membrane | transporter | efficacy,response to therapy |  | 2030 | 63959 | 63997 |
| LRRFIP1 | LRR binding FLII interacting protein 1 | LRRFIP1 | 1.326 | 1.52E-41 | 23 | Cytoplasm | transcription regulator |  |  | 9208 | 16978 |  |
| KLF4 | Kruppel like factor 4 | KLF4 | 1.325 | 9.74E-26 | 3 | Nucleus | transcription regulator |  |  | 9314 | 16600 | 114505 |
| ANXA11 | annexin A11 | ANXA11 | 1.322 | 6.96E-54 | 19 | Nucleus | other |  |  | 311 | 11744 | 290527 |
| SYTL3 | synaptotagmin like 3 | SYTL3 | 1.319 | 2.55E-42 | 13 | Cytoplasm | other |  |  | 94120 | 83672 | 499017 |
| DOK3 | docking protein 3 | DOK3 | 1.306 | 8.59E-29 | 23 | Cytoplasm | other |  |  | 79930 | 27261 | 306760 |
| CALM1 (includes others) | calmodulin 1 | CALM1 | 1.302 | 3.19E-55 | 5 | Cytoplasm | other |  |  | 801|805|808 |  |  |
| ETV3 | ETS variant 3 | ETV3 | 1.279 | 8.65E-40 | 7 | Nucleus | transcription regulator |  |  | 2117 | 27049 | 295297 |
| SELL | selectin L | SELL | 1.275 | 8.49E-59 | 14 | Plasma Membrane | transmembrane receptor | efficacy |  | 6402 | 20343 | 29259 |
| TMEM173 | transmembrane protein 173 | TMEM173 | 1.274 | 7.3E-59 | 10 | Cytoplasm | other |  | MK-1454, MIW815 | 340061 | 72512 | 498840 |
| LRRC25 | leucine rich repeat containing 25 | LRRC25 | 1.269 | 7.49E-65 |  | Other | other |  |  | 126364 | 211228 | 498605 |
| RPSA | ribosomal protein SA | RPSA | 1.262 | 5.56E-49 | 1 | Cytoplasm | translation regulator |  |  | 3921 | 16785 | 29236 |
| GPSM3 | G protein signaling modulator 3 | GPSM3 | 1.261 | 6.18E-85 | 12 | Cytoplasm | other |  |  | 63940 | 106512 | 406163 |
| MGST2 | microsomal glutathione S-transferase 2 | MGST2 | 1.255 | 1.68E-33 | 8 | Cytoplasm | enzyme |  |  | 4258 | 211666 | 295037 |
| LPXN | leupaxin | LPXN | 1.254 | 3.6E-27 |  | Cytoplasm | transcription regulator |  |  | 9404 | 107321 | 293783 |
| RBM3 | RNA binding motif protein 3 | RBM3 | 1.254 | 5.01E-26 | 1 | Cytoplasm | other |  |  | 5935 | 100043257|19652 | 114488 |
| PPARD | peroxisome proliferator activated receptor delta | PPARD | 1.247 | 4.91E-27 |  | Nucleus | ligand-dependent nuclear receptor | diagnosis | treprostinil, icosapent, GW501516, bezafibrate | 5467 | 19015 | 25682 |
| MAGT1 | magnesium transporter 1 | MAGT1 | 1.242 | 1.5E-31 |  | Plasma Membrane | enzyme |  |  | 84061 | 67075 | 116967 |
| C2 | complement C2 | C2 | 1.241 | 1.55E-50 | 25 | Extracellular Space | peptidase |  |  | 717 | 12263 | 24231 |
| FCGBP | Fc fragment of IgG binding protein | FCGBP | 1.237 | 5.04E-47 | 13 | Extracellular Space | other |  |  | 8857 | 215384 | 100303643 |
| MAP2K3 | mitogen-activated protein kinase kinase 3 | MAP2K3 | 1.232 | 3.66E-21 |  | Cytoplasm | kinase |  |  | 5606 | 26397 | 303200 |
| CCRL2 | C-C motif chemokine receptor like 2 | CCRL2 | 1.218 | 5.69E-34 |  | Plasma Membrane | G-protein coupled receptor |  |  | 9034 | 54199 | 316019 |
| SCIMP | SLP adaptor and CSK interacting membrane protein | SCIMP | 1.211 | 4.49E-70 | 6 | Plasma Membrane | other |  |  | 388325 | 327957 | 691993 |
| ROCK1 | Rho associated coiled-coil containing protein kinase 1 | ROCK1 | 1.206 | 6.47E-48 |  | Cytoplasm | kinase |  |  | 6093 | 19877 | 81762 |
| RPL22 | ribosomal protein L22 | RPL22 | 1.206 | 8.6E-52 | 1 | Nucleus | other |  |  | 6146 | 19934 | 81768 |
| HACD4 | 3-hydroxyacyl-CoA dehydratase 4 | PTPLAD2 | 1.205 | 6.2E-55 | 21 | Cytoplasm | enzyme |  |  | 401494 | 66775 | 362540 |
| SYAP1 | synapse associated protein 1 | SYAP1 | 1.205 | 2.69E-28 | 18 | Nucleus | other |  |  | 94056 | 67043 | 302678 |
| CSGALNACT1 | chondroitin sulfate N-acetylgalactosaminyltransferase 1 | CSGALNACT1 | 1.192 | 2.35E-21 | 20 | Cytoplasm | enzyme |  |  | 55790 | 234356 | 306375 |
| NUDT16 | nudix hydrolase 16 | NUDT16 | 1.185 | 4.04E-25 | 4 | Nucleus | enzyme |  |  | 131870 | 75686 | 363129 |
| PLBD1 | phospholipase B domain containing 1 | PLBD1 | 1.182 | 7.86E-34 |  | Extracellular Space | enzyme |  |  | 79887 | 66857 | 297694 |
| EEF1G | eukaryotic translation elongation factor 1 gamma | EEF1G | 1.176 | 9.04E-30 | 1 | Cytoplasm | translation regulator |  |  | 1937 | 67160 | 293725 |
| RHOH | ras homolog family member H | RHOH | 1.176 | 1.6E-54 | 17,23 | Plasma Membrane | enzyme |  |  | 399 | 74734 | 305341 |
| SOD2 | superoxide dismutase 2 | SOD2 | 1.175 | 1.91E-33 | 3 | Cytoplasm | enzyme | diagnosis,unspecified application |  | 6648 | 20656 | 24787 |
| F13A1 | coagulation factor XIII A chain | F13A1 | 1.169 | 3.18E-35 |  | Extracellular Space | enzyme |  |  | 2162 | 74145 | 60327 |
| SIRPA | signal regulatory protein alpha | SIRPA | 1.16 | 2.67E-23 | 4 | Plasma Membrane | phosphatase |  |  | 140885 | 19261 | 25528 |
| NCF4 | neutrophil cytosolic factor 4 | NCF4 | 1.156 | 1.37E-32 | 7 | Cytoplasm | enzyme |  |  | 4689 | 17972 | 500904 |
| ADGRE2 | adhesion G protein-coupled receptor E2 | EMR2 | 1.153 | 4.36E-59 |  | Plasma Membrane | other |  |  | 30817 |  |  |
| ARHGAP18 | Rho GTPase activating protein 18 | ARHGAP18 | 1.146 | 5.93E-57 | 6 | Cytoplasm | other |  |  | 93663 | 73910 | 293947 |
| H3F3A/H3F3B | H3 histone family member 3A | H3F3A | 1.144 | 8.06E-34 | 16 | Nucleus | other | diagnosis |  | 3020|3021 | 15078|15081 | 117056|100361558 |
| PTPN1 | protein tyrosine phosphatase, non-receptor type 1 | PTPN1 | 1.144 | 6.99E-29 | 4 | Cytoplasm | phosphatase | prognosis | protein tyrosine phosphatase 1B inhibitor, trodusquemine | 5770 | 19246 | 24697 |
| CD300C | CD300c molecule | CD300C | 1.141 | 2.66E-44 | 6 | Plasma Membrane | transmembrane receptor |  |  | 10871 | 387565|140497 | 287813|501742|498022|100909671|303666|501745 |
| GADD45B | growth arrest and DNA damage inducible beta | GADD45B | 1.133 | 1.12E-21 |  | Cytoplasm | other |  |  | 4616 | 17873 | 299626 |
| ABL2 | ABL proto-oncogene 2, non-receptor tyrosine kinase | ABL2 | 1.129 | 8.33E-34 | 7 | Cytoplasm | kinase |  | nilotinib, dasatinib | 27 | 11352 | 304883 |
| SPHK1 | sphingosine kinase 1 | SPHK1 | 1.129 | 1.39E-21 | 5 | Cytoplasm | kinase | diagnosis,disease progression,prognosis,unspecified application |  | 8877 | 20698 | 170897 |
| LY96 | lymphocyte antigen 96 | LY96 | 1.108 | 1.11E-30 | 15 | Plasma Membrane | transmembrane receptor |  |  | 23643 | 17087 | 448830 |
| CMKLR1 | chemerin chemokine-like receptor 1 | CMKLR1 | 1.101 | 5.25E-51 |  | Plasma Membrane | G-protein coupled receptor |  |  | 1240 | 14747 | 60669 |
| RNF144B | ring finger protein 144B | RNF144B | 1.1 | 2.44E-65 | 20 | Cytoplasm | enzyme |  |  | 255488 | 218215 | 364681 |
| IGFLR1 | IGF like family receptor 1 | IGFLR1 | 1.095 | 1.12E-33 | 17 | Plasma Membrane | other |  |  | 79713 | 101883 | 499126 |
| CALHM6 | calcium homeostasis modulator family member 6 | FAM26F | 1.093 | 3.87E-25 | 17 | Other | other |  |  | 441168 | 215900 | 294430 |
| PSTPIP2 | proline-serine-threonine phosphatase interacting protein 2 | PSTPIP2 | 1.088 | 4.79E-76 | 9 | Cytoplasm | other |  |  | 9050 | 19201 | 307248 |
| NUP98 | nucleoporin 98 | NUP98 | 1.086 | 9.83E-25 | 21 | Nucleus | transporter |  |  | 4928 | 269966 | 81738 |
| TRPM2 | transient receptor potential cation channel subfamily M member 2 | TRPM2 | 1.085 | 2.53E-62 | 24 | Plasma Membrane | ion channel |  |  | 7226 | 28240 | 294329 |
| TREM1 | triggering receptor expressed on myeloid cells 1 | TREM1 | 1.082 | 8.15E-38 | 10 | Plasma Membrane | transmembrane receptor | efficacy |  | 54210 | 58217 | 301229 |
| HK1 | hexokinase 1 | HK1 | 1.081 | 1.05E-24 |  | Cytoplasm | kinase |  |  | 3098 | 15275 | 25058 |
| FLI1 | Fli-1 proto-oncogene, ETS transcription factor | FLI1 | 1.073 | 4.66E-67 | 13 | Nucleus | transcription regulator |  |  | 2313 | 14247 | 315532 |
| MAPKAPK3 | mitogen-activated protein kinase-activated protein kinase 3 | MAPKAPK3 | 1.068 | 2.92E-29 | 11 | Nucleus | kinase | unspecified application |  | 7867 | 102626 | 315994 |
| UNC93B1 | unc-93 homolog B1, TLR signaling regulator | UNC93B1 | 1.065 | 8.27E-40 | 11 | Cytoplasm | transporter |  |  | 81622 | 54445 | 361689 |
| GPRIN3 | GPRIN family member 3 | GPRIN3 | 1.063 | 1.09E-71 | 18 | Other | other |  |  | 285513 | 243385 | 502784 |
| LYN | LYN proto-oncogene, Src family tyrosine kinase | LYN | 1.062 | 2.12E-60 | 14 | Cytoplasm | kinase |  | bafetinib, nintedanib, JNJ-26483327, rebastinib, docetaxel/nintedanib, bosutinib, tolimidone | 4067 | 17096 | 81515 |
| PIK3AP1 | phosphoinositide-3-kinase adaptor protein 1 | PIK3AP1 | 1.057 | 1.49E-54 | 13,17 | Cytoplasm | kinase |  |  | 118788 | 83490 | 294048 |
| ARHGAP9 | Rho GTPase activating protein 9 | ARHGAP9 | 1.049 | 6.2E-37 | 7 | Cytoplasm | other |  |  | 64333 | 216445 | 362893 |
| ALOX15B | arachidonate 15-lipoxygenase, type B | ALOX15B | 1.047 | 1.75E-39 | 8 | Cytoplasm | enzyme | diagnosis,disease progression |  | 247 | 11688 | 266604 |
| ABHD16A | abhydrolase domain containing 16A | ABHD16A | 1.043 | 3.34E-26 |  | Other | other |  |  | 7920 | 193742 | 361796 |
| EIF3L | eukaryotic translation initiation factor 3 subunit L | EIF3L | 1.043 | 5.16E-49 | 8 | Cytoplasm | other |  |  | 51386 | 223691 |  |
| SP110 | SP110 nuclear body protein | SP110 | 1.042 | 9.9E-21 | 20 | Nucleus | other |  |  | 3431 | 546061|109032|624083|677525|101056250|102638047 | 301570 |
| EIF3A | eukaryotic translation initiation factor 3 subunit A | EIF3A | 1.04 | 7.76E-29 | 8 | Cytoplasm | other |  |  | 8661 | 13669 | 292148 |
| PPP1R3B | protein phosphatase 1 regulatory subunit 3B | PPP1R3B | 1.04 | 1.12E-33 |  | Cytoplasm | other |  |  | 79660 | 244416 | 192280 |
| PTGER4 | prostaglandin E receptor 4 | PTGER4 | 1.036 | 8.93E-45 | 4 | Plasma Membrane | G-protein coupled receptor | unspecified application | misoprostol, prostaglandin E2, prostaglandin E1, E7046, diclofenac/misoprostol, rivenprost | 5734 | 19219 | 84023 |
| TM9SF2 | transmembrane 9 superfamily member 2 | TM9SF2 | 1.035 | 3.64E-28 | 11 | Plasma Membrane | transporter |  |  | 9375 | 68059 | 306197 |
| TMCC3 | transmembrane and coiled-coil domain family 3 | TMCC3 | 1.029 | 1.39E-40 | 21 | Other | other |  |  | 57458 | 319880 | 314751 |
| STK10 | serine/threonine kinase 10 | STK10 | 1.027 | 1.18E-39 | 23 | Cytoplasm | kinase |  |  | 6793 | 20868 | 29398 |
| FLII | FLII, actin remodeling protein | FLII | 1.021 | 2.24E-22 | 23 | Nucleus | other |  |  | 2314 | 14248 | 287375 |
| SLC25A3 | solute carrier family 25 member 3 | SLC25A3 | 1 | 5.23E-32 | 2 | Cytoplasm | transporter |  |  | 5250 | 18674 | 245959 |

Cluster 7 microglia

| © 2000-2018 QIAGEN. All rights reserved. |  |  |  |  |  |  |  |  |  |  |  |  |
| --- | --- | --- | --- | --- | --- | --- | --- | --- | --- | --- | --- | --- |
| Symbol | Entrez Gene Name | Gene Symbol - human (HUGO / HGNC / Entrez Gene)/Gene Symbol - mouse (Entrez Gene)/Gene Symbol - rat (Entrez Gene) | Expr Log Ratio | Expr False Discovery Rate (q-value) | Networks | Location | Type(s) | Biomarker Application(s) | Drug(s) | Entrez Gene ID for Human | Entrez Gene ID for Mouse | Entrez Gene ID for Rat |
| HNRNPH1 | heterogeneous nuclear ribonucleoprotein H1 | HNRNPH1 | 2.351 | 7.54E-68 | 1 | Nucleus | other | diagnosis |  | 3187 | 59013 |  |
| TRA2A | transformer 2 alpha homolog | TRA2A | 1.99 | 1.05E-60 | 3 | Nucleus | other |  |  | 29896 | 101214 | 500116 |
| CKB | creatine kinase B | CKB | 1.957 | 8.18E-45 | 4 | Cytoplasm | kinase | safety |  | 1152 | 12709 | 24264 |
| ORC4 | origin recognition complex subunit 4 | ORC4 | 1.775 | 1.54E-77 | 2 | Nucleus | other |  |  | 5000 | 26428 | 295596 |
| SHISA9 | shisa family member 9 | SHISA9 | 1.748 | 3.15E-127 | 10 | Plasma Membrane | other |  |  | 729993 | 72555 | 100361134 |
| ARGLU1 | arginine and glutamate rich 1 | ARGLU1 | 1.722 | 1.88E-28 | 2 | Other | other |  |  | 55082 | 234023 | 290912 |
| TMEM212 | transmembrane protein 212 | TMEM212 | 1.669 | 1.62E-115 |  | Other | other |  |  | 389177 | 208613 | 499586 |
| SON | SON DNA binding protein | SON | 1.653 | 1.14E-28 | 22 | Nucleus | other |  |  | 6651 | 20658 | 304092 |
| MAGED2 | MAGE family member D2 | MAGED2 | 1.61 | 4.56E-30 | 6 | Plasma Membrane | other |  |  | 10916 | 80884 | 113947 |
| ASTN2 | astrotactin 2 | ASTN2 | 1.606 | 1.9E-76 | 6 | Cytoplasm | other |  |  | 23245 | 56079 | 100361323 |
| SPC25 | SPC25, NDC80 kinetochore complex component | SPC25 | 1.522 | 9.48E-66 | 18 | Cytoplasm | other |  |  | 57405 | 66442 | 295661 |
| OPHN1 | oligophrenin 1 | OPHN1 | 1.501 | 1.12E-79 | 11 | Cytoplasm | other |  |  | 4983 | 94190 | 312108 |
| DDX17 | DEAD-box helicase 17 | DDX17 | 1.465 | 2.84E-43 | 1 | Nucleus | enzyme | unspecified application |  | 10521 | 67040 |  |
| MAGED1 | MAGE family member D1 | MAGED1 | 1.463 | 8.88E-51 | 9 | Plasma Membrane | transcription regulator |  |  | 9500 | 94275 | 84469 |
| ODF2L | outer dense fiber of sperm tails 2 like | ODF2L | 1.401 | 3.6E-68 | 2 | Cytoplasm | other |  |  | 57489 | 52184 | 685425 |
| JUN | Jun proto-oncogene, AP-1 transcription factor subunit | JUN | 1.266 | 4.04E-37 | 12 | Nucleus | transcription regulator | prognosis |  | 3725 | 16476 | 24516 |
| ABCC9 | ATP binding cassette subfamily C member 9 | ABCC9 | 1.246 | 3.7E-103 | 9 | Plasma Membrane | ion channel |  |  | 10060 | 20928 | 25560 |
| NFIA | nuclear factor I A | NFIA | 1.232 | 1.16E-35 | 23 | Nucleus | transcription regulator |  |  | 4774 | 18027 | 25492 |
| LDHB | lactate dehydrogenase B | LDHB | 1.196 | 4.4E-34 | 9 | Cytoplasm | enzyme | unspecified application |  | 3945 | 16832 | 24534 |
| L2HGDH | L-2-hydroxyglutarate dehydrogenase | L2HGDH | 1.153 | 1.2E-74 | 19 | Cytoplasm | enzyme |  |  | 79944 | 217666 | 314196 |
| GLIPR1L2 | GLIPR1 like 2 | GLIPR1L2 | 1.073 | 3.62E-67 | 19 | Extracellular Space | other |  |  | 144321 | 67537 | 366890 |
| SHTN1 | shootin 1 | KIAA1598 | 1.044 | 1.39E-27 | 8 | Plasma Membrane | other |  |  | 57698 | 71653 | 292139 |
| GOSR1 | golgi SNAP receptor complex member 1 | GOSR1 | 1.036 | 5.09E-43 | 2 | Cytoplasm | transporter |  |  | 9527 | 53334 | 94189 |
| ST6GAL1 | ST6 beta-galactoside alpha-2,6-sialyltransferase 1 | ST6GAL1 | 1.016 | 2.31E-32 | 7 | Cytoplasm | enzyme |  |  | 6480 | 20440 | 25197 |
| LRTOMT | leucine rich transmembrane and O-methyltransferase domain containing | LRTOMT | 1.001 | 1.94E-29 | 16 | Plasma Membrane | enzyme |  |  | 220074 | 791260 | 308868 |

Cluster 7 pro-inflammatory

| © 2000-2018 QIAGEN. All rights reserved. |  |  |  |  |  |  |  |  |  |  |  |  |
| --- | --- | --- | --- | --- | --- | --- | --- | --- | --- | --- | --- | --- |
| Symbol | Entrez Gene Name | Gene Symbol - human (HUGO / HGNC / Entrez Gene)/Gene Symbol - mouse (Entrez Gene)/Gene Symbol - rat (Entrez Gene) | Expr Log Ratio | Expr False Discovery Rate (q-value) | Networks | Location | Type(s) | Biomarker Application(s) | Drug(s) | Entrez Gene ID for Human | Entrez Gene ID for Mouse | Entrez Gene ID for Rat |
| HNRNPH1 | heterogeneous nuclear ribonucleoprotein H1 | HNRNPH1 | 2.351 | 7.54E-68 | 1 | Nucleus | other | diagnosis |  | 3187 | 59013 |  |
| TRA2A | transformer 2 alpha homolog | TRA2A | 1.99 | 1.05E-60 | 3 | Nucleus | other |  |  | 29896 | 101214 | 500116 |
| GPM6A | glycoprotein M6A | GPM6A | 1.985 | 3.45E-24 | 20 | Plasma Membrane | ion channel |  |  | 2823 | 234267 | 306439 |
| CKB | creatine kinase B | CKB | 1.957 | 8.18E-45 | 4 | Cytoplasm | kinase | safety |  | 1152 | 12709 | 24264 |
| JAM3 | junctional adhesion molecule 3 | JAM3 | 1.831 | 9.13E-31 | 8 | Plasma Membrane | other |  |  | 83700 | 83964 | 315509 |
| PCDH11X | protocadherin 11 X-linked | PCDH11X | 1.808 | 2.51E-64 | 17 | Plasma Membrane | other |  |  | 27328 | 245578 | 317204 |
| SHISA9 | shisa family member 9 | SHISA9 | 1.748 | 3.15E-127 | 10 | Plasma Membrane | other |  |  | 729993 | 72555 | 100361134 |
| ARGLU1 | arginine and glutamate rich 1 | ARGLU1 | 1.722 | 1.88E-28 | 2 | Other | other |  |  | 55082 | 234023 | 290912 |
| JAG1 | jagged 1 | JAG1 | 1.703 | 1.7E-32 | 13 | Extracellular Space | growth factor | efficacy |  | 182 | 16449 | 29146 |
| SON | SON DNA binding protein | SON | 1.653 | 1.14E-28 | 22 | Nucleus | other |  |  | 6651 | 20658 | 304092 |
| SRSF6 | serine and arginine rich splicing factor 6 | SRSF6 | 1.569 | 1.32E-27 | 1 | Nucleus | other |  |  | 6431 | 67996 | 362264 |
| OPHN1 | oligophrenin 1 | OPHN1 | 1.501 | 1.12E-79 | 11 | Cytoplasm | other |  |  | 4983 | 94190 | 312108 |
| SOX9 | SRY-box 9 | SOX9 | 1.459 | 1.15E-21 | 23 | Nucleus | transcription regulator | disease progression,prognosis |  | 6662 | 20682 | 140586 |
| DCLK2 | doublecortin like kinase 2 | DCLK2 | 1.428 | 3.29E-52 | 4 | Cytoplasm | kinase |  |  | 166614 | 70762 | 310698 |
| TANC1 | tetratricopeptide repeat, ankyrin repeat and coiled-coil containing 1 | TANC1 | 1.392 | 3.04E-33 | 10 | Plasma Membrane | other |  |  | 85461 | 66860 | 311055 |
| CHD7 | chromodomain helicase DNA binding protein 7 | CHD7 | 1.342 | 9.68E-43 | 12 | Nucleus | enzyme |  |  | . | 320790 | 312974 |
| MAML2 | mastermind like transcriptional coactivator 2 | MAML2 | 1.325 | 1.58E-59 | 14 | Nucleus | transcription regulator |  |  | 84441 |  | 689844 |
| JUN | Jun proto-oncogene, AP-1 transcription factor subunit | JUN | 1.266 | 4.04E-37 | 12 | Nucleus | transcription regulator | prognosis |  | 3725 | 16476 | 24516 |
| ARHGAP12 | Rho GTPase activating protein 12 | ARHGAP12 | 1.257 | 4.14E-22 | 17 | Cytoplasm | other |  |  | 94134 | 75415 | 307016 |
| ABCC9 | ATP binding cassette subfamily C member 9 | ABCC9 | 1.246 | 3.7E-103 | 9 | Plasma Membrane | ion channel |  |  | 10060 | 20928 | 25560 |
| PTK2 | protein tyrosine kinase 2 | PTK2 | 1.186 | 9.27E-21 | 18 | Cytoplasm | kinase | diagnosis,disease progression,efficacy,prognosis | BI 853520, CT-707, TPX-0005 | 5747 | 14083 | 25614 |
| SOX2-OT | SOX2 overlapping transcript | SOX2-OT | 1.177 | 8.8E-53 |  | Other | other |  |  | 347689 |  |  |
| TOR1AIP2 | torsin 1A interacting protein 2 | TOR1AIP2 | 1.174 | 1.94E-36 | 11 | Cytoplasm | other |  |  | 163590 | 240832 | 304881 |
| MTPAP | mitochondrial poly(A) polymerase | MTPAP | 1.146 | 2.26E-46 | 6 | Cytoplasm | enzyme |  |  | 55149 | 67440 | 307050 |
| NOL4L | nucleolar protein 4 like | C20orf112 | 1.116 | 6.78E-29 | 17 | Other | other |  |  | 140688 | 329540 |  |
| TNRC6B | trinucleotide repeat containing 6B | TNRC6B | 1.109 | 6.4E-63 | 11 | Other | other |  |  | 23112 | 213988 | 192178 |
| ITM2C | integral membrane protein 2C | ITM2C | 1.081 | 3.25E-25 | 2 | Cytoplasm | other |  |  | 81618 | 64294 | 301575 |
| YES1 | YES proto-oncogene 1, Src family tyrosine kinase | YES1 | 1.065 | 5.07E-24 | 13 | Cytoplasm | kinase |  | dasatinib, JNJ-26483327, dasatinib/dexamethasone, dasatinib/nilotinib, cytarabine/dasatinib/dexamethasone/methotrexate | 7525 | 22612 | 24884 |
| SHTN1 | shootin 1 | KIAA1598 | 1.044 | 1.39E-27 | 8 | Plasma Membrane | other |  |  | 57698 | 71653 | 292139 |
