# Supplementary material for: Differential microglia and macrophage profiles in human IDH-mutant and -wild type glioblastoma
Source: Oncotarget. 2019 May 3;10(33):3129–43. doi: 10.18632/oncotarget.26863 (PMC6517100; doi:10.18632/oncotarget.26863)
Supplement: Supplementary file 4 [file oncotarget-10-3129-s004.doc]

| **Cluster 1** |  |  |  |  |
| --- | --- | --- | --- | --- |
| © 2000-2018 QIAGEN. All rights reserved. |  |  |  |  |
| Ingenuity Canonical Pathways | -log(B-H p-value) | Ratio | z-score | Molecules |
| EIF2 Signaling | 13.3 | 0.132 | 4.123 | RPL11,RAP2A,RPL22,RPLP1,RPS3A,RPS23,PPP1CB,RPL26,RPL7A,CCND1,RPS4X,RPL19,RPS3,PABPC1,RPL4,RPL23,EIF3E,RPL9,RPL15,EIF3F,RPS4Y1,HNRNPA1,RPL10,RPS27A,RPL5,RPL6,RPS25,EIF3L,RPSA,RPLP0 |
| Regulation of eIF4 and p70S6K Signaling | 2.46 | 0.0798 | #NUM! | PABPC1,RAP2A,EIF3F,RPS4Y1,RPS3A,RPS23,RPS27A,RPS25,EIF3E,RPS3,RPS4X,EIF3L,RPSA |
| Notch Signaling | 2 | 0.158 | 1 | DLL1,CNTN1,MFNG,MAML2,RBPJ,DLL3 |
| PTEN Signaling | 1.82 | 0.08 | -2.53 | PTK2,RAP2A,CSNK2A1,NTRK2,CASP9,NTRK3,PDGFRA,CSNK2B,CCND1,DDR1 |
| mTOR Signaling | 1.8 | 0.0628 | #NUM! | RAP2A,EIF3F,RPS4Y1,RPS3A,RPS23,RPS27A,RPS25,EIF3E,RPS3,RPS4X,EIF3L,RPSA,EIF4B |
| Thyroid Cancer Signaling | 0.987 | 0.109 | #NUM! | RAP2A,TCF4,NTRK2,NTRK3,CCND1 |
| Wnt/β-catenin Signaling | 0.987 | 0.0581 | 0.333 | SOX4,CSNK1E,CSNK2A1,TCF4,SOX6,RPS27A,SOX8,CSNK2B,SOX11,CCND1 |
| Amyloid Processing | 0.902 | 0.098 | #NUM! | CSNK1E,CSNK2A1,MAPT,CSNK2B,APP |
| Glutamate Degradation III (via 4-aminobutyrate) | 0.902 | 0.4 | #NUM! | ABAT,GAD1 |
| Semaphorin Signaling in Neurons | 0.902 | 0.0943 | #NUM! | PTK2,DPYSL2,CRMP1,DPYSL3,DPYSL4 |
| Cell Cycle: G1/S Checkpoint Regulation | 0.521 | 0.0746 | 0 | RPL11,CCND2,HDAC2,RPL5,CCND1 |
| RhoA Signaling | 0.521 | 0.0565 | 1.633 | PTK2,SEPT8,RTKN,PFN2,PPP1CB,WASF1,SEPT2 |
| NF-κB Signaling | 0.521 | 0.0481 | 2.333 | RAP2A,CSNK2A1,NTRK2,HDAC2,NTRK3,BMP2,PDGFRA,CSNK2B,DDR1 |
| Ephrin B Signaling | 0.466 | 0.0685 | 2 | PTK2,EPHB1,RACK1,GNAI1,HNRNPK |
| Chemokine Signaling | 0.414 | 0.0649 | 1.342 | PTK2,RAP2A,CALM1 (includes others),GNAI1,PPP1CB |
| Axonal Guidance Signaling | 0.414 | 0.035 | #NUM! | DPYSL2,RAP2A,SEMA5A,CRKL,BMP2,ARHGEF7,RACK1,GNAI1,ADAM22,PTK2,MICAL1,EPHB1,NTRK2,TUBA1A,NTRK3,PFN2 |
| Acyl Carrier Protein Metabolism | 0.414 | 1 | #NUM! | AASDHPPT |
| Clathrin-mediated Endocytosis Signaling | 0.414 | 0.0435 | #NUM! | CSNK2A1,FGF12,RPS27A,RAB11A,HIP1,CSNK2B,CTTN,HIP1R,APOD |
| Cleavage and Polyadenylation of Pre-mRNA | 0.414 | 0.167 | #NUM! | CPSF6,NUDT21 |
| IGF-1 Signaling | 0.414 | 0.0536 | 1.342 | PTK2,RAP2A,CSNK2A1,CASP9,YWHAE,CSNK2B |
| Glutamate Receptor Signaling | 0.392 | 0.0702 | #NUM! | CALM1 (includes others),GRIK2,GRIA4,GRIA3 |
| DNA Methylation and Transcriptional Repression Signaling | 0.361 | 0.0882 | #NUM! | H3F3A/H3F3B,HDAC2,RBBP4 |
| Telomere Extension by Telomerase | 0.322 | 0.133 | #NUM! | HNRNPA1,HNRNPA2B1 |
| Parkinson's Signaling | 0.322 | 0.125 | #NUM! | CASP9,SNCAIP |
| β-alanine Degradation I | 0.322 | 0.5 | #NUM! | ABAT |
| Glutamate Dependent Acid Resistance | 0.322 | 0.5 | #NUM! | GAD1 |
| PDGF Signaling | 0.322 | 0.0521 | 2.236 | RAP2A,CSNK2A1,CRKL,PDGFRA,CSNK2B |
| Actin Cytoskeleton Signaling | 0.322 | 0.0386 | 1.89 | PTK2,RAP2A,CYFIP2,CRKL,ARHGEF7,FGF12,PFN2,PPP1CB,WASF1 |
| Gap Junction Signaling | 0.322 | 0.0398 | #NUM! | CSNK1E,RAP2A,DBN1,TUBA1A,GNAI1,GRIK2,GRIA4,GRIA3 |
| STAT3 Pathway | 0.322 | 0.0485 | 2.236 | RAP2A,NTRK2,NTRK3,PDGFRA,DDR1 |
| GADD45 Signaling | 0.322 | 0.105 | #NUM! | CCND2,CCND1 |
| Androgen Signaling | 0.322 | 0.0438 | #NUM! | CALM1 (includes others),POLR2F,RACK1,GNAI1,CCND1,NCOA4 |
| Breast Cancer Regulation by Stathmin1 | 0.322 | 0.0379 | #NUM! | STMN1,RAP2A,CALM1 (includes others),TUBA1A,ARHGEF7,RACK1,GNAI1,PPP1CB |
| Diphthamide Biosynthesis | 0.322 | 0.333 | #NUM! | EEF2 |
| 5-aminoimidazole Ribonucleotide Biosynthesis I | 0.322 | 0.333 | #NUM! | GART |
| Oxidized GTP and dGTP Detoxification | 0.322 | 0.333 | #NUM! | DDX6 |
| 4-aminobutyrate Degradation I | 0.322 | 0.333 | #NUM! | ABAT |
| Cardiomyocyte Differentiation via BMP Receptors | 0.322 | 0.1 | #NUM! | BMP2,NPPA |
| Granzyme A Signaling | 0.322 | 0.1 | #NUM! | SET,H1F0 |
| Aryl Hydrocarbon Receptor Signaling | 0.322 | 0.0426 | 2 | GSTT2/GSTT2B,CCND2,NFIA,GSTA4,NFIB,CCND1 |
| PAK Signaling | 0.322 | 0.0467 | 2.236 | PTK2,RAP2A,ARHGEF7,PDGFRA,DSCAM |
| Agrin Interactions at Neuromuscular Junction | 0.322 | 0.0533 | 2 | PTK2,RAP2A,ARHGEF7,CTTN |
| Signaling by Rho Family GTPases | 0.322 | 0.0357 | 1.89 | STMN1,PTK2,SEPT8,ARHGEF7,CDH10,RACK1,GNAI1,WASF1,SEPT2 |
| Endoplasmic Reticulum Stress Pathway | 0.318 | 0.0952 | #NUM! | CASP9,TAOK3 |
| CREB Signaling in Neurons | 0.309 | 0.0367 | 2.449 | RAP2A,CALM1 (includes others),POLR2F,RACK1,GNAI1,GRIK2,GRIA4,GRIA3 |
| Integrin Signaling | 0.309 | 0.0365 | 2.449 | PTK2,RAP2A,TSPAN7,CRKL,ARHGEF7,PFN2,PPP1CB,CTTN |
| Epithelial Adherens Junction Signaling | 0.283 | 0.04 | #NUM! | RAP2A,EPN2,TCF4,DLL1,TUBA1A,WASF1 |
| Uracil Degradation II (Reductive) | 0.283 | 0.25 | #NUM! | DPYSL2 |
| Thymine Degradation | 0.283 | 0.25 | #NUM! | DPYSL2 |
| TCA Cycle II (Eukaryotic) | 0.272 | 0.0833 | #NUM! | SUCLG1,DHTKD1 |
| HIPPO signaling | 0.256 | 0.046 | #NUM! | CSNK1E,YWHAE,PPP1CB,CRB1 |
| Creatine-phosphate Biosynthesis | 0.256 | 0.2 | #NUM! | CKB |
| 2-ketoglutarate Dehydrogenase Complex | 0.256 | 0.2 | #NUM! | DHTKD1 |
| Tetrahydrofolate Salvage from 5,10-methenyltetrahydrofolate | 0.256 | 0.2 | #NUM! | GART |
| Lysine Degradation II | 0.256 | 0.2 | #NUM! | AASDHPPT |
| Lysine Degradation V | 0.256 | 0.2 | #NUM! | AASDHPPT |
| Trans, trans-farnesyl Diphosphate Biosynthesis | 0.256 | 0.2 | #NUM! | IDI1 |
| Synaptic Long Term Potentiation | 0.229 | 0.0397 | 1.342 | RAP2A,CALM1 (includes others),PPP1CB,GRIA4,GRIA3 |
| Reelin Signaling in Neurons | 0.225 | 0.0435 | #NUM! | PAFAH1B2,MAPT,CRKL,APP |
| Nur77 Signaling in T Lymphocytes | 0.225 | 0.0508 | #NUM! | CALM1 (includes others),CASP9,HDAC2 |
| α-Adrenergic Signaling | 0.225 | 0.043 | #NUM! | RAP2A,CALM1 (includes others),RACK1,GNAI1 |
| PEDF Signaling | 0.225 | 0.043 | 2 | RAP2A,TCF4,ZEB1,TCF12 |
| GABA Receptor Signaling | 0.208 | 0.0421 | #NUM! | ABAT,GAD1,RPS27A,GABBR1 |
| Huntington's Disease Signaling | 0.208 | 0.032 | #NUM! | POLR2F,CASP9,HDAC2,ATP5F1A,RACK1,HSPA1A/HSPA1B,RPS27A,HIP1 |
| Mitochondrial Dysfunction | 0.208 | 0.0351 | #NUM! | NDUFA5,CASP9,ATP5F1A,COX7A2L,UQCRB,APP |
| Glutathione-mediated Detoxification | 0.208 | 0.0645 | #NUM! | GSTT2/GSTT2B,GSTA4 |
| Adipogenesis pathway | 0.208 | 0.0373 | #NUM! | SIRT2,HDAC2,BMP2,TBL1XR1,RBBP4 |
| Regulation of Cellular Mechanics by Calpain Protease | 0.206 | 0.0476 | #NUM! | PTK2,RAP2A,CCND1 |
| CCR3 Signaling in Eosinophils | 0.202 | 0.0368 | #NUM! | RAP2A,CALM1 (includes others),RACK1,GNAI1,PPP1CB |
| p70S6K Signaling | 0.194 | 0.0362 | 2 | RAP2A,YWHAE,EEF2,MAPT,GNAI1 |
| RhoGDI Signaling | 0.194 | 0.0339 | -1 | ARHGEF7,CDH10,RACK1,GDI2,GNAI1,WASF1 |
| Ephrin Receptor Signaling | 0.184 | 0.0335 | 2.236 | PTK2,RAP2A,EPHB1,CRKL,RACK1,GNAI1 |
| Circadian Rhythm Signaling | 0.182 | 0.0588 | #NUM! | CSNK1E,VIPR2 |
| Human Embryonic Stem Cell Pluripotency | 0.17 | 0.035 | #NUM! | TCF4,NTRK2,NTRK3,BMP2,PDGFRA |
| CDK5 Signaling | 0.17 | 0.0381 | 1 | RAP2A,NTRK2,MAPT,PPP1CB |
| Molecular Mechanisms of Cancer | 0.17 | 0.0279 | #NUM! | PTK2,RAP2A,TCF4,CASP9,CCND2,BMP2,ARHGEF7,GNAI1,RBPJ,HIPK2,CCND1 |
| Endometrial Cancer Signaling | 0.168 | 0.0429 | #NUM! | RAP2A,CASP9,CCND1 |
| IL-2 Signaling | 0.168 | 0.0429 | #NUM! | RAP2A,CSNK2A1,CSNK2B |
| Protein Kinase A Signaling | 0.157 | 0.0274 | 0.333 | PTK2,CALM1 (includes others),TCF4,H3F3A/H3F3B,YWHAE,RACK1,PTPRS,GNAI1,PPP1CB,PTPRZ1,H1F0 |
| Oxidative Phosphorylation | 0.157 | 0.0367 | 2 | NDUFA5,ATP5F1A,COX7A2L,UQCRB |
| Hereditary Breast Cancer Signaling | 0.153 | 0.0333 | #NUM! | RAP2A,POLR2F,HDAC2,RPS27A,CCND1 |
| Amyotrophic Lateral Sclerosis Signaling | 0.153 | 0.036 | 2 | CASP9,GRIK2,GRIA4,GRIA3 |
| p53 Signaling | 0.153 | 0.036 | #NUM! | CCND2,HIPK2,ST13,CCND1 |
| Chronic Myeloid Leukemia Signaling | 0.149 | 0.0357 | #NUM! | RAP2A,HDAC2,CRKL,CCND1 |
| Myc Mediated Apoptosis Signaling | 0.134 | 0.0395 | #NUM! | RAP2A,CASP9,YWHAE |
| Role of Wnt/GSK-3β Signaling in the Pathogenesis of Influenza | 0.134 | 0.039 | #NUM! | CSNK1E,TCF4,NCOA4 |
| Purine Nucleotides De Novo Biosynthesis II | 0.134 | 0.0909 | #NUM! | GART |
| Role of PI3K/AKT Signaling in the Pathogenesis of Influenza | 0.134 | 0.038 | #NUM! | CASP9,CRKL,GNAI1 |
| GM-CSF Signaling | 0.134 | 0.038 | #NUM! | RAP2A,RACK1,CCND1 |
| Phospholipase C Signaling | 0.134 | 0.0287 | 1.633 | RAP2A,MARCKS,CALM1 (includes others),HDAC2,ARHGEF7,RACK1,PPP1CB |
| HGF Signaling | 0.134 | 0.0336 | 2 | PTK2,RAP2A,CRKL,CCND1 |
| IL-8 Signaling | 0.134 | 0.0296 | 2.236 | PTK2,RAP2A,CCND2,RACK1,GNAI1,CCND1 |
| Glioma Signaling | 0.134 | 0.0333 | #NUM! | RAP2A,CALM1 (includes others),PDGFRA,CCND1 |
| Assembly of RNA Polymerase I Complex | 0.134 | 0.0833 | #NUM! | POLR2F |
| Pregnenolone Biosynthesis | 0.134 | 0.0833 | #NUM! | MICAL1 |
| Cyclins and Cell Cycle Regulation | 0.134 | 0.037 | #NUM! | CCND2,HDAC2,CCND1 |
| Sirtuin Signaling Pathway | 0.134 | 0.0274 | 1.342 | NDUFA5,POLR2F,SIRT2,H3F3A/H3F3B,TUBA1A,ATP5F1A,H1F0,APP |
| Rac Signaling | 0.134 | 0.0325 | 2 | PTK2,RAP2A,CYFIP2,WASF1 |
| Gαi Signaling | 0.134 | 0.0325 | #NUM! | RAP2A,RACK1,GNAI1,GABBR1 |
| Non-Small Cell Lung Cancer Signaling | 0.134 | 0.0361 | #NUM! | RAP2A,CASP9,CCND1 |
| Neurotrophin/TRK Signaling | 0.134 | 0.0361 | #NUM! | RAP2A,NTRK2,NTRK3 |
| Oleate Biosynthesis II (Animals) | 0.134 | 0.0769 | #NUM! | SCD5 |
| Mevalonate Pathway I | 0.134 | 0.0769 | #NUM! | IDI1 |
| Angiopoietin Signaling | 0.134 | 0.0357 | #NUM! | PTK2,RAP2A,CASP9 |
| Sphingosine-1-phosphate Signaling | 0.134 | 0.032 | 1 | PTK2,CASP9,PDGFRA,GNAI1 |
| Small Cell Lung Cancer Signaling | 0.134 | 0.0353 | #NUM! | PTK2,CASP9,CCND1 |
| Leukocyte Extravasation Signaling | 0.134 | 0.0284 | 2 | PTK2,CRKL,JAM2,GNAI1,THY1,CTTN |
| Sperm Motility | 0.13 | 0.0315 | 2 | PTK2,PAFAH1B2,CALM1 (includes others),NPPA |
| fMLP Signaling in Neutrophils | 0.119 | 0.031 | #NUM! | RAP2A,CALM1 (includes others),RACK1,GNAI1 |
| Cell Cycle: G2/M DNA Damage Checkpoint Regulation | 0.118 | 0.04 | #NUM! | YWHAE,HIPK2 |
| Histidine Degradation VI | 0.118 | 0.0667 | #NUM! | MICAL1 |
| Regulation of Actin-based Motility by Rho | 0.118 | 0.0333 | #NUM! | PFN2,PPP1CB,WASF1 |
| Protein Ubiquitination Pathway | 0.118 | 0.0264 | #NUM! | USP3,PSMB5,USP22,HSPA1A/HSPA1B,RPS27A,UBE2V2,UBE2E2 |
| Estrogen Receptor Signaling | 0.118 | 0.0299 | #NUM! | RAP2A,POLR2F,H3F3A/H3F3B,GTF2F2 |
| Factors Promoting Cardiogenesis in Vertebrates | 0.118 | 0.0326 | #NUM! | TCF4,BMP2,NPPA |
| Docosahexaenoic Acid (DHA) Signaling | 0.118 | 0.0385 | #NUM! | CASP9,APP |
| Granzyme B Signaling | 0.118 | 0.0625 | #NUM! | CASP9 |
| Synaptic Long Term Depression | 0.118 | 0.0278 | 2.236 | PAFAH1B2,RAP2A,GNAI1,GRIA4,GRIA3 |
| Neuregulin Signaling | 0.118 | 0.0319 | #NUM! | RAP2A,CRKL,TMEFF2 |
| Bladder Cancer Signaling | 0.118 | 0.0319 | #NUM! | RAP2A,FGF12,CCND1 |
| HER-2 Signaling in Breast Cancer | 0.118 | 0.0319 | #NUM! | RAP2A,CASP9,CCND1 |
| 14-3-3-mediated Signaling | 0.118 | 0.0292 | #NUM! | RAP2A,TUBA1A,YWHAE,MAPT |
| Iron homeostasis signaling pathway | 0.118 | 0.0292 | #NUM! | ATP6V0E2,BMP2,PDGFRA,ATP6V1G2 |
| CCR5 Signaling in Macrophages | 0.118 | 0.0316 | #NUM! | CALM1 (includes others),RACK1,GNAI1 |
| Transcriptional Regulatory Network in Embryonic Stem Cells | 0.118 | 0.037 | #NUM! | SET,H3F3A/H3F3B |
| Superpathway of Geranylgeranyldiphosphate Biosynthesis I (via Mevalonate) | 0.118 | 0.0588 | #NUM! | IDI1 |
| Sumoylation Pathway | 0.114 | 0.0312 | #NUM! | HDAC2,GDI2,ZEB1 |
| Valine Degradation I | 0.109 | 0.0556 | #NUM! | ABAT |
| TR/RXR Activation | 0.109 | 0.0306 | #NUM! | THRA,TBL1XR1,NCOA4 |
| ATM Signaling | 0.109 | 0.0306 | #NUM! | ZEB1,CBX5,CBX3 |
| Acute Myeloid Leukemia Signaling | 0.104 | 0.0303 | #NUM! | RAP2A,TCF4,CCND1 |
| Ubiquinol-10 Biosynthesis (Eukaryotic) | 0.0982 | 0.0526 | #NUM! | MICAL1 |
| Role of NFAT in Regulation of the Immune Response | 0.0901 | 0.026 | 2 | CSNK1E,RAP2A,CALM1 (includes others),RACK1,GNAI1 |
| Inflammasome pathway | 0.0901 | 0.05 | #NUM! | P2RX7 |
| Prostate Cancer Signaling | 0.0901 | 0.0291 | #NUM! | RAP2A,CASP9,CCND1 |
| Phagosome Maturation | 0.0901 | 0.027 | #NUM! | ATP6V0E2,TUBA1A,ATP6V1G2,PRDX2 |
| Regulation of the Epithelial-Mesenchymal Transition Pathway | 0.0901 | 0.0256 | #NUM! | RAP2A,TCF4,FGF12,RBPJ,ZEB1 |
| Induction of Apoptosis by HIV1 | 0.0901 | 0.0328 | #NUM! | CASP9,SLC25A3 |
| Melanoma Signaling | 0.0901 | 0.0328 | #NUM! | RAP2A,CCND1 |
| FAK Signaling | 0.09 | 0.0286 | #NUM! | PTK2,RAP2A,ARHGEF7 |
| Actin Nucleation by ARP-WASP Complex | 0.0871 | 0.0323 | #NUM! | RAP2A,WASF1 |
| Cholecystokinin/Gastrin-mediated Signaling | 0.0826 | 0.028 | #NUM! | PTK2,RAP2A,CCK |
| Polyamine Regulation in Colon Cancer | 0.0813 | 0.0455 | #NUM! | TCF4 |
| VEGF Signaling | 0.0756 | 0.0275 | #NUM! | PTK2,RAP2A,YWHAE |
| SAPK/JNK Signaling | 0.0751 | 0.0273 | #NUM! | RAP2A,CRKL,HNRNPK |
| ERK/MAPK Signaling | 0.0751 | 0.0245 | 1.342 | PTK2,RAP2A,H3F3A/H3F3B,CRKL,PPP1CB |
| Calcium-induced T Lymphocyte Apoptosis | 0.0751 | 0.0303 | #NUM! | CALM1 (includes others),HDAC2 |
| Calcium Signaling | 0.0751 | 0.0243 | 2 | RAP2A,CALM1 (includes others),HDAC2,GRIA4,GRIA3 |
| Tumoricidal Function of Hepatic Natural Killer Cells | 0.0751 | 0.0417 | #NUM! | CASP9 |
| Glutathione Redox Reactions I | 0.0751 | 0.0417 | #NUM! | GSTT2/GSTT2B |
| CDP-diacylglycerol Biosynthesis I | 0.0751 | 0.0417 | #NUM! | AGPAT1 |
| EGF Signaling | 0.0722 | 0.0294 | #NUM! | CSNK2A1,CSNK2B |
| Neuropathic Pain Signaling In Dorsal Horn Neurons | 0.0722 | 0.0261 | #NUM! | NTRK2,GRIA4,GRIA3 |
| Thrombin Signaling | 0.0722 | 0.0238 | #NUM! | PTK2,RAP2A,RACK1,GNAI1,PPP1CB |
| Dopamine-DARPP32 Feedback in cAMP Signaling | 0.0722 | 0.0244 | 0 | CSNK1E,CALM1 (includes others),GNAI1,PPP1CB |
| Osteoarthritis Pathway | 0.0722 | 0.0236 | 2 | TCF4,CASP9,BMP2,RBPJ,P2RX7 |
| Antiproliferative Role of TOB in T Cell Signaling | 0.0722 | 0.0385 | #NUM! | PABPC1 |
| Estrogen-mediated S-phase Entry | 0.0722 | 0.0385 | #NUM! | CCND1 |
| Phosphatidylglycerol Biosynthesis II (Non-plastidic) | 0.0722 | 0.0385 | #NUM! | AGPAT1 |
| Paxillin Signaling | 0.0722 | 0.0252 | #NUM! | PTK2,RAP2A,ARHGEF7 |
| Melatonin Signaling | 0.0722 | 0.0278 | #NUM! | CALM1 (includes others),GNAI1 |
| Basal Cell Carcinoma Signaling | 0.0722 | 0.0278 | #NUM! | TCF4,BMP2 |
| ERK5 Signaling | 0.0722 | 0.0278 | #NUM! | RAP2A,YWHAE |
| G Beta Gamma Signaling | 0.0722 | 0.025 | #NUM! | RAP2A,RACK1,GNAI1 |
| Neuroprotective Role of THOP1 in Alzheimer's Disease | 0.0722 | 0.025 | #NUM! | YWHAE,MAPT,APP |
| GPCR-Mediated Integration of Enteroendocrine Signaling Exemplified by an L Cell | 0.0722 | 0.0274 | #NUM! | GNAI1,CCK |
| IL-15 Production | 0.0722 | 0.0357 | #NUM! | PTK2 |
| Superpathway of Cholesterol Biosynthesis | 0.0722 | 0.0357 | #NUM! | IDI1 |
| CXCR4 Signaling | 0.0722 | 0.0234 | #NUM! | PTK2,RAP2A,RACK1,GNAI1 |
| GNRH Signaling | 0.0722 | 0.0234 | #NUM! | PTK2,RAP2A,CALM1 (includes others),GNAI1 |
| ErbB2-ErbB3 Signaling | 0.0722 | 0.0267 | #NUM! | RAP2A,CCND1 |
| Hypoxia Signaling in the Cardiovascular System | 0.0722 | 0.0267 | #NUM! | UBE2V2,UBE2E2 |
| Glioma Invasiveness Signaling | 0.0681 | 0.0263 | #NUM! | PTK2,RAP2A |
| Role of NFAT in Cardiac Hypertrophy | 0.0602 | 0.0222 | #NUM! | RAP2A,CALM1 (includes others),HDAC2,RACK1,GNAI1 |
| Role of MAPK Signaling in the Pathogenesis of Influenza | 0.0602 | 0.0256 | #NUM! | PAFAH1B2,RAP2A |
| Natural Killer Cell Signaling | 0.0579 | 0.0234 | #NUM! | RAP2A,KLRC2,KLRC3 |
| Germ Cell-Sertoli Cell Junction Signaling | 0.0579 | 0.0223 | #NUM! | PTK2,RAP2A,EPN2,TUBA1A |
| Cytotoxic T Lymphocyte-mediated Apoptosis of Target Cells | 0.0579 | 0.0312 | #NUM! | CASP9 |
| G Protein Signaling Mediated by Tubby | 0.0579 | 0.0312 | #NUM! | RACK1 |
| Antiproliferative Role of Somatostatin Receptor 2 | 0.0579 | 0.0247 | #NUM! | RAP2A,RACK1 |
| Cellular Effects of Sildenafil (Viagra) | 0.0579 | 0.0229 | #NUM! | CALM1 (includes others),PPP1CB,NPPA |
| PI3K/AKT Signaling | 0.0579 | 0.0229 | #NUM! | RAP2A,YWHAE,CCND1 |
| IL-15 Signaling | 0.0579 | 0.0244 | #NUM! | PTK2,RAP2A |
| BMP signaling pathway | 0.0579 | 0.0244 | #NUM! | RAP2A,BMP2 |
| Sertoli Cell-Sertoli Cell Junction Signaling | 0.0555 | 0.0217 | #NUM! | RAP2A,EPN2,TUBA1A,JAM2 |
| IL-6 Signaling | 0.0555 | 0.0224 | #NUM! | RAP2A,CSNK2A1,CSNK2B |
| Retinoate Biosynthesis I | 0.0555 | 0.0294 | #NUM! | BMP2 |
| TWEAK Signaling | 0.052 | 0.0286 | #NUM! | CASP9 |
| Nucleotide Excision Repair Pathway | 0.052 | 0.0286 | #NUM! | POLR2F |
| Estrogen-Dependent Breast Cancer Signaling | 0.052 | 0.0233 | #NUM! | RAP2A,CCND1 |
| Regulation of IL-2 Expression in Activated and Anergic T Lymphocytes | 0.052 | 0.0233 | #NUM! | RAP2A,CALM1 (includes others) |
| RAR Activation | 0.0495 | 0.0211 | #NUM! | CSNK2A1,BMP2,CSNK2B,RPL7A |
| Renal Cell Carcinoma Signaling | 0.0495 | 0.0227 | #NUM! | RAP2A,RPS27A |
| P2Y Purigenic Receptor Signaling Pathway | 0.0495 | 0.0214 | #NUM! | RAP2A,RACK1,GNAI1 |
| Cell Cycle Regulation by BTG Family Proteins | 0.0495 | 0.027 | #NUM! | CCND1 |
| IL-3 Signaling | 0.0495 | 0.0225 | #NUM! | RAP2A,CRKL |
| Gα12/13 Signaling | 0.0495 | 0.0213 | #NUM! | PTK2,RAP2A,CDH10 |
| Antigen Presentation Pathway | 0.0495 | 0.0263 | #NUM! | PSMB5 |
| FGF Signaling | 0.0495 | 0.022 | #NUM! | CRKL,FGF12 |
| IL-7 Signaling Pathway | 0.0495 | 0.022 | #NUM! | PTK2,CCND1 |
| Endothelin-1 Signaling | 0.0495 | 0.0204 | 2 | PAFAH1B2,RAP2A,CASP9,GNAI1 |
| tRNA Charging | 0.0495 | 0.0256 | #NUM! | EPRS |
| IL-1 Signaling | 0.0495 | 0.0217 | #NUM! | RACK1,GNAI1 |
| TGF-β Signaling | 0.0495 | 0.0215 | #NUM! | RAP2A,BMP2 |
| Oncostatin M Signaling | 0.0495 | 0.025 | #NUM! | RAP2A |
| Insulin Receptor Signaling | 0.0495 | 0.0204 | #NUM! | RAP2A,CRKL,PPP1CB |
| Adrenomedullin signaling pathway | 0.0495 | 0.02 | 2 | PTK2,RAP2A,CALM1 (includes others),CALCRL |
| Role of PKR in Interferon Induction and Antiviral Response | 0.0495 | 0.0244 | #NUM! | CASP9 |
| nNOS Signaling in Skeletal Muscle Cells | 0.0495 | 0.0244 | #NUM! | CALM1 (includes others) |
| Colorectal Cancer Metastasis Signaling | 0.0495 | 0.0197 | 1 | RAP2A,TCF4,CASP9,RACK1,CCND1 |
| Apoptosis Signaling | 0.0475 | 0.0208 | #NUM! | RAP2A,CASP9 |
| Ovarian Cancer Signaling | 0.0475 | 0.02 | #NUM! | RAP2A,TCF4,CCND1 |
| iNOS Signaling | 0.0343 | 0.0222 | #NUM! | CALM1 (includes others) |
| Pyrimidine Ribonucleotides Interconversion | 0.0343 | 0.0222 | #NUM! | HNRNPA1 |
| PPAR Signaling | 0.0343 | 0.0198 | #NUM! | RAP2A,PDGFRA |
| Dermatan Sulfate Biosynthesis (Late Stages) | 0.0336 | 0.0217 | #NUM! | DSEL |
| Triacylglycerol Biosynthesis | 0.0336 | 0.0217 | #NUM! | AGPAT1 |
| nNOS Signaling in Neurons | 0.0319 | 0.0213 | #NUM! | CALM1 (includes others) |
| Pyrimidine Ribonucleotides De Novo Biosynthesis | 0.0319 | 0.0213 | #NUM! | HNRNPA1 |
| Chondroitin Sulfate Biosynthesis (Late Stages) | 0.025 | 0.0204 | #NUM! | CHST9 |
| UVC-Induced MAPK Signaling | 0.025 | 0.0204 | #NUM! | RAP2A |
| TNFR1 Signaling | 0.0237 | 0.02 | #NUM! | CASP9 |
| Assembly of RNA Polymerase II Complex | 0.0237 | 0.02 | #NUM! | POLR2F |
| Glucocorticoid Receptor Signaling | 0 | 0.0145 | #NUM! | RAP2A,POLR2F,GTF2F2,HSPA1A/HSPA1B,NPPA |
| Fc Epsilon RI Signaling | 0 | 0.008 | #NUM! | RAP2A |
| NRF2-mediated Oxidative Stress Response | 0 | 0.0151 | #NUM! | RAP2A,GSTT2/GSTT2B,GSTA4 |
| PPARα/RXRα Activation | 0 | 0.00538 | #NUM! | RAP2A |
| LPS/IL-1 Mediated Inhibition of RXR Function | 0 | 0.00901 | #NUM! | GSTT2/GSTT2B,GSTA4 |
| Acute Phase Response Signaling | 0 | 0.017 | #NUM! | RAP2A,TCF4,HNRNPK |
| LXR/RXR Activation | 0 | 0.00826 | #NUM! | APOD |
| Hepatic Fibrosis / Hepatic Stellate Cell Activation | 0 | 0.00535 | #NUM! | PDGFRA |
| VDR/RXR Activation | 0 | 0.0128 | #NUM! | CSNK2A1 |
| FXR/RXR Activation | 0 | 0.00794 | #NUM! | APOD |
| Ceramide Signaling | 0 | 0.0101 | #NUM! | RAP2A |
| Tight Junction Signaling | 0 | 0.018 | #NUM! | CPSF6,NUDT21,JAM2 |
| Erythropoietin Signaling | 0 | 0.0115 | #NUM! | RAP2A |
| Caveolar-mediated Endocytosis Signaling | 0 | 0.0141 | #NUM! | COPG2 |
| Fcγ Receptor-mediated Phagocytosis in Macrophages and Monocytes | 0 | 0.0108 | #NUM! | RAB11A |
| IL-12 Signaling and Production in Macrophages | 0 | 0.00685 | #NUM! | APOD |
| FcγRIIB Signaling in B Lymphocytes | 0 | 0.0118 | #NUM! | RAP2A |
| LPS-stimulated MAPK Signaling | 0 | 0.0108 | #NUM! | RAP2A |
| NF-κB Activation by Viruses | 0 | 0.0108 | #NUM! | RAP2A |
| CD27 Signaling in Lymphocytes | 0 | 0.0189 | #NUM! | CASP9 |
| Lymphotoxin β Receptor Signaling | 0 | 0.0149 | #NUM! | CASP9 |
| IL-17 Signaling | 0 | 0.011 | #NUM! | RAP2A |
| Thrombopoietin Signaling | 0 | 0.0141 | #NUM! | RAP2A |
| CD28 Signaling in T Helper Cells | 0 | 0.00758 | #NUM! | CALM1 (includes others) |
| Virus Entry via Endocytic Pathways | 0 | 0.00862 | #NUM! | RAP2A |
| HIF1α Signaling | 0 | 0.00806 | #NUM! | RAP2A |
| Relaxin Signaling | 0 | 0.0127 | #NUM! | RACK1,GNAI1 |
| CNTF Signaling | 0 | 0.0143 | #NUM! | RAP2A |
| Renin-Angiotensin Signaling | 0 | 0.0156 | #NUM! | PTK2,RAP2A |
| Cardiac Hypertrophy Signaling | 0 | 0.0166 | #NUM! | RAP2A,CALM1 (includes others),RACK1,GNAI1 |
| iCOS-iCOSL Signaling in T Helper Cells | 0 | 0.00813 | #NUM! | CALM1 (includes others) |
| Corticotropin Releasing Hormone Signaling | 0 | 0.0144 | #NUM! | CALM1 (includes others),GNAI1 |
| Mitotic Roles of Polo-Like Kinase | 0 | 0.0152 | #NUM! | RAD21 |
| HMGB1 Signaling | 0 | 0.00719 | #NUM! | RAP2A |
| FLT3 Signaling in Hematopoietic Progenitor Cells | 0 | 0.0108 | #NUM! | RAP2A |
| Melanocyte Development and Pigmentation Signaling | 0 | 0.00962 | #NUM! | RAP2A |
| Aldosterone Signaling in Epithelial Cells | 0 | 0.00595 | #NUM! | HSPA1A/HSPA1B |
| Role of NANOG in Mammalian Embryonic Stem Cell Pluripotency | 0 | 0.0156 | #NUM! | RAP2A,BMP2 |
| Prolactin Signaling | 0 | 0.0112 | #NUM! | RAP2A |
| Type I Diabetes Mellitus Signaling | 0 | 0.018 | #NUM! | CASP9,GAD1 |
| Production of Nitric Oxide and Reactive Oxygen Species in Macrophages | 0 | 0.0103 | #NUM! | PPP1CB,APOD |
| Pancreatic Adenocarcinoma Signaling | 0 | 0.0167 | #NUM! | CASP9,CCND1 |
| Systemic Lupus Erythematosus Signaling | 0 | 0.0172 | #NUM! | RAP2A,SNRPN,HNRNPA2B1,SNRPB2 |
| Cdc42 Signaling | 0 | 0.00599 | #NUM! | PPP1CB |
| ILK Signaling | 0 | 0.0152 | #NUM! | PTK2,BMP2,CCND1 |
| Retinoic acid Mediated Apoptosis Signaling | 0 | 0.0161 | #NUM! | CASP9 |
| AMPK Signaling | 0 | 0.0185 | #NUM! | EEF2,RAB2A,RAB11A,CCND1 |
| Role of Osteoblasts, Osteoclasts and Chondrocytes in Rheumatoid Arthritis | 0 | 0.0172 | #NUM! | CALM1 (includes others),TCF4,CASP9,BMP2 |
| Atherosclerosis Signaling | 0 | 0.0157 | #NUM! | PAFAH1B2,APOD |
| Role of Macrophages, Fibroblasts and Endothelial Cells in Rheumatoid Arthritis | 0 | 0.0125 | #NUM! | RAP2A,CALM1 (includes others),TCF4,CCND1 |
| RANK Signaling in Osteoclasts | 0 | 0.0098 | #NUM! | CALM1 (includes others) |
| Glioblastoma Multiforme Signaling | 0 | 0.0179 | #NUM! | RAP2A,PDGFRA,CCND1 |
| PKCθ Signaling in T Lymphocytes | 0 | 0.00606 | #NUM! | RAP2A |
| PI3K Signaling in B Lymphocytes | 0 | 0.0147 | #NUM! | RAP2A,CALM1 (includes others) |
| Cell Cycle Control of Chromosomal Replication | 0 | 0.0179 | #NUM! | ORC4 |
| Role of Tissue Factor in Cancer | 0 | 0.00769 | #NUM! | RAP2A |
| Role of JAK1 and JAK3 in γc Cytokine Signaling | 0 | 0.013 | #NUM! | RAP2A |
| NGF Signaling | 0 | 0.008 | #NUM! | RAP2A |
| Telomerase Signaling | 0 | 0.0171 | #NUM! | RAP2A,HDAC2 |
| Mouse Embryonic Stem Cell Pluripotency | 0 | 0.0179 | #NUM! | RAP2A,TCF4 |
| eNOS Signaling | 0 | 0.0174 | #NUM! | CALM1 (includes others),CASP9,HSPA1A/HSPA1B |
| VEGF Family Ligand-Receptor Interactions | 0 | 0.0106 | #NUM! | RAP2A |
| Ephrin A Signaling | 0 | 0.0167 | #NUM! | PTK2 |
| ErbB Signaling | 0 | 0.00962 | #NUM! | RAP2A |
| ErbB4 Signaling | 0 | 0.0128 | #NUM! | RAP2A |
| GDNF Family Ligand-Receptor Interactions | 0 | 0.0122 | #NUM! | RAP2A |
| Netrin Signaling | 0 | 0.0154 | #NUM! | ENAH |
| D-myo-inositol-5-phosphate Metabolism | 0 | 0.0123 | #NUM! | SET,NUDT11 |
| Triacylglycerol Degradation | 0 | 0.0185 | #NUM! | ABHD16A |
| Phospholipases | 0 | 0.0161 | #NUM! | PAFAH1B2 |
| Chondroitin Sulfate Biosynthesis | 0 | 0.0175 | #NUM! | CHST9 |
| Dermatan Sulfate Biosynthesis | 0 | 0.0169 | #NUM! | DSEL |
| D-myo-inositol (1,4,5,6)-Tetrakisphosphate Biosynthesis | 0 | 0.0139 | #NUM! | SET,NUDT11 |
| Superpathway of Inositol Phosphate Compounds | 0 | 0.0127 | #NUM! | SET,NUDT11,PDGFRA |
| D-myo-inositol (3,4,5,6)-tetrakisphosphate Biosynthesis | 0 | 0.0139 | #NUM! | SET,NUDT11 |
| 3-phosphoinositide Degradation | 0 | 0.0127 | #NUM! | SET,NUDT11 |
| 3-phosphoinositide Biosynthesis | 0 | 0.0149 | #NUM! | SET,NUDT11,PDGFRA |
| Antioxidant Action of Vitamin C | 0 | 0.00926 | #NUM! | PAFAH1B2 |
| Gαq Signaling | 0 | 0.0124 | #NUM! | CALM1 (includes others),RACK1 |
| Gαs Signaling | 0 | 0.0182 | #NUM! | VIPR2,RACK1 |
| Remodeling of Epithelial Adherens Junctions | 0 | 0.0145 | #NUM! | TUBA1A |
| Agranulocyte Adhesion and Diapedesis | 0 | 0.00518 | #NUM! | GNAI1 |
| Granulocyte Adhesion and Diapedesis | 0 | 0.011 | #NUM! | GNAI1,THY1 |
| Tec Kinase Signaling | 0 | 0.0176 | #NUM! | PTK2,RACK1,GNAI1 |
| UVA-Induced MAPK Signaling | 0 | 0.0179 | #NUM! | RAP2A,CASP9 |
| UVB-Induced MAPK Signaling | 0 | 0.0152 | #NUM! | H3F3A/H3F3B |
| PCP pathway | 0 | 0.0164 | #NUM! | PFN2 |
| Unfolded protein response | 0 | 0.0179 | #NUM! | HSPA1A/HSPA1B |
| Toll-like Receptor Signaling | 0 | 0.0132 | #NUM! | RPS27A |
| Nitric Oxide Signaling in the Cardiovascular System | 0 | 0.00885 | #NUM! | CALM1 (includes others) |
| Cardiac β-adrenergic Signaling | 0 | 0.0142 | #NUM! | RACK1,PPP1CB |
| JAK/Stat Signaling | 0 | 0.0112 | #NUM! | RAP2A |
| Xenobiotic Metabolism Signaling | 0 | 0.0135 | #NUM! | RAP2A,GSTT2/GSTT2B,GSTA4,UGT8 |
| IL-4 Signaling | 0 | 0.0105 | #NUM! | RAP2A |
| B Cell Receptor Signaling | 0 | 0.0155 | #NUM! | PTK2,RAP2A,CALM1 (includes others) |
| Death Receptor Signaling | 0 | 0.0108 | #NUM! | CASP9 |
| Dopamine Receptor Signaling | 0 | 0.013 | #NUM! | PPP1CB |
| cAMP-mediated signaling | 0 | 0.0175 | 2 | CALM1 (includes others),VIPR2,GNAI1,GABBR1 |
| p38 MAPK Signaling | 0 | 0.0167 | #NUM! | H3F3A/H3F3B,MAPT |
| T Cell Receptor Signaling | 0 | 0.0174 | #NUM! | RAP2A,CALM1 (includes others) |
| G-Protein Coupled Receptor Signaling | 0 | 0.0142 | #NUM! | RAP2A,VIPR2,GNAI1,GABBR1 |
| GPCR-Mediated Nutrient Sensing in Enteroendocrine Cells | 0 | 0.0179 | #NUM! | GNAI1,CCK |
| Gustation Pathway | 0 | 0.00649 | #NUM! | P2RX7 |
| Phagosome Formation | 0 | 0.00763 | #NUM! | MARCKS |
| Macropinocytosis Signaling | 0 | 0.0115 | #NUM! | RAP2A |
| Cancer Drug Resistance By Drug Efflux | 0 | 0.0182 | #NUM! | RAP2A |
| Th1 and Th2 Activation Pathway | 0 | 0.00541 | #NUM! | DLL1 |
| Th1 Pathway | 0 | 0.00741 | #NUM! | DLL1 |
| Th2 Pathway | 0 | 0.00667 | #NUM! | DLL1 |
| GP6 Signaling Pathway | 0 | 0.0149 | #NUM! | PTK2,CALM1 (includes others) |
| Neuroinflammation Signaling Pathway | 0 | 0.0161 | 1 | MAPT,GAD1,GABBR1,P2RX7,APP |
| Opioid Signaling Pathway | 0 | 0.0121 | #NUM! | RAP2A,CALM1 (includes others),GNAI1 |
| Eicosanoid Signaling | 0 | 0.0149 | #NUM! | PAFAH1B2 |

| Cluster 2 |  |  |  |  |
| --- | --- | --- | --- | --- |
| © 2000-2018 QIAGEN. All rights reserved. |  |  |  |  |
| Ingenuity Canonical Pathways | -log(B-H p-value) | Ratio | z-score | Molecules |
| 14-3-3-mediated Signaling | 2.35 | 0.0511 | 1 | FOS,BAD,TUBB2A,VIM,GFAP,TUBA1C,SFN |
| NRF2-mediated Oxidative Stress Response | 2.35 | 0.0402 | 1 | GSTM1,FOS,MGST1,SOD2,TXN,SQSTM1,CBR1,GSTK1 |
| IGF-1 Signaling | 2.22 | 0.0536 | #NUM! | FOS,BAD,IGFBP7,CYR61,SFN,IGFBP2 |
| Aryl Hydrocarbon Receptor Signaling | 1.79 | 0.0426 | #NUM! | CDKN2A,GSTM1,FOS,MGST1,GSTK1,HSPB1 |
| Glutathione Redox Reactions I | 1.65 | 0.125 | #NUM! | GSTM1,MGST1,GSTK1 |
| Acute Phase Response Signaling | 1.47 | 0.0341 | #NUM! | C1R,FOS,SOD2,C1S,SERPINA3,RBP1 |
| Glutathione-mediated Detoxification | 1.47 | 0.0968 | #NUM! | GSTM1,MGST1,GSTK1 |
| LPS/IL-1 Mediated Inhibition of RXR Function | 1.08 | 0.027 | #NUM! | GSTM1,MGST1,SMOX,FABP7,FABP5,GSTK1 |
| CCR5 Signaling in Macrophages | 1.08 | 0.0421 | #NUM! | CALM1 (includes others),FOS,GNB2,GNG5 |
| Cell Cycle: G2/M DNA Damage Checkpoint Regulation | 1.02 | 0.06 | #NUM! | CDKN2A,GADD45A,SFN |
| Mitochondrial Dysfunction | 1.01 | 0.0292 | #NUM! | SOD2,ATP5MF,ATP5ME,NDUFB2,ATP5F1E |
| Oxidative Phosphorylation | 1.01 | 0.0367 | 2 | ATP5MF,ATP5ME,NDUFB2,ATP5F1E |
| Signaling by Rho Family GTPases | 1.01 | 0.0238 | 2 | FOS,RHOC,GNB2,VIM,GFAP,GNG5 |
| Glutamate Receptor Signaling | 1.01 | 0.0526 | #NUM! | CALM1 (includes others),SLC1A3,GNG5 |
| Atherosclerosis Signaling | 0.854 | 0.0315 | #NUM! | PLA2G5,F3,CLU,TNFRSF12A |
| ERK5 Signaling | 0.844 | 0.0417 | #NUM! | FOS,BAD,SFN |
| Breast Cancer Regulation by Stathmin1 | 0.844 | 0.0237 | #NUM! | CALM1 (includes others),TUBB2A,GNB2,TUBA1C,GNG5 |
| CCR3 Signaling in Eosinophils | 0.844 | 0.0294 | #NUM! | CALM1 (includes others),PLA2G5,GNB2,GNG5 |
| Androgen Signaling | 0.844 | 0.0292 | #NUM! | POLR2G,CALM1 (includes others),GNB2,GNG5 |
| Myc Mediated Apoptosis Signaling | 0.838 | 0.0395 | #NUM! | CDKN2A,BAD,SFN |
| Glioma Invasiveness Signaling | 0.838 | 0.0395 | #NUM! | TIMP1,RHOC,CD44 |
| Glycine Degradation (Creatine Biosynthesis) | 0.836 | 0.5 | #NUM! | GATM |
| G Protein Signaling Mediated by Tubby | 0.808 | 0.0625 | #NUM! | GNB2,GNG5 |
| HIPPO signaling | 0.808 | 0.0345 | #NUM! | WWTR1,CD44,SFN |
| Cardiac Hypertrophy Signaling | 0.808 | 0.0207 | 2 | CALM1 (includes others),RHOC,GNB2,GNG5,HSPB1 |
| Gαq Signaling | 0.808 | 0.0248 | #NUM! | CALM1 (includes others),RHOC,GNB2,GNG5 |
| Phospholipase C Signaling | 0.808 | 0.0205 | 2 | CALM1 (includes others),RHOC,PLA2G5,GNB2,GNG5 |
| L-carnitine Biosynthesis | 0.808 | 0.333 | #NUM! | BBOX1 |
| IL-1 Signaling | 0.808 | 0.0326 | #NUM! | FOS,GNB2,GNG5 |
| Huntington's Disease Signaling | 0.808 | 0.02 | #NUM! | POLR2G,GNB2,HAP1,GNG5,ATP5F1E |
| IL-17A Signaling in Fibroblasts | 0.808 | 0.0571 | #NUM! | FOS,CEBPD |
| α-Adrenergic Signaling | 0.808 | 0.0323 | #NUM! | CALM1 (includes others),GNB2,GNG5 |
| Colorectal Cancer Metastasis Signaling | 0.808 | 0.0197 | 1 | FOS,BAD,RHOC,GNB2,GNG5 |
| Tec Kinase Signaling | 0.808 | 0.0235 | #NUM! | FOS,RHOC,GNB2,GNG5 |
| CXCR4 Signaling | 0.808 | 0.0234 | #NUM! | FOS,RHOC,GNB2,GNG5 |
| Complement System | 0.808 | 0.0541 | #NUM! | C1R,C1S |
| RhoGDI Signaling | 0.808 | 0.0226 | #NUM! | RHOC,GNB2,CD44,GNG5 |
| Retinoate Biosynthesis II | 0.808 | 0.25 | #NUM! | RBP1 |
| Heme Degradation | 0.808 | 0.25 | #NUM! | BLVRB |
| Spermine and Spermidine Degradation I | 0.808 | 0.25 | #NUM! | SMOX |
| Melatonin Degradation II | 0.808 | 0.25 | #NUM! | SMOX |
| Oncostatin M Signaling | 0.803 | 0.05 | #NUM! | MT2A,CHI3L1 |
| nNOS Signaling in Skeletal Muscle Cells | 0.793 | 0.0488 | #NUM! | CALM1 (includes others),SNTA1 |
| MIF Regulation of Innate Immunity | 0.765 | 0.0465 | #NUM! | FOS,PLA2G5 |
| Role of NFAT in Regulation of the Immune Response | 0.756 | 0.0208 | #NUM! | CALM1 (includes others),FOS,GNB2,GNG5 |
| iNOS Signaling | 0.756 | 0.0444 | #NUM! | CALM1 (includes others),FOS |
| p53 Signaling | 0.756 | 0.027 | #NUM! | CDKN2A,GADD45A,SFN |
| ILK Signaling | 0.732 | 0.0203 | #NUM! | FOS,RHOC,VIM,TMSB10/TMSB4X |
| Sirtuin Signaling Pathway | 0.727 | 0.0171 | 1 | SOD2,GADD45A,TUBA1C,NDUFB2,ATP5F1E |
| IL-8 Signaling | 0.711 | 0.0197 | #NUM! | FOS,RHOC,GNB2,GNG5 |
| ERK/MAPK Signaling | 0.711 | 0.0196 | 1 | FOS,BAD,PLA2G5,HSPB1 |
| Thioredoxin Pathway | 0.676 | 0.143 | #NUM! | TXN |
| Osteoarthritis Pathway | 0.676 | 0.0189 | #NUM! | RARRES2,ANXA5,ANXA2,HES1 |
| fMLP Signaling in Neutrophils | 0.656 | 0.0233 | #NUM! | CALM1 (includes others),GNB2,GNG5 |
| CREB Signaling in Neurons | 0.656 | 0.0183 | #NUM! | POLR2G,CALM1 (includes others),GNB2,GNG5 |
| Superoxide Radicals Degradation | 0.648 | 0.125 | #NUM! | SOD2 |
| Melanoma Signaling | 0.605 | 0.0328 | #NUM! | CDKN2A,BAD |
| HMGB1 Signaling | 0.605 | 0.0216 | #NUM! | FOS,RHOC,IL25 |
| P2Y Purigenic Receptor Signaling Pathway | 0.605 | 0.0214 | #NUM! | FOS,GNB2,GNG5 |
| Calcium Transport I | 0.584 | 0.1 | #NUM! | ANXA5 |
| UVB-Induced MAPK Signaling | 0.571 | 0.0303 | #NUM! | FOS,BAD |
| Hereditary Breast Cancer Signaling | 0.571 | 0.02 | #NUM! | POLR2G,GADD45A,SFN |
| Ovarian Cancer Signaling | 0.571 | 0.02 | #NUM! | CDKN2A,GJA1,CD44 |
| Opioid Signaling Pathway | 0.571 | 0.0162 | 1 | CALM1 (includes others),FOS,BAD,GNG5 |
| IL-10 Signaling | 0.571 | 0.029 | #NUM! | FOS,BLVRB |
| Remodeling of Epithelial Adherens Junctions | 0.571 | 0.029 | #NUM! | TUBB2A,TUBA1C |
| Relaxin Signaling | 0.541 | 0.019 | #NUM! | FOS,GNB2,GNG5 |
| Ephrin B Signaling | 0.541 | 0.0274 | #NUM! | GNB2,GNG5 |
| Chemokine Signaling | 0.511 | 0.026 | #NUM! | CALM1 (includes others),FOS |
| Phenylalanine Degradation IV (Mammalian, via Side Chain) | 0.511 | 0.0714 | #NUM! | SMOX |
| VDR/RXR Activation | 0.511 | 0.0256 | #NUM! | GADD45A,HES1 |
| Antiproliferative Role of Somatostatin Receptor 2 | 0.496 | 0.0247 | #NUM! | GNB2,GNG5 |
| GNRH Signaling | 0.496 | 0.0175 | #NUM! | CALM1 (includes others),FOS,GNG5 |
| Wnt/β-catenin Signaling | 0.496 | 0.0174 | #NUM! | CDKN2A,GJA1,CD44 |
| Non-Small Cell Lung Cancer Signaling | 0.496 | 0.0241 | #NUM! | CDKN2A,BAD |
| Extrinsic Prothrombin Activation Pathway | 0.496 | 0.0625 | #NUM! | F3 |
| Vitamin-C Transport | 0.496 | 0.0625 | #NUM! | TXN |
| Regulation of IL-2 Expression in Activated and Anergic T Lymphocytes | 0.483 | 0.0233 | #NUM! | CALM1 (includes others),FOS |
| Germ Cell-Sertoli Cell Junction Signaling | 0.483 | 0.0168 | #NUM! | RHOC,TUBB2A,TUBA1C |
| Protein Kinase A Signaling | 0.472 | 0.0125 | 1 | CALM1 (includes others),BAD,GNB2,GNG5,SFN |
| IL-3 Signaling | 0.471 | 0.0225 | #NUM! | FOS,BAD |
| GADD45 Signaling | 0.46 | 0.0526 | #NUM! | GADD45A |
| DNA damage-induced 14-3-3σ Signaling | 0.46 | 0.0526 | #NUM! | SFN |
| Xenobiotic Metabolism Signaling | 0.46 | 0.0135 | #NUM! | GSTM1,MGST1,SMOX,GSTK1 |
| VEGF Family Ligand-Receptor Interactions | 0.453 | 0.0213 | #NUM! | FOS,PLA2G5 |
| The Visual Cycle | 0.453 | 0.05 | #NUM! | RBP1 |
| Sumoylation Pathway | 0.453 | 0.0208 | #NUM! | FOS,RHOC |
| Production of Nitric Oxide and Reactive Oxygen Species in Macrophages | 0.453 | 0.0155 | #NUM! | FOS,RHOC,CLU |
| Ceramide Signaling | 0.439 | 0.0202 | #NUM! | FOS,BAD |
| Adrenomedullin signaling pathway | 0.439 | 0.015 | #NUM! | CALM1 (includes others),FOS,BAD |
| Neuroinflammation Signaling Pathway | 0.439 | 0.0129 | #NUM! | FOS,SOD2,PLA2G5,SLC1A3 |
| Gap Junction Signaling | 0.439 | 0.0149 | #NUM! | GJA1,TUBB2A,TUBA1C |
| RANK Signaling in Osteoclasts | 0.434 | 0.0196 | #NUM! | CALM1 (includes others),FOS |
| Putrescine Degradation III | 0.434 | 0.0435 | #NUM! | SMOX |
| Thrombin Signaling | 0.422 | 0.0143 | #NUM! | RHOC,GNB2,GNG5 |
| Cholecystokinin/Gastrin-mediated Signaling | 0.422 | 0.0187 | #NUM! | FOS,RHOC |
| IL-17A Signaling in Gastric Cells | 0.422 | 0.04 | #NUM! | FOS |
| Tryptophan Degradation X (Mammalian, via Tryptamine) | 0.422 | 0.04 | #NUM! | SMOX |
| Antioxidant Action of Vitamin C | 0.422 | 0.0185 | #NUM! | PLA2G5,TXN |
| VEGF Signaling | 0.422 | 0.0183 | #NUM! | BAD,SFN |
| Gαs Signaling | 0.422 | 0.0182 | #NUM! | GNB2,GNG5 |
| SAPK/JNK Signaling | 0.422 | 0.0182 | #NUM! | GADD45A,GNG5 |
| Chronic Myeloid Leukemia Signaling | 0.418 | 0.0179 | #NUM! | CDKN2A,BAD |
| Mouse Embryonic Stem Cell Pluripotency | 0.418 | 0.0179 | #NUM! | ID3,ID4 |
| T Cell Receptor Signaling | 0.407 | 0.0174 | #NUM! | CALM1 (includes others),FOS |
| Role of NFAT in Cardiac Hypertrophy | 0.407 | 0.0133 | #NUM! | CALM1 (includes others),GNB2,GNG5 |
| HGF Signaling | 0.407 | 0.0168 | #NUM! | CDKN2A,FOS |
| TNFR2 Signaling | 0.407 | 0.0333 | #NUM! | FOS |
| Glioma Signaling | 0.407 | 0.0167 | #NUM! | CDKN2A,CALM1 (includes others) |
| Pancreatic Adenocarcinoma Signaling | 0.407 | 0.0167 | #NUM! | CDKN2A,BAD |
| G Beta Gamma Signaling | 0.407 | 0.0167 | #NUM! | GNB2,GNG5 |
| Neuroprotective Role of THOP1 in Alzheimer's Disease | 0.407 | 0.0167 | #NUM! | C1R,SERPINA3 |
| p38 MAPK Signaling | 0.407 | 0.0167 | #NUM! | PLA2G5,HSPB1 |
| Role of Osteoblasts, Osteoclasts and Chondrocytes in Rheumatoid Arthritis | 0.402 | 0.0129 | #NUM! | CALM1 (includes others),FOS,BAD |
| iCOS-iCOSL Signaling in T Helper Cells | 0.402 | 0.0163 | #NUM! | CALM1 (includes others),BAD |
| Gαi Signaling | 0.402 | 0.0163 | #NUM! | GNB2,GNG5 |
| Sperm Motility | 0.391 | 0.0157 | #NUM! | CALM1 (includes others),PLA2G5 |
| DNA Methylation and Transcriptional Repression Signaling | 0.391 | 0.0294 | #NUM! | HIST4H4 |
| Retinoate Biosynthesis I | 0.391 | 0.0294 | #NUM! | RBP1 |
| Role of Tissue Factor in Cancer | 0.391 | 0.0154 | #NUM! | CYR61,F3 |
| PI3K/AKT Signaling | 0.391 | 0.0153 | #NUM! | BAD,SFN |
| Coagulation System | 0.391 | 0.0286 | #NUM! | F3 |
| TWEAK Signaling | 0.391 | 0.0286 | #NUM! | TNFRSF12A |
| MIF-mediated Glucocorticoid Regulation | 0.391 | 0.0286 | #NUM! | PLA2G5 |
| Nucleotide Excision Repair Pathway | 0.391 | 0.0286 | #NUM! | POLR2G |
| CD28 Signaling in T Helper Cells | 0.391 | 0.0152 | #NUM! | CALM1 (includes others),FOS |
| Adipogenesis pathway | 0.388 | 0.0149 | #NUM! | CEBPD,RBP1 |
| IL-6 Signaling | 0.388 | 0.0149 | #NUM! | FOS,HSPB1 |
| Dopamine Degradation | 0.385 | 0.027 | #NUM! | SMOX |
| PI3K Signaling in B Lymphocytes | 0.385 | 0.0147 | #NUM! | CALM1 (includes others),FOS |
| Notch Signaling | 0.383 | 0.0263 | #NUM! | HES1 |
| p70S6K Signaling | 0.383 | 0.0145 | #NUM! | BAD,SFN |
| Corticotropin Releasing Hormone Signaling | 0.382 | 0.0144 | #NUM! | CALM1 (includes others),FOS |
| April Mediated Signaling | 0.382 | 0.0256 | #NUM! | FOS |
| Inhibition of Matrix Metalloproteases | 0.382 | 0.0256 | #NUM! | TIMP1 |
| Cardiac β-adrenergic Signaling | 0.382 | 0.0142 | #NUM! | GNB2,GNG5 |
| Noradrenaline and Adrenaline Degradation | 0.379 | 0.025 | #NUM! | SMOX |
| B Cell Activating Factor Signaling | 0.373 | 0.0244 | #NUM! | FOS |
| Molecular Mechanisms of Cancer | 0.373 | 0.0102 | #NUM! | CDKN2A,FOS,BAD,RHOC |
| IL-12 Signaling and Production in Macrophages | 0.373 | 0.0137 | #NUM! | FOS,CLU |
| Retinol Biosynthesis | 0.373 | 0.0238 | #NUM! | RBP1 |
| Phagosome Maturation | 0.373 | 0.0135 | #NUM! | TUBB2A,TUBA1C |
| Role of p14/p19ARF in Tumor Suppression | 0.373 | 0.0233 | #NUM! | CDKN2A |
| Serotonin Receptor Signaling | 0.373 | 0.0233 | #NUM! | SMOX |
| Epithelial Adherens Junction Signaling | 0.37 | 0.0133 | #NUM! | TUBB2A,TUBA1C |
| Pyrimidine Ribonucleotides Interconversion | 0.362 | 0.0222 | #NUM! | ANXA1 |
| nNOS Signaling in Neurons | 0.352 | 0.0213 | #NUM! | CALM1 (includes others) |
| Pyrimidine Ribonucleotides De Novo Biosynthesis | 0.352 | 0.0213 | #NUM! | ANXA1 |
| UVC-Induced MAPK Signaling | 0.339 | 0.0204 | #NUM! | FOS |
| TNFR1 Signaling | 0.337 | 0.02 | #NUM! | FOS |
| Assembly of RNA Polymerase II Complex | 0.337 | 0.02 | #NUM! | POLR2G |
| Docosahexaenoic Acid (DHA) Signaling | 0.33 | 0.0192 | #NUM! | BAD |
| Aldosterone Signaling in Epithelial Cells | 0.33 | 0.0119 | #NUM! | CRYAB,HSPB1 |
| Glioblastoma Multiforme Signaling | 0.33 | 0.0119 | #NUM! | CDKN2A,RHOC |
| CD27 Signaling in Lymphocytes | 0.33 | 0.0189 | #NUM! | FOS |
| Semaphorin Signaling in Neurons | 0.33 | 0.0189 | #NUM! | RHOC |
| Role of Cytokines in Mediating Communication between Immune Cells | 0.329 | 0.0185 | #NUM! | IL25 |
| Transcriptional Regulatory Network in Embryonic Stem Cells | 0.329 | 0.0185 | #NUM! | HIST4H4 |
| eNOS Signaling | 0.327 | 0.0116 | #NUM! | CALM1 (includes others),AQP4 |
| Unfolded protein response | 0.321 | 0.0179 | #NUM! | CEBPD |
| Ephrin Receptor Signaling | 0.309 | 0.0112 | #NUM! | GNB2,GNG5 |
| Nur77 Signaling in T Lymphocytes | 0.308 | 0.0169 | #NUM! | CALM1 (includes others) |
| Granulocyte Adhesion and Diapedesis | 0.308 | 0.011 | #NUM! | CXCL14,HSPB1 |
| Axonal Guidance Signaling | 0.308 | 0.00875 | #NUM! | TUBB2A,GNB2,TUBA1C,GNG5 |
| Sertoli Cell-Sertoli Cell Junction Signaling | 0.308 | 0.0109 | #NUM! | TUBB2A,TUBA1C |
| Role of Macrophages, Fibroblasts and Endothelial Cells in Rheumatoid Arthritis | 0.308 | 0.00938 | #NUM! | CALM1 (includes others),FOS,CEBPD |
| Actin Nucleation by ARP-WASP Complex | 0.308 | 0.0161 | #NUM! | RHOC |
| Phospholipases | 0.308 | 0.0161 | #NUM! | PLA2G5 |
| autophagy | 0.308 | 0.0161 | #NUM! | SQSTM1 |
| RAR Activation | 0.298 | 0.0105 | #NUM! | FOS,RBP1 |
| PXR/RXR Activation | 0.296 | 0.0154 | #NUM! | GSTM1 |
| Calcium-induced T Lymphocyte Apoptosis | 0.296 | 0.0152 | #NUM! | CALM1 (includes others) |
| B Cell Receptor Signaling | 0.296 | 0.0103 | #NUM! | CALM1 (includes others),BAD |
| Cell Cycle: G1/S Checkpoint Regulation | 0.296 | 0.0149 | #NUM! | CDKN2A |
| Eicosanoid Signaling | 0.296 | 0.0149 | #NUM! | PLA2G5 |
| Endothelin-1 Signaling | 0.296 | 0.0102 | #NUM! | FOS,PLA2G5 |
| EGF Signaling | 0.295 | 0.0147 | #NUM! | FOS |
| Endometrial Cancer Signaling | 0.293 | 0.0143 | #NUM! | BAD |
| Superpathway of Melatonin Degradation | 0.293 | 0.0143 | #NUM! | SMOX |
| IL-2 Signaling | 0.293 | 0.0143 | #NUM! | FOS |
| Caveolar-mediated Endocytosis Signaling | 0.293 | 0.0141 | #NUM! | ITGA7 |
| Thrombopoietin Signaling | 0.293 | 0.0141 | #NUM! | FOS |
| Glucocorticoid Receptor Signaling | 0.292 | 0.0087 | #NUM! | POLR2G,FOS,ANXA1 |
| Melatonin Signaling | 0.292 | 0.0139 | #NUM! | CALM1 (includes others) |
| ErbB2-ErbB3 Signaling | 0.281 | 0.0133 | #NUM! | BAD |
| Leukocyte Extravasation Signaling | 0.281 | 0.00948 | #NUM! | TIMP1,CD44 |
| Toll-like Receptor Signaling | 0.281 | 0.0132 | #NUM! | FOS |
| Serotonin Degradation | 0.281 | 0.013 | #NUM! | SMOX |
| Dopamine Receptor Signaling | 0.281 | 0.013 | #NUM! | SMOX |
| Role of MAPK Signaling in the Pathogenesis of Influenza | 0.279 | 0.0128 | #NUM! | PLA2G5 |
| CD40 Signaling | 0.277 | 0.0127 | #NUM! | FOS |
| Role of BRCA1 in DNA Damage Response | 0.275 | 0.0125 | #NUM! | GADD45A |
| Integrin Signaling | 0.275 | 0.00913 | #NUM! | RHOC,ITGA7 |
| Cyclins and Cell Cycle Regulation | 0.275 | 0.0123 | #NUM! | CDKN2A |
| GDNF Family Ligand-Receptor Interactions | 0.274 | 0.0122 | #NUM! | FOS |
| Neurotrophin/TRK Signaling | 0.272 | 0.012 | #NUM! | FOS |
| Angiopoietin Signaling | 0.271 | 0.0119 | #NUM! | BAD |
| Growth Hormone Signaling | 0.271 | 0.0118 | #NUM! | FOS |
| Estrogen-Dependent Breast Cancer Signaling | 0.271 | 0.0116 | #NUM! | FOS |
| Erythropoietin Signaling | 0.271 | 0.0115 | #NUM! | FOS |
| Macropinocytosis Signaling | 0.271 | 0.0115 | #NUM! | RAB34 |
| Renal Cell Carcinoma Signaling | 0.271 | 0.0114 | #NUM! | FOS |
| Prolactin Signaling | 0.271 | 0.0112 | #NUM! | FOS |
| JAK/Stat Signaling | 0.271 | 0.0112 | #NUM! | FOS |
| Regulation of Actin-based Motility by Rho | 0.271 | 0.0111 | #NUM! | RHOC |
| IL-17 Signaling | 0.271 | 0.011 | #NUM! | TIMP1 |
| IL-7 Signaling Pathway | 0.271 | 0.011 | #NUM! | BAD |
| LPS-stimulated MAPK Signaling | 0.271 | 0.0108 | #NUM! | FOS |
| FLT3 Signaling in Hematopoietic Progenitor Cells | 0.271 | 0.0108 | #NUM! | BAD |
| PEDF Signaling | 0.271 | 0.0108 | #NUM! | SOD2 |
| Death Receptor Signaling | 0.271 | 0.0108 | #NUM! | HSPB1 |
| TGF-β Signaling | 0.271 | 0.0108 | #NUM! | FOS |
| Neuregulin Signaling | 0.271 | 0.0106 | #NUM! | BAD |
| Bladder Cancer Signaling | 0.271 | 0.0106 | #NUM! | CDKN2A |
| HER-2 Signaling in Breast Cancer | 0.271 | 0.0106 | #NUM! | BAD |
| Apoptosis Signaling | 0.269 | 0.0104 | #NUM! | BAD |
| PDGF Signaling | 0.269 | 0.0104 | #NUM! | FOS |
| ATM Signaling | 0.264 | 0.0102 | #NUM! | GADD45A |
| Acute Myeloid Leukemia Signaling | 0.263 | 0.0101 | #NUM! | BAD |
| PPAR Signaling | 0.258 | 0.0099 | #NUM! | FOS |
| Prostate Cancer Signaling | 0.254 | 0.00971 | #NUM! | BAD |
| ErbB Signaling | 0.253 | 0.00962 | #NUM! | FOS |
| Protein Ubiquitination Pathway | 0.243 | 0.00755 | #NUM! | CRYAB,HSPB1 |
| Type I Diabetes Mellitus Signaling | 0.238 | 0.00901 | #NUM! | CPE |
| UVA-Induced MAPK Signaling | 0.238 | 0.00893 | #NUM! | FOS |
| GPCR-Mediated Nutrient Sensing in Enteroendocrine Cells | 0.238 | 0.00893 | #NUM! | GNG5 |
| Nitric Oxide Signaling in the Cardiovascular System | 0.238 | 0.00885 | #NUM! | CALM1 (includes others) |
| Neuropathic Pain Signaling In Dorsal Horn Neurons | 0.234 | 0.0087 | #NUM! | FOS |
| Paxillin Signaling | 0.226 | 0.0084 | #NUM! | ITGA7 |
| LXR/RXR Activation | 0.223 | 0.00826 | #NUM! | CLU |
| Rac Signaling | 0.223 | 0.00813 | #NUM! | CD44 |
| Fc Epsilon RI Signaling | 0.223 | 0.008 | #NUM! | PLA2G5 |
| Sphingosine-1-phosphate Signaling | 0.223 | 0.008 | #NUM! | RHOC |
| PTEN Signaling | 0.223 | 0.008 | #NUM! | BAD |
| Synaptic Long Term Potentiation | 0.223 | 0.00794 | #NUM! | CALM1 (includes others) |
| FXR/RXR Activation | 0.223 | 0.00794 | #NUM! | CLU |
| Renin-Angiotensin Signaling | 0.22 | 0.00781 | #NUM! | FOS |
| Cellular Effects of Sildenafil (Viagra) | 0.217 | 0.00763 | #NUM! | CALM1 (includes others) |
| Phagosome Formation | 0.217 | 0.00763 | #NUM! | RHOC |
| Estrogen Receptor Signaling | 0.214 | 0.00746 | #NUM! | POLR2G |
| GP6 Signaling Pathway | 0.214 | 0.00746 | #NUM! | CALM1 (includes others) |
| Role of Pattern Recognition Receptors in Recognition of Bacteria and Viruses | 0.21 | 0.0073 | #NUM! | IL25 |
| Insulin Receptor Signaling | 0.193 | 0.0068 | #NUM! | BAD |
| Th2 Pathway | 0.189 | 0.00667 | #NUM! | IL25 |
| Gustation Pathway | 0.184 | 0.00649 | #NUM! | GNG5 |
| Hepatic Cholestasis | 0.176 | 0.00625 | #NUM! | IL25 |
| Actin Cytoskeleton Signaling | 0 | 0.00429 | #NUM! | TMSB10/TMSB4X |
| Synaptic Long Term Depression | 0 | 0.00556 | #NUM! | PLA2G5 |
| Hepatic Fibrosis / Hepatic Stellate Cell Activation | 0 | 0.00535 | #NUM! | TIMP1 |
| Tight Junction Signaling | 0 | 0.00599 | #NUM! | FOS |
| Clathrin-mediated Endocytosis Signaling | 0 | 0.00483 | #NUM! | CLU |
| mTOR Signaling | 0 | 0.00483 | #NUM! | RHOC |
| Systemic Lupus Erythematosus Signaling | 0 | 0.00429 | #NUM! | FOS |
| Cdc42 Signaling | 0 | 0.00599 | #NUM! | FOS |
| PKCθ Signaling in T Lymphocytes | 0 | 0.00606 | #NUM! | FOS |
| Dopamine-DARPP32 Feedback in cAMP Signaling | 0 | 0.0061 | #NUM! | CALM1 (includes others) |
| Agranulocyte Adhesion and Diapedesis | 0 | 0.00518 | #NUM! | CXCL14 |
| cAMP-mediated signaling | 0 | 0.00439 | #NUM! | CALM1 (includes others) |
| Th1 and Th2 Activation Pathway | 0 | 0.00541 | #NUM! | IL25 |
| Calcium Signaling | 0 | 0.00485 | #NUM! | CALM1 (includes others) |

| Cluster 3 |  |  |  |  |
| --- | --- | --- | --- | --- |
| © 2000-2018 QIAGEN. All rights reserved. |  |  |  |  |
| Ingenuity Canonical Pathways | -log(B-H p-value) | Ratio | z-score | Molecules |
| Phagosome Maturation | 1.34 | 0.0405 | #NUM! | B2M,TUBA1A,TUBB2A,TUBB4A,SNAP25,TUBB2B |
| Breast Cancer Regulation by Stathmin1 | 1.34 | 0.0332 | #NUM! | STMN1,CALM1 (includes others),TUBA1A,TUBB2A,TUBB4A,GNG3,TUBB2B |
| Remodeling of Epithelial Adherens Junctions | 1.18 | 0.058 | #NUM! | TUBA1A,TUBB2A,TUBB4A,TUBB2B |
| 14-3-3-mediated Signaling | 1.03 | 0.0365 | #NUM! | TUBA1A,YWHAH,TUBB2A,TUBB4A,TUBB2B |
| Oleate Biosynthesis II (Animals) | 1.03 | 0.154 | #NUM! | SCD5,FADS2 |
| Epithelial Adherens Junction Signaling | 1.03 | 0.0333 | #NUM! | EPN2,TUBA1A,TUBB2A,TUBB4A,TUBB2B |
| Germ Cell-Sertoli Cell Junction Signaling | 0.782 | 0.0279 | #NUM! | EPN2,TUBA1A,TUBB2A,TUBB4A,TUBB2B |
| Sertoli Cell-Sertoli Cell Junction Signaling | 0.782 | 0.0272 | #NUM! | EPN2,TUBA1A,TUBB2A,TUBB4A,TUBB2B |
| Gap Junction Signaling | 0.678 | 0.0249 | #NUM! | TUBA1A,TUBB2A,GRIA2,TUBB4A,TUBB2B |
| Glycine Degradation (Creatine Biosynthesis) | 0.65 | 0.5 | #NUM! | GATM |
| Interferon Signaling | 0.436 | 0.0556 | #NUM! | IFI6,ISG15 |
| Retinoate Biosynthesis II | 0.431 | 0.25 | #NUM! | RBP1 |
| Axonal Guidance Signaling | 0.362 | 0.0153 | #NUM! | LRRC4C,TUBA1A,TUBB2A,TUBB4A,GNG3,EFNA1,TUBB2B |
| Sirtuin Signaling Pathway | 0.257 | 0.0171 | 2 | SIRT2,SLC25A4,TUBA1A,GABARAPL2,MAP1LC3A |
| Glutamate Receptor Signaling | 0.247 | 0.0351 | #NUM! | CALM1 (includes others),GRIA2 |
| Prostanoid Biosynthesis | 0.247 | 0.111 | #NUM! | PTGDS |
| Sucrose Degradation V (Mammalian) | 0.247 | 0.111 | #NUM! | ALDOC |
| Adipogenesis pathway | 0.247 | 0.0224 | #NUM! | SIRT2,BSCL2,RBP1 |
| Protein Ubiquitination Pathway | 0.136 | 0.0151 | #NUM! | B2M,UCHL1,CRYAB,DNAJC6 |
| Parkinson's Signaling | 0.136 | 0.0625 | #NUM! | UCHL1 |
| γ-linolenate Biosynthesis II (Animals) | 0.136 | 0.0588 | #NUM! | FADS2 |
| GADD45 Signaling | 0.136 | 0.0526 | #NUM! | CDK4 |
| The Visual Cycle | 0.136 | 0.05 | #NUM! | RBP1 |
| α-Adrenergic Signaling | 0.136 | 0.0215 | #NUM! | CALM1 (includes others),GNG3 |
| CCR5 Signaling in Macrophages | 0.136 | 0.0211 | #NUM! | CALM1 (includes others),GNG3 |
| GABA Receptor Signaling | 0.136 | 0.0211 | #NUM! | KCNQ2,SLC6A1 |
| NRF2-mediated Oxidative Stress Response | 0.136 | 0.0151 | #NUM! | GSTM3,DNAJC6,CBR1 |
| Lipid Antigen Presentation by CD1 | 0.136 | 0.0385 | #NUM! | B2M |
| Estrogen-mediated S-phase Entry | 0.136 | 0.0385 | #NUM! | CDK4 |
| Glycolysis I | 0.136 | 0.0385 | #NUM! | ALDOC |
| Gluconeogenesis I | 0.136 | 0.0385 | #NUM! | ALDOC |
| CREB Signaling in Neurons | 0.136 | 0.0138 | #NUM! | CALM1 (includes others),GRIA2,GNG3 |
| Neuropathic Pain Signaling In Dorsal Horn Neurons | 0.136 | 0.0174 | #NUM! | KCNQ2,GRIA2 |
| Glutathione-mediated Detoxification | 0.136 | 0.0323 | #NUM! | GSTM3 |
| Glioma Signaling | 0.136 | 0.0167 | #NUM! | CALM1 (includes others),CDK4 |
| Cytotoxic T Lymphocyte-mediated Apoptosis of Target Cells | 0.136 | 0.0312 | #NUM! | B2M |
| G Protein Signaling Mediated by Tubby | 0.136 | 0.0312 | #NUM! | GNG3 |
| LXR/RXR Activation | 0.136 | 0.0165 | #NUM! | CLU,APOD |
| Retinoate Biosynthesis I | 0.136 | 0.0294 | #NUM! | RBP1 |
| Synaptic Long Term Potentiation | 0.136 | 0.0159 | #NUM! | CALM1 (includes others),GRIA2 |
| FXR/RXR Activation | 0.136 | 0.0159 | #NUM! | CLU,APOD |
| Atherosclerosis Signaling | 0.136 | 0.0157 | #NUM! | CLU,APOD |
| Coagulation System | 0.136 | 0.0286 | #NUM! | PLAT |
| fMLP Signaling in Neutrophils | 0.136 | 0.0155 | #NUM! | CALM1 (includes others),GNG3 |
| Cellular Effects of Sildenafil (Viagra) | 0.136 | 0.0153 | #NUM! | CALM1 (includes others),KCNQ2 |
| Cell Cycle Regulation by BTG Family Proteins | 0.136 | 0.027 | #NUM! | CDK4 |
| Phospholipase C Signaling | 0.136 | 0.0123 | #NUM! | PEBP1,CALM1 (includes others),GNG3 |
| GP6 Signaling Pathway | 0.136 | 0.0149 | #NUM! | CALM1 (includes others),COL9A3 |
| Antigen Presentation Pathway | 0.136 | 0.0263 | #NUM! | B2M |
| CCR3 Signaling in Eosinophils | 0.136 | 0.0147 | #NUM! | CALM1 (includes others),GNG3 |
| Androgen Signaling | 0.136 | 0.0146 | #NUM! | CALM1 (includes others),GNG3 |
| Signaling by Rho Family GTPases | 0.136 | 0.0119 | #NUM! | STMN1,SEPT8,GNG3 |
| nNOS Signaling in Skeletal Muscle Cells | 0.136 | 0.0244 | #NUM! | CALM1 (includes others) |
| Aryl Hydrocarbon Receptor Signaling | 0.136 | 0.0142 | #NUM! | GSTM3,CDK4 |
| Retinol Biosynthesis | 0.136 | 0.0238 | #NUM! | RBP1 |
| IL-12 Signaling and Production in Macrophages | 0.136 | 0.0137 | #NUM! | CLU,APOD |
| iNOS Signaling | 0.136 | 0.0222 | #NUM! | CALM1 (includes others) |
| Triacylglycerol Biosynthesis | 0.136 | 0.0217 | #NUM! | PLPP4 |
| nNOS Signaling in Neurons | 0.136 | 0.0213 | #NUM! | CALM1 (includes others) |
| Cell Cycle: G2/M DNA Damage Checkpoint Regulation | 0.136 | 0.02 | #NUM! | YWHAH |
| Gαq Signaling | 0.136 | 0.0124 | #NUM! | CALM1 (includes others),GNG3 |
| Tight Junction Signaling | 0.136 | 0.012 | #NUM! | CDK4,SNAP25 |
| Aldosterone Signaling in Epithelial Cells | 0.136 | 0.0119 | #NUM! | CRYAB,DNAJC6 |
| Cell Cycle Control of Chromosomal Replication | 0.136 | 0.0179 | #NUM! | CDK4 |
| Wnt/β-catenin Signaling | 0.136 | 0.0116 | #NUM! | DKK3,SOX10 |
| Nur77 Signaling in T Lymphocytes | 0.136 | 0.0169 | #NUM! | CALM1 (includes others) |
| Ephrin A Signaling | 0.136 | 0.0167 | #NUM! | EFNA1 |
| Ephrin Receptor Signaling | 0.136 | 0.0112 | #NUM! | GNG3,EFNA1 |
| Induction of Apoptosis by HIV1 | 0.136 | 0.0164 | #NUM! | SLC25A4 |
| Melanoma Signaling | 0.136 | 0.0164 | #NUM! | CDK4 |
| autophagy | 0.136 | 0.0161 | #NUM! | MAP1LC3A |
| Neuroinflammation Signaling Pathway | 0.136 | 0.00965 | #NUM! | B2M,S100B,SLC6A1 |
| Activation of IRF by Cytosolic Pattern Recognition Receptors | 0.136 | 0.0159 | #NUM! | ISG15 |
| Regulation of Cellular Mechanics by Calpain Protease | 0.136 | 0.0159 | #NUM! | CDK4 |
| Pyridoxal 5'-phosphate Salvage Pathway | 0.136 | 0.0154 | #NUM! | CDK4 |
| Hepatic Fibrosis / Hepatic Stellate Cell Activation | 0.136 | 0.0107 | #NUM! | COL9A3,BAMBI |
| Calcium-induced T Lymphocyte Apoptosis | 0.136 | 0.0152 | #NUM! | CALM1 (includes others) |
| Cell Cycle: G1/S Checkpoint Regulation | 0.136 | 0.0149 | #NUM! | CDK4 |
| Eicosanoid Signaling | 0.136 | 0.0149 | #NUM! | PTGDS |
| Role of NFAT in Regulation of the Immune Response | 0.136 | 0.0104 | #NUM! | CALM1 (includes others),GNG3 |
| Production of Nitric Oxide and Reactive Oxygen Species in Macrophages | 0.136 | 0.0103 | #NUM! | CLU,APOD |
| Caveolar-mediated Endocytosis Signaling | 0.136 | 0.0141 | #NUM! | B2M |
| Melatonin Signaling | 0.136 | 0.0139 | #NUM! | CALM1 (includes others) |
| ERK5 Signaling | 0.136 | 0.0139 | #NUM! | YWHAH |
| Adrenomedullin signaling pathway | 0.136 | 0.01 | #NUM! | CALM1 (includes others),KCNQ2 |
| Ephrin B Signaling | 0.136 | 0.0137 | #NUM! | GNG3 |
| Myc Mediated Apoptosis Signaling | 0.136 | 0.0132 | #NUM! | YWHAH |
| Calcium Signaling | 0.136 | 0.00971 | #NUM! | CALM1 (includes others),GRIA2 |
| Clathrin-mediated Endocytosis Signaling | 0.136 | 0.00966 | #NUM! | CLU,APOD |
| Chemokine Signaling | 0.136 | 0.013 | #NUM! | CALM1 (includes others) |
| Leukocyte Extravasation Signaling | 0.136 | 0.00948 | #NUM! | EDIL3,THY1 |
| Antiproliferative Role of Somatostatin Receptor 2 | 0.136 | 0.0123 | #NUM! | GNG3 |
| Cyclins and Cell Cycle Regulation | 0.136 | 0.0123 | #NUM! | CDK4 |
| Non-Small Cell Lung Cancer Signaling | 0.136 | 0.012 | #NUM! | CDK4 |
| Integrin Signaling | 0.136 | 0.00913 | #NUM! | TSPAN3,TSPAN7 |
| Small Cell Lung Cancer Signaling | 0.136 | 0.0118 | #NUM! | CDK4 |
| Allograft Rejection Signaling | 0.136 | 0.0118 | #NUM! | B2M |
| LPS/IL-1 Mediated Inhibition of RXR Function | 0.136 | 0.00901 | #NUM! | GSTM3,FABP5 |
| Regulation of IL-2 Expression in Activated and Anergic T Lymphocytes | 0.136 | 0.0116 | #NUM! | CALM1 (includes others) |
| HIPPO signaling | 0.136 | 0.0115 | #NUM! | YWHAH |
| Role of NFAT in Cardiac Hypertrophy | 0.136 | 0.00889 | #NUM! | CALM1 (includes others),GNG3 |
| OX40 Signaling Pathway | 0.136 | 0.011 | #NUM! | B2M |
| Reelin Signaling in Neurons | 0.136 | 0.0109 | #NUM! | APBB1 |
| IL-1 Signaling | 0.136 | 0.0109 | #NUM! | GNG3 |
| Role of Osteoblasts, Osteoclasts and Chondrocytes in Rheumatoid Arthritis | 0.136 | 0.00858 | #NUM! | CALM1 (includes others),DKK3 |
| Bladder Cancer Signaling | 0.136 | 0.0106 | #NUM! | CDK4 |
| Communication between Innate and Adaptive Immune Cells | 0.136 | 0.0105 | #NUM! | B2M |
| Salvage Pathways of Pyrimidine Ribonucleotides | 0.136 | 0.0103 | #NUM! | CDK4 |
| Cardiac Hypertrophy Signaling | 0.136 | 0.0083 | #NUM! | CALM1 (includes others),GNG3 |
| CTLA4 Signaling in Cytotoxic T Lymphocytes | 0.136 | 0.0101 | #NUM! | B2M |
| RANK Signaling in Osteoclasts | 0.136 | 0.0098 | #NUM! | CALM1 (includes others) |
| Huntington's Disease Signaling | 0.136 | 0.008 | #NUM! | GNG3,SNAP25 |
| Protein Kinase A Signaling | 0.136 | 0.00748 | #NUM! | CALM1 (includes others),YWHAH,GNG3 |
| Melanocyte Development and Pigmentation Signaling | 0.136 | 0.00962 | #NUM! | SOX10 |
| Gαs Signaling | 0.136 | 0.00909 | #NUM! | GNG3 |
| Amyotrophic Lateral Sclerosis Signaling | 0.136 | 0.00901 | #NUM! | GRIA2 |
| p53 Signaling | 0.136 | 0.00901 | #NUM! | CDK4 |
| Type I Diabetes Mellitus Signaling | 0.136 | 0.00901 | #NUM! | CPE |
| Chronic Myeloid Leukemia Signaling | 0.136 | 0.00893 | #NUM! | CDK4 |
| IGF-1 Signaling | 0.136 | 0.00893 | #NUM! | YWHAH |
| GPCR-Mediated Nutrient Sensing in Enteroendocrine Cells | 0.136 | 0.00893 | #NUM! | GNG3 |
| Nitric Oxide Signaling in the Cardiovascular System | 0.136 | 0.00885 | #NUM! | CALM1 (includes others) |
| T Cell Receptor Signaling | 0.136 | 0.0087 | #NUM! | CALM1 (includes others) |
| Virus Entry via Endocytic Pathways | 0.136 | 0.00862 | #NUM! | B2M |
| Pancreatic Adenocarcinoma Signaling | 0.136 | 0.00833 | #NUM! | CDK4 |
| G Beta Gamma Signaling | 0.136 | 0.00833 | #NUM! | GNG3 |
| iCOS-iCOSL Signaling in T Helper Cells | 0.136 | 0.00813 | #NUM! | CALM1 (includes others) |
| Gαi Signaling | 0.136 | 0.00813 | #NUM! | GNG3 |
| HIF1α Signaling | 0.136 | 0.00806 | #NUM! | P4HTM |
| RhoA Signaling | 0.136 | 0.00806 | #NUM! | SEPT8 |
| PTEN Signaling | 0.136 | 0.008 | #NUM! | YWHAH |
| Sperm Motility | 0.135 | 0.00787 | #NUM! | CALM1 (includes others) |
| PI3K/AKT Signaling | 0.132 | 0.00763 | #NUM! | YWHAH |
| CD28 Signaling in T Helper Cells | 0.132 | 0.00758 | #NUM! | CALM1 (includes others) |
| PI3K Signaling in B Lymphocytes | 0.132 | 0.00735 | #NUM! | CALM1 (includes others) |
| p70S6K Signaling | 0.132 | 0.00725 | #NUM! | YWHAH |
| Corticotropin Releasing Hormone Signaling | 0.132 | 0.00719 | #NUM! | CALM1 (includes others) |
| HMGB1 Signaling | 0.132 | 0.00719 | #NUM! | PLAT |
| P2Y Purigenic Receptor Signaling Pathway | 0.132 | 0.00714 | #NUM! | GNG3 |
| Cardiac β-adrenergic Signaling | 0.132 | 0.00709 | #NUM! | GNG3 |
| Insulin Receptor Signaling | 0.126 | 0.0068 | #NUM! | VAMP2 |
| Hereditary Breast Cancer Signaling | 0.126 | 0.00667 | #NUM! | CDK4 |
| Ovarian Cancer Signaling | 0.126 | 0.00667 | #NUM! | CDK4 |
| Glucocorticoid Receptor Signaling | 0 | 0.0029 | #NUM! | YWHAH |
| Synaptic Long Term Depression | 0 | 0.00556 | #NUM! | GRIA2 |
| Acute Phase Response Signaling | 0 | 0.00568 | #NUM! | RBP1 |
| RAR Activation | 0 | 0.00526 | #NUM! | RBP1 |
| IL-8 Signaling | 0 | 0.00493 | #NUM! | GNG3 |
| CXCR4 Signaling | 0 | 0.00585 | #NUM! | GNG3 |
| Dendritic Cell Maturation | 0 | 0.00515 | #NUM! | B2M |
| Relaxin Signaling | 0 | 0.00633 | #NUM! | GNG3 |
| Thrombin Signaling | 0 | 0.00476 | #NUM! | GNG3 |
| Molecular Mechanisms of Cancer | 0 | 0.00254 | #NUM! | CDK4 |
| GNRH Signaling | 0 | 0.00585 | #NUM! | CALM1 (includes others) |
| Colorectal Cancer Metastasis Signaling | 0 | 0.00394 | #NUM! | GNG3 |
| Cdc42 Signaling | 0 | 0.00599 | #NUM! | B2M |
| Role of Macrophages, Fibroblasts and Endothelial Cells in Rheumatoid Arthritis | 0 | 0.00625 | #NUM! | CALM1 (includes others),DKK3 |
| Glioblastoma Multiforme Signaling | 0 | 0.00595 | #NUM! | CDK4 |
| Dopamine-DARPP32 Feedback in cAMP Signaling | 0 | 0.0061 | #NUM! | CALM1 (includes others) |
| RhoGDI Signaling | 0 | 0.00565 | #NUM! | GNG3 |
| eNOS Signaling | 0 | 0.00581 | #NUM! | CALM1 (includes others) |
| Granulocyte Adhesion and Diapedesis | 0 | 0.00552 | #NUM! | THY1 |
| Tec Kinase Signaling | 0 | 0.00588 | #NUM! | GNG3 |
| ERK/MAPK Signaling | 0 | 0.0049 | #NUM! | YWHAH |
| Xenobiotic Metabolism Signaling | 0 | 0.00338 | #NUM! | GSTM3 |
| B Cell Receptor Signaling | 0 | 0.00515 | #NUM! | CALM1 (includes others) |
| cAMP-mediated signaling | 0 | 0.00439 | #NUM! | CALM1 (includes others) |
| Osteoarthritis Pathway | 0 | 0.00472 | #NUM! | PRG4 |
| Opioid Signaling Pathway | 0 | 0.00405 | #NUM! | CALM1 (includes others) |

| Cluster 4 |  |  |  |  |
| --- | --- | --- | --- | --- |
| © 2000-2018 QIAGEN. All rights reserved. |  |  |  |  |
| Ingenuity Canonical Pathways | -log(B-H p-value) | Ratio | z-score | Molecules |
| Dendritic Cell Maturation | 16.8 | 0.139 | 5 | PLCB2,HLA-B,HLA-DQA1,CD83,HLA-DQB1,FCGR1A,HLA-DRB1,HLA-DMA,HLA-DRA,LTBR,TNFRSF1B,FCGR3A/FCGR3B,FCGR1B,TYROBP,GRB2,FCGR2A,TLR2,NFKBID,IL18,HLA-C,IL1RN,TREM2,FCER1G,CD86,IL1B,IRF8,HLA-DRB5 |
| Communication between Innate and Adaptive Immune Cells | 12 | 0.179 | #NUM! | CD4,HLA-B,CD83,CCL3,TLR2,IL18,CCL4,HLA-DRB1,HLA-C,CCL3L3,IL1RN,TNFSF13,HLA-DRA,FCER1G,CD86,IL1B,HLA-DRB5 |
| Graft-versus-Host Disease Signaling | 11.4 | 0.271 | #NUM! | HLA-B,HLA-DQA1,HLA-DQB1,IL18,HLA-DRB1,IL1RN,HLA-DMA,HLA-C,HLA-DRA,FCER1G,CD86,IL1B,HLA-DRB5 |
| Altered T Cell and B Cell Signaling in Rheumatoid Arthritis | 10.2 | 0.167 | #NUM! | SPP1,HLA-B,HLA-DQA1,HLA-DQB1,TLR2,IL18,HLA-DRB1,IL1RN,HLA-DMA,TNFSF13,HLA-DRA,FCER1G,CD86,IL1B,HLA-DRB5 |
| Role of NFAT in Regulation of the Immune Response | 10.1 | 0.104 | 4.359 | PLCB2,GRB2,FCGR2A,CD4,HLA-B,HLA-DQA1,HLA-DQB1,FCGR1A,GNAI2,CALM1 (includes others),NFKBID,HLA-DRB1,HLA-DMA,HLA-DRA,LYN,FCER1G,CD86,FCGR3A/FCGR3B,HLA-DRB5,FCGR1B |
| CD28 Signaling in T Helper Cells | 10 | 0.129 | 2.121 | PTPN6,ARPC1B,GRB2,CD4,HLA-B,HLA-DQA1,HLA-DQB1,PTPRC,CALM1 (includes others),NFKBID,HLA-DRB1,HLA-DMA,ARPC2,HLA-DRA,FCER1G,CD86,HLA-DRB5 |
| Antigen Presentation Pathway | 8.67 | 0.263 | #NUM! | HLA-DRB1,HLA-C,HLA-DMA,HLA-DRA,HLA-B,HLA-DQA1,HLA-DQB1,CD74,HLA-DPB1,HLA-DRB5 |
| Crosstalk between Dendritic Cells and Natural Killer Cells | 8.2 | 0.146 | #NUM! | IL18,HLA-DRB1,HLA-C,TYROBP,TREM2,HLA-B,HLA-DRA,CD86,CD83,TLN1,LTBR,TNFRSF1B,HLA-DRB5 |
| Nur77 Signaling in T Lymphocytes | 8.01 | 0.186 | #NUM! | CALM1 (includes others),HLA-DRB1,HLA-DMA,HLA-DRA,HLA-B,FCER1G,NR4A1,HLA-DQA1,CD86,HLA-DQB1,HLA-DRB5 |
| Th1 Pathway | 7.99 | 0.111 | 3.317 | GRB2,CD4,HAVCR2,HLA-B,HLA-DQA1,LGALS9,HLA-DQB1,ITGB2,IL18,HLA-DRB1,HLA-DMA,HLA-DRA,CD86,HLA-DPB1,HLA-DRB5 |
| Th1 and Th2 Activation Pathway | 7.91 | 0.0919 | #NUM! | GRB2,CD4,HAVCR2,HLA-B,HLA-DQA1,LGALS9,HLA-DQB1,SPI1,IL25,ITGB2,IL18,HLA-DRB1,HLA-DMA,HLA-DRA,CD86,HLA-DPB1,HLA-DRB5 |
| Autoimmune Thyroid Disease Signaling | 7.81 | 0.208 | #NUM! | HLA-DRB1,HLA-C,HLA-DMA,HLA-DRA,HLA-B,FCER1G,HLA-DQA1,CD86,HLA-DQB1,HLA-DRB5 |
| B Cell Development | 7.74 | 0.25 | #NUM! | PTPRC,HLA-DRB1,HLA-DMA,HLA-DRA,HLA-B,HLA-DQA1,CD86,HLA-DQB1,HLA-DRB5 |
| Calcium-induced T Lymphocyte Apoptosis | 7.65 | 0.167 | 3.162 | CALM1 (includes others),HLA-DRB1,HLA-DMA,CD4,HLA-DRA,HLA-B,FCER1G,NR4A1,HLA-DQA1,HLA-DQB1,HLA-DRB5 |
| Type I Diabetes Mellitus Signaling | 7.26 | 0.117 | 2 | HLA-B,HLA-DQA1,HLA-DQB1,NFKBID,HLA-DRB1,HLA-DMA,HLA-C,HLA-DRA,FCER1G,CD86,IL1B,TNFRSF1B,HLA-DRB5 |
| OX40 Signaling Pathway | 7.26 | 0.132 | #NUM! | NFKBID,HLA-DRB1,HLA-C,HLA-DMA,CD4,HLA-DRA,HLA-B,FCER1G,HLA-DQA1,HLA-DQB1,HLA-DPB1,HLA-DRB5 |
| T Helper Cell Differentiation | 7.25 | 0.151 | #NUM! | IL18,HLA-DRB1,HLA-DMA,HLA-DRA,HLA-B,FCER1G,HLA-DQA1,CD86,HLA-DQB1,TNFRSF1B,HLA-DRB5 |
| Fcγ Receptor-mediated Phagocytosis in Macrophages and Monocytes | 7.2 | 0.129 | 3.464 | HMOX1,RAC2,NCF1,ARPC1B,FCGR2A,ARPC2,LYN,HCK,TLN1,FYB1,FCGR1A,FCGR3A/FCGR3B |
| TREM1 Signaling | 7.17 | 0.147 | 3.317 | TLR2,TREM1,IL18,GRB2,TYROBP,LAT2,CD86,IL1B,CD83,CCL3,ITGAX |
| Production of Nitric Oxide and Reactive Oxygen Species in Macrophages | 6.97 | 0.0825 | 3.742 | APOE,PTPN6,GRB2,APOC2,NCF4,SPI1,TLR2,NFKBID,NCF1,CYBA,APOC1,S100A8,MAP3K8,SERPINA1,IRF8,TNFRSF1B |
| LXR/RXR Activation | 6.93 | 0.107 | -0.577 | APOE,LY96,IL18,C3,MSR1,IL1RN,APOC1,CD14,IL1B,SERPINA1,APOC2,S100A8,TNFRSF1B |
| iCOS-iCOSL Signaling in T Helper Cells | 6.87 | 0.106 | 3.162 | PTPRC,NFKBID,CALM1 (includes others),HLA-DRB1,GRB2,HLA-DMA,CD4,HLA-B,HLA-DRA,FCER1G,HLA-DQA1,HLA-DQB1,HLA-DRB5 |
| Th2 Pathway | 6.76 | 0.0933 | 1 | GRB2,CD4,HLA-B,HLA-DQA1,HLA-DQB1,SPI1,IL25,ITGB2,HLA-DRB1,HLA-DMA,HLA-DRA,CD86,HLA-DPB1,HLA-DRB5 |
| Allograft Rejection Signaling | 6.68 | 0.129 | #NUM! | HLA-DRB1,HLA-C,HLA-DMA,HLA-DRA,HLA-B,FCER1G,HLA-DQA1,CD86,HLA-DQB1,HLA-DPB1,HLA-DRB5 |
| Phagosome Formation | 6.59 | 0.0992 | #NUM! | PLCB2,MSR1,FCGR2A,GRB2,FCGR1A,TLR2,ITGB2,CLEC7A,FCER1G,C3AR1,FCGR3A/FCGR3B,FCGR1B,ITGAX |
| Neuroinflammation Signaling Pathway | 6.36 | 0.0611 | 3.771 | GRB2,TYROBP,PYCARD,HLA-B,HLA-DQA1,HLA-DQB1,CCL3,CSF1R,TLR2,HMOX1,IL18,HLA-DRB1,HLA-C,HLA-DMA,TREM2,HLA-DRA,CD86,IL1B,HLA-DRB5 |
| PKCθ Signaling in T Lymphocytes | 6.3 | 0.0848 | 3.742 | RAC2,GRB2,CD4,HLA-B,HLA-DQA1,HLA-DQB1,NFKBID,HLA-DRB1,HLA-DMA,HLA-DRA,FCER1G,CD86,MAP3K8,HLA-DRB5 |
| Cdc42 Signaling | 6.25 | 0.0838 | 2.236 | ARPC1B,MYL6,HLA-B,HLA-DQA1,HLA-DQB1,HLA-DRB1,HLA-C,HLA-DMA,ARPC2,HLA-DRA,FCER1G,HLA-DPB1,HLA-DRB5,MYL12A |
| Granulocyte Adhesion and Diapedesis | 5.83 | 0.0773 | #NUM! | CD99,CKLF,CCL3,CCL4L1/CCL4L2,FPR1,GNAI2,CXCL16,ITGB2,IL18,CCL4,CCL3L3,IL1RN,IL1B,TNFRSF1B |
| Complement System | 5.29 | 0.189 | 2 | ITGB2,C3,C1QA,C1QC,C1QB,C3AR1,ITGAX |
| Systemic Lupus Erythematosus Signaling | 5.27 | 0.0644 | #NUM! | PTPN6,FCGR2A,GRB2,HLA-B,FCGR1A,PTPRC,IL18,IL1RN,HLA-C,LYN,FCER1G,CD86,IL1B,FCGR3A/FCGR3B,FCGR1B |
| Phagosome Maturation | 5.18 | 0.0811 | #NUM! | CTSD,CTSL,HLA-DRB1,HLA-C,CTSH,CTSS,TCIRG1,HLA-DRA,HLA-B,CTSB,CTSC,HLA-DRB5 |
| Atherosclerosis Signaling | 5.01 | 0.0866 | #NUM! | APOE,ITGB2,IL18,MSR1,IL1RN,APOC1,IL1B,SERPINA1,APOC2,S100A8,ALOX5 |
| Agranulocyte Adhesion and Diapedesis | 4.76 | 0.0674 | #NUM! | GNAI2,CXCL16,ITGB2,CD99,IL18,CCL4,MYL6,IL1RN,CCL3L3,CKLF,IL1B,CCL3,CCL4L1/CCL4L2 |
| Role of Pattern Recognition Receptors in Recognition of Bacteria and Viruses | 4.71 | 0.0803 | 2.646 | TLR2,CLEC7A,IL18,C3,GRB2,IL1B,C1QA,C1QC,C1QB,C3AR1,IL25 |
| IL-12 Signaling and Production in Macrophages | 4.45 | 0.0753 | #NUM! | TLR2,APOE,IL18,GRB2,APOC1,SERPINA1,MAP3K8,APOC2,S100A8,IRF8,SPI1 |
| Acute Phase Response Signaling | 4.45 | 0.0682 | 2.714 | HAMP,NFKBID,HMOX1,FTL,IL18,C3,GRB2,IL1RN,IL1B,SERPINA1,TNFRSF1B,A2M |
| IL-4 Signaling | 4.4 | 0.0947 | #NUM! | PTPN6,HLA-DRB1,GRB2,HLA-DMA,HLA-DRA,HLA-B,HLA-DQA1,HLA-DQB1,HLA-DRB5 |
| Leukocyte Extravasation Signaling | 4.39 | 0.0616 | 1.732 | CD99,RAC2,MYL6,GRB2,ARHGAP4,NCF4,GNAI2,TEC,ITGB2,NCF1,ARHGAP9,TIMP1,CYBA |
| Natural Killer Cell Signaling | 4.2 | 0.0781 | #NUM! | RAC2,PTPN6,LAIR1,FCGR2A,GRB2,TYROBP,FCER1G,HCST,FCGR3A/FCGR3B,CD300A |
| LPS/IL-1 Mediated Inhibition of RXR Function | 4.18 | 0.0586 | 0.816 | APOE,IL18,LY96,MGST2,IL1RN,APOC1,CD14,IL1B,FABP5,APOC2,TNFRSF1B,ACSL1,GSTO1 |
| IL-10 Signaling | 3.57 | 0.101 | #NUM! | NFKBID,HMOX1,IL18,FCGR2A,IL1RN,CD14,IL1B |
| FXR/RXR Activation | 3.46 | 0.0714 | #NUM! | APOE,IL18,C3,IL1RN,APOC1,FBP1,IL1B,SERPINA1,APOC2 |
| fMLP Signaling in Neutrophils | 3.39 | 0.0698 | 2.121 | GNAI2,NFKBID,CALM1 (includes others),NCF1,PLCB2,ARPC1B,GRB2,ARPC2,FPR1 |
| B Cell Receptor Signaling | 3.39 | 0.0567 | 2.333 | PTPRC,NFKBID,RAC2,CALM1 (includes others),PTPN6,APBB1IP,FCGR2A,GRB2,LYN,MAP3K8,BCL2A1 |
| Inflammasome pathway | 3.06 | 0.2 | 2 | IL18,PYCARD,CTSB,IL1B |
| autophagy | 2.94 | 0.0968 | #NUM! | CTSD,CTSL,CTSH,CTSS,CTSB,CTSC |
| Regulation of Actin-based Motility by Rho | 2.89 | 0.0778 | 2.449 | RAC2,PFN1,ARPC1B,MYL6,ARPC2,GSN,MYL12A |
| NF-κB Signaling | 2.88 | 0.0535 | 2.53 | TLR2,NFKBID,IL18,GRB2,IL1RN,FCER1G,IL1B,MAP3K8,LTBR,TNFRSF1B |
| Role of Hypercytokinemia/hyperchemokinemia in the Pathogenesis of Influenza | 2.79 | 0.116 | #NUM! | IL18,CCL4,IL1RN,IL1B,CCL3 |
| Actin Cytoskeleton Signaling | 2.74 | 0.0472 | 2.53 | RAC2,PFN1,ARPC1B,MYL6,GRB2,ARPC2,CD14,TLN1,TMSB10/TMSB4X,GSN,MYL12A |
| CTLA4 Signaling in Cytotoxic T Lymphocytes | 2.67 | 0.0707 | #NUM! | PTPN6,HLA-C,GRB2,HLA-B,FCER1G,CD86,AP1B1 |
| Role of Macrophages, Fibroblasts and Endothelial Cells in Rheumatoid Arthritis | 2.67 | 0.0406 | #NUM! | TLR2,NFKBID,CALM1 (includes others),IL18,PLCB2,GRB2,IL1RN,CEBPD,IL1B,LTBR,TNFRSF1B,FCGR1A,FCGR3A/FCGR3B |
| IL-6 Signaling | 2.6 | 0.0597 | 2.646 | NFKBID,IL18,GRB2,IL1RN,CD14,IL1B,TNFRSF1B,A2M |
| Clathrin-mediated Endocytosis Signaling | 2.58 | 0.0483 | #NUM! | APOE,ITGB2,ARPC1B,GRB2,ARPC2,APOC1,SERPINA1,APOC2,S100A8,AP1B1 |
| Toll-like Receptor Signaling | 2.53 | 0.0789 | 2.449 | TLR2,LY96,IL18,IL1RN,CD14,IL1B |
| Leukotriene Biosynthesis | 2.46 | 0.231 | #NUM! | MGST2,LTC4S,ALOX5 |
| Integrin Signaling | 2.41 | 0.0457 | 3 | RAC2,ITGB2,PFN1,ARPC1B,GRB2,ARPC2,TLN1,GSN,ITGAX,MYL12A |
| Role of Osteoblasts, Osteoclasts and Chondrocytes in Rheumatoid Arthritis | 2.21 | 0.0429 | #NUM! | NFKBID,CALM1 (includes others),IL18,SPP1,GRB2,IL1RN,IL1B,GSN,TNFRSF1B,CSF1R |
| MIF-mediated Glucocorticoid Regulation | 2.2 | 0.114 | 2 | NFKBID,LY96,CD14,CD74 |
| RhoA Signaling | 2.16 | 0.0565 | 1.134 | PFN1,ARHGAP9,ARPC1B,MYL6,ARPC2,ARHGAP4,MYL12A |
| Hepatic Cholestasis | 2.16 | 0.05 | #NUM! | NFKBID,LY96,IL18,IL1RN,CD14,IL1B,TNFRSF1B,IL25 |
| Differential Regulation of Cytokine Production in Macrophages and T Helper Cells by IL-17A and IL-17F | 2.08 | 0.167 | #NUM! | CCL4,IL1B,CCL3 |
| CCR5 Signaling in Macrophages | 2.08 | 0.0632 | 1 | GNAI2,CALM1 (includes others),CCL4,CD4,FCER1G,CCL3 |
| PI3K Signaling in B Lymphocytes | 1.95 | 0.0515 | 2.449 | CD81,PTPRC,NFKBID,CALM1 (includes others),PLCB2,C3,LYN |
| PPAR Signaling | 1.95 | 0.0594 | -2.449 | NFKBID,IL18,GRB2,IL1RN,IL1B,TNFRSF1B |
| Iron homeostasis signaling pathway | 1.94 | 0.0511 | #NUM! | HAMP,HMOX1,FTL,TCIRG1,CD163,SLC11A1,FTH1 |
| MIF Regulation of Innate Immunity | 1.93 | 0.093 | 2 | NFKBID,LY96,CD14,CD74 |
| RhoGDI Signaling | 1.93 | 0.0452 | -1.89 | GNAI2,ARHGAP9,ARPC1B,MYL6,ARPC2,ARHGAP4,ARHGDIB,MYL12A |
| iNOS Signaling | 1.86 | 0.0889 | #NUM! | NFKBID,CALM1 (includes others),LY96,CD14 |
| Differential Regulation of Cytokine Production in Intestinal Epithelial Cells by IL-17A and IL-17F | 1.81 | 0.13 | #NUM! | CCL4,IL1B,CCL3 |
| Virus Entry via Endocytic Pathways | 1.69 | 0.0517 | #NUM! | RAC2,ITGB2,HLA-C,GRB2,HLA-B,AP1B1 |
| Salvage Pathways of Pyrimidine Deoxyribonucleotides | 1.67 | 0.25 | #NUM! | TYMP,TK1 |
| Phospholipase C Signaling | 1.63 | 0.0369 | 2.646 | HMOX1,CALM1 (includes others),PLCB2,MYL6,FCGR2A,GRB2,LYN,FCER1G,MYL12A |
| Role of Cytokines in Mediating Communication between Immune Cells | 1.61 | 0.0741 | #NUM! | IL18,IL1RN,IL1B,IL25 |
| IL-8 Signaling | 1.61 | 0.0394 | 2.828 | GNAI2,HMOX1,RAC2,ITGB2,PLCB2,GRB2,HBEGF,ITGAX |
| Opioid Signaling Pathway | 1.61 | 0.0364 | 1 | GNAI2,RAC2,RGS1,CALM1 (includes others),RGS19,RGS10,LYN,HCK,AP1B1 |
| Pathogenesis of Multiple Sclerosis | 1.6 | 0.222 | #NUM! | CCL4,CCL3 |
| Osteoarthritis Pathway | 1.52 | 0.0377 | 2.236 | TLR2,SPP1,S100A9,IL1B,ANXA2,S100A8,HTRA1,TNFRSF1B |
| Reelin Signaling in Neurons | 1.51 | 0.0543 | #NUM! | APOE,ITGB2,GRB2,LYN,HCK |
| Role of Tissue Factor in Cancer | 1.51 | 0.0462 | #NUM! | GRB2,LYN,HCK,PLAUR,IL1B,HBEGF |
| CXCR4 Signaling | 1.51 | 0.0409 | 2.646 | GNAI2,PLCB2,MYL6,GRB2,CD4,LYN,MYL12A |
| Cytotoxic T Lymphocyte-mediated Apoptosis of Target Cells | 1.47 | 0.0938 | #NUM! | HLA-C,HLA-B,FCER1G |
| GP6 Signaling Pathway | 1.46 | 0.0448 | 2.449 | CALM1 (includes others),APBB1IP,GRB2,LYN,FCER1G,TLN1 |
| Coagulation System | 1.37 | 0.0857 | #NUM! | PLAUR,SERPINA1,A2M |
| RANK Signaling in Osteoclasts | 1.36 | 0.049 | 2 | NFKBID,CALM1 (includes others),GRB2,MAP3K8,GSN |
| Eicosanoid Signaling | 1.34 | 0.0597 | #NUM! | LTC4S,ALOX5AP,TBXAS1,ALOX5 |
| Hepatic Fibrosis / Hepatic Stellate Cell Activation | 1.34 | 0.0374 | #NUM! | LY96,MYL6,TIMP1,CD14,IL1B,TNFRSF1B,A2M |
| Role of IL-17A in Psoriasis | 1.33 | 0.154 | #NUM! | S100A9,S100A8 |
| Cholecystokinin/Gastrin-mediated Signaling | 1.29 | 0.0467 | 2.236 | PLCB2,IL18,GRB2,IL1RN,IL1B |
| Antioxidant Action of Vitamin C | 1.28 | 0.0463 | #NUM! | SLC2A5,NFKBID,HMOX1,PLCB2,GSTO1 |
| Caveolar-mediated Endocytosis Signaling | 1.28 | 0.0563 | #NUM! | ITGB2,HLA-C,HLA-B,ITGAX |
| Glucocorticoid Receptor Signaling | 1.23 | 0.029 | #NUM! | NFKBID,GRB2,SGK1,IL1RN,ANXA1,IL1B,CD163,CCL3,FCGR1A,A2M |
| Adrenomedullin signaling pathway | 1.22 | 0.035 | 1.89 | CALM1 (includes others),PLCB2,IL18,C3,GRB2,IL1RN,IL1B |
| T Cell Receptor Signaling | 1.19 | 0.0435 | #NUM! | PTPRC,TEC,CALM1 (includes others),GRB2,CD4 |
| Chemokine Signaling | 1.18 | 0.0519 | 2 | GNAI2,CALM1 (includes others),PLCB2,CCL4 |
| GM-CSF Signaling | 1.15 | 0.0506 | 2 | GRB2,LYN,HCK,BCL2A1 |
| Gαi Signaling | 1.09 | 0.0407 | 1.342 | GNAI2,GRB2,RGS10,ADORA3,FPR1 |
| Tec Kinase Signaling | 1.07 | 0.0353 | 2.236 | TEC,GNAI2,GRB2,LYN,FCER1G,HCK |
| GNRH Signaling | 1.06 | 0.0351 | 2.236 | GNAI2,CALM1 (includes others),PLCB2,GRB2,HBEGF,MAP3K8 |
| Macropinocytosis Signaling | 1.03 | 0.046 | #NUM! | ITGB2,GRB2,CD14,CSF1R |
| Docosahexaenoic Acid (DHA) Signaling | 0.989 | 0.0577 | #NUM! | GRB2,IL1B,BCL2A1 |
| G-Protein Coupled Receptor Signaling | 0.96 | 0.0284 | #NUM! | GNAI2,NFKBID,PLCB2,GRB2,RGS10,ADORA3,MAP3K8,FPR1 |
| NF-κB Activation by Viruses | 0.952 | 0.043 | 2 | NFKBID,ITGB2,GRB2,CD4 |
| HMGB1 Signaling | 0.927 | 0.036 | 2.236 | IL18,GRB2,IL1B,TNFRSF1B,IL25 |
| Gα12/13 Signaling | 0.913 | 0.0355 | 2 | TEC,NFKBID,MYL6,GRB2,MYL12A |
| Thyroid Hormone Biosynthesis | 0.913 | 0.333 | #NUM! | CTSD |
| Tumoricidal Function of Hepatic Natural Killer Cells | 0.913 | 0.0833 | #NUM! | SERPINB9,SRGN |
| Glutathione Redox Reactions I | 0.913 | 0.0833 | #NUM! | MGST2,GPX1 |
| Cardiac Hypertrophy Signaling | 0.905 | 0.029 | 1.89 | GNAI2,CALM1 (includes others),PLCB2,MYL6,GRB2,MAP3K8,MYL12A |
| Lipid Antigen Presentation by CD1 | 0.858 | 0.0769 | #NUM! | FCER1G,AP1B1 |
| NRF2-mediated Oxidative Stress Response | 0.841 | 0.0302 | #NUM! | HMOX1,FTL,MGST2,GRB2,GSTO1,FTH1 |
| Actin Nucleation by ARP-WASP Complex | 0.841 | 0.0484 | #NUM! | ARPC1B,GRB2,ARPC2 |
| Signaling by Rho Family GTPases | 0.834 | 0.0278 | 2.449 | GNAI2,ARPC1B,MYL6,GRB2,ARPC2,VIM,MYL12A |
| Arsenate Detoxification I (Glutaredoxin) | 0.833 | 0.25 | #NUM! | GSTO1 |
| Heme Degradation | 0.833 | 0.25 | #NUM! | HMOX1 |
| Spermine and Spermidine Degradation I | 0.833 | 0.25 | #NUM! | SAT1 |
| Ascorbate Recycling (Cytosolic) | 0.833 | 0.25 | #NUM! | GSTO1 |
| Acetate Conversion to Acetyl-CoA | 0.833 | 0.25 | #NUM! | ACSL1 |
| Lymphotoxin β Receptor Signaling | 0.785 | 0.0448 | #NUM! | NFKBID,GRB2,LTBR |
| TNFR2 Signaling | 0.783 | 0.0667 | #NUM! | NFKBID,TNFRSF1B |
| Gαq Signaling | 0.767 | 0.0311 | 2 | NFKBID,HMOX1,CALM1 (includes others),PLCB2,GRB2 |
| Glutathione-mediated Detoxification | 0.765 | 0.0645 | #NUM! | MGST2,GSTO1 |
| Melatonin Signaling | 0.728 | 0.0417 | #NUM! | GNAI2,CALM1 (includes others),PLCB2 |
| MSP-RON Signaling Pathway | 0.728 | 0.0417 | #NUM! | TLR2,ITGB2,GRB2 |
| Paxillin Signaling | 0.716 | 0.0336 | #NUM! | ITGB2,GRB2,TLN1,ITGAX |
| DNA Methylation and Transcriptional Repression Signaling | 0.716 | 0.0588 | #NUM! | HIST4H4,SAP30 |
| Role of JAK2 in Hormone-like Cytokine Signaling | 0.716 | 0.0588 | #NUM! | PTPN6,SH2B3 |
| Neuroprotective Role of THOP1 in Alzheimer's Disease | 0.716 | 0.0333 | #NUM! | HLA-C,HLA-B,ST14,HTRA1 |
| p38 MAPK Signaling | 0.716 | 0.0333 | 2 | IL18,IL1RN,IL1B,TNFRSF1B |
| IL-17A Signaling in Fibroblasts | 0.7 | 0.0571 | #NUM! | NFKBID,CEBPD |
| Glioma Invasiveness Signaling | 0.696 | 0.0395 | #NUM! | TIMP1,GRB2,PLAUR |
| Axonal Guidance Signaling | 0.689 | 0.0219 | #NUM! | GNAI2,RAC2,PLCB2,PFN1,ARPC1B,MYL6,ADAM28,GRB2,ARPC2,MYL12A |
| cAMP-mediated signaling | 0.689 | 0.0263 | 1.342 | GNAI2,CALM1 (includes others),AKAP13,RGS10,ADORA3,FPR1 |
| Fc Epsilon RI Signaling | 0.686 | 0.032 | 2 | RAC2,GRB2,LYN,FCER1G |
| VDR/RXR Activation | 0.686 | 0.0385 | #NUM! | SERPINB1,SPP1,CD14 |
| Protein Kinase A Signaling | 0.686 | 0.0224 | -0.816 | PTPRC,GNAI2,NFKBID,CALM1 (includes others),AKAP13,PTPN6,PLCB2,MYL6,MYL12A |
| Role of PI3K/AKT Signaling in the Pathogenesis of Influenza | 0.676 | 0.038 | #NUM! | GNAI2,NFKBID,GRB2 |
| Germ Cell-Sertoli Cell Junction Signaling | 0.674 | 0.0279 | #NUM! | RAC2,GRB2,MAP3K8,GSN,A2M |
| Ephrin Receptor Signaling | 0.674 | 0.0279 | 2.236 | GNAI2,RAC2,ARPC1B,GRB2,ARPC2 |
| Superpathway of Inositol Phosphate Compounds | 0.657 | 0.0254 | 2.449 | PTPRC,PTPN6,PLCB2,GRB2,DUSP23,CD86 |
| April Mediated Signaling | 0.657 | 0.0513 | #NUM! | NFKBID,TNFSF13 |
| Inhibition of Matrix Metalloproteases | 0.657 | 0.0513 | #NUM! | TIMP1,A2M |
| Cellular Effects of Sildenafil (Viagra) | 0.653 | 0.0305 | #NUM! | CALM1 (includes others),PLCB2,MYL6,MYL12A |
| Xenobiotic Metabolism Signaling | 0.648 | 0.0236 | #NUM! | HMOX1,FTL,MGST2,GRB2,IL1B,MAP3K8,GSTO1 |
| Role of PKR in Interferon Induction and Antiviral Response | 0.631 | 0.0488 | #NUM! | NFKBID,FCGR1A |
| Growth Hormone Signaling | 0.631 | 0.0353 | #NUM! | PTPN6,GRB2,A2M |
| Regulation of IL-2 Expression in Activated and Anergic T Lymphocytes | 0.624 | 0.0349 | #NUM! | NFKBID,CALM1 (includes others),GRB2 |
| CCR3 Signaling in Eosinophils | 0.624 | 0.0294 | #NUM! | GNAI2,CALM1 (includes others),PLCB2,GRB2 |
| Erythropoietin Signaling | 0.617 | 0.0345 | #NUM! | NFKBID,PTPN6,GRB2 |
| p70S6K Signaling | 0.612 | 0.029 | 2 | GNAI2,PLCB2,GRB2,LYN |
| Prostanoid Biosynthesis | 0.601 | 0.111 | #NUM! | TBXAS1 |
| Stearate Biosynthesis I (Animals) | 0.599 | 0.0455 | #NUM! | TBXAS1,ACSL1 |
| Aryl Hydrocarbon Receptor Signaling | 0.596 | 0.0284 | #NUM! | CTSD,MGST2,IL1B,GSTO1 |
| ILK Signaling | 0.587 | 0.0254 | 2 | ITGB2,MYL6,GRB2,VIM,TMSB10/TMSB4X |
| Role of IL-17F in Allergic Inflammatory Airway Diseases | 0.576 | 0.0435 | #NUM! | CCL4,IL1B |
| LPS-stimulated MAPK Signaling | 0.576 | 0.0323 | #NUM! | NFKBID,GRB2,CD14 |
| Death Receptor Signaling | 0.576 | 0.0323 | #NUM! | NFKBID,TNFRSF1B,ARHGDIB |
| 3-phosphoinositide Biosynthesis | 0.571 | 0.0249 | 2.236 | PTPRC,PTPN6,GRB2,DUSP23,CD86 |
| Hematopoiesis from Pluripotent Stem Cells | 0.556 | 0.0417 | #NUM! | CD4,FCER1G |
| Apoptosis Signaling | 0.554 | 0.0312 | #NUM! | NFKBID,BCL2A1,TNFRSF1B |
| Primary Immunodeficiency Signaling | 0.547 | 0.0408 | #NUM! | PTPRC,CD4 |
| TR/RXR Activation | 0.541 | 0.0306 | #NUM! | SLC16A3,UCP2,GRB2 |
| Ceramide Signaling | 0.536 | 0.0303 | #NUM! | CTSD,GRB2,TNFRSF1B |
| Acute Myeloid Leukemia Signaling | 0.536 | 0.0303 | #NUM! | GRB2,CSF1R,SPI1 |
| Thrombin Signaling | 0.533 | 0.0238 | #NUM! | GNAI2,PLCB2,MYL6,GRB2,MYL12A |
| Type II Diabetes Mellitus Signaling | 0.531 | 0.026 | #NUM! | NFKBID,GRB2,TNFRSF1B,ACSL1 |
| CD27 Signaling in Lymphocytes | 0.508 | 0.0377 | #NUM! | NFKBID,MAP3K8 |
| Fatty Acid Activation | 0.503 | 0.0769 | #NUM! | ACSL1 |
| Choline Biosynthesis III | 0.503 | 0.0769 | #NUM! | HMOX1 |
| Glycogen Degradation II | 0.503 | 0.0769 | #NUM! | TYMP |
| D-myo-inositol-5-phosphate Metabolism | 0.488 | 0.0247 | 2 | PTPRC,PTPN6,PLCB2,DUSP23 |
| PAK Signaling | 0.486 | 0.028 | #NUM! | MYL6,GRB2,MYL12A |
| Oxidative Phosphorylation | 0.472 | 0.0275 | #NUM! | COX6B1,ATP5ME,ATP5F1E |
| SAPK/JNK Signaling | 0.467 | 0.0273 | #NUM! | RAC2,GRB2,FCER1G |
| Glycogen Degradation III | 0.459 | 0.0667 | #NUM! | TYMP |
| Mitochondrial Dysfunction | 0.443 | 0.0234 | #NUM! | COX6B1,UCP2,ATP5ME,ATP5F1E |
| Vitamin-C Transport | 0.441 | 0.0625 | #NUM! | GSTO1 |
| Induction of Apoptosis by HIV1 | 0.441 | 0.0328 | #NUM! | NFKBID,TNFRSF1B |
| Phospholipases | 0.433 | 0.0323 | #NUM! | HMOX1,PLCB2 |
| Regulation of Cellular Mechanics by Calpain Protease | 0.428 | 0.0317 | #NUM! | GRB2,TLN1 |
| γ-linolenate Biosynthesis II (Animals) | 0.428 | 0.0588 | #NUM! | ACSL1 |
| Mitochondrial L-carnitine Shuttle Pathway | 0.428 | 0.0588 | #NUM! | ACSL1 |
| Pancreatic Adenocarcinoma Signaling | 0.416 | 0.025 | #NUM! | HMOX1,GRB2,HBEGF |
| G Beta Gamma Signaling | 0.416 | 0.025 | #NUM! | GNAI2,GRB2,HBEGF |
| Pyridoxal 5'-phosphate Salvage Pathway | 0.416 | 0.0308 | #NUM! | SGK1,MAP3K8 |
| Rac Signaling | 0.4 | 0.0244 | #NUM! | ARPC1B,GRB2,ARPC2 |
| Sphingosine-1-phosphate Signaling | 0.39 | 0.024 | #NUM! | GNAI2,PLCB2,GRB2 |
| PPARα/RXRα Activation | 0.39 | 0.0215 | #NUM! | NFKBID,PLCB2,GRB2,IL1B |
| Role of IL-17A in Arthritis | 0.39 | 0.029 | #NUM! | NFKBID,GRB2 |
| Remodeling of Epithelial Adherens Junctions | 0.39 | 0.029 | #NUM! | ARPC1B,ARPC2 |
| Huntington's Disease Signaling | 0.387 | 0.02 | #NUM! | CTSD,PLCB2,GRB2,SGK1,ATP5F1E |
| Sperm Motility | 0.387 | 0.0236 | #NUM! | SLC16A10,CALM1 (includes others),PLCB2 |
| Oxidative Ethanol Degradation III | 0.372 | 0.0476 | #NUM! | ACSL1 |
| ERK5 Signaling | 0.372 | 0.0278 | #NUM! | SGK1,MAP3K8 |
| PI3K/AKT Signaling | 0.37 | 0.0229 | #NUM! | NFKBID,GRB2,MAP3K8 |
| Ephrin B Signaling | 0.37 | 0.0274 | #NUM! | GNAI2,RAC2 |
| GPCR-Mediated Integration of Enteroendocrine Signaling Exemplified by an L Cell | 0.37 | 0.0274 | #NUM! | GNAI2,PLCB2 |
| Polyamine Regulation in Colon Cancer | 0.366 | 0.0455 | #NUM! | SAT1 |
| Endothelin-1 Signaling | 0.36 | 0.0204 | 2 | GNAI2,HMOX1,PLCB2,GRB2 |
| Agrin Interactions at Neuromuscular Junction | 0.36 | 0.0267 | #NUM! | RAC2,ITGB2 |
| Putrescine Degradation III | 0.356 | 0.0435 | #NUM! | SAT1 |
| 14-3-3-mediated Signaling | 0.348 | 0.0219 | #NUM! | PLCB2,GRB2,VIM |
| Role of JAK1, JAK2 and TYK2 in Interferon Signaling | 0.344 | 0.0417 | #NUM! | PTPN6 |
| IL-17A Signaling in Airway Cells | 0.344 | 0.0256 | #NUM! | NFKBID,GRB2 |
| Corticotropin Releasing Hormone Signaling | 0.343 | 0.0216 | #NUM! | GNAI2,CALM1 (includes others),NR4A1 |
| CD40 Signaling | 0.341 | 0.0253 | #NUM! | NFKBID,GRB2 |
| P2Y Purigenic Receptor Signaling Pathway | 0.341 | 0.0214 | #NUM! | GNAI2,PLCB2,GRB2 |
| Ethanol Degradation IV | 0.34 | 0.04 | #NUM! | ACSL1 |
| Antiproliferative Role of Somatostatin Receptor 2 | 0.336 | 0.0247 | #NUM! | PTPN6,GRB2 |
| mTOR Signaling | 0.336 | 0.0193 | #NUM! | HMOX1,GRB2,RPS27,RPS18 |
| Glycolysis I | 0.336 | 0.0385 | #NUM! | FBP1 |
| Gluconeogenesis I | 0.336 | 0.0385 | #NUM! | FBP1 |
| GDNF Family Ligand-Receptor Interactions | 0.336 | 0.0244 | #NUM! | GRB2,DOK3 |
| D-myo-inositol (1,4,5,6)-Tetrakisphosphate Biosynthesis | 0.336 | 0.0208 | #NUM! | PTPRC,PTPN6,DUSP23 |
| D-myo-inositol (3,4,5,6)-tetrakisphosphate Biosynthesis | 0.336 | 0.0208 | #NUM! | PTPRC,PTPN6,DUSP23 |
| D-myo-inositol (1,4,5)-Trisphosphate Biosynthesis | 0.329 | 0.037 | #NUM! | PLCB2 |
| Breast Cancer Regulation by Stathmin1 | 0.329 | 0.019 | #NUM! | GNAI2,CALM1 (includes others),PLCB2,GRB2 |
| Angiopoietin Signaling | 0.329 | 0.0238 | #NUM! | NFKBID,GRB2 |
| FcγRIIB Signaling in B Lymphocytes | 0.326 | 0.0235 | #NUM! | GRB2,LYN |
| Small Cell Lung Cancer Signaling | 0.326 | 0.0235 | #NUM! | NFKBID,GRB2 |
| Leptin Signaling in Obesity | 0.321 | 0.0233 | #NUM! | PLCB2,GRB2 |
| Epithelial Adherens Junction Signaling | 0.319 | 0.02 | #NUM! | ARPC1B,MYL6,ARPC2 |
| CREB Signaling in Neurons | 0.312 | 0.0183 | 2 | GNAI2,CALM1 (includes others),PLCB2,GRB2 |
| IL-3 Signaling | 0.309 | 0.0225 | #NUM! | PTPN6,GRB2 |
| JAK/Stat Signaling | 0.309 | 0.0225 | #NUM! | PTPN6,GRB2 |
| IL-17 Signaling | 0.307 | 0.022 | #NUM! | TIMP1,GRB2 |
| FGF Signaling | 0.307 | 0.022 | #NUM! | PTPN6,GRB2 |
| IL-7 Signaling Pathway | 0.307 | 0.022 | #NUM! | GRB2,LYN |
| IL-1 Signaling | 0.307 | 0.0217 | #NUM! | GNAI2,NFKBID |
| Role of NFAT in Cardiac Hypertrophy | 0.307 | 0.0178 | 1 | GNAI2,CALM1 (includes others),PLCB2,GRB2 |
| Relaxin Signaling | 0.307 | 0.019 | #NUM! | GNAI2,NFKBID,GRB2 |
| 3-phosphoinositide Degradation | 0.307 | 0.019 | #NUM! | PTPRC,PTPN6,DUSP23 |
| 4-1BB Signaling in T Lymphocytes | 0.307 | 0.0312 | #NUM! | NFKBID |
| G Protein Signaling Mediated by Tubby | 0.307 | 0.0312 | #NUM! | PLCB2 |
| Fatty Acid β-oxidation I | 0.307 | 0.0312 | #NUM! | ACSL1 |
| α-Adrenergic Signaling | 0.307 | 0.0215 | #NUM! | GNAI2,CALM1 (includes others) |
| PEDF Signaling | 0.307 | 0.0215 | #NUM! | NFKBID,GRB2 |
| Neuregulin Signaling | 0.305 | 0.0213 | #NUM! | GRB2,HBEGF |
| HER-2 Signaling in Breast Cancer | 0.305 | 0.0213 | #NUM! | ITGB2,GRB2 |
| Regulation of eIF4 and p70S6K Signaling | 0.293 | 0.0184 | #NUM! | GRB2,RPS27,RPS18 |
| Salvage Pathways of Pyrimidine Ribonucleotides | 0.292 | 0.0206 | #NUM! | SGK1,MAP3K8 |
| Dopamine-DARPP32 Feedback in cAMP Signaling | 0.292 | 0.0183 | #NUM! | GNAI2,CALM1 (includes others),PLCB2 |
| TWEAK Signaling | 0.287 | 0.0286 | #NUM! | NFKBID |
| Interferon Signaling | 0.281 | 0.0278 | #NUM! | IFITM2 |
| Aldosterone Signaling in Epithelial Cells | 0.281 | 0.0179 | #NUM! | PLCB2,GRB2,SGK1 |
| Ethanol Degradation II | 0.274 | 0.027 | #NUM! | ACSL1 |
| Prostate Cancer Signaling | 0.271 | 0.0194 | #NUM! | NFKBID,GRB2 |
| STAT3 Pathway | 0.271 | 0.0194 | #NUM! | PTPN6,IL1B |
| Melanocyte Development and Pigmentation Signaling | 0.27 | 0.0192 | #NUM! | PTPN6,GRB2 |
| ErbB Signaling | 0.27 | 0.0192 | #NUM! | GRB2,HBEGF |
| FAK Signaling | 0.266 | 0.019 | #NUM! | GRB2,TLN1 |
| Oncostatin M Signaling | 0.258 | 0.025 | #NUM! | GRB2 |
| B Cell Activating Factor Signaling | 0.254 | 0.0244 | #NUM! | NFKBID |
| nNOS Signaling in Skeletal Muscle Cells | 0.254 | 0.0244 | #NUM! | CALM1 (includes others) |
| VEGF Signaling | 0.254 | 0.0183 | #NUM! | PTPN6,GRB2 |
| Synaptic Long Term Depression | 0.254 | 0.0167 | #NUM! | GNAI2,PLCB2,LYN |
| Amyotrophic Lateral Sclerosis Signaling | 0.248 | 0.018 | #NUM! | GRB2,GPX1 |
| UVA-Induced MAPK Signaling | 0.248 | 0.0179 | #NUM! | PLCB2,GRB2 |
| GPCR-Mediated Nutrient Sensing in Enteroendocrine Cells | 0.248 | 0.0179 | #NUM! | GNAI2,PLCB2 |
| Role of p14/p19ARF in Tumor Suppression | 0.248 | 0.0233 | #NUM! | GRB2 |
| Nitric Oxide Signaling in the Cardiovascular System | 0.246 | 0.0177 | #NUM! | CALM1 (includes others),GRB2 |
| Role of RIG1-like Receptors in Antiviral Innate Immunity | 0.244 | 0.0227 | #NUM! | NFKBID |
| Neuropathic Pain Signaling In Dorsal Horn Neurons | 0.242 | 0.0174 | #NUM! | PLCB2,GRB2 |
| IL-9 Signaling | 0.242 | 0.0222 | #NUM! | GRB2 |
| Pyrimidine Ribonucleotides Interconversion | 0.242 | 0.0222 | #NUM! | ANXA1 |
| Role of Oct4 in Mammalian Embryonic Stem Cell Pluripotency | 0.236 | 0.0217 | #NUM! | SPP1 |
| nNOS Signaling in Neurons | 0.233 | 0.0213 | #NUM! | CALM1 (includes others) |
| Pyrimidine Ribonucleotides De Novo Biosynthesis | 0.233 | 0.0213 | #NUM! | ANXA1 |
| HGF Signaling | 0.233 | 0.0168 | #NUM! | GRB2,MAP3K8 |
| Glioma Signaling | 0.231 | 0.0167 | #NUM! | CALM1 (includes others),GRB2 |
| TNFR1 Signaling | 0.219 | 0.02 | #NUM! | NFKBID |
| HIF1α Signaling | 0.219 | 0.0161 | #NUM! | SLC2A5,GRB2 |
| NGF Signaling | 0.218 | 0.016 | #NUM! | GRB2,MAP3K8 |
| PTEN Signaling | 0.218 | 0.016 | #NUM! | RAC2,GRB2 |
| Gap Junction Signaling | 0.217 | 0.0149 | #NUM! | GNAI2,PLCB2,GRB2 |
| Synaptic Long Term Potentiation | 0.217 | 0.0159 | #NUM! | CALM1 (includes others),PLCB2 |
| Renin-Angiotensin Signaling | 0.213 | 0.0156 | #NUM! | PTPN6,GRB2 |
| ERK/MAPK Signaling | 0.213 | 0.0147 | #NUM! | RAC2,GRB2,TLN1 |
| Calcium Signaling | 0.209 | 0.0146 | #NUM! | CALM1 (includes others),MYL6,TPM3 |
| Transcriptional Regulatory Network in Embryonic Stem Cells | 0.209 | 0.0185 | #NUM! | HIST4H4 |
| Unfolded protein response | 0.2 | 0.0179 | #NUM! | CEBPD |
| Adipogenesis pathway | 0.199 | 0.0149 | #NUM! | CEBPD,SAP30 |
| Glutamate Receptor Signaling | 0.198 | 0.0175 | #NUM! | CALM1 (includes others) |
| Androgen Signaling | 0.192 | 0.0146 | #NUM! | GNAI2,CALM1 (includes others) |
| Ephrin A Signaling | 0.186 | 0.0167 | #NUM! | GRB2 |
| Melanoma Signaling | 0.185 | 0.0164 | #NUM! | GRB2 |
| PCP pathway | 0.185 | 0.0164 | #NUM! | PFN1 |
| Activation of IRF by Cytosolic Pattern Recognition Receptors | 0.179 | 0.0159 | #NUM! | NFKBID |
| Wnt/Ca+ pathway | 0.179 | 0.0159 | #NUM! | PLCB2 |
| EIF2 Signaling | 0.174 | 0.0132 | #NUM! | GRB2,RPS27,RPS18 |
| Netrin Signaling | 0.174 | 0.0154 | #NUM! | RAC2 |
| Insulin Receptor Signaling | 0.174 | 0.0136 | #NUM! | GRB2,SGK1 |
| UVB-Induced MAPK Signaling | 0.172 | 0.0152 | #NUM! | GRB2 |
| EGF Signaling | 0.165 | 0.0147 | #NUM! | GRB2 |
| CNTF Signaling | 0.162 | 0.0143 | #NUM! | GRB2 |
| Endometrial Cancer Signaling | 0.162 | 0.0143 | #NUM! | GRB2 |
| IL-2 Signaling | 0.162 | 0.0143 | #NUM! | GRB2 |
| Thrombopoietin Signaling | 0.16 | 0.0141 | #NUM! | GRB2 |
| ErbB2-ErbB3 Signaling | 0.148 | 0.0133 | #NUM! | GRB2 |
| Hypoxia Signaling in the Cardiovascular System | 0.148 | 0.0133 | #NUM! | NFKBID |
| Myc Mediated Apoptosis Signaling | 0.146 | 0.0132 | #NUM! | GRB2 |
| p53 Signaling | 0 | 0.00901 | #NUM! | GRB2 |
| Tight Junction Signaling | 0 | 0.012 | #NUM! | MYL6,TNFRSF1B |
| IL-15 Signaling | 0 | 0.0122 | #NUM! | GRB2 |
| Molecular Mechanisms of Cancer | 0 | 0.0127 | #NUM! | GNAI2,NFKBID,RAC2,PLCB2,GRB2 |
| FLT3 Signaling in Hematopoietic Progenitor Cells | 0 | 0.0108 | #NUM! | GRB2 |
| Human Embryonic Stem Cell Pluripotency | 0 | 0.00699 | #NUM! | GRB2 |
| Role of NANOG in Mammalian Embryonic Stem Cell Pluripotency | 0 | 0.00781 | #NUM! | GRB2 |
| Prolactin Signaling | 0 | 0.0112 | #NUM! | GRB2 |
| Renal Cell Carcinoma Signaling | 0 | 0.0114 | #NUM! | GRB2 |
| Chronic Myeloid Leukemia Signaling | 0 | 0.00893 | #NUM! | GRB2 |
| Non-Small Cell Lung Cancer Signaling | 0 | 0.012 | #NUM! | GRB2 |
| Colorectal Cancer Metastasis Signaling | 0 | 0.00787 | #NUM! | TLR2,GRB2 |
| AMPK Signaling | 0 | 0.00463 | #NUM! | GRB2 |
| Hereditary Breast Cancer Signaling | 0 | 0.00667 | #NUM! | GRB2 |
| Ovarian Cancer Signaling | 0 | 0.00667 | #NUM! | GRB2 |
| Estrogen-Dependent Breast Cancer Signaling | 0 | 0.0116 | #NUM! | GRB2 |
| Glioblastoma Multiforme Signaling | 0 | 0.0119 | #NUM! | PLCB2,GRB2 |
| Role of JAK1 and JAK3 in γc Cytokine Signaling | 0 | 0.013 | #NUM! | GRB2 |
| Sertoli Cell-Sertoli Cell Junction Signaling | 0 | 0.0109 | #NUM! | MAP3K8,A2M |
| Telomerase Signaling | 0 | 0.00855 | #NUM! | GRB2 |
| Mouse Embryonic Stem Cell Pluripotency | 0 | 0.00893 | #NUM! | GRB2 |
| eNOS Signaling | 0 | 0.0116 | #NUM! | CALM1 (includes others),GRB2 |
| VEGF Family Ligand-Receptor Interactions | 0 | 0.0106 | #NUM! | GRB2 |
| ErbB4 Signaling | 0 | 0.0128 | #NUM! | GRB2 |
| Gαs Signaling | 0 | 0.00909 | #NUM! | HCK |
| Regulation of the Epithelial-Mesenchymal Transition Pathway | 0 | 0.00513 | #NUM! | GRB2 |
| Estrogen Receptor Signaling | 0 | 0.00746 | #NUM! | GRB2 |
| Cardiac β-adrenergic Signaling | 0 | 0.00709 | #NUM! | AKAP13 |
| Protein Ubiquitination Pathway | 0 | 0.00755 | #NUM! | HLA-C,HLA-B |
| GABA Receptor Signaling | 0 | 0.0105 | #NUM! | AP1B1 |
| Neurotrophin/TRK Signaling | 0 | 0.012 | #NUM! | GRB2 |
| IGF-1 Signaling | 0 | 0.00893 | #NUM! | GRB2 |
| TGF-β Signaling | 0 | 0.0108 | #NUM! | GRB2 |
| PDGF Signaling | 0 | 0.0104 | #NUM! | GRB2 |
| BMP signaling pathway | 0 | 0.0122 | #NUM! | GRB2 |
| Gustation Pathway | 0 | 0.013 | #NUM! | PLCB2,P2RX4 |
| Sumoylation Pathway | 0 | 0.0104 | #NUM! | ARHGDIB |
| Sirtuin Signaling Pathway | 0 | 0.0103 | #NUM! | UCP2,TSPO,ATP5F1E |

| Cluster 5 |  |  |  |  |
| --- | --- | --- | --- | --- |
| © 2000-2018 QIAGEN. All rights reserved. |  |  |  |  |
| Ingenuity Canonical Pathways | -log(B-H p-value) | Ratio | z-score | Molecules |
| Dendritic Cell Maturation | 16.8 | 0.186 | 5.831 | B2M,IL1A,PLCB2,HLA-DOA,ICAM1,HLA-B,PIK3R5,HLA-DQA1,CD83,HLA-DQB1,FCGR1A,HLA-DRB1,NFKBIA,HLA-DMA,HLA-DRA,HLA-DMB,ATF4,LTBR,TNFRSF1B,FCGR3A/FCGR3B,FCGR1B,FCGR2A,TYROBP,GRB2,CD58,TLR2,NFKBID,IL18,HLA-C,TREM2,FCER1G,CD86,IL1B,IRF8,TNF,HLA-DRB5 |
| Antigen Presentation Pathway | 15.9 | 0.474 | #NUM! | B2M,HLA-DOA,HLA-B,HLA-DQA1,CD74,HLA-DQB1,HLA-DRB1,HLA-C,HLA-DMA,HLA-DMB,HLA-DRA,HLA-DQA2,HLA-F,HLA-DPB1,TAPBP,HLA-DPA1,HLA-DRB5,HLA-E |
| Neuroinflammation Signaling Pathway | 15.9 | 0.138 | 6.091 | B2M,HLA-DOA,ICAM1,TGFBR1,PYCARD,HLA-B,PIK3R5,HLA-DQA1,SLC1A3,HLA-DQB1,CCL5,CX3CR1,CCL3,HMOX1,SOD2,HLA-DRB1,HLA-DMA,HLA-DRA,TLR1,HLA-DMB,CYBB,TLR7,ATF4,CXCL8,NLRP3,TYROBP,GRB2,IFNGR1,IRAK3,CSF1R,TLR2,IL18,HLA-C,SYK,TREM2,NCF2,IL1B,CD86,CFLAR,JAK3,TNF,HLA-DRB5,IRAK2 |
| Communication between Innate and Adaptive Immune Cells | 15.5 | 0.263 | #NUM! | B2M,CXCL8,IL1A,CD4,HLA-B,CD83,CCL5,CCL3,TLR2,IL18,HLA-DRB1,CCL4,HLA-C,CCL3L3,TNFSF13,HLA-DRA,TLR1,TLR7,FCER1G,IL1B,CD86,HLA-F,TNF,HLA-E,HLA-DRB5 |
| Graft-versus-Host Disease Signaling | 13.9 | 0.375 | #NUM! | IL1A,HLA-DOA,HLA-B,HLA-DQA1,HLA-DQB1,IL18,HLA-DRB1,HLA-C,HLA-DMA,HLA-DMB,HLA-DRA,FCER1G,CD86,IL1B,HLA-F,TNF,HLA-DRB5,HLA-E |
| Role of NFAT in Regulation of the Immune Response | 13.2 | 0.161 | 5.292 | BLNK,PLCB2,HLA-DOA,CD4,HLA-B,PIK3R5,HLA-DQA1,HLA-DQB1,FCGR1A,GNB4,NFKBIA,HLA-DRB1,HLA-DMA,HLA-DRA,HLA-DMB,GNA13,FCGR3A/FCGR3B,FCGR1B,FCGR2A,GRB2,MEF2A,BTK,NFKBID,CALM1 (includes others),SYK,LYN,FCER1G,CD86,MEF2C,LCP2,HLA-DRB5 |
| CD28 Signaling in T Helper Cells | 13.1 | 0.197 | 3.207 | ACTR2,HLA-DOA,PTPN6,ARPC1B,GRB2,CD4,HLA-B,HLA-DQA1,PIK3R5,HLA-DQB1,PTPRC,CALM1 (includes others),NFKBID,ACTR3,NFKBIA,HLA-DRB1,HLA-DMA,ARPC2,SYK,HLA-DRA,HLA-DMB,FCER1G,CD86,HLA-DRB5,ARPC4,LCP2 |
| Th1 and Th2 Activation Pathway | 12.9 | 0.162 | #NUM! | HLA-DOA,ICAM1,TGFBR1,HAVCR2,CD4,HLA-B,PIK3R5,HLA-DQA1,LGALS9,HLA-DQB1,HLA-DRB1,HLA-DMA,HLA-DRA,HLA-DMB,HLA-DPB1,HLA-DPA1,CCR1,STAT6,CXCR4,BHLHE41,GRB2,IKZF1,IFNGR1,ITGB2,IL18,IL10RA,CD86,JAK3,HLA-DQA2,HLA-DRB5 |
| Phagosome Formation | 12.3 | 0.191 | #NUM! | FCAR,PLCB2,MSR1,PIK3R5,FCGR1A,RHOH,RHOG,TLR1,TLR7,FCGR3A/FCGR3B,FCGR1B,C5AR1,GRB2,FCGR2A,INPP5D,TLR2,ITGB2,CLEC7A,ITGAM,SYK,PRKCD,FCER1G,PRKCH,C3AR1,ITGAX |
| Fcγ Receptor-mediated Phagocytosis in Macrophages and Monocytes | 11.8 | 0.226 | 4.583 | ACTR2,ARPC1B,FCGR2A,FYB1,FCGR1A,INPP5D,PLD4,HMOX1,ACTR3,PRKCD,SYK,EZR,ARPC2,LYN,VAMP3,HCK,PRKCH,FCGR3A/FCGR3B,ARPC4,LCP2,FGR |
| T Helper Cell Differentiation | 11.8 | 0.26 | #NUM! | IL6ST,STAT6,HLA-DOA,TGFBR1,HLA-B,HLA-DQA1,IFNGR1,HLA-DQB1,IL18,HLA-DRB1,HLA-DMA,HLA-DMB,HLA-DRA,FCER1G,IL10RA,CD86,TNFRSF1B,TNF,HLA-DRB5 |
| Th1 Pathway | 11.2 | 0.178 | 4 | HLA-DOA,ICAM1,GRB2,CD4,HAVCR2,HLA-B,HLA-DQA1,PIK3R5,LGALS9,IFNGR1,HLA-DQB1,ITGB2,IL18,HLA-DRB1,HLA-DMA,HLA-DMB,HLA-DRA,IL10RA,CD86,HLA-DQA2,JAK3,HLA-DPB1,HLA-DPA1,HLA-DRB5 |
| Th2 Pathway | 11.1 | 0.167 | 2.333 | HLA-DOA,ICAM1,TGFBR1,CD4,HLA-B,PIK3R5,HLA-DQA1,HLA-DQB1,HLA-DRB1,HLA-DMA,HLA-DMB,HLA-DRA,HLA-DPB1,HLA-DPA1,CCR1,STAT6,CXCR4,BHLHE41,GRB2,IKZF1,ITGB2,CD86,JAK3,HLA-DQA2,HLA-DRB5 |
| OX40 Signaling Pathway | 11 | 0.22 | -1 | B2M,HLA-DOA,CD4,HLA-B,HLA-DQA1,HLA-DQB1,NFKBID,HLA-DRB1,NFKBIA,HLA-C,HLA-DMA,HLA-DMB,HLA-DRA,FCER1G,HLA-DQA2,HLA-F,HLA-DPB1,HLA-DPA1,HLA-DRB5,HLA-E |
| Allograft Rejection Signaling | 10.6 | 0.224 | #NUM! | B2M,HLA-DOA,HLA-B,HLA-DQA1,HLA-DQB1,HLA-DRB1,HLA-C,HLA-DMA,HLA-DMB,HLA-DRA,FCER1G,CD86,HLA-DQA2,HLA-F,HLA-DPB1,TNF,HLA-DPA1,HLA-DRB5,HLA-E |
| Type I Diabetes Mellitus Signaling | 10.3 | 0.189 | 2.828 | HLA-DOA,HLA-B,HLA-DQA1,IFNGR1,HLA-DQB1,NFKBID,HLA-DRB1,NFKBIA,HLA-C,HLA-DMA,HLA-DMB,HLA-DRA,FCER1G,CD86,IL1B,MAP2K3,TNFRSF1B,HLA-F,TNF,HLA-DRB5,HLA-E |
| Role of Pattern Recognition Receptors in Recognition of Bacteria and Viruses | 10.3 | 0.168 | 4 | CXCL8,IL1A,NLRP3,C5AR1,C3,GRB2,PIK3R5,C1QA,C1QC,CCL5,C1QB,TLR2,CLEC7A,IL18,PRKCD,SYK,TLR1,TLR7,OSM,IL1B,PRKCH,TNF,C3AR1 |
| Altered T Cell and B Cell Signaling in Rheumatoid Arthritis | 10.2 | 0.211 | #NUM! | IL1A,HLA-DOA,HLA-B,HLA-DQA1,HLA-DQB1,TLR2,IL18,HLA-DRB1,HLA-DMA,TNFSF13,HLA-DMB,TLR1,HLA-DRA,TLR7,FCER1G,CD86,IL1B,TNF,HLA-DRB5 |
| TREM1 Signaling | 9.62 | 0.227 | 4.123 | CXCL8,TREM1,ICAM1,NLRP3,GRB2,TYROBP,LAT2,CD83,CCL3,TLR2,IL18,TLR1,TLR7,CD86,IL1B,TNF,ITGAX |
| Toll-like Receptor Signaling | 9.54 | 0.224 | 2.887 | IL1A,TNFAIP3,IRAK3,TLR2,IL18,LY96,NFKBIA,UBA52,TLR1,TLR7,RPS27A,IL1B,CD14,MAP2K3,UBC,TNF,IRAK2 |
| Production of Nitric Oxide and Reactive Oxygen Species in Macrophages | 9.49 | 0.134 | 5 | APOE,PIK3R5,APOC2,RHOH,LYZ,NFKBIA,RHOG,CYBA,CYBB,SERPINA1,TNFRSF1B,PTPN6,GRB2,IFNGR1,NCF4,TLR2,NFKBID,PRKCD,NCF2,APOC1,MAP3K8,PRKCH,IRF8,JAK3,TNF,SIRPA |
| Autoimmune Thyroid Disease Signaling | 9.46 | 0.292 | #NUM! | HLA-DOA,HLA-B,HLA-DQA1,HLA-DQB1,HLA-DRB1,HLA-DMA,HLA-C,HLA-DRA,HLA-DMB,FCER1G,CD86,HLA-F,HLA-E,HLA-DRB5 |
| EIF2 Signaling | 8.71 | 0.119 | 3.5 | RPL22,RPLP1,RPS3A,RPS23,PIK3R5,RPL26,RPS4X,RPS20,UBA52,EIF3A,ATF4,RPS3,PABPC1,RPL4,RPL3,GRB2,RPL23,EIF2S3,RPL28,RPL9,PPP1R15A,RPL10,RPS27A,RPL5,RPL6,EIF3L,RPSA |
| Cdc42 Signaling | 8.62 | 0.138 | 2.646 | B2M,ACTR2,HLA-DOA,ARPC1B,HLA-B,HLA-DQA1,HLA-DQB1,HLA-DRB1,ACTR3,HLA-C,HLA-DMA,ARPC2,HLA-DMB,HLA-DRA,FCER1G,HLA-DQA2,HLA-F,HLA-DPB1,HLA-DPA1,HLA-DRB5,ARPC4,HLA-E,MYL12A |
| Calcium-induced T Lymphocyte Apoptosis | 8.54 | 0.227 | 3.742 | HLA-DOA,CD4,HLA-B,HLA-DQA1,HLA-DQB1,CALM1 (includes others),HLA-DRB1,HLA-DMA,PRKCD,HLA-DRA,HLA-DMB,NR4A1,FCER1G,PRKCH,HLA-DRB5 |
| Crosstalk between Dendritic Cells and Natural Killer Cells | 8.5 | 0.191 | #NUM! | TYROBP,CD69,HLA-B,CD83,IL18,HLA-DRB1,HLA-C,TREM2,HLA-DRA,TLR7,CD86,LTBR,TNFRSF1B,HLA-F,TNF,HLA-DRB5,HLA-E |
| Granulocyte Adhesion and Diapedesis | 7.96 | 0.127 | #NUM! | CSF3R,CXCL8,IL1A,SELL,ICAM1,C5AR1,CXCR4,CCL5,CCL3,CCL4L1/CCL4L2,SELPLG,FPR1,CXCL16,ITGB2,IL18,CCL4,ITGAM,CCL3L3,EZR,IL1B,TNFRSF1B,TNF,MSN |
| iCOS-iCOSL Signaling in T Helper Cells | 7.94 | 0.154 | 3.357 | HLA-DOA,GRB2,CD4,HLA-B,HLA-DQA1,PIK3R5,HLA-DQB1,INPP5D,PTPRC,CALM1 (includes others),NFKBID,NFKBIA,HLA-DRB1,HLA-DMA,HLA-DMB,HLA-DRA,FCER1G,HLA-DRB5,LCP2 |
| Role of Macrophages, Fibroblasts and Endothelial Cells in Rheumatoid Arthritis | 7.93 | 0.0969 | #NUM! | IL6ST,IL1A,PLCB2,ICAM1,PIK3R5,CCL5,FCGR1A,NFKBIA,TLR1,TLR7,ATF4,OSM,LTBR,TNFRSF1B,FCGR3A/FCGR3B,CXCL8,C5AR1,GRB2,IRAK3,PDGFB,ROCK1,TLR2,NFKBID,CALM1 (includes others),IL18,PRKCD,IL1B,MAP2K3,PRKCH,TNF,IRAK2 |
| Atherosclerosis Signaling | 7.73 | 0.15 | #NUM! | APOE,CXCL8,IL1A,ICAM1,MSR1,CXCR4,APOC2,PDGFB,SELPLG,PLBD1,ITGB2,LYZ,IL18,ALOX15B,APOC1,IL1B,SERPINA1,ALOX5,TNF |
| B Cell Development | 7.68 | 0.306 | #NUM! | PTPRC,HLA-DOA,HLA-DRB1,HLA-DMA,HLA-DRA,HLA-B,HLA-DMB,HLA-DQA1,CD86,HLA-DQB1,HLA-DRB5 |
| Complement System | 7.55 | 0.297 | 1.89 | ITGB2,CD55,C5AR1,C3,ITGAM,C1QA,C1QC,C1QB,C3AR1,C2,ITGAX |
| Nur77 Signaling in T Lymphocytes | 7.26 | 0.22 | #NUM! | CALM1 (includes others),HLA-DOA,HLA-DRB1,HLA-DMA,HLA-B,HLA-DMB,HLA-DRA,FCER1G,NR4A1,HLA-DQA1,CD86,HLA-DQB1,HLA-DRB5 |
| IL-4 Signaling | 7.24 | 0.168 | #NUM! | STAT6,PTPN6,HLA-DOA,IL13RA1,GRB2,HLA-B,HLA-DQA1,PIK3R5,HLA-DQB1,INPP5D,HLA-DRB1,HLA-DMA,HLA-DMB,HLA-DRA,JAK3,HLA-DRB5 |
| p38 MAPK Signaling | 6.56 | 0.142 | 3.5 | IL1A,TGFBR1,MAPKAPK3,MEF2A,IRAK3,IL18,H3F3A/H3F3B,DUSP1,MKNK1,IL1B,ATF4,MAP2K3,MEF2C,RPS6KA1,TNFRSF1B,TNF,IRAK2 |
| IL-8 Signaling | 6.43 | 0.108 | 4.583 | CXCL8,PLCB2,ICAM1,GRB2,PIK3R5,IRAK3,RHOH,ROCK1,PLD4,GNB4,ITGB2,HMOX1,ARRB2,RHOG,ITGAM,PRKCD,NCF2,CYBB,PRKCH,GNA13,IRAK2,ITGAX |
| B Cell Receptor Signaling | 6.12 | 0.108 | 3.441 | BLNK,PTPN6,APBB1IP,GRB2,FCGR2A,PIK3R5,INPP5D,PTPRC,BTK,CALM1 (includes others),NFKBID,NFKBIA,SYK,LYN,ATF4,MAP2K3,MAP3K8,MEF2C,PIK3AP1,RASSF5,BCL2A1 |
| fMLP Signaling in Neutrophils | 6.12 | 0.132 | 2.84 | ACTR2,PLCB2,ARPC1B,GRB2,PIK3R5,FPR1,GNB4,CALM1 (includes others),NFKBID,NFKBIA,ACTR3,PRKCD,ARPC2,NCF2,CYBB,PRKCH,ARPC4 |
| LXR/RXR Activation | 5.77 | 0.132 | -0.775 | APOE,IL1A,C3,MSR1,APOC2,ABCA1,LYZ,IL18,LY96,APOC1,IL1B,CD14,SERPINA1,PLTP,TNFRSF1B,TNF |
| Cholecystokinin/Gastrin-mediated Signaling | 5.73 | 0.14 | 3.873 | PLCB2,IL1A,GRB2,MEF2A,RHOH,ROCK1,IL18,RHOG,PRKCD,IL1B,PRKCH,MEF2C,MAP2K3,GNA13,TNF |
| Phospholipase C Signaling | 5.68 | 0.0943 | 4.243 | BLNK,PLCB2,GRB2,FCGR2A,MEF2A,RHOH,BTK,PLD4,GNB4,CALM1 (includes others),HMOX1,RHOG,PRKCD,SYK,LYN,FCER1G,ATF4,PRKCH,MEF2C,GNA13,ADCY7,LCP2,MYL12A |
| IL-10 Signaling | 5.57 | 0.174 | #NUM! | CCR1,NFKBID,HMOX1,IL1A,IL18,NFKBIA,FCGR2A,IL10RA,CD14,IL1B,MAP2K3,TNF |
| Agranulocyte Adhesion and Diapedesis | 5.57 | 0.104 | #NUM! | CXCL8,IL1A,SELL,ICAM1,C5AR1,MYH9,CXCR4,CCL5,CCL3,CCL4L1/CCL4L2,SELPLG,CXCL16,ITGB2,IL18,CCL4,CCL3L3,EZR,IL1B,TNF,MSN |
| Leukocyte Extravasation Signaling | 5.57 | 0.0995 | 3.3 | ICAM1,GRB2,CXCR4,ARHGAP4,PIK3R5,NCF4,RHOH,SELPLG,ROCK1,BTK,ITGB2,ITGAM,ARHGAP9,CYBA,EZR,PRKCD,NCF2,CYBB,PRKCH,RASSF5,MSN |
| Natural Killer Cell Signaling | 5.49 | 0.125 | #NUM! | PTPN6,LAIR1,GRB2,FCGR2A,TYROBP,PIK3R5,LILRB1,INPP5D,CD300A,SYK,PRKCD,FCER1G,PRKCH,HCST,FCGR3A/FCGR3B,LCP2 |
| IL-12 Signaling and Production in Macrophages | 5.42 | 0.116 | #NUM! | APOE,STAT6,GRB2,PIK3R5,IFNGR1,APOC2,TLR2,LYZ,IL18,PRKCD,APOC1,PRKCH,SERPINA1,MAP3K8,IRF8,REL,TNF |
| PKCθ Signaling in T Lymphocytes | 5.34 | 0.109 | 4.243 | HLA-DOA,GRB2,CD4,HLA-B,HLA-DQA1,PIK3R5,HLA-DQB1,NFKBID,NFKBIA,HLA-DRB1,HLA-DMA,HLA-DMB,HLA-DRA,FCER1G,CD86,MAP3K8,HLA-DRB5,LCP2 |
| NF-κB Signaling | 5.18 | 0.102 | 2.524 | IL1A,TGFBR1,GRB2,PIK3R5,TNFAIP3,IRAK3,TLR2,NFKBID,IL18,NFKBIA,TLR1,PELI1,TLR7,FCER1G,IL1B,MAP3K8,LTBR,TNFRSF1B,TNF |
| Clathrin-mediated Endocytosis Signaling | 5.14 | 0.0966 | #NUM! | APOE,ACTR2,ARPC1B,GRB2,PIK3R5,APOC2,AP1B1,PDGFB,ITGB2,ARRB2,LYZ,ACTR3,UBA52,ARPC2,APOC1,DAB2,RPS27A,SERPINA1,UBC,ARPC4 |
| mTOR Signaling | 5.14 | 0.0966 | 3.162 | RPS3A,DDIT4,GRB2,RPS23,PIK3R5,RHOH,RPS4X,PLD4,HMOX1,RHOG,RPS20,PRKCD,EIF3A,RPS27A,PRKCH,RPS6KA1,RPS3,EIF3L,EIF4B,RPSA |
| RhoA Signaling | 5 | 0.121 | 2.673 | ACTR2,ARPC1B,ARHGAP4,ROCK1,LPAR6,ACTR3,ARHGAP9,ABL2,EZR,ARPC2,LPAR5,GNA13,ARPC4,MYL12A,MSN |
| Acute Phase Response Signaling | 4.97 | 0.102 | 3.5 | IL6ST,IL1A,C3,GRB2,HMOX1,NFKBID,FTL,IL18,NFKBIA,SOD2,OSM,IL1B,MAP2K3,SERPINA1,TNFRSF1B,TNF,A2M,C2 |
| Actin Cytoskeleton Signaling | 4.95 | 0.0901 | 3 | ACTR2,MYH9,WASF2,ARPC1B,GRB2,PIK3R5,GSN,PDGFB,ROCK1,ACTR3,CYFIP1,EZR,ARPC2,CD14,GNA13,SSH2,NCKAP1L,ARHGAP24,ARPC4,MYL12A,MSN |
| Systemic Lupus Erythematosus Signaling | 4.95 | 0.0901 | #NUM! | PTPN6,IL1A,GRB2,FCGR2A,HLA-B,PIK3R5,FCGR1A,INPP5D,PTPRC,IL18,HLA-C,LYN,TLR7,FCER1G,CD86,IL1B,HLA-F,TNF,FCGR3A/FCGR3B,FCGR1B,HLA-E |
| Role of Hypercytokinemia/hyperchemokinemia in the Pathogenesis of Influenza | 4.89 | 0.209 | #NUM! | CCR1,CXCL8,IL1A,IL18,CCL4,IL1B,CCL5,CCL3,TNF |
| Phagosome Maturation | 4.73 | 0.108 | #NUM! | B2M,TCIRG1,HLA-B,TUBB,HLA-DRB1,CTSL,HLA-C,CTSS,CTSH,HLA-DRA,NCF2,CYBB,VAMP3,CTSB,CTSC,HLA-DRB5 |
| iNOS Signaling | 4.73 | 0.2 | 2.828 | NFKBID,CALM1 (includes others),LY96,NFKBIA,CD14,IFNGR1,IRAK3,JAK3,IRAK2 |
| RhoGDI Signaling | 4.35 | 0.096 | -3.357 | ACTR2,WASF2,ARPC1B,ARHGAP4,RHOH,ARHGDIB,ROCK1,GNB4,ACTR3,RHOG,ARHGAP9,EZR,ARPC2,GNA13,ARPC4,MYL12A,MSN |
| Hepatic Cholestasis | 4.31 | 0.1 | #NUM! | CXCL8,IL1A,IRAK3,NFKBID,IL18,LY96,NFKBIA,PRKCD,OSM,IL1B,CD14,PRKCH,TNFRSF1B,TNF,ADCY7,IRAK2 |
| Gαq Signaling | 4.29 | 0.0994 | 3.051 | RGS2,PLCB2,GRB2,PIK3R5,RHOH,ROCK1,BTK,PLD4,GNB4,CALM1 (includes others),HMOX1,NFKBID,NFKBIA,RHOG,PRKCD,PRKCH |
| Inflammasome pathway | 4.15 | 0.3 | 2.449 | NAIP,IL18,NLRP3,PYCARD,CTSB,IL1B |
| Tec Kinase Signaling | 4 | 0.0941 | 3.606 | STAT6,GRB2,PIK3R5,RHOH,BTK,GNB4,RHOG,PRKCD,LYN,HCK,FCER1G,PRKCH,GNA13,JAK3,TNF,FGR |
| IL-6 Signaling | 3.99 | 0.104 | 3.606 | IL6ST,CXCL8,NFKBID,IL1A,IL18,NFKBIA,GRB2,PIK3R5,CD14,IL1B,MAP2K3,TNFRSF1B,TNF,A2M |
| CXCR4 Signaling | 3.98 | 0.0936 | 3.207 | PLCB2,CXCR4,GRB2,CD4,PIK3R5,RHOH,ROCK1,GNB4,RHOG,PRKCD,LYN,PRKCH,GNA13,ELMO1,ADCY7,MYL12A |
| PI3K Signaling in B Lymphocytes | 3.93 | 0.103 | 3.207 | BLNK,PLCB2,C3,ATF6,INPP5D,BTK,PTPRC,CALM1 (includes others),NFKBID,NFKBIA,SYK,LYN,ATF4,PIK3AP1 |
| HMGB1 Signaling | 3.83 | 0.101 | 3.742 | CXCL8,IL1A,ICAM1,GRB2,PIK3R5,IFNGR1,RHOH,IL18,RHOG,OSM,IL1B,MAP2K3,TNFRSF1B,TNF |
| Differential Regulation of Cytokine Production in Intestinal Epithelial Cells by IL-17A and IL-17F | 3.81 | 0.261 | #NUM! | IL1A,CCL4,IL1B,CCL5,CCL3,TNF |
| Cardiac Hypertrophy Signaling | 3.73 | 0.0788 | 4.123 | PLCB2,TGFBR1,MAPKAPK3,GRB2,MEF2A,PIK3R5,RHOH,ROCK1,GNB4,CALM1 (includes others),RHOG,MAP2K3,MAP3K8,MEF2C,RPS6KA1,GNA13,ADCY7,ADRB2,MYL12A |
| Actin Nucleation by ARP-WASP Complex | 3.63 | 0.145 | 2.828 | ROCK1,ACTR2,ACTR3,RHOG,ARPC1B,GRB2,ARPC2,RHOH,ARPC4 |
| Pathogenesis of Multiple Sclerosis | 3.46 | 0.444 | #NUM! | CCR1,CCL4,CCL5,CCL3 |
| CTLA4 Signaling in Cytotoxic T Lymphocytes | 3.38 | 0.111 | #NUM! | B2M,PTPN6,HLA-C,GRB2,SYK,HLA-B,FCER1G,PIK3R5,CD86,AP1B1,LCP2 |
| Eicosanoid Signaling | 3.37 | 0.134 | #NUM! | PLBD1,ALOX15B,LTC4S,PTGS1,ALOX5AP,HPGDS,TBXAS1,ALOX5,PTGER4 |
| Differential Regulation of Cytokine Production in Macrophages and T Helper Cells by IL-17A and IL-17F | 3.3 | 0.278 | #NUM! | CCL4,IL1B,CCL5,CCL3,TNF |
| Integrin Signaling | 3.27 | 0.0776 | 4 | ACTR2,ARPC1B,GRB2,PIK3R5,GSN,RHOH,PDGFB,ROCK1,ITGB2,ITGAM,ACTR3,RHOG,LIMS1,ARPC2,ARPC4,MYL12A,ITGAX |
| TNFR2 Signaling | 3.16 | 0.2 | 1 | NFKBID,NAIP,NFKBIA,TNFAIP3,TNFRSF1B,TNF |
| Regulation of eIF4 and p70S6K Signaling | 3.13 | 0.0859 | #NUM! | PABPC1,RPS3A,GRB2,RPS23,PIK3R5,EIF2S3,RPS4X,RPS20,MKNK1,EIF3A,RPS27A,RPS3,EIF3L,RPSA |
| Fc Epsilon RI Signaling | 3.12 | 0.096 | 2.714 | BTK,GRB2,SYK,PRKCD,LYN,FCER1G,PIK3R5,PRKCH,MAP2K3,TNF,INPP5D,LCP2 |
| Regulation of Actin-based Motility by Rho | 3.09 | 0.111 | 3 | ROCK1,ACTR2,ACTR3,RHOG,ARPC1B,ARPC2,GSN,RHOH,ARPC4,MYL12A |
| Hepatic Fibrosis / Hepatic Stellate Cell Activation | 3.05 | 0.0802 | #NUM! | CXCL8,IL1A,ICAM1,TGFBR1,MYH9,IFNGR1,CCL5,PDGFB,LY96,IL10RA,IL1B,CD14,TNFRSF1B,A2M,TNF |
| Signaling by Rho Family GTPases | 3.05 | 0.0714 | 4 | ACTR2,ARPC1B,GRB2,PIK3R5,RHOH,ROCK1,GNB4,RHOG,ACTR3,CYFIP1,EZR,ARPC2,NCF2,CYBB,GNA13,ARPC4,MYL12A,MSN |
| Glucocorticoid Receptor Signaling | 3.04 | 0.0638 | #NUM! | CXCL8,ICAM1,TGFBR1,GRB2,SGK1,HSPA1A/HSPA1B,PIK3R5,CCL5,CD163,CCL3,FCGR1A,TSC22D3,NFKBID,NFKBIA,DUSP1,CDKN1A,IL1B,FKBP5,JAK3,TNF,A2M,ADRB2 |
| Death Receptor Signaling | 3 | 0.108 | 0.632 | ROCK1,NFKBID,NAIP,NFKBIA,ZC3HAV1,CFLAR,TNFRSF1B,TNF,ARHGDIB,PARP14 |
| VDR/RXR Activation | 2.92 | 0.115 | 0.378 | SERPINB1,PPARD,PRKCD,CDKN1A,CD14,PRKCH,CCL5,THBD,KLF4 |
| CD40 Signaling | 2.88 | 0.114 | 0.707 | NFKBID,NFKBIA,ICAM1,GRB2,PTGS1,PIK3R5,TNFAIP3,MAP2K3,JAK3 |
| GP6 Signaling Pathway | 2.88 | 0.0896 | 3.464 | BTK,CALM1 (includes others),RHOG,APBB1IP,GRB2,SYK,PRKCD,LYN,FCER1G,PIK3R5,PRKCH,LCP2 |
| PPAR Signaling | 2.72 | 0.099 | -3.162 | NFKBID,IL1A,IL18,NFKBIA,GRB2,PPARD,IL1B,TNFRSF1B,TNF,PDGFB |
| Opioid Signaling Pathway | 2.72 | 0.0688 | 1.807 | RGS1,RGS19,AP1B1,FOSB,CALM1 (includes others),ARRB2,NFKBIA,PRKCD,RGS10,LYN,HCK,ATF4,PRKCH,MAP2K3,RPS6KA1,ADCY7,FGR |
| Gα12/13 Signaling | 2.69 | 0.0851 | 3.162 | ROCK1,BTK,NFKBID,LPAR6,NFKBIA,GRB2,MEF2A,PIK3R5,LPAR5,MEF2C,GNA13,MYL12A |
| Role of IL-17A in Arthritis | 2.62 | 0.116 | #NUM! | CXCL8,NFKBID,NFKBIA,GRB2,PIK3R5,MAP2K3,RPS6KA1,CCL5 |
| Caveolar-mediated Endocytosis Signaling | 2.54 | 0.113 | #NUM! | B2M,ITGB2,CD55,ITGAM,HLA-C,PTPN1,HLA-B,ITGAX |
| G-Protein Coupled Receptor Signaling | 2.52 | 0.0638 | #NUM! | RGS2,PLCB2,GRB2,PIK3R5,ADORA3,FPR1,P2RY13,NFKBID,NFKBIA,DUSP1,RGS10,P2RY12,ATF4,MAP3K8,RPS6KA1,ADCY7,PTGER4,ADRB2 |
| ERK5 Signaling | 2.51 | 0.111 | 2.828 | IL6ST,SGK1,MEF2A,ATF4,MEF2C,MAP3K8,RPS6KA1,GNA13 |
| Role of Tissue Factor in Cancer | 2.46 | 0.0846 | #NUM! | CXCL8,ARRB2,GRB2,LYN,PIK3R5,HCK,PLAUR,IL1B,RPS6KA1,GNA13,FGR |
| IL-1 Signaling | 2.44 | 0.0978 | 2.828 | NFKBID,GNB4,IL1A,NFKBIA,MAP2K3,IRAK3,GNA13,ADCY7,IRAK2 |
| LPS/IL-1 Mediated Inhibition of RXR Function | 2.35 | 0.0676 | 0 | APOE,IL1A,APOC2,ABCA1,IL18,LY96,MGST2,APOC1,IL1B,CD14,PLTP,TNFRSF1B,ABCC4,TNF,ACSL1 |
| CCR5 Signaling in Macrophages | 2.35 | 0.0947 | 1.633 | CALM1 (includes others),GNB4,CCL4,CD4,PRKCD,FCER1G,PRKCH,CCL5,CCL3 |
| Virus Entry via Endocytic Pathways | 2.3 | 0.0862 | #NUM! | B2M,ITGB2,CD55,HLA-C,GRB2,PRKCD,HLA-B,PIK3R5,PRKCH,AP1B1 |
| Induction of Apoptosis by HIV1 | 2.29 | 0.115 | 0.378 | NFKBID,NAIP,NFKBIA,CXCR4,SLC25A3,TNFRSF1B,TNF |
| Protein Kinase A Signaling | 2.25 | 0.0549 | 0.728 | PTPN6,PLCB2,TGFBR1,DUSP2,PTPRC,ROCK1,AKAP13,GNB4,CALM1 (includes others),NFKBID,H3F3A/H3F3B,NFKBIA,DUSP1,PRKCD,PTPN1,ATF4,PRKCH,GNA13,EBI3,ADCY7,SIRPA,MYL12A |
| Prostanoid Biosynthesis | 2.22 | 0.333 | #NUM! | PTGS1,HPGDS,TBXAS1 |
| Rac Signaling | 2.13 | 0.0813 | 3.162 | ACTR2,ACTR3,ARPC1B,GRB2,CYFIP1,ARPC2,NCF2,CYBB,PIK3R5,ARPC4 |
| Superpathway of Inositol Phosphate Compounds | 2.11 | 0.0636 | 3.873 | PLCB2,PTPN6,GRB2,PIK3R5,DUSP2,INPP5D,PTPRC,PLD4,DUSP1,NUDT16,PTPN1,CD86,PIK3AP1,PALD1,SIRPA |
| Sphingosine-1-phosphate Signaling | 2.09 | 0.08 | 2.333 | PLCB2,RHOG,GRB2,SPHK1,PIK3R5,GNA13,ADCY7,RHOH,PDGFB,ASAH1 |
| FXR/RXR Activation | 2.06 | 0.0794 | #NUM! | APOE,IL1A,IL18,C3,APOC1,IL1B,SERPINA1,APOC2,PLTP,TNF |
| Coagulation System | 2.05 | 0.143 | 0.447 | F13A1,PLAUR,SERPINA1,THBD,A2M |
| MIF-mediated Glucocorticoid Regulation | 2.05 | 0.143 | 2.236 | NFKBID,LY96,NFKBIA,CD14,CD74 |
| Macropinocytosis Signaling | 2.04 | 0.092 | 2.236 | ITGB2,GRB2,PRKCD,PIK3R5,CD14,PRKCH,CSF1R,PDGFB |
| Antioxidant Action of Vitamin C | 2.01 | 0.0833 | -2.828 | SLC2A5,PLBD1,PLD4,NFKBID,HMOX1,PLCB2,NFKBIA,CSF2RA,TNF |
| NRF2-mediated Oxidative Stress Response | 1.95 | 0.0653 | 2.828 | GRB2,PIK3R5,HERPUD1,HMOX1,FTL,SOD2,MGST2,PRKCD,ATF4,MAP2K3,PRKCH,FKBP5,ABCC4 |
| Axonal Guidance Signaling | 1.92 | 0.0503 | #NUM! | ACTR2,PLCB2,ARPC1B,GRB2,CXCR4,PIK3R5,TUBB,PDGFB,ROCK1,GNB4,SEMA4D,ACTR3,ADAM28,PRKCD,ARPC2,MKNK1,SRGAP2,PRKCH,PLXNB2,GNA13,RASSF5,ARPC4,MYL12A |
| Ephrin Receptor Signaling | 1.9 | 0.067 | 3.317 | ROCK1,GNB4,ACTR2,ACTR3,ARPC1B,GRB2,CXCR4,ARPC2,ATF4,GNA13,PDGFB,ARPC4 |
| LPS-stimulated MAPK Signaling | 1.89 | 0.086 | 2.828 | NFKBID,NFKBIA,GRB2,PRKCD,PIK3R5,CD14,PRKCH,MAP2K3 |
| NF-κB Activation by Viruses | 1.89 | 0.086 | 2.828 | NFKBID,ITGB2,NFKBIA,GRB2,CD4,PRKCD,PIK3R5,PRKCH |
| PEDF Signaling | 1.89 | 0.086 | 0 | ROCK1,NFKBID,NFKBIA,SOD2,WASF2,GRB2,PIK3R5,CFLAR |
| cAMP-mediated signaling | 1.88 | 0.0614 | 1.941 | RGS2,ADORA3,FPR1,AKAP13,CALM1 (includes others),P2RY13,DUSP1,RGS10,P2RY12,ATF4,RPS6KA1,ADCY7,PTGER4,ADRB2 |
| Colorectal Cancer Metastasis Signaling | 1.86 | 0.0591 | 3.207 | IL6ST,TGFBR1,GRB2,PIK3R5,IFNGR1,RHOH,TLR2,GNB4,RHOG,TLR1,TLR7,JAK3,ADCY7,TNF,PTGER4 |
| Apoptosis Signaling | 1.81 | 0.0833 | 0.707 | ROCK1,NFKBID,NAIP,NFKBIA,RPS6KA1,BCL2A1,TNFRSF1B,TNF |
| Role of Osteoblasts, Osteoclasts and Chondrocytes in Rheumatoid Arthritis | 1.81 | 0.0601 | #NUM! | NAIP,NFKBID,CALM1 (includes others),IL1A,IL18,NFKBIA,GRB2,PIK3R5,IL1B,MAP2K3,GSN,TNFRSF1B,TNF,CSF1R |
| Leukotriene Biosynthesis | 1.8 | 0.231 | #NUM! | MGST2,LTC4S,ALOX5 |
| Role of PKR in Interferon Induction and Antiviral Response | 1.79 | 0.122 | #NUM! | NFKBID,NFKBIA,MAP2K3,FCGR1A,TNF |
| Lipid Antigen Presentation by CD1 | 1.78 | 0.154 | #NUM! | B2M,FCER1G,PSAP,AP1B1 |
| Antiproliferative Role of TOB in T Cell Signaling | 1.78 | 0.154 | #NUM! | PABPC1,RB1,TGFBR1,RPS6KA1 |
| GM-CSF Signaling | 1.74 | 0.0886 | 2.449 | RUNX1,GRB2,CSF2RA,LYN,PIK3R5,HCK,BCL2A1 |
| MIF Regulation of Innate Immunity | 1.72 | 0.116 | 2.236 | NFKBID,LY96,NFKBIA,CD14,CD74 |
| IL-15 Signaling | 1.66 | 0.0854 | #NUM! | CXCL8,STAT6,GRB2,SYK,PIK3R5,JAK3,TNF |
| Trehalose Degradation II (Trehalase) | 1.65 | 0.4 | #NUM! | HK1,HK2 |
| Role of IL-17F in Allergic Inflammatory Airway Diseases | 1.6 | 0.109 | 2.236 | CXCL8,CCL4,IL1B,ATF4,RPS6KA1 |
| FcγRIIB Signaling in B Lymphocytes | 1.59 | 0.0824 | 1.89 | BLNK,BTK,GRB2,SYK,LYN,PIK3R5,INPP5D |
| Growth Hormone Signaling | 1.59 | 0.0824 | 1.89 | PTPN6,GRB2,PRKCD,PIK3R5,PRKCH,RPS6KA1,A2M |
| Role of NFAT in Cardiac Hypertrophy | 1.59 | 0.0578 | 3.464 | IL6ST,PLCB2,TGFBR1,GRB2,MEF2A,PIK3R5,GNB4,CALM1 (includes others),PRKCD,MEF2C,MAP2K3,PRKCH,ADCY7 |
| 3-phosphoinositide Biosynthesis | 1.57 | 0.0597 | 3.464 | PTPRC,PTPN6,NUDT16,GRB2,DUSP1,PTPN1,PIK3R5,CD86,PIK3AP1,PALD1,DUSP2,SIRPA |
| Erythropoietin Signaling | 1.55 | 0.0805 | #NUM! | NFKBID,PTPN6,NFKBIA,GRB2,PRKCD,PIK3R5,PRKCH |
| Germ Cell-Sertoli Cell Junction Signaling | 1.54 | 0.0615 | #NUM! | TGFBR1,RHOG,GRB2,PIK3R5,MAP2K3,MAP3K8,GSN,TUBB,TNF,RHOH,A2M |
| Primary Immunodeficiency Signaling | 1.51 | 0.102 | #NUM! | BLNK,PTPRC,BTK,CD4,JAK3 |
| IL-3 Signaling | 1.51 | 0.0787 | 1.134 | STAT6,PTPN6,GRB2,PRKCD,PIK3R5,PRKCH,INPP5D |
| JAK/Stat Signaling | 1.51 | 0.0787 | 0.378 | STAT6,PTPN6,GRB2,PTPN1,CDKN1A,PIK3R5,JAK3 |
| Cytotoxic T Lymphocyte-mediated Apoptosis of Target Cells | 1.51 | 0.125 | #NUM! | B2M,HLA-C,HLA-B,FCER1G |
| Remodeling of Epithelial Adherens Junctions | 1.5 | 0.087 | 2.236 | ACTR2,ACTR3,ARPC1B,ARPC2,TUBB,ARPC4 |
| TNFR1 Signaling | 1.49 | 0.1 | 1 | NFKBID,NAIP,NFKBIA,TNFAIP3,TNF |
| Thrombin Signaling | 1.46 | 0.0571 | 2.53 | ROCK1,GNB4,PLCB2,RHOG,GRB2,PRKCD,PIK3R5,PRKCH,GNA13,ADCY7,RHOH,MYL12A |
| Iron homeostasis signaling pathway | 1.46 | 0.0657 | #NUM! | HMOX1,FTL,TCIRG1,SLC11A2,SLC25A37,CD163,JAK3,SLC11A1,PDGFB |
| Reelin Signaling in Neurons | 1.46 | 0.0761 | #NUM! | APOE,ITGB2,GRB2,LYN,PIK3R5,HCK,FGR |
| Breast Cancer Regulation by Stathmin1 | 1.46 | 0.0569 | #NUM! | ROCK1,CALM1 (includes others),GNB4,PLCB2,GRB2,PRKCD,CDKN1A,PIK3R5,PRKCH,GNA13,TUBB,ADCY7 |
| p70S6K Signaling | 1.45 | 0.0652 | 3 | BTK,PLCB2,EEF2,GRB2,SYK,PRKCD,LYN,PIK3R5,PRKCH |
| D-myo-inositol-5-phosphate Metabolism | 1.45 | 0.0617 | 3.162 | PTPRC,PLD4,PTPN6,PLCB2,NUDT16,DUSP1,PTPN1,PALD1,DUSP2,SIRPA |
| T Cell Receptor Signaling | 1.45 | 0.0696 | #NUM! | PTPRC,BTK,CALM1 (includes others),NFKBIA,GRB2,CD4,PIK3R5,LCP2 |
| MSP-RON Signaling Pathway | 1.44 | 0.0833 | #NUM! | TLR2,ITGB2,ITGAM,GRB2,PIK3R5,TNF |
| P2Y Purigenic Receptor Signaling Pathway | 1.42 | 0.0643 | 2.121 | GNB4,PLCB2,GRB2,PRKCD,PIK3R5,ATF4,P2RY12,PRKCH,ADCY7 |
| Role of Cytokines in Mediating Communication between Immune Cells | 1.39 | 0.0926 | #NUM! | CXCL8,IL1A,IL18,IL1B,TNF |
| PDGF Signaling | 1.38 | 0.0729 | 2.646 | ABL2,GRB2,SPHK1,PIK3R5,JAK3,PDGFB,INPP5D |
| HGF Signaling | 1.38 | 0.0672 | 1.633 | ETS2,GRB2,PRKCD,CDKN1A,PIK3R5,PRKCH,MAP3K8,ELF1 |
| Glioma Signaling | 1.37 | 0.0667 | 2.449 | RB1,CALM1 (includes others),GRB2,PRKCD,CDKN1A,PIK3R5,PRKCH,PDGFB |
| Pancreatic Adenocarcinoma Signaling | 1.37 | 0.0667 | 2.646 | PLD4,HMOX1,RB1,TGFBR1,GRB2,CDKN1A,PIK3R5,JAK3 |
| Endothelin-1 Signaling | 1.33 | 0.0561 | 2.714 | PLBD1,PLD4,HMOX1,PLCB2,GRB2,PRKCD,PTGS1,PIK3R5,PRKCH,GNA13,ADCY7 |
| Acute Myeloid Leukemia Signaling | 1.33 | 0.0707 | 0 | CSF3R,RUNX1,GRB2,CSF2RA,PIK3R5,MAP2K3,CSF1R |
| Role of JAK1 and JAK3 in γc Cytokine Signaling | 1.33 | 0.0779 | #NUM! | BLNK,STAT6,GRB2,SYK,PIK3R5,JAK3 |
| Gαi Signaling | 1.32 | 0.065 | 1.134 | P2RY13,GNB4,GRB2,RGS10,ADORA3,P2RY12,ADCY7,FPR1 |
| Airway Pathology in Chronic Obstructive Pulmonary Disease | 1.32 | 0.25 | #NUM! | CXCL8,TNF |
| Huntington's Disease Signaling | 1.32 | 0.052 | 3 | GNB4,PLCB2,GRB2,SGK1,UBA52,PRKCD,HSPA1A/HSPA1B,VAMP3,PIK3R5,RPS27A,ATF4,PRKCH,UBC |
| Role of PI3K/AKT Signaling in the Pathogenesis of Influenza | 1.3 | 0.0759 | 0.447 | NFKBID,NFKBIA,GRB2,PIK3R5,CCL5,PLAC8 |
| NGF Signaling | 1.3 | 0.064 | 2.646 | ROCK1,RHOG,GRB2,PRKCD,PIK3R5,ATF4,MAP3K8,RPS6KA1 |
| Adrenomedullin signaling pathway | 1.3 | 0.055 | 2.714 | CALM1 (includes others),IL1A,PLCB2,IL18,C3,GRB2,PIK3R5,IL1B,MAP2K3,TNF,ADCY7 |
| RANK Signaling in Osteoclasts | 1.29 | 0.0686 | 2.236 | NFKBID,CALM1 (includes others),NFKBIA,GRB2,PIK3R5,MAP3K8,GSN |
| Molecular Mechanisms of Cancer | 1.28 | 0.0457 | #NUM! | NAIP,PLCB2,TGFBR1,GRB2,PIK3R5,RHOH,RB1,NFKBID,NFKBIA,RHOG,PRKCD,CDKN1A,PRKCH,MAP2K3,GNA13,CFLAR,JAK3,ADCY7 |
| Prostate Cancer Signaling | 1.27 | 0.068 | #NUM! | NFKBID,RB1,NFKBIA,GRB2,CDKN1A,PIK3R5,ATF4 |
| Oncostatin M Signaling | 1.26 | 0.1 | 2 | IL6ST,GRB2,OSM,JAK3 |
| Renin-Angiotensin Signaling | 1.25 | 0.0625 | 2.646 | PTPN6,GRB2,PRKCD,PIK3R5,PRKCH,CCL5,TNF,ADCY7 |
| ERK/MAPK Signaling | 1.25 | 0.0539 | 2.111 | H3F3A/H3F3B,ETS2,GRB2,DUSP1,PRKCD,MKNK1,PIK3R5,ATF4,RPS6KA1,DUSP2,ELF1 |
| Type II Diabetes Mellitus Signaling | 1.24 | 0.0584 | 2.828 | NFKBID,NFKBIA,GRB2,PRKCD,PIK3R5,PRKCH,TNFRSF1B,TNF,ACSL1 |
| PI3K/AKT Signaling | 1.21 | 0.0611 | 1.134 | NFKBID,NFKBIA,LIMS1,GRB2,CDKN1A,MAP3K8,JAK3,INPP5D |
| autophagy | 1.21 | 0.0806 | #NUM! | CTSL,CTSH,CTSS,CTSB,CTSC |
| Tumoricidal Function of Hepatic Natural Killer Cells | 1.21 | 0.125 | #NUM! | SERPINB9,ICAM1,SRGN |
| 3-phosphoinositide Degradation | 1.18 | 0.057 | 3 | PTPRC,PTPN6,NUDT16,DUSP1,PTPN1,PALD1,DUSP2,SIRPA,INPP5D |
| GDP-glucose Biosynthesis | 1.17 | 0.2 | #NUM! | HK1,HK2 |
| IL-17A Signaling in Gastric Cells | 1.17 | 0.12 | #NUM! | CXCL8,CCL5,TNF |
| Osteoarthritis Pathway | 1.17 | 0.0519 | 1.508 | TLR2,CXCL8,TGFBR1,DDIT4,PPARD,SPHK1,IL1B,ATF4,MEF2C,TNFRSF1B,TNF |
| Gαs Signaling | 1.17 | 0.0636 | 1.633 | GNB4,RGS2,HCK,ATF4,ADCY7,PTGER4,ADRB2 |
| PPARα/RXRα Activation | 1.17 | 0.0538 | 0.378 | NFKBID,PLCB2,NFKBIA,TGFBR1,GRB2,IL1B,MEF2C,MAP2K3,ADCY7,ABCA1 |
| Pyridoxal 5'-phosphate Salvage Pathway | 1.15 | 0.0769 | 2.236 | SGK1,PRKCD,PRKCH,MAP2K3,MAP3K8 |
| CCR3 Signaling in Eosinophils | 1.15 | 0.0588 | 2.236 | ROCK1,CALM1 (includes others),GNB4,PLCB2,GRB2,PRKCD,PIK3R5,PRKCH |
| Renal Cell Carcinoma Signaling | 1.14 | 0.0682 | #NUM! | GRB2,UBA52,PIK3R5,RPS27A,UBC,PDGFB |
| 14-3-3-mediated Signaling | 1.14 | 0.0584 | 2.646 | PLCB2,GRB2,PRKCD,PIK3R5,PRKCH,RPS6KA1,TUBB,TNF |
| UVB-Induced MAPK Signaling | 1.14 | 0.0758 | 2 | H3F3A/H3F3B,GRB2,PRKCD,PIK3R5,PRKCH |
| IL-9 Signaling | 1.14 | 0.0889 | 2 | GRB2,PIK3R5,JAK3,TNF |
| Glucose and Glucose-1-phosphate Degradation | 1.12 | 0.182 | #NUM! | HK1,HK2 |
| UDP-N-acetyl-D-galactosamine Biosynthesis II | 1.12 | 0.182 | #NUM! | HK1,HK2 |
| Lymphotoxin β Receptor Signaling | 1.12 | 0.0746 | 1 | NFKBID,NFKBIA,GRB2,PIK3R5,LTBR |
| CREB Signaling in Neurons | 1.12 | 0.0505 | 3 | CALM1 (includes others),GNB4,PLCB2,GRB2,PRKCD,PIK3R5,ATF4,PRKCH,RPS6KA1,GNA13,ADCY7 |
| Corticotropin Releasing Hormone Signaling | 1.12 | 0.0576 | 2.646 | CALM1 (includes others),PRKCD,MEF2A,NR4A1,ATF4,MEF2C,PRKCH,ADCY7 |
| Glioblastoma Multiforme Signaling | 1.08 | 0.0536 | 1.667 | RB1,PLCB2,RHOG,GRB2,PRKCD,CDKN1A,PIK3R5,RHOH,PDGFB |
| FLT3 Signaling in Hematopoietic Progenitor Cells | 1.07 | 0.0645 | 1.633 | STAT6,GRB2,PIK3R5,ATF4,RPS6KA1,INPP5D |
| Hematopoiesis from Pluripotent Stem Cells | 1.07 | 0.0833 | #NUM! | CXCL8,IL1A,CD4,FCER1G |
| D-myo-inositol (1,4,5,6)-Tetrakisphosphate Biosynthesis | 1.06 | 0.0556 | 2.828 | PTPRC,PTPN6,NUDT16,DUSP1,PTPN1,PALD1,DUSP2,SIRPA |
| D-myo-inositol (3,4,5,6)-tetrakisphosphate Biosynthesis | 1.06 | 0.0556 | 2.828 | PTPRC,PTPN6,NUDT16,DUSP1,PTPN1,PALD1,DUSP2,SIRPA |
| HER-2 Signaling in Breast Cancer | 1.06 | 0.0638 | #NUM! | ITGB2,GRB2,PRKCD,CDKN1A,PIK3R5,PRKCH |
| GNRH Signaling | 1.05 | 0.0526 | 2.828 | CALM1 (includes others),PLCB2,GRB2,PRKCD,ATF4,PRKCH,MAP2K3,MAP3K8,ADCY7 |
| eNOS Signaling | 1.04 | 0.0523 | 2.333 | CALM1 (includes others),LPAR6,GRB2,PRKCD,HSPA1A/HSPA1B,PIK3R5,LPAR5,PRKCH,ADCY7 |
| Melatonin Signaling | 1.03 | 0.0694 | 2.236 | CALM1 (includes others),PLCB2,PRKCD,PRKCH,MAP2K3 |
| Choline Biosynthesis III | 1.02 | 0.154 | #NUM! | PLD4,HMOX1 |
| Ephrin B Signaling | 1.01 | 0.0685 | 1 | ROCK1,GNB4,CXCR4,CAP1,GNA13 |
| Hereditary Breast Cancer Signaling | 0.988 | 0.0533 | #NUM! | RB1,GADD45B,GRB2,UBA52,CDKN1A,PIK3R5,RPS27A,UBC |
| Epithelial Adherens Junction Signaling | 0.988 | 0.0533 | #NUM! | ACTR2,MYH9,ACTR3,TGFBR1,ARPC1B,ARPC2,TUBB,ARPC4 |
| Docosahexaenoic Acid (DHA) Signaling | 0.984 | 0.0769 | #NUM! | GRB2,PIK3R5,IL1B,BCL2A1 |
| Glioma Invasiveness Signaling | 0.965 | 0.0658 | 2.236 | RHOG,GRB2,PIK3R5,PLAUR,RHOH |
| CD27 Signaling in Lymphocytes | 0.965 | 0.0755 | #NUM! | NFKBID,NFKBIA,MAP2K3,MAP3K8 |
| Semaphorin Signaling in Neurons | 0.965 | 0.0755 | #NUM! | ROCK1,SEMA4D,RHOG,RHOH |
| Chemokine Signaling | 0.947 | 0.0649 | 2.236 | CALM1 (includes others),PLCB2,CCL4,CXCR4,CCL5 |
| IL-17A Signaling in Airway Cells | 0.93 | 0.0641 | 0.447 | NFKBID,NFKBIA,GRB2,PIK3R5,JAK3 |
| STAT3 Pathway | 0.928 | 0.0583 | 0.816 | PTPN6,IL1A,TGFBR1,CDKN1A,IL1B,PDGFB |
| Glycogen Degradation III | 0.925 | 0.133 | #NUM! | GAA,TYMP |
| Melanocyte Development and Pigmentation Signaling | 0.916 | 0.0577 | 1.633 | PTPN6,GRB2,PIK3R5,ATF4,RPS6KA1,ADCY7 |
| Role of JAK2 in Hormone-like Cytokine Signaling | 0.912 | 0.0882 | #NUM! | PTPN6,PTPN1,SIRPA |
| Unfolded protein response | 0.908 | 0.0714 | #NUM! | PPP1R15A,HSPA1A/HSPA1B,ATF4,ATF6 |
| Antiproliferative Role of Somatostatin Receptor 2 | 0.89 | 0.0617 | 1 | GNB4,PTPN6,GRB2,CDKN1A,PIK3R5 |
| TWEAK Signaling | 0.89 | 0.0857 | #NUM! | NFKBID,NAIP,NFKBIA |
| IL-17A Signaling in Fibroblasts | 0.89 | 0.0857 | #NUM! | NFKBID,NFKBIA,NFKBIZ |
| Extrinsic Prothrombin Activation Pathway | 0.889 | 0.125 | #NUM! | F13A1,THBD |
| Neurotrophin/TRK Signaling | 0.858 | 0.0602 | 2.236 | GRB2,PIK3R5,ATF4,MAP2K3,RPS6KA1 |
| Diphthamide Biosynthesis | 0.828 | 0.333 | #NUM! | EEF2 |
| Small Cell Lung Cancer Signaling | 0.827 | 0.0588 | #NUM! | NFKBID,RB1,NFKBIA,GRB2,PIK3R5 |
| Melanoma Signaling | 0.816 | 0.0656 | 0 | RB1,GRB2,CDKN1A,PIK3R5 |
| UVA-Induced MAPK Signaling | 0.816 | 0.0536 | 2 | PLCB2,GRB2,PIK3R5,ZC3HAV1,RPS6KA1,PARP14 |
| Regulation of IL-2 Expression in Activated and Anergic T Lymphocytes | 0.816 | 0.0581 | #NUM! | NFKBID,CALM1 (includes others),NFKBIA,TGFBR1,GRB2 |
| Aldosterone Signaling in Epithelial Cells | 0.807 | 0.0476 | 2.449 | PLCB2,GRB2,SGK1,DUSP1,PRKCD,HSPA1A/HSPA1B,PIK3R5,PRKCH |
| Retinoic acid Mediated Apoptosis Signaling | 0.807 | 0.0645 | 1 | ZC3HAV1,TNFRSF10D,CFLAR,PARP14 |
| Phospholipases | 0.807 | 0.0645 | 2 | PLBD1,PLD4,HMOX1,PLCB2 |
| ILK Signaling | 0.807 | 0.0457 | 2.828 | ITGB2,MYH9,RHOG,LIMS1,GRB2,PIK3R5,ATF4,TNF,RHOH |
| April Mediated Signaling | 0.802 | 0.0769 | #NUM! | NFKBID,NFKBIA,TNFSF13 |
| GADD45 Signaling | 0.781 | 0.105 | #NUM! | GADD45B,CDKN1A |
| Telomerase Signaling | 0.762 | 0.0513 | 2 | RB1,ETS2,GRB2,CDKN1A,PIK3R5,ELF1 |
| FGF Signaling | 0.752 | 0.0549 | 1.342 | PTPN6,GRB2,PIK3R5,ATF4,MAP2K3 |
| The Visual Cycle | 0.749 | 0.1 | #NUM! | DHRS3,DHRS9 |
| Airway Inflammation in Asthma | 0.741 | 0.25 | #NUM! | TNF |
| Heme Degradation | 0.741 | 0.25 | #NUM! | HMOX1 |
| Spermine and Spermidine Degradation I | 0.741 | 0.25 | #NUM! | SAT1 |
| Acetate Conversion to Acetyl-CoA | 0.741 | 0.25 | #NUM! | ACSL1 |
| Xenobiotic Metabolism Signaling | 0.738 | 0.0405 | #NUM! | HMOX1,IL1A,FTL,MGST2,GRB2,PRKCD,PIK3R5,IL1B,PRKCH,MAP2K3,MAP3K8,TNF |
| G Beta Gamma Signaling | 0.737 | 0.05 | 2.236 | BTK,GNB4,GRB2,PRKCD,PRKCH,GNA13 |
| α-Adrenergic Signaling | 0.737 | 0.0538 | #NUM! | CALM1 (includes others),GNB4,PRKCD,PRKCH,ADCY7 |
| Endoplasmic Reticulum Stress Pathway | 0.729 | 0.0952 | #NUM! | ATF4,ATF6 |
| Role of p14/p19ARF in Tumor Suppression | 0.729 | 0.0698 | #NUM! | RB1,GRB2,PIK3R5 |
| GABA Receptor Signaling | 0.713 | 0.0526 | #NUM! | UBA52,RPS27A,AP1B1,UBC,ADCY7 |
| Sumoylation Pathway | 0.701 | 0.0521 | #NUM! | NFKBIA,RHOG,RHOH,ARHGDIB,SP100 |
| CNTF Signaling | 0.695 | 0.0571 | 2 | IL6ST,GRB2,PIK3R5,RPS6KA1 |
| IL-2 Signaling | 0.695 | 0.0571 | 2 | GRB2,SYK,PIK3R5,JAK3 |
| Salvage Pathways of Pyrimidine Ribonucleotides | 0.692 | 0.0515 | 2.236 | SGK1,PRKCD,PRKCH,MAP2K3,MAP3K8 |
| Thrombopoietin Signaling | 0.683 | 0.0563 | 2 | GRB2,PRKCD,PIK3R5,PRKCH |
| TR/RXR Activation | 0.683 | 0.051 | #NUM! | SLC16A3,UCP2,GRB2,PIK3R5,NCOA4 |
| Synaptic Long Term Potentiation | 0.683 | 0.0476 | 2.449 | CALM1 (includes others),PLCB2,PRKCD,ATF4,PRKCH,RPS6KA1 |
| Role of Oct4 in Mammalian Embryonic Stem Cell Pluripotency | 0.68 | 0.0652 | #NUM! | RB1,ETS2,MEF2A |
| Protein Citrullination | 0.678 | 0.2 | #NUM! | PADI2 |
| Tetrahydrofolate Salvage from 5,10-methenyltetrahydrofolate | 0.678 | 0.2 | #NUM! | MTHFD2 |
| Ceramide Signaling | 0.677 | 0.0505 | 0.447 | GRB2,SPHK1,PIK3R5,TNFRSF1B,TNF |
| nNOS Signaling in Neurons | 0.666 | 0.0638 | #NUM! | CALM1 (includes others),PRKCD,PRKCH |
| Relaxin Signaling | 0.661 | 0.0443 | 0.447 | NFKBID,GNB4,NFKBIA,GRB2,PIK3R5,GNA13,ADCY7 |
| Role of JAK1, JAK2 and TYK2 in Interferon Signaling | 0.661 | 0.0833 | #NUM! | PTPN6,IFNGR1 |
| Glutathione Redox Reactions I | 0.661 | 0.0833 | #NUM! | MGST2,GPX1 |
| Role of JAK family kinases in IL-6-type Cytokine Signaling | 0.634 | 0.08 | #NUM! | IL6ST,OSM |
| ErbB Signaling | 0.623 | 0.0481 | 2.236 | GRB2,PRKCD,PIK3R5,PRKCH,MAP2K3 |
| Chondroitin and Dermatan Biosynthesis | 0.62 | 0.167 | #NUM! | CSGALNACT1 |
| Lactose Degradation III | 0.62 | 0.167 | #NUM! | PSAP |
| Estrogen-mediated S-phase Entry | 0.613 | 0.0769 | #NUM! | RB1,CDKN1A |
| Role of MAPK Signaling in the Pathogenesis of Influenza | 0.605 | 0.0513 | #NUM! | PLBD1,MAP2K3,CCL5,TNF |
| ErbB4 Signaling | 0.605 | 0.0513 | 2 | GRB2,PRKCD,PIK3R5,PRKCH |
| PAK Signaling | 0.597 | 0.0467 | 2.236 | GRB2,PIK3R5,TNF,PDGFB,MYL12A |
| D-myo-inositol (1,4,5)-Trisphosphate Biosynthesis | 0.594 | 0.0741 | #NUM! | PLD4,PLCB2 |
| Androgen Signaling | 0.593 | 0.0438 | 2 | CALM1 (includes others),GNB4,PRKCD,PRKCH,GNA13,NCOA4 |
| VEGF Signaling | 0.579 | 0.0459 | 1 | ROCK1,PTPN6,GRB2,PIK3R5,EIF2S3 |
| Ceramide Biosynthesis | 0.575 | 0.143 | #NUM! | SPTLC2 |
| Adenine and Adenosine Salvage III | 0.575 | 0.143 | #NUM! | ADA2 |
| Ceramide Degradation | 0.575 | 0.143 | #NUM! | ASAH1 |
| p53 Signaling | 0.563 | 0.045 | 0 | RB1,GADD45B,GRB2,CDKN1A,PIK3R5 |
| Aryl Hydrocarbon Receptor Signaling | 0.563 | 0.0426 | 0.447 | RB1,IL1A,MGST2,CDKN1A,IL1B,TNF |
| Chronic Myeloid Leukemia Signaling | 0.557 | 0.0446 | #NUM! | RB1,TGFBR1,GRB2,CDKN1A,PIK3R5 |
| GPCR-Mediated Nutrient Sensing in Enteroendocrine Cells | 0.557 | 0.0446 | 2.236 | PLCB2,PRKCD,LPAR5,PRKCH,ADCY7 |
| Non-Small Cell Lung Cancer Signaling | 0.557 | 0.0482 | #NUM! | RB1,GRB2,PIK3R5,RASSF5 |
| Nitric Oxide Signaling in the Cardiovascular System | 0.549 | 0.0442 | 2.236 | CALM1 (includes others),GRB2,PRKCD,PIK3R5,PRKCH |
| Angiopoietin Signaling | 0.548 | 0.0476 | #NUM! | NFKBID,NFKBIA,GRB2,PIK3R5 |
| Histidine Degradation III | 0.54 | 0.125 | #NUM! | MTHFD2 |
| Superoxide Radicals Degradation | 0.54 | 0.125 | #NUM! | SOD2 |
| Purine Ribonucleosides Degradation to Ribose-1-phosphate | 0.54 | 0.125 | #NUM! | ADA2 |
| Salvage Pathways of Pyrimidine Deoxyribonucleotides | 0.54 | 0.125 | #NUM! | TYMP |
| Neuropathic Pain Signaling In Dorsal Horn Neurons | 0.538 | 0.0435 | 2.236 | PLCB2,GRB2,PRKCD,PIK3R5,PRKCH |
| Estrogen-Dependent Breast Cancer Signaling | 0.534 | 0.0465 | #NUM! | GRB2,HSD17B11,PIK3R5,ATF4 |
| Leptin Signaling in Obesity | 0.534 | 0.0465 | #NUM! | PLCB2,GRB2,PIK3R5,ADCY7 |
| Glutathione-mediated Detoxification | 0.532 | 0.0645 | #NUM! | MGST2,HPGDS |
| 4-1BB Signaling in T Lymphocytes | 0.515 | 0.0625 | #NUM! | NFKBID,NFKBIA |
| G Protein Signaling Mediated by Tubby | 0.515 | 0.0625 | #NUM! | GNB4,PLCB2 |
| Paxillin Signaling | 0.51 | 0.042 | #NUM! | ITGB2,ITGAM,GRB2,PIK3R5,ITGAX |
| Sphingosine and Sphingosine-1-phosphate Metabolism | 0.51 | 0.111 | #NUM! | ASAH1 |
| Folate Transformations I | 0.51 | 0.111 | #NUM! | MTHFD2 |
| Prolactin Signaling | 0.51 | 0.0449 | 2 | GRB2,PRKCD,PIK3R5,PRKCH |
| Ephrin A Signaling | 0.51 | 0.05 | #NUM! | ROCK1,GRB2,PIK3R5 |
| AMPK Signaling | 0.495 | 0.037 | 1.89 | PFKFB3,EEF2,GRB2,CDKN1A,PIK3R5,ATF4,MAP2K3,ADRB2 |
| IL-17 Signaling | 0.493 | 0.044 | #NUM! | CXCL8,GRB2,PIK3R5,MAP2K3 |
| IL-7 Signaling Pathway | 0.493 | 0.044 | 2 | GRB2,LYN,PIK3R5,JAK3 |
| Circadian Rhythm Signaling | 0.49 | 0.0588 | #NUM! | BHLHE41,ATF4 |
| Retinoate Biosynthesis I | 0.49 | 0.0588 | #NUM! | DHRS3,DHRS9 |
| Factors Promoting Cardiogenesis in Vertebrates | 0.487 | 0.0435 | #NUM! | TGFBR1,PRKCD,MEF2C,PRKCH |
| Activation of IRF by Cytosolic Pattern Recognition Receptors | 0.481 | 0.0476 | #NUM! | NFKBID,NFKBIA,TNF |
| Regulation of Cellular Mechanics by Calpain Protease | 0.481 | 0.0476 | #NUM! | RB1,GRB2,EZR |
| PTEN Signaling | 0.472 | 0.04 | -0.447 | TGFBR1,GRB2,CDKN1A,PIK3R5,INPP5D |
| VEGF Family Ligand-Receptor Interactions | 0.472 | 0.0426 | 2 | GRB2,PRKCD,PIK3R5,PRKCH |
| RAR Activation | 0.465 | 0.0368 | #NUM! | DHRS3,DHRS9,DUSP1,PRKCD,PRKCH,REL,ADCY7 |
| Interferon Signaling | 0.465 | 0.0556 | #NUM! | IFNGR1,IFITM2 |
| Sperm Motility | 0.459 | 0.0394 | 2.236 | PLBD1,CALM1 (includes others),PLCB2,PRKCD,PRKCH |
| Cell Cycle Regulation by BTG Family Proteins | 0.452 | 0.0541 | #NUM! | RB1,BTG2 |
| Ethanol Degradation II | 0.452 | 0.0541 | #NUM! | DHRS9,ACSL1 |
| Cell Cycle: G1/S Checkpoint Regulation | 0.442 | 0.0448 | #NUM! | RB1,CDKN1A,RPL5 |
| ATM Signaling | 0.442 | 0.0408 | #NUM! | NFKBIA,GADD45B,CDKN1A,ATF4 |
| Cellular Effects of Sildenafil (Viagra) | 0.432 | 0.0382 | #NUM! | CALM1 (includes others),PLCB2,MYH9,ADCY7,MYL12A |
| Dopamine-DARPP32 Feedback in cAMP Signaling | 0.429 | 0.0366 | 2.449 | CALM1 (includes others),PLCB2,PRKCD,ATF4,PRKCH,ADCY7 |
| Gap Junction Signaling | 0.407 | 0.0348 | #NUM! | PLCB2,GRB2,PRKCD,PIK3R5,PRKCH,TUBB,ADCY7 |
| Role of IL-17A in Psoriasis | 0.407 | 0.0769 | #NUM! | CXCL8 |
| Fatty Acid Activation | 0.407 | 0.0769 | #NUM! | ACSL1 |
| Glycogen Degradation II | 0.407 | 0.0769 | #NUM! | TYMP |
| B Cell Activating Factor Signaling | 0.404 | 0.0488 | #NUM! | NFKBID,NFKBIA |
| Mechanisms of Viral Exit from Host Cells | 0.404 | 0.0488 | #NUM! | PRKCD,PRKCH |
| Intrinsic Prothrombin Activation Pathway | 0.393 | 0.0476 | #NUM! | F13A1,THBD |
| Retinol Biosynthesis | 0.393 | 0.0476 | #NUM! | DHRS3,DHRS9 |
| GPCR-Mediated Integration of Enteroendocrine Signaling Exemplified by an L Cell | 0.393 | 0.0411 | #NUM! | PLCB2,ADCY7,ADRB2 |
| ErbB2-ErbB3 Signaling | 0.376 | 0.04 | #NUM! | GRB2,PIK3R5,JAK3 |
| Hypoxia Signaling in the Cardiovascular System | 0.376 | 0.04 | #NUM! | NFKBID,NFKBIA,ATF4 |
| Role of RIG1-like Receptors in Antiviral Innate Immunity | 0.373 | 0.0455 | #NUM! | NFKBID,NFKBIA |
| Stearate Biosynthesis I (Animals) | 0.373 | 0.0455 | #NUM! | TBXAS1,ACSL1 |
| Human Embryonic Stem Cell Pluripotency | 0.363 | 0.035 | #NUM! | TGFBR1,GRB2,SPHK1,PIK3R5,PDGFB |
| Pyrimidine Ribonucleotides Interconversion | 0.363 | 0.0444 | #NUM! | ENTPD1,NUDT16 |
| SAPK/JNK Signaling | 0.363 | 0.0364 | 2 | GRB2,FCER1G,PIK3R5,GNA13 |
| Amyotrophic Lateral Sclerosis Signaling | 0.356 | 0.036 | 0 | NAIP,GRB2,GPX1,PIK3R5 |
| Chondroitin Sulfate Degradation (Metazoa) | 0.354 | 0.0625 | #NUM! | GM2A |
| Adenosine Nucleotides Degradation II | 0.354 | 0.0625 | #NUM! | ADA2 |
| Mouse Embryonic Stem Cell Pluripotency | 0.354 | 0.0357 | 2 | IL6ST,GRB2,PIK3R5,JAK3 |
| Synaptic Long Term Depression | 0.354 | 0.0333 | 2.449 | PLBD1,PLCB2,PRKCD,LYN,PRKCH,GNA13 |
| Insulin Receptor Signaling | 0.349 | 0.034 | 0.447 | GRB2,SGK1,PTPN1,PIK3R5,INPP5D |
| Pyrimidine Ribonucleotides De Novo Biosynthesis | 0.349 | 0.0426 | #NUM! | ENTPD1,NUDT16 |
| γ-linolenate Biosynthesis II (Animals) | 0.342 | 0.0588 | #NUM! | ACSL1 |
| Dermatan Sulfate Degradation (Metazoa) | 0.342 | 0.0588 | #NUM! | GM2A |
| Mitochondrial L-carnitine Shuttle Pathway | 0.342 | 0.0588 | #NUM! | ACSL1 |
| GDNF Family Ligand-Receptor Interactions | 0.333 | 0.0366 | #NUM! | GRB2,PIK3R5,DOK3 |
| UVC-Induced MAPK Signaling | 0.333 | 0.0408 | #NUM! | PRKCD,PRKCH |
| D-myo-inositol (1,4,5)-trisphosphate Degradation | 0.326 | 0.0556 | #NUM! | INPP5D |
| Cell Cycle: G2/M DNA Damage Checkpoint Regulation | 0.324 | 0.04 | #NUM! | CDKN1A,RPS6KA1 |
| Gustation Pathway | 0.316 | 0.0325 | #NUM! | P2RY13,PLCB2,LPAR6,P2RY12,ADCY7 |
| Neuroprotective Role of THOP1 in Alzheimer's Disease | 0.316 | 0.0333 | #NUM! | HLA-C,HLA-B,HLA-F,HLA-E |
| 1D-myo-inositol Hexakisphosphate Biosynthesis II (Mammalian) | 0.316 | 0.0526 | #NUM! | INPP5D |
| Purine Nucleotides Degradation II (Aerobic) | 0.316 | 0.0526 | #NUM! | ADA2 |
| D-myo-inositol (1,3,4)-trisphosphate Biosynthesis | 0.316 | 0.0526 | #NUM! | INPP5D |
| Protein Ubiquitination Pathway | 0.301 | 0.0302 | #NUM! | B2M,USP4,HLA-C,UBA52,HLA-B,HSPA1A/HSPA1B,RPS27A,UBC |
| Cardiomyocyte Differentiation via BMP Receptors | 0.301 | 0.05 | #NUM! | MEF2C |
| Transcriptional Regulatory Network in Embryonic Stem Cells | 0.296 | 0.037 | #NUM! | H3F3A/H3F3B,SKIL |
| Triacylglycerol Degradation | 0.296 | 0.037 | #NUM! | DAGLB,ABHD16A |
| Oxidative Ethanol Degradation III | 0.289 | 0.0476 | #NUM! | ACSL1 |
| Polyamine Regulation in Colon Cancer | 0.276 | 0.0455 | #NUM! | SAT1 |
| Role of NANOG in Mammalian Embryonic Stem Cell Pluripotency | 0.276 | 0.0312 | 2 | IL6ST,GRB2,PIK3R5,JAK3 |
| Glutamate Receptor Signaling | 0.274 | 0.0351 | #NUM! | CALM1 (includes others),SLC1A3 |
| TGF-β Signaling | 0.272 | 0.0323 | #NUM! | TGFBR1,GRB2,MAP2K3 |
| Neuregulin Signaling | 0.269 | 0.0319 | #NUM! | GRB2,PRKCD,PRKCH |
| Bladder Cancer Signaling | 0.269 | 0.0319 | #NUM! | CXCL8,RB1,CDKN1A |
| Putrescine Degradation III | 0.269 | 0.0435 | #NUM! | SAT1 |
| Tight Junction Signaling | 0.269 | 0.0299 | #NUM! | MYH9,TGFBR1,VAMP3,TNFRSF1B,TNF |
| CDP-diacylglycerol Biosynthesis I | 0.259 | 0.0417 | #NUM! | LPCAT2 |
| Superpathway of D-myo-inositol (1,4,5)-trisphosphate Metabolism | 0.259 | 0.0417 | #NUM! | INPP5D |
| Wnt/β-catenin Signaling | 0.249 | 0.0291 | #NUM! | TGFBR1,UBA52,PPARD,RPS27A,UBC |
| Ethanol Degradation IV | 0.249 | 0.04 | #NUM! | ACSL1 |
| Phosphatidylglycerol Biosynthesis II (Non-plastidic) | 0.24 | 0.0385 | #NUM! | LPCAT2 |
| Wnt/Ca+ pathway | 0.24 | 0.0317 | #NUM! | PLCB2,ATF4 |
| Sirtuin Signaling Pathway | 0.224 | 0.0274 | 0.378 | CXCL8,PFKFB3,GADD45B,H3F3A/H3F3B,SOD2,UCP2,TNF,ABCA1 |
| IL-15 Production | 0.22 | 0.0357 | #NUM! | JAK3 |
| EGF Signaling | 0.209 | 0.0294 | #NUM! | GRB2,PIK3R5 |
| Sertoli Cell-Sertoli Cell Junction Signaling | 0.207 | 0.0272 | #NUM! | MAP2K3,MAP3K8,TUBB,TNF,A2M |
| Sonic Hedgehog Signaling | 0.204 | 0.0333 | #NUM! | ARRB2 |
| Endometrial Cancer Signaling | 0.201 | 0.0286 | #NUM! | GRB2,PIK3R5 |
| Fatty Acid β-oxidation I | 0.189 | 0.0312 | #NUM! | ACSL1 |
| DNA Methylation and Transcriptional Repression Signaling | 0.175 | 0.0294 | #NUM! | H3F3A/H3F3B |
| Inhibition of Angiogenesis by TSP1 | 0.175 | 0.0294 | #NUM! | TGFBR1 |
| Mitochondrial Dysfunction | 0 | 0.0117 | #NUM! | SOD2,UCP2 |
| PXR/RXR Activation | 0 | 0.0154 | #NUM! | TNF |
| Role of BRCA1 in DNA Damage Response | 0 | 0.025 | #NUM! | RB1,CDKN1A |
| HIF1α Signaling | 0 | 0.0242 | #NUM! | SLC2A5,GRB2,PIK3R5 |
| Agrin Interactions at Neuromuscular Junction | 0 | 0.0133 | #NUM! | ITGB2 |
| CDK5 Signaling | 0 | 0.019 | #NUM! | FOSB,ADCY7 |
| Mitotic Roles of Polo-Like Kinase | 0 | 0.0152 | #NUM! | PLK3 |
| Role of CHK Proteins in Cell Cycle Checkpoint Control | 0 | 0.0175 | #NUM! | CDKN1A |
| Thyroid Cancer Signaling | 0 | 0.0217 | #NUM! | CXCL8 |
| Myc Mediated Apoptosis Signaling | 0 | 0.0263 | #NUM! | GRB2,PIK3R5 |
| FAK Signaling | 0 | 0.019 | #NUM! | GRB2,PIK3R5 |
| Ovarian Cancer Signaling | 0 | 0.0267 | #NUM! | RB1,GRB2,PTGS1,PIK3R5 |
| Role of Wnt/GSK-3β Signaling in the Pathogenesis of Influenza | 0 | 0.013 | #NUM! | NCOA4 |
| Cyclins and Cell Cycle Regulation | 0 | 0.0247 | #NUM! | RB1,CDKN1A |
| Cell Cycle Control of Chromosomal Replication | 0 | 0.0179 | #NUM! | ORC4 |
| nNOS Signaling in Skeletal Muscle Cells | 0 | 0.0244 | #NUM! | CALM1 (includes others) |
| Thyroid Hormone Metabolism II (via Conjugation and/or Degradation) | 0 | 0.0233 | #NUM! | CSGALNACT1 |
| Nicotine Degradation III | 0 | 0.0179 | #NUM! | CSGALNACT1 |
| Melatonin Degradation I | 0 | 0.0154 | #NUM! | CSGALNACT1 |
| Estrogen Biosynthesis | 0 | 0.0244 | #NUM! | HSD17B11 |
| Chondroitin Sulfate Biosynthesis | 0 | 0.0175 | #NUM! | CSGALNACT1 |
| Dermatan Sulfate Biosynthesis | 0 | 0.0169 | #NUM! | CSGALNACT1 |
| Nicotine Degradation II | 0 | 0.0154 | #NUM! | CSGALNACT1 |
| Serotonin Degradation | 0 | 0.026 | #NUM! | DHRS9,CSGALNACT1 |
| Triacylglycerol Biosynthesis | 0 | 0.0217 | #NUM! | LPCAT2 |
| Chondroitin Sulfate Biosynthesis (Late Stages) | 0 | 0.0204 | #NUM! | CSGALNACT1 |
| Superpathway of Melatonin Degradation | 0 | 0.0143 | #NUM! | CSGALNACT1 |
| Noradrenaline and Adrenaline Degradation | 0 | 0.025 | #NUM! | DHRS9 |
| Inhibition of Matrix Metalloproteases | 0 | 0.0256 | #NUM! | A2M |
| Regulation of the Epithelial-Mesenchymal Transition Pathway | 0 | 0.0256 | #NUM! | TGFBR1,GRB2,PIK3R5,MAP2K3,JAK3 |
| Adipogenesis pathway | 0 | 0.0224 | #NUM! | RB1,RPS6KA1,TNF |
| HIPPO signaling | 0 | 0.0115 | #NUM! | MOB1A |
| PCP pathway | 0 | 0.0164 | #NUM! | ROCK1 |
| Estrogen Receptor Signaling | 0 | 0.0224 | #NUM! | DDX5,H3F3A/H3F3B,GRB2 |
| Cardiac β-adrenergic Signaling | 0 | 0.0213 | #NUM! | GNB4,AKAP13,ADCY7 |
| Serotonin Receptor Signaling | 0 | 0.0233 | #NUM! | ADCY7 |
| IGF-1 Signaling | 0 | 0.0179 | #NUM! | GRB2,PIK3R5 |
| Dopamine Receptor Signaling | 0 | 0.013 | #NUM! | ADCY7 |
| BMP signaling pathway | 0 | 0.0122 | #NUM! | GRB2 |
| Calcium Signaling | 0 | 0.0243 | 2 | CALM1 (includes others),MYH9,MEF2A,ATF4,MEF2C |

| Cluster 6 |  |  |  |  |
| --- | --- | --- | --- | --- |
| © 2000-2018 QIAGEN. All rights reserved. |  |  |  |  |
| Ingenuity Canonical Pathways | -log(B-H p-value) | Ratio | z-score | Molecules |
| Oxidative Phosphorylation | 3.18 | 0.0826 | 3 | NDUFS5,NDUFA7,NDUFB7,ATP5MF,NDUFS6,NDUFA3,NDUFAB1,NDUFB2,ATP5F1E |
| Sirtuin Signaling Pathway | 3.18 | 0.0479 | -0.577 | TOMM40,EPAS1,NDUFA7,TUBA4A,ATP5F1E,NDUFS5,SOD2,GADD45A,NDUFB7,NDUFS6,NAMPT,NDUFA3,NDUFAB1,NDUFB2 |
| Mitochondrial Dysfunction | 2.7 | 0.0585 | #NUM! | NDUFS5,SOD2,NDUFA7,NDUFB7,ATP5MF,NDUFS6,NDUFA3,NDUFAB1,NDUFB2,ATP5F1E |
| Oncostatin M Signaling | 2.24 | 0.125 | 2 | MT2A,SHC1,EPAS1,OSMR,CHI3L1 |
| Caveolar-mediated Endocytosis Signaling | 2.09 | 0.0845 | #NUM! | FLNA,HLA-A,CAV1,ITGA7,CAVIN1,EGFR |
| Acute Phase Response Signaling | 1.48 | 0.0455 | 2.449 | C1R,SHC1,NFKBIA,SOD2,TNFRSF1A,C1S,OSMR,SERPINA3 |
| Spermine and Spermidine Degradation I | 1.48 | 0.5 | #NUM! | SMOX,SAT1 |
| ILK Signaling | 1.23 | 0.0406 | 1.134 | PPP2CB,RHOC,FLNA,TNFRSF1A,VIM,TMSB10/TMSB4X,PPP1R14B,ACTN1 |
| Glioma Signaling | 1.19 | 0.05 | 2 | CDKN2A,SHC1,CALM1 (includes others),CAMK1,CDKN2B,EGFR |
| Breast Cancer Regulation by Stathmin1 | 1.19 | 0.0379 | #NUM! | SHC1,PPP2CB,CALM1 (includes others),CAMK1,TUBB6,TUBB2A,TUBA4A,PPP1R14B |
| HIPPO signaling | 1.19 | 0.0575 | -1 | PPP2CB,WWTR1,CD44,SFN,PPP1R14B |
| Atherosclerosis Signaling | 1.19 | 0.0472 | #NUM! | COL1A2,CSF1,PLA2G5,F3,CLU,TNFRSF12A |
| Glycolysis I | 1.19 | 0.115 | #NUM! | PKM,ENO2,GAPDH |
| Gluconeogenesis I | 1.19 | 0.115 | #NUM! | ENO2,GAPDH,MDH2 |
| 14-3-3-mediated Signaling | 1.07 | 0.0438 | #NUM! | TUBB6,TNFRSF1A,TUBB2A,TUBA4A,VIM,SFN |
| Phagosome Maturation | 0.934 | 0.0405 | #NUM! | DYNLL1,TUBB6,HLA-A,TUBB2A,TUBA4A,PRDX6 |
| Remodeling of Epithelial Adherens Junctions | 0.879 | 0.058 | #NUM! | TUBB6,TUBB2A,TUBA4A,ACTN1 |
| IGF-1 Signaling | 0.855 | 0.0446 | #NUM! | SHC1,IGFBP7,CYR61,SFN,IGFBP2 |
| Neuroprotective Role of THOP1 in Alzheimer's Disease | 0.774 | 0.0417 | 1 | C1R,HLA-A,TPP1,SERPINA3,HTRA1 |
| Integrin Signaling | 0.774 | 0.032 | 2.646 | SHC1,RHOC,CAV1,CAPN2,ITGA7,ACTN1,MYL12A |
| Germ Cell-Sertoli Cell Junction Signaling | 0.667 | 0.0335 | #NUM! | TUBB6,RHOC,TNFRSF1A,TUBB2A,TUBA4A,ACTN1 |
| Granulocyte Adhesion and Diapedesis | 0.665 | 0.0331 | #NUM! | HRH1,SDC2,TNFRSF1A,CXCL14,MSN,HSPB1 |
| Hepatic Fibrosis / Hepatic Stellate Cell Activation | 0.625 | 0.0321 | #NUM! | COL1A2,TIMP1,CSF1,TNFRSF1A,COL8A1,EGFR |
| Cell Cycle: G2/M DNA Damage Checkpoint Regulation | 0.625 | 0.06 | #NUM! | CDKN2A,GADD45A,SFN |
| Death Receptor Signaling | 0.605 | 0.043 | 0 | NFKBIA,TNFRSF1A,LMNA,HSPB1 |
| Production of Nitric Oxide and Reactive Oxygen Species in Macrophages | 0.604 | 0.0309 | 0.816 | PPP2CB,NFKBIA,RHOC,TNFRSF1A,PPP1R14B,CLU |
| Apoptosis Signaling | 0.601 | 0.0417 | 2 | NFKBIA,TNFRSF1A,LMNA,CAPN2 |
| NRF2-mediated Oxidative Stress Response | 0.601 | 0.0302 | 2 | MGST1,SOD2,JUNB,CDC34,SQSTM1,CBR1 |
| Spermidine Biosynthesis I | 0.601 | 0.5 | #NUM! | SRM |
| Epithelial Adherens Junction Signaling | 0.574 | 0.0333 | #NUM! | TUBB6,TUBB2A,TUBA4A,ACTN1,EGFR |
| Putrescine Degradation III | 0.559 | 0.087 | #NUM! | SMOX,SAT1 |
| Glutathione Redox Reactions I | 0.542 | 0.0833 | #NUM! | MGST1,PRDX6 |
| Osteoarthritis Pathway | 0.542 | 0.0283 | 2 | EPAS1,TNFRSF1A,ANXA2,NAMPT,HTRA1,SLC39A8 |
| Regulation of Cellular Mechanics by Calpain Protease | 0.514 | 0.0476 | #NUM! | CAPN2,ACTN1,EGFR |
| NADH Repair | 0.509 | 0.333 | #NUM! | GAPDH |
| LPS/IL-1 Mediated Inhibition of RXR Function | 0.499 | 0.027 | #NUM! | CHST2,MGST1,TNFRSF1A,SMOX,FABP7,FABP5 |
| Calcium-induced T Lymphocyte Apoptosis | 0.499 | 0.0455 | #NUM! | CALM1 (includes others),HLA-A,CAPN2 |
| Telomerase Signaling | 0.473 | 0.0342 | #NUM! | SHC1,PPP2CB,TPP1,EGFR |
| Actin Cytoskeleton Signaling | 0.473 | 0.0258 | 2.236 | SHC1,FLNA,TMSB10/TMSB4X,ACTN1,MSN,MYL12A |
| Heme Degradation | 0.473 | 0.25 | #NUM! | BLVRB |
| Melatonin Degradation II | 0.473 | 0.25 | #NUM! | SMOX |
| Fatty Acid β-oxidation III (Unsaturated, Odd Number) | 0.473 | 0.25 | #NUM! | ECI2 |
| T Helper Cell Differentiation | 0.473 | 0.0411 | #NUM! | TNFRSF1A,HLA-A,BCL6 |
| iCOS-iCOSL Signaling in T Helper Cells | 0.473 | 0.0325 | #NUM! | SHC1,CALM1 (includes others),NFKBIA,HLA-A |
| Myc Mediated Apoptosis Signaling | 0.473 | 0.0395 | #NUM! | CDKN2A,SHC1,SFN |
| Glioma Invasiveness Signaling | 0.473 | 0.0395 | #NUM! | TIMP1,RHOC,CD44 |
| Sertoli Cell-Sertoli Cell Junction Signaling | 0.473 | 0.0272 | #NUM! | TUBB6,TNFRSF1A,TUBB2A,TUBA4A,ACTN1 |
| Dopamine Receptor Signaling | 0.473 | 0.039 | #NUM! | PPP2CB,SMOX,PPP1R14B |
| Role of Tissue Factor in Cancer | 0.473 | 0.0308 | #NUM! | CSF1,CYR61,F3,EGFR |
| Serine Biosynthesis | 0.473 | 0.2 | #NUM! | PSPH |
| PI3K/AKT Signaling | 0.473 | 0.0305 | 0 | SHC1,PPP2CB,NFKBIA,SFN |
| TWEAK Signaling | 0.473 | 0.0571 | #NUM! | NFKBIA,TNFRSF12A |
| MIF-mediated Glucocorticoid Regulation | 0.473 | 0.0571 | #NUM! | NFKBIA,PLA2G5 |
| IL-17A Signaling in Fibroblasts | 0.473 | 0.0571 | #NUM! | NFKBIA,CEBPD |
| Huntington's Disease Signaling | 0.473 | 0.024 | #NUM! | SHC1,HAP1,CAPN2,POLR2L,ATP5F1E,EGFR |
| Cyclins and Cell Cycle Regulation | 0.47 | 0.037 | #NUM! | CDKN2A,PPP2CB,CDKN2B |
| IL-6 Signaling | 0.47 | 0.0299 | 2 | SHC1,NFKBIA,TNFRSF1A,HSPB1 |
| Role of Macrophages, Fibroblasts and Endothelial Cells in Rheumatoid Arthritis | 0.47 | 0.0219 | #NUM! | SFRP4,CALM1 (includes others),NFKBIA,CSF1,TNFRSF1A,IL32,CEBPD |
| Dendritic Cell Maturation | 0.47 | 0.0258 | 2.236 | COL1A2,NFKBIA,TNFRSF1A,HLA-A,IL32 |
| Complement System | 0.47 | 0.0541 | #NUM! | C1R,C1S |
| p70S6K Signaling | 0.47 | 0.029 | #NUM! | SHC1,PPP2CB,SFN,EGFR |
| Chondroitin and Dermatan Biosynthesis | 0.47 | 0.167 | #NUM! | CHPF |
| NAD Biosynthesis III | 0.47 | 0.167 | #NUM! | NAMPT |
| Inhibition of Matrix Metalloproteases | 0.458 | 0.0513 | #NUM! | TIMP1,SDC2 |
| Gap Junction Signaling | 0.458 | 0.0249 | #NUM! | TUBB6,TUBB2A,TUBA4A,CAV1,EGFR |
| Protein Ubiquitination Pathway | 0.458 | 0.0226 | #NUM! | CRYAB,STUB1,HLA-A,CDC34,ELOB,HSPB1 |
| ERK/MAPK Signaling | 0.458 | 0.0245 | 0.447 | SHC1,PPP2CB,PLA2G5,PPP1R14B,HSPB1 |
| Role of PKR in Interferon Induction and Antiviral Response | 0.458 | 0.0488 | #NUM! | NFKBIA,TNFRSF1A |
| Superpathway of Serine and Glycine Biosynthesis I | 0.458 | 0.143 | #NUM! | PSPH |
| Phosphatidylcholine Biosynthesis I | 0.458 | 0.143 | #NUM! | CHPT1 |
| Aspartate Degradation II | 0.458 | 0.143 | #NUM! | MDH2 |
| Thrombin Signaling | 0.438 | 0.0238 | 2 | SHC1,CAMK1,RHOC,MYL12A,EGFR |
| MIF Regulation of Innate Immunity | 0.438 | 0.0465 | #NUM! | NFKBIA,PLA2G5 |
| Superoxide Radicals Degradation | 0.42 | 0.125 | #NUM! | SOD2 |
| iNOS Signaling | 0.418 | 0.0444 | #NUM! | CALM1 (includes others),NFKBIA |
| Sumoylation Pathway | 0.418 | 0.0312 | #NUM! | NFKBIA,STUB1,RHOC |
| ATM Signaling | 0.403 | 0.0306 | #NUM! | PPP2CB,NFKBIA,GADD45A |
| nNOS Signaling in Neurons | 0.401 | 0.0426 | #NUM! | CALM1 (includes others),CAPN2 |
| PPAR Signaling | 0.387 | 0.0297 | #NUM! | SHC1,NFKBIA,TNFRSF1A |
| Gαq Signaling | 0.387 | 0.0248 | #NUM! | HRH1,CALM1 (includes others),NFKBIA,RHOC |
| Chondroitin Sulfate Biosynthesis (Late Stages) | 0.387 | 0.0408 | #NUM! | CHST2,CHPF |
| TNFR1 Signaling | 0.383 | 0.04 | #NUM! | NFKBIA,TNFRSF1A |
| Assembly of RNA Polymerase II Complex | 0.383 | 0.04 | #NUM! | TAF10,POLR2L |
| Role of Osteoblasts, Osteoclasts and Chondrocytes in Rheumatoid Arthritis | 0.365 | 0.0215 | #NUM! | SFRP4,CALM1 (includes others),NFKBIA,CSF1,TNFRSF1A |
| Glioblastoma Multiforme Signaling | 0.365 | 0.0238 | 1 | CDKN2A,SHC1,RHOC,EGFR |
| Cholecystokinin/Gastrin-mediated Signaling | 0.365 | 0.028 | #NUM! | SHC1,RHOC,EGFR |
| VEGF Signaling | 0.359 | 0.0275 | #NUM! | SHC1,SFN,ACTN1 |
| Role of Cytokines in Mediating Communication between Immune Cells | 0.359 | 0.037 | #NUM! | IL32,IL25 |
| eNOS Signaling | 0.359 | 0.0233 | #NUM! | CALM1 (includes others),STUB1,CAV1,AQP4 |
| Wnt/β-catenin Signaling | 0.359 | 0.0233 | #NUM! | CDKN2A,PPP2CB,SFRP4,CD44 |
| p53 Signaling | 0.359 | 0.027 | #NUM! | CDKN2A,GADD45A,SFN |
| Type I Diabetes Mellitus Signaling | 0.359 | 0.027 | #NUM! | NFKBIA,TNFRSF1A,HLA-A |
| Hematopoiesis from Multipotent Stem Cells | 0.352 | 0.0833 | #NUM! | CSF1 |
| Phospholipase C Signaling | 0.352 | 0.0205 | 2.236 | SHC1,CALM1 (includes others),RHOC,PLA2G5,MYL12A |
| RhoGDI Signaling | 0.352 | 0.0226 | -2 | RHOC,CD44,MSN,MYL12A |
| Chondroitin Sulfate Biosynthesis | 0.352 | 0.0351 | #NUM! | CHST2,CHPF |
| Virus Entry via Endocytic Pathways | 0.345 | 0.0259 | #NUM! | FLNA,HLA-A,CAV1 |
| Choline Biosynthesis III | 0.345 | 0.0769 | #NUM! | CHPT1 |
| Nur77 Signaling in T Lymphocytes | 0.345 | 0.0339 | #NUM! | CALM1 (includes others),HLA-A |
| Dermatan Sulfate Biosynthesis | 0.345 | 0.0339 | #NUM! | CHST2,CHPF |
| Pancreatic Adenocarcinoma Signaling | 0.343 | 0.025 | #NUM! | CDKN2A,CDKN2B,EGFR |
| G Beta Gamma Signaling | 0.343 | 0.025 | #NUM! | SHC1,CAV1,EGFR |
| p38 MAPK Signaling | 0.343 | 0.025 | #NUM! | TNFRSF1A,PLA2G5,HSPB1 |
| Induction of Apoptosis by HIV1 | 0.343 | 0.0328 | #NUM! | NFKBIA,TNFRSF1A |
| Phenylalanine Degradation IV (Mammalian, via Side Chain) | 0.343 | 0.0714 | #NUM! | SMOX |
| Agranulocyte Adhesion and Diapedesis | 0.305 | 0.0207 | #NUM! | HRH1,TNFRSF1A,CXCL14,MSN |
| B Cell Receptor Signaling | 0.305 | 0.0206 | 2 | SHC1,CALM1 (includes others),NFKBIA,BCL6 |
| Extrinsic Prothrombin Activation Pathway | 0.305 | 0.0625 | #NUM! | F3 |
| Parkinson's Signaling | 0.305 | 0.0625 | #NUM! | GPR37 |
| Cell Cycle: G1/S Checkpoint Regulation | 0.299 | 0.0299 | #NUM! | CDKN2A,CDKN2B |
| EGF Signaling | 0.293 | 0.0294 | #NUM! | SHC1,EGFR |
| Cellular Effects of Sildenafil (Viagra) | 0.293 | 0.0229 | #NUM! | CALM1 (includes others),GPR37,MYL12A |
| IL-10 Signaling | 0.293 | 0.029 | #NUM! | NFKBIA,BLVRB |
| CD28 Signaling in T Helper Cells | 0.293 | 0.0227 | #NUM! | CALM1 (includes others),NFKBIA,HLA-A |
| Estrogen Receptor Signaling | 0.287 | 0.0224 | #NUM! | SHC1,TAF10,POLR2L |
| GP6 Signaling Pathway | 0.287 | 0.0224 | #NUM! | COL1A2,CALM1 (includes others),COL8A1 |
| ERK5 Signaling | 0.279 | 0.0278 | #NUM! | SFN,EGFR |
| Androgen Signaling | 0.279 | 0.0219 | #NUM! | SHC1,CALM1 (includes others),POLR2L |
| GADD45 Signaling | 0.279 | 0.0526 | #NUM! | GADD45A |
| DNA damage-induced 14-3-3σ Signaling | 0.279 | 0.0526 | #NUM! | SFN |
| HMGB1 Signaling | 0.274 | 0.0216 | #NUM! | RHOC,TNFRSF1A,IL25 |
| Hypoxia Signaling in the Cardiovascular System | 0.272 | 0.0267 | #NUM! | NFKBIA,CDC34 |
| Leukocyte Extravasation Signaling | 0.272 | 0.019 | 1 | TIMP1,CD44,ACTN1,MSN |
| Aryl Hydrocarbon Receptor Signaling | 0.272 | 0.0213 | #NUM! | CDKN2A,MGST1,HSPB1 |
| Heparan Sulfate Biosynthesis (Late Stages) | 0.27 | 0.0263 | #NUM! | CHST2,PRDX6 |
| Chemokine Signaling | 0.265 | 0.026 | #NUM! | CALM1 (includes others),CAMK1 |
| Maturity Onset Diabetes of Young (MODY) Signaling | 0.265 | 0.0476 | #NUM! | GAPDH |
| Polyamine Regulation in Colon Cancer | 0.25 | 0.0455 | #NUM! | SAT1 |
| Xenobiotic Metabolism Signaling | 0.244 | 0.0169 | #NUM! | CHST2,PPP2CB,MGST1,CAMK1,SMOX |
| Hereditary Breast Cancer Signaling | 0.244 | 0.02 | #NUM! | GADD45A,SFN,POLR2L |
| Ovarian Cancer Signaling | 0.244 | 0.02 | #NUM! | CDKN2A,CD44,EGFR |
| Non-Small Cell Lung Cancer Signaling | 0.244 | 0.0241 | #NUM! | CDKN2A,EGFR |
| Heparan Sulfate Biosynthesis | 0.244 | 0.0241 | #NUM! | CHST2,PRDX6 |
| TCA Cycle II (Eukaryotic) | 0.244 | 0.0417 | #NUM! | MDH2 |
| EIF2 Signaling | 0.244 | 0.0176 | #NUM! | SHC1,RPL35,RPS18,RPL36 |
| Type II Diabetes Mellitus Signaling | 0.244 | 0.0195 | #NUM! | NFKBIA,TNFRSF1A,PKM |
| Small Cell Lung Cancer Signaling | 0.244 | 0.0235 | #NUM! | NFKBIA,CDKN2B |
| IL-17A Signaling in Gastric Cells | 0.244 | 0.04 | #NUM! | EGFR |
| Role of JAK family kinases in IL-6-type Cytokine Signaling | 0.244 | 0.04 | #NUM! | OSMR |
| Tryptophan Degradation X (Mammalian, via Tryptamine) | 0.244 | 0.04 | #NUM! | SMOX |
| Regulation of IL-2 Expression in Activated and Anergic T Lymphocytes | 0.244 | 0.0233 | #NUM! | CALM1 (includes others),NFKBIA |
| Erythropoietin Signaling | 0.239 | 0.023 | #NUM! | SHC1,NFKBIA |
| Antiproliferative Role of TOB in T Cell Signaling | 0.235 | 0.0385 | #NUM! | CDC34 |
| Hepatic Cholestasis | 0.233 | 0.0188 | #NUM! | NFKBIA,TNFRSF1A,IL25 |
| Regulation of Actin-based Motility by Rho | 0.233 | 0.0222 | #NUM! | RHOC,MYL12A |
| Altered T Cell and B Cell Signaling in Rheumatoid Arthritis | 0.233 | 0.0222 | #NUM! | CSF1,HLA-A |
| OX40 Signaling Pathway | 0.233 | 0.022 | #NUM! | NFKBIA,HLA-A |
| IL-7 Signaling Pathway | 0.233 | 0.022 | #NUM! | SHC1,BCL6 |
| Regulation of eIF4 and p70S6K Signaling | 0.233 | 0.0184 | #NUM! | SHC1,PPP2CB,RPS18 |
| Dopamine-DARPP32 Feedback in cAMP Signaling | 0.233 | 0.0183 | #NUM! | PPP2CB,CALM1 (includes others),PPP1R14B |
| Cardiac Hypertrophy Signaling | 0.233 | 0.0166 | 2 | CALM1 (includes others),RHOC,MYL12A,HSPB1 |
| PEDF Signaling | 0.233 | 0.0215 | #NUM! | NFKBIA,SOD2 |
| Protein Kinase A Signaling | 0.233 | 0.015 | 0 | CALM1 (includes others),NFKBIA,FLNA,SFN,PPP1R14B,MYL12A |
| Neuregulin Signaling | 0.233 | 0.0213 | #NUM! | SHC1,EGFR |
| Bladder Cancer Signaling | 0.233 | 0.0213 | #NUM! | CDKN2A,EGFR |
| VEGF Family Ligand-Receptor Interactions | 0.233 | 0.0213 | #NUM! | SHC1,PLA2G5 |
| IL-4 Signaling | 0.229 | 0.0211 | #NUM! | SHC1,HLA-A |
| PDGF Signaling | 0.228 | 0.0208 | #NUM! | SHC1,CAV1 |
| TNFR2 Signaling | 0.228 | 0.0333 | #NUM! | NFKBIA |
| Glutathione-mediated Detoxification | 0.223 | 0.0323 | #NUM! | MGST1 |
| Ceramide Signaling | 0.223 | 0.0202 | #NUM! | PPP2CB,TNFRSF1A |
| CTLA4 Signaling in Cytotoxic T Lymphocytes | 0.223 | 0.0202 | #NUM! | PPP2CB,HLA-A |
| Signaling by Rho Family GTPases | 0.223 | 0.0159 | 2 | RHOC,VIM,MSN,MYL12A |
| Cytotoxic T Lymphocyte-mediated Apoptosis of Target Cells | 0.223 | 0.0312 | #NUM! | HLA-A |
| 4-1BB Signaling in T Lymphocytes | 0.223 | 0.0312 | #NUM! | NFKBIA |
| Fatty Acid β-oxidation I | 0.223 | 0.0312 | #NUM! | ECI2 |
| RANK Signaling in Osteoclasts | 0.214 | 0.0196 | #NUM! | CALM1 (includes others),NFKBIA |
| Inhibition of Angiogenesis by TSP1 | 0.212 | 0.0294 | #NUM! | SDC2 |
| Role of JAK2 in Hormone-like Cytokine Signaling | 0.212 | 0.0294 | #NUM! | SHC1 |
| ErbB Signaling | 0.212 | 0.0192 | #NUM! | SHC1,EGFR |
| CDK5 Signaling | 0.212 | 0.019 | #NUM! | PPP2CB,PPP1R14B |
| FAK Signaling | 0.212 | 0.019 | #NUM! | CAPN2,EGFR |
| Coagulation System | 0.212 | 0.0286 | #NUM! | F3 |
| Nucleotide Excision Repair Pathway | 0.212 | 0.0286 | #NUM! | POLR2L |
| Glucocorticoid Receptor Signaling | 0.212 | 0.0145 | #NUM! | SHC1,NFKBIA,ANXA1,TAF10,POLR2L |
| PAK Signaling | 0.209 | 0.0187 | #NUM! | SHC1,MYL12A |
| B Cell Development | 0.209 | 0.0278 | #NUM! | HLA-A |
| Antioxidant Action of Vitamin C | 0.209 | 0.0185 | #NUM! | NFKBIA,PLA2G5 |
| NF-κB Signaling | 0.209 | 0.016 | #NUM! | NFKBIA,TNFRSF1A,EGFR |
| Cell Cycle Regulation by BTG Family Proteins | 0.209 | 0.027 | #NUM! | PPP2CB |
| Dopamine Degradation | 0.209 | 0.027 | #NUM! | SMOX |
| SAPK/JNK Signaling | 0.208 | 0.0182 | #NUM! | SHC1,GADD45A |
| Antigen Presentation Pathway | 0.206 | 0.0263 | #NUM! | HLA-A |
| Notch Signaling | 0.206 | 0.0263 | #NUM! | HEY1 |
| Role of NFAT in Regulation of the Immune Response | 0.204 | 0.0156 | #NUM! | CALM1 (includes others),NFKBIA,HLA-A |
| Nitric Oxide Signaling in the Cardiovascular System | 0.204 | 0.0177 | #NUM! | CALM1 (includes others),CAV1 |
| April Mediated Signaling | 0.204 | 0.0256 | #NUM! | NFKBIA |
| Noradrenaline and Adrenaline Degradation | 0.201 | 0.025 | #NUM! | SMOX |
| Neuropathic Pain Signaling In Dorsal Horn Neurons | 0.201 | 0.0174 | #NUM! | CAMK1,GPR37 |
| T Cell Receptor Signaling | 0.201 | 0.0174 | #NUM! | CALM1 (includes others),NFKBIA |
| B Cell Activating Factor Signaling | 0.198 | 0.0244 | #NUM! | NFKBIA |
| nNOS Signaling in Skeletal Muscle Cells | 0.198 | 0.0244 | #NUM! | CALM1 (includes others) |
| Intrinsic Prothrombin Activation Pathway | 0.195 | 0.0238 | #NUM! | COL1A2 |
| Adrenomedullin signaling pathway | 0.195 | 0.015 | #NUM! | SHC1,CALM1 (includes others),GPR37 |
| Paxillin Signaling | 0.195 | 0.0168 | #NUM! | ITGA7,ACTN1 |
| Axonal Guidance Signaling | 0.195 | 0.0131 | #NUM! | SHC1,TUBB6,SDC2,TUBB2A,TUBA4A,MYL12A |
| Role of p14/p19ARF in Tumor Suppression | 0.195 | 0.0233 | #NUM! | CDKN2A |
| Serotonin Receptor Signaling | 0.195 | 0.0233 | #NUM! | SMOX |
| LXR/RXR Activation | 0.194 | 0.0165 | #NUM! | TNFRSF1A,CLU |
| Role of RIG1-like Receptors in Antiviral Innate Immunity | 0.192 | 0.0227 | #NUM! | NFKBIA |
| Gαi Signaling | 0.192 | 0.0163 | #NUM! | SHC1,CAV1 |
| Pyrimidine Ribonucleotides Interconversion | 0.192 | 0.0222 | #NUM! | ANXA1 |
| mTOR Signaling | 0.192 | 0.0145 | #NUM! | PPP2CB,RHOC,RPS18 |
| RhoA Signaling | 0.192 | 0.0161 | #NUM! | MSN,MYL12A |
| PTEN Signaling | 0.192 | 0.016 | #NUM! | SHC1,EGFR |
| Thyroid Cancer Signaling | 0.192 | 0.0217 | #NUM! | SHC1 |
| Dermatan Sulfate Biosynthesis (Late Stages) | 0.192 | 0.0217 | #NUM! | CHST2 |
| Synaptic Long Term Potentiation | 0.192 | 0.0159 | #NUM! | CALM1 (includes others),PPP1R14B |
| Sperm Motility | 0.192 | 0.0157 | #NUM! | CALM1 (includes others),PLA2G5 |
| Pyrimidine Ribonucleotides De Novo Biosynthesis | 0.192 | 0.0213 | #NUM! | ANXA1 |
| Autoimmune Thyroid Disease Signaling | 0.192 | 0.0208 | #NUM! | HLA-A |
| Graft-versus-Host Disease Signaling | 0.192 | 0.0208 | #NUM! | HLA-A |
| Hematopoiesis from Pluripotent Stem Cells | 0.192 | 0.0208 | #NUM! | CSF1 |
| fMLP Signaling in Neutrophils | 0.192 | 0.0155 | #NUM! | CALM1 (includes others),NFKBIA |
| UVC-Induced MAPK Signaling | 0.187 | 0.0204 | #NUM! | EGFR |
| CREB Signaling in Neurons | 0.183 | 0.0138 | #NUM! | SHC1,CALM1 (includes others),POLR2L |
| Molecular Mechanisms of Cancer | 0.182 | 0.0127 | #NUM! | CDKN2A,SHC1,NFKBIA,RHOC,CDKN2B |
| Amyloid Processing | 0.182 | 0.0196 | #NUM! | CAPN2 |
| Adipogenesis pathway | 0.182 | 0.0149 | #NUM! | TNFRSF1A,CEBPD |
| CCR3 Signaling in Eosinophils | 0.182 | 0.0147 | #NUM! | CALM1 (includes others),PLA2G5 |
| PI3K Signaling in B Lymphocytes | 0.182 | 0.0147 | #NUM! | CALM1 (includes others),NFKBIA |
| Neuroinflammation Signaling Pathway | 0.182 | 0.0129 | 1 | SOD2,TNFRSF1A,HLA-A,PLA2G5 |
| Iron homeostasis signaling pathway | 0.182 | 0.0146 | #NUM! | EPAS1,EGFR |
| CD27 Signaling in Lymphocytes | 0.182 | 0.0189 | #NUM! | NFKBIA |
| Semaphorin Signaling in Neurons | 0.182 | 0.0189 | #NUM! | RHOC |
| Role of NFAT in Cardiac Hypertrophy | 0.182 | 0.0133 | #NUM! | SHC1,CALM1 (includes others),CAMK1 |
| Triacylglycerol Degradation | 0.179 | 0.0185 | #NUM! | PRDX6 |
| Gα12/13 Signaling | 0.176 | 0.0142 | #NUM! | NFKBIA,MYL12A |
| Cardiac β-adrenergic Signaling | 0.176 | 0.0142 | #NUM! | PPP2CB,PPP1R14B |
| Unfolded protein response | 0.174 | 0.0179 | #NUM! | CEBPD |
| Role of CHK Proteins in Cell Cycle Checkpoint Control | 0.172 | 0.0175 | #NUM! | PPP2CB |
| Glutamate Receptor Signaling | 0.172 | 0.0175 | #NUM! | CALM1 (includes others) |
| Insulin Receptor Signaling | 0.163 | 0.0136 | #NUM! | SHC1,PPP1R14B |
| Th2 Pathway | 0.159 | 0.0133 | #NUM! | HLA-A,IL25 |
| Melanoma Signaling | 0.159 | 0.0164 | #NUM! | CDKN2A |
| PCP pathway | 0.159 | 0.0164 | #NUM! | JUNB |
| Actin Nucleation by ARP-WASP Complex | 0.159 | 0.0161 | #NUM! | RHOC |
| Phospholipases | 0.159 | 0.0161 | #NUM! | PLA2G5 |
| autophagy | 0.159 | 0.0161 | #NUM! | SQSTM1 |
| Activation of IRF by Cytosolic Pattern Recognition Receptors | 0.156 | 0.0159 | #NUM! | NFKBIA |
| Opioid Signaling Pathway | 0.154 | 0.0121 | #NUM! | CALM1 (includes others),NFKBIA,CAMK1 |
| Mitotic Roles of Polo-Like Kinase | 0.147 | 0.0152 | #NUM! | PPP2CB |
| UVB-Induced MAPK Signaling | 0.147 | 0.0152 | #NUM! | EGFR |
| Lymphotoxin β Receptor Signaling | 0.147 | 0.0149 | #NUM! | NFKBIA |
| Eicosanoid Signaling | 0.147 | 0.0149 | #NUM! | PLA2G5 |
| Colorectal Cancer Metastasis Signaling | 0.147 | 0.0118 | #NUM! | RHOC,TNFRSF1A,EGFR |
| Role of IL-17A in Arthritis | 0.144 | 0.0145 | #NUM! | NFKBIA |
| Superpathway of Melatonin Degradation | 0.144 | 0.0143 | #NUM! | SMOX |
| IL-2 Signaling | 0.144 | 0.0143 | #NUM! | SHC1 |
| PKCθ Signaling in T Lymphocytes | 0.144 | 0.0121 | #NUM! | NFKBIA,HLA-A |
| Thrombopoietin Signaling | 0.144 | 0.0141 | #NUM! | SHC1 |
| Tight Junction Signaling | 0.144 | 0.012 | #NUM! | PPP2CB,TNFRSF1A |
| Cdc42 Signaling | 0.144 | 0.012 | #NUM! | HLA-A,MYL12A |
| Melatonin Signaling | 0.144 | 0.0139 | #NUM! | CALM1 (includes others) |
| MSP-RON Signaling Pathway | 0.144 | 0.0139 | #NUM! | CSF1 |
| Aldosterone Signaling in Epithelial Cells | 0.144 | 0.0119 | #NUM! | CRYAB,HSPB1 |
| CXCR4 Signaling | 0.143 | 0.0117 | #NUM! | RHOC,MYL12A |
| GNRH Signaling | 0.143 | 0.0117 | #NUM! | CALM1 (includes others),EGFR |
| Agrin Interactions at Neuromuscular Junction | 0.143 | 0.0133 | #NUM! | EGFR |
| ErbB2-ErbB3 Signaling | 0.143 | 0.0133 | #NUM! | SHC1 |
| Toll-like Receptor Signaling | 0.143 | 0.0132 | #NUM! | NFKBIA |
| Role of JAK1 and JAK3 in γc Cytokine Signaling | 0.143 | 0.013 | #NUM! | SHC1 |
| Serotonin Degradation | 0.143 | 0.013 | #NUM! | SMOX |
| VDR/RXR Activation | 0.143 | 0.0128 | #NUM! | GADD45A |
| Role of MAPK Signaling in the Pathogenesis of Influenza | 0.143 | 0.0128 | #NUM! | PLA2G5 |
| IL-17A Signaling in Airway Cells | 0.143 | 0.0128 | #NUM! | NFKBIA |
| ErbB4 Signaling | 0.143 | 0.0128 | #NUM! | SHC1 |
| CD40 Signaling | 0.143 | 0.0127 | #NUM! | NFKBIA |
| Role of PI3K/AKT Signaling in the Pathogenesis of Influenza | 0.143 | 0.0127 | #NUM! | NFKBIA |
| GM-CSF Signaling | 0.143 | 0.0127 | #NUM! | SHC1 |
| Ephrin Receptor Signaling | 0.143 | 0.0112 | #NUM! | SHC1,SDC2 |
| Role of BRCA1 in DNA Damage Response | 0.143 | 0.0125 | #NUM! | GADD45A |
| Synaptic Long Term Depression | 0.143 | 0.0111 | #NUM! | PPP2CB,PLA2G5 |
| IL-15 Signaling | 0.139 | 0.0122 | #NUM! | SHC1 |
| GDNF Family Ligand-Receptor Interactions | 0.139 | 0.0122 | #NUM! | SHC1 |
| Neurotrophin/TRK Signaling | 0.138 | 0.012 | #NUM! | SHC1 |
| Th1 and Th2 Activation Pathway | 0.138 | 0.0108 | #NUM! | HLA-A,IL25 |
| Angiopoietin Signaling | 0.137 | 0.0119 | #NUM! | NFKBIA |
| FcγRIIB Signaling in B Lymphocytes | 0.137 | 0.0118 | #NUM! | SHC1 |
| Allograft Rejection Signaling | 0.137 | 0.0118 | #NUM! | HLA-A |
| Estrogen-Dependent Breast Cancer Signaling | 0.136 | 0.0116 | #NUM! | EGFR |
| Macropinocytosis Signaling | 0.136 | 0.0115 | #NUM! | CSF1 |
| Renal Cell Carcinoma Signaling | 0.136 | 0.0114 | #NUM! | ELOB |
| IL-3 Signaling | 0.136 | 0.0112 | #NUM! | SHC1 |
| Prolactin Signaling | 0.136 | 0.0112 | #NUM! | SHC1 |
| Crosstalk between Dendritic Cells and Natural Killer Cells | 0.136 | 0.0112 | #NUM! | HLA-A |
| JAK/Stat Signaling | 0.136 | 0.0112 | #NUM! | SHC1 |
| IL-17 Signaling | 0.132 | 0.011 | #NUM! | TIMP1 |
| IL-1 Signaling | 0.131 | 0.0109 | #NUM! | NFKBIA |
| Natural Killer Cell Signaling | 0 | 0.00781 | #NUM! | SHC1 |
| Amyotrophic Lateral Sclerosis Signaling | 0 | 0.00901 | #NUM! | CAPN2 |
| Fc Epsilon RI Signaling | 0 | 0.008 | #NUM! | PLA2G5 |
| PPARα/RXRα Activation | 0 | 0.0108 | #NUM! | SHC1,NFKBIA |
| FXR/RXR Activation | 0 | 0.00794 | #NUM! | CLU |
| α-Adrenergic Signaling | 0 | 0.0108 | #NUM! | CALM1 (includes others) |
| Clathrin-mediated Endocytosis Signaling | 0 | 0.00483 | #NUM! | CLU |
| IL-8 Signaling | 0 | 0.00985 | #NUM! | RHOC,EGFR |
| IL-12 Signaling and Production in Macrophages | 0 | 0.00685 | #NUM! | CLU |
| Role of Pattern Recognition Receptors in Recognition of Bacteria and Viruses | 0 | 0.0073 | #NUM! | IL25 |
| LPS-stimulated MAPK Signaling | 0 | 0.0108 | #NUM! | NFKBIA |
| NF-κB Activation by Viruses | 0 | 0.0108 | #NUM! | NFKBIA |
| CCR5 Signaling in Macrophages | 0 | 0.0105 | #NUM! | CALM1 (includes others) |
| HIF1α Signaling | 0 | 0.00806 | #NUM! | ELOB |
| Endothelin-1 Signaling | 0 | 0.0102 | #NUM! | SHC1,PLA2G5 |
| Relaxin Signaling | 0 | 0.00633 | #NUM! | NFKBIA |
| Renin-Angiotensin Signaling | 0 | 0.00781 | #NUM! | SHC1 |
| Corticotropin Releasing Hormone Signaling | 0 | 0.00719 | #NUM! | CALM1 (includes others) |
| HGF Signaling | 0 | 0.0084 | #NUM! | CDKN2A |
| FLT3 Signaling in Hematopoietic Progenitor Cells | 0 | 0.0108 | #NUM! | SHC1 |
| Melanocyte Development and Pigmentation Signaling | 0 | 0.00962 | #NUM! | SHC1 |
| Role of NANOG in Mammalian Embryonic Stem Cell Pluripotency | 0 | 0.00781 | #NUM! | SHC1 |
| Prostate Cancer Signaling | 0 | 0.00971 | #NUM! | NFKBIA |
| Chronic Myeloid Leukemia Signaling | 0 | 0.00893 | #NUM! | CDKN2A |
| Communication between Innate and Adaptive Immune Cells | 0 | 0.0105 | #NUM! | HLA-A |
| Sphingosine-1-phosphate Signaling | 0 | 0.008 | #NUM! | RHOC |
| Systemic Lupus Erythematosus Signaling | 0 | 0.00429 | #NUM! | HLA-A |
| AMPK Signaling | 0 | 0.00463 | #NUM! | PPP2CB |
| Rac Signaling | 0 | 0.00813 | #NUM! | CD44 |
| HER-2 Signaling in Breast Cancer | 0 | 0.0106 | #NUM! | EGFR |
| NGF Signaling | 0 | 0.008 | #NUM! | SHC1 |
| Mouse Embryonic Stem Cell Pluripotency | 0 | 0.00893 | #NUM! | ID3 |
| D-myo-inositol-5-phosphate Metabolism | 0 | 0.00617 | #NUM! | PPP1R14B |
| D-myo-inositol (1,4,5,6)-Tetrakisphosphate Biosynthesis | 0 | 0.00694 | #NUM! | PPP1R14B |
| Superpathway of Inositol Phosphate Compounds | 0 | 0.00847 | #NUM! | PPP1R14B,EGFR |
| D-myo-inositol (3,4,5,6)-tetrakisphosphate Biosynthesis | 0 | 0.00694 | #NUM! | PPP1R14B |
| 3-phosphoinositide Degradation | 0 | 0.00633 | #NUM! | PPP1R14B |
| 3-phosphoinositide Biosynthesis | 0 | 0.00995 | #NUM! | PPP1R14B,EGFR |
| Salvage Pathways of Pyrimidine Ribonucleotides | 0 | 0.0103 | #NUM! | UPP1 |
| Regulation of the Epithelial-Mesenchymal Transition Pathway | 0 | 0.00513 | #NUM! | EGFR |
| Tec Kinase Signaling | 0 | 0.00588 | #NUM! | RHOC |
| UVA-Induced MAPK Signaling | 0 | 0.00893 | #NUM! | EGFR |
| STAT3 Pathway | 0 | 0.00971 | #NUM! | EGFR |
| GABA Receptor Signaling | 0 | 0.0105 | #NUM! | GPR37 |
| cAMP-mediated signaling | 0 | 0.00877 | #NUM! | CALM1 (includes others),CAMK1 |
| G-Protein Coupled Receptor Signaling | 0 | 0.0106 | #NUM! | SHC1,HRH1,NFKBIA |
| Phagosome Formation | 0 | 0.00763 | #NUM! | RHOC |
| Th1 Pathway | 0 | 0.00741 | #NUM! | HLA-A |
| Calcium Signaling | 0 | 0.00971 | #NUM! | CALM1 (includes others),CAMK1 |

| Cluster 7 |  |  |  |  |
| --- | --- | --- | --- | --- |
| © 2000-2018 QIAGEN. All rights reserved. |  |  |  |  |
| Ingenuity Canonical Pathways | -log(B-H p-value) | Ratio | z-score | Molecules |
| EIF2 Signaling | 8 | 0.11 | 3.873 | RPL11,RPL22,RPLP1,RPL36A,RPL26,RPL7A,CCND1,RPS4X,RPL23A,PAIP1,EIF3A,RPS24,RPL4,RPS8,RPL23,EIF2S3,RPL28,RPL9,RPL5,RPS15A,RPL6,RPS25,EIF3L,RPSA,RPLP0 |
| Protein Kinase A Signaling | 2.86 | 0.0599 | 1.342 | MYH10,TCF4,YWHAE,ADD2,GNG2,MAP3K1,RACK1,YWHAZ,GNAI1,PLCG1,AKAP7,PTEN,PTK2,YWHAQ,GNB4,CALM1 (includes others),H3F3A/H3F3B,PTPRS,DCC,PTPRZ1,H1F0,CDC26,PPP3CA,PRKAR1A |
| Axonal Guidance Signaling | 1.69 | 0.0503 | #NUM! | TUBB3,RGS3,SLIT1,ITSN1,SEMA5A,ARHGEF7,CRKL,GNG2,RACK1,GNAI1,PLCG1,DPYSL5,TUBB,ROBO1,PTK2,GNB4,ACTR3,NTRK3,DCC,PFN2,TUBB4A,PPP3CA,PRKAR1A |
| Regulation of eIF4 and p70S6K Signaling | 1.68 | 0.0736 | #NUM! | PPP2R1A,EIF4EBP2,RPS8,PAIP1,EIF3A,RPS25,RPS15A,EIF2S3,RPS4X,EIF3L,RPSA,RPS24 |
| Signaling by Rho Family GTPases | 1.59 | 0.0595 | 2.714 | ARHGEF7,GNG2,RACK1,SEPT7,GNAI1,WASF1,PTK2,STMN1,GNB4,PIP5K1A,CDH2,JUN,ACTR3,CDH13,SEPT2 |
| Epithelial Adherens Junction Signaling | 1.59 | 0.0733 | #NUM! | MYH10,YES1,TCF4,TUBB3,CDH2,ACTR3,WASF1,TUBB4A,MAGI2,TUBB,PTEN |
| Amyloid Processing | 1.49 | 0.118 | #NUM! | CAPN5,CSNK2A1,MAPT,CSNK1A1,PRKAR1A,APP |
| 14-3-3-mediated Signaling | 1.49 | 0.073 | 1.134 | YWHAQ,TSC1,TUBB3,JUN,YWHAE,MAPT,YWHAZ,PLCG1,TUBB4A,TUBB |
| Androgen Signaling | 1.49 | 0.073 | 0.816 | CALM1 (includes others),GNB4,CACNB1,KAT7,JUN,GNG2,RACK1,GNAI1,CCND1,PRKAR1A |
| Ephrin B Signaling | 1.46 | 0.0959 | 1 | PTK2,GNB4,RGS3,ITSN1,GNG2,RACK1,GNAI1 |
| CCR5 Signaling in Macrophages | 1.46 | 0.0842 | 2 | CALM1 (includes others),GNB4,CACNB1,JUN,GNG2,RACK1,GNAI1,PLCG1 |
| Glutamate Receptor Signaling | 1.39 | 0.105 | #NUM! | CALM1 (includes others),GNG2,GRIA2,SLC38A1,GRIA4,GRIA3 |
| Gap Junction Signaling | 1.39 | 0.0597 | #NUM! | TUBB3,NOV,GRIA2,CSNK1A1,GNAI1,PLCG1,TUBB4A,TUBB,GRIA4,PPP3CA,PRKAR1A,GRIA3 |
| PTEN Signaling | 1.39 | 0.072 | -1.667 | PTK2,CSNK2A1,CASP3,NTRK3,BMPR1A,PDGFRA,MAGI2,CCND1,PTEN |
| RhoGDI Signaling | 1.39 | 0.0621 | -2.646 | GNB4,PIP5K1A,CDH2,ACTR3,ARHGEF7,GNG2,RACK1,GNAI1,ARHGAP12,WASF1,CDH13 |
| Breast Cancer Regulation by Stathmin1 | 1.3 | 0.0569 | #NUM! | STMN1,CALM1 (includes others),GNB4,PPP2R1A,TUBB3,ARHGEF7,GNG2,RACK1,GNAI1,TUBB4A,TUBB,PRKAR1A |
| Amyotrophic Lateral Sclerosis Signaling | 1.22 | 0.0721 | 1.134 | CAPN5,CASP3,NEFL,GRIA2,SOD1,GRIA4,PPP3CA,GRIA3 |
| IGF-1 Signaling | 1.22 | 0.0714 | 2 | PTK2,YWHAQ,CSNK2A1,NOV,JUN,YWHAE,YWHAZ,PRKAR1A |
| Role of NFAT in Cardiac Hypertrophy | 1.16 | 0.0533 | 2.121 | CALM1 (includes others),GNB4,CACNB1,HDAC2,GNG2,MAP3K1,RACK1,CSNK1A1,GNAI1,PLCG1,PPP3CA,PRKAR1A |
| IL-1 Signaling | 1.16 | 0.0761 | 2 | GNB4,JUN,GNG2,MAP3K1,RACK1,GNAI1,PRKAR1A |
| α-Adrenergic Signaling | 1.16 | 0.0753 | #NUM! | CALM1 (includes others),GNB4,GNG2,RACK1,GNAI1,PLCG1,PRKAR1A |
| Wnt/β-catenin Signaling | 1.15 | 0.0581 | 0.333 | SOX4,CSNK2A1,PPP2R1A,TCF4,CDH2,JUN,CSNK1A1,SOX9,SOX11,CCND1 |
| G Protein Signaling Mediated by Tubby | 1.13 | 0.125 | #NUM! | GNB4,GNG2,RACK1,PLCG1 |
| Actin Cytoskeleton Signaling | 1.13 | 0.0515 | 2.53 | PTK2,ABI2,MYH10,PIP5K1A,ACTR3,CYFIP2,CRKL,ARHGEF7,FGF14,PFN2,TRIO,WASF1 |
| Rac Signaling | 1.13 | 0.065 | 2.828 | PTK2,ABI2,PIP5K1A,ACTR3,CYFIP2,JUN,MAP3K1,WASF1 |
| ATM Signaling | 1.13 | 0.0714 | 0.447 | SMC3,PPP2R1A,CBX1,JUN,TLK1,ZEB1,CBX3 |
| RhoA Signaling | 1.13 | 0.0645 | 2.121 | PTK2,PIP5K1A,ACTR3,SEPT7,PFN2,ARHGAP12,WASF1,SEPT2 |
| CREB Signaling in Neurons | 0.982 | 0.0505 | 2.646 | CALM1 (includes others),GNB4,CACNB1,GNG2,RACK1,GRIA2,GNAI1,PLCG1,GRIA4,PRKAR1A,GRIA3 |
| Huntington's Disease Signaling | 0.982 | 0.048 | -0.447 | ATP5PF,CAPN5,GNB4,JUN,CASP3,HDAC2,CASP2,GNG2,RACK1,DCTN1,GOSR1,HIP1 |
| GP6 Signaling Pathway | 0.982 | 0.0597 | 2.828 | PTK2,CALM1 (includes others),COL6A1,COL6A2,SCHIP1,COL9A3,COL20A1,KLF12 |
| Notch Signaling | 0.982 | 0.105 | #NUM! | CNTN1,MAML2,DLL3,JAG1 |
| p70S6K Signaling | 0.934 | 0.058 | 1.342 | YWHAQ,PPP2R1A,YWHAE,EEF2,MAPT,YWHAZ,GNAI1,PLCG1 |
| Tight Junction Signaling | 0.934 | 0.0539 | #NUM! | MYH10,PPP2R1A,JUN,JAM3,CPSF3,GOSR1,MAGI2,PRKAR1A,PTEN |
| Netrin Signaling | 0.852 | 0.0769 | #NUM! | CACNB1,DCC,PPP3CA,PRKAR1A,ENAH |
| Calcium Signaling | 0.852 | 0.0485 | 2.646 | MYH10,CALM1 (includes others),CACNB1,LETM1,HDAC2,GRIA2,GRIA4,PPP3CA,PRKAR1A,GRIA3 |
| Reelin Signaling in Neurons | 0.852 | 0.0652 | #NUM! | PAFAH1B2,YES1,MAPT,CRKL,DCX,APP |
| mTOR Signaling | 0.852 | 0.0483 | #NUM! | TSC1,PPP2R1A,RPS8,EIF3A,RPS25,RPS15A,RPS4X,EIF3L,RPSA,RPS24 |
| Cell Cycle: G1/S Checkpoint Regulation | 0.852 | 0.0746 | 0 | RPL11,CCND2,HDAC2,RPL5,CCND1 |
| G Beta Gamma Signaling | 0.852 | 0.0583 | 2.236 | GNB4,CACNB1,GNG2,RACK1,GNAI1,PLCG1,PRKAR1A |
| Fcγ Receptor-mediated Phagocytosis in Macrophages and Monocytes | 0.852 | 0.0645 | 2.449 | YES1,PIP5K1A,ACTR3,PLCG1,RAB11A,PTEN |
| Ephrin Receptor Signaling | 0.847 | 0.0503 | 2.333 | PTK2,GNB4,RGS3,ACTR3,ITSN1,CRKL,GNG2,RACK1,GNAI1 |
| PDGF Signaling | 0.811 | 0.0625 | 2.449 | CSNK2A1,JUN,CRKL,MAP3K1,PDGFRA,PLCG1 |
| Sertoli Cell-Sertoli Cell Junction Signaling | 0.798 | 0.0489 | #NUM! | TUBB3,JUN,JAM3,MAP3K1,TUBB4A,MAGI2,TUBB,PRKAR1A,PTEN |
| Synaptic Long Term Potentiation | 0.788 | 0.0556 | 2.646 | CALM1 (includes others),GRIA2,PLCG1,GRIA4,PPP3CA,PRKAR1A,GRIA3 |
| Lanosterol Biosynthesis | 0.765 | 1 | #NUM! | LSS |
| fMLP Signaling in Neutrophils | 0.765 | 0.0543 | 2.236 | CALM1 (includes others),GNB4,ACTR3,GNG2,RACK1,GNAI1,PPP3CA |
| Purine Nucleotides De Novo Biosynthesis II | 0.765 | 0.182 | #NUM! | PAICS,GART |
| TNFR1 Signaling | 0.765 | 0.08 | 2 | JUN,CASP3,CASP2,MAP3K1 |
| PI3K/AKT Signaling | 0.757 | 0.0534 | 0.378 | YWHAQ,TSC1,PPP2R1A,YWHAE,YWHAZ,CCND1,PTEN |
| Role of NFAT in Regulation of the Immune Response | 0.757 | 0.0469 | 2.449 | CALM1 (includes others),GNB4,JUN,GNG2,RACK1,CSNK1A1,GNAI1,PLCG1,PPP3CA |
| Chemokine Signaling | 0.739 | 0.0649 | 2.236 | PTK2,CALM1 (includes others),JUN,GNAI1,PLCG1 |
| Cleavage and Polyadenylation of Pre-mRNA | 0.728 | 0.167 | #NUM! | CPSF3,WDR33 |
| Tec Kinase Signaling | 0.666 | 0.0471 | 2 | PTK2,GNB4,YES1,TNFRSF10B,GNG2,RACK1,GNAI1,PLCG1 |
| P2Y Purigenic Receptor Signaling Pathway | 0.666 | 0.05 | 1.342 | GNB4,JUN,GNG2,RACK1,GNAI1,PLCG1,PRKAR1A |
| GNRH Signaling | 0.666 | 0.0468 | 2.449 | PTK2,CALM1 (includes others),CACNB1,JUN,GNG2,MAP3K1,GNAI1,PRKAR1A |
| p53 Signaling | 0.663 | 0.0541 | #NUM! | CCND2,JUN,TNFRSF10B,ST13,CCND1,PTEN |
| Clathrin-mediated Endocytosis Signaling | 0.643 | 0.0435 | #NUM! | CSNK2A1,ACTR3,AP3M1,AP2B1,FGF14,RAB11A,HIP1,CTTN,PPP3CA |
| DNA Methylation and Transcriptional Repression Signaling | 0.643 | 0.0882 | #NUM! | H3F3A/H3F3B,HDAC2,RBBP4 |
| Cardiac Hypertrophy Signaling | 0.643 | 0.0415 | 2.121 | CALM1 (includes others),GNB4,JUN,GNG2,MAP3K1,RACK1,GNAI1,PLCG1,PPP3CA,PRKAR1A |
| Nur77 Signaling in T Lymphocytes | 0.643 | 0.0678 | #NUM! | CALM1 (includes others),CASP3,HDAC2,PPP3CA |
| Regulation of IL-2 Expression in Activated and Anergic T Lymphocytes | 0.643 | 0.0581 | #NUM! | CALM1 (includes others),JUN,MAP3K1,PLCG1,PPP3CA |
| Neuropathic Pain Signaling In Dorsal Horn Neurons | 0.643 | 0.0522 | 2.449 | KCNQ2,GRIA2,PLCG1,GRIA4,PRKAR1A,GRIA3 |
| HIPPO signaling | 0.64 | 0.0575 | -1.342 | YWHAQ,PPP2R1A,YWHAE,YWHAZ,NF2 |
| Leukocyte Extravasation Signaling | 0.64 | 0.0427 | 1.89 | PTK2,JAM3,MMP16,CRKL,GNAI1,ARHGAP12,THY1,PLCG1,CTTN |
| Synaptic Long Term Depression | 0.627 | 0.0444 | 2.121 | PAFAH1B2,PPP2R1A,CACNB1,GRIA2,GNAI1,PLCG1,GRIA4,GRIA3 |
| L-DOPA Degradation | 0.626 | 0.5 | #NUM! | LRTOMT |
| HGF Signaling | 0.616 | 0.0504 | 2.449 | PTK2,JUN,CRKL,MAP3K1,PLCG1,CCND1 |
| Granzyme B Signaling | 0.616 | 0.125 | #NUM! | CASP3,LMNB1 |
| Parkinson's Signaling | 0.616 | 0.125 | #NUM! | UCHL1,CASP3 |
| Integrin Signaling | 0.594 | 0.0411 | 2.333 | PTK2,CAPN5,ACTR3,CRKL,ARHGEF7,PFN2,PLCG1,CTTN,PTEN |
| Colorectal Cancer Metastasis Signaling | 0.592 | 0.0394 | 1.414 | GNB4,TCF4,JUN,CASP3,MMP16,GNG2,RACK1,DCC,CCND1,PRKAR1A |
| Hepatic Fibrosis / Hepatic Stellate Cell Activation | 0.591 | 0.0428 | #NUM! | MYH10,COL6A1,COL6A2,PDGFRA,COL9A3,COL20A1,KLF12,IFNAR1 |
| Sirtuin Signaling Pathway | 0.578 | 0.0377 | 0 | ATP5PF,TOMM22,H3F3A/H3F3B,JUN,NDUFV3,TOMM20,SDHC,H1F0,SOD1,LDHB,APP |
| Sphingosine-1-phosphate Signaling | 0.578 | 0.048 | 0.816 | PTK2,CASP3,CASP2,PDGFRA,GNAI1,PLCG1 |
| Calcium-induced T Lymphocyte Apoptosis | 0.578 | 0.0606 | #NUM! | CALM1 (includes others),HDAC2,PLCG1,PPP3CA |
| Sumoylation Pathway | 0.564 | 0.0521 | 0 | JUN,HDAC2,SENP5,SLC19A1,ZEB1 |
| EGF Signaling | 0.564 | 0.0588 | 2 | CSNK2A1,JUN,MAP3K1,PLCG1 |
| Remodeling of Epithelial Adherens Junctions | 0.564 | 0.058 | #NUM! | TUBB3,ACTR3,TUBB4A,TUBB |
| GADD45 Signaling | 0.564 | 0.105 | #NUM! | CCND2,CCND1 |
| D-myo-inositol (1,3,4)-trisphosphate Biosynthesis | 0.564 | 0.105 | #NUM! | PMPCA,PTEN |
| Diphthamide Biosynthesis | 0.564 | 0.333 | #NUM! | EEF2 |
| NADH Repair | 0.564 | 0.333 | #NUM! | NAXE |
| 5-aminoimidazole Ribonucleotide Biosynthesis I | 0.564 | 0.333 | #NUM! | GART |
| Inosine-5'-phosphate Biosynthesis II | 0.564 | 0.333 | #NUM! | PAICS |
| Oxidized GTP and dGTP Detoxification | 0.564 | 0.333 | #NUM! | DDX6 |
| Dopamine-DARPP32 Feedback in cAMP Signaling | 0.556 | 0.0427 | 1.89 | CALM1 (includes others),PPP2R1A,CSNK1A1,GNAI1,PLCG1,PPP3CA,PRKAR1A |
| CD28 Signaling in T Helper Cells | 0.553 | 0.0455 | 2.449 | CALM1 (includes others),ACTR3,JUN,MAP3K1,PLCG1,PPP3CA |
| Granzyme A Signaling | 0.546 | 0.1 | #NUM! | SET,H1F0 |
| Melatonin Signaling | 0.54 | 0.0556 | 2 | CALM1 (includes others),GNAI1,PLCG1,PRKAR1A |
| IL-8 Signaling | 0.523 | 0.0394 | 2.449 | PTK2,GNB4,CCND2,JUN,GNG2,RACK1,GNAI1,CCND1 |
| ERK/MAPK Signaling | 0.518 | 0.0392 | 1.633 | PTK2,YWHAQ,PPP2R1A,H3F3A/H3F3B,CRKL,YWHAZ,PLCG1,PRKAR1A |
| Thyroid Cancer Signaling | 0.518 | 0.0652 | #NUM! | TCF4,NTRK3,CCND1 |
| FAK Signaling | 0.518 | 0.0476 | #NUM! | PTK2,CAPN5,ARHGEF7,PLCG1,PTEN |
| Agrin Interactions at Neuromuscular Junction | 0.513 | 0.0533 | 2 | PTK2,JUN,ARHGEF7,CTTN |
| nNOS Signaling in Neurons | 0.511 | 0.0638 | #NUM! | CAPN5,CALM1 (includes others),PPP3CA |
| Corticotropin Releasing Hormone Signaling | 0.511 | 0.0432 | 1 | CALM1 (includes others),CACNB1,JUN,GNAI1,PLCG1,PRKAR1A |
| Myc Mediated Apoptosis Signaling | 0.51 | 0.0526 | #NUM! | YWHAQ,YWHAE,CASP3,YWHAZ |
| Aryl Hydrocarbon Receptor Signaling | 0.501 | 0.0426 | 1.342 | CCND2,JUN,NFIA,GSTA4,CCND1,SMARCA4 |
| Cardiac β-adrenergic Signaling | 0.501 | 0.0426 | #NUM! | GNB4,PPP2R1A,GNG2,RACK1,AKAP7,PRKAR1A |
| Opioid Signaling Pathway | 0.491 | 0.0364 | 2.333 | CALM1 (includes others),YES1,CACNB1,RGS3,AP2B1,GNG2,GNAI1,PPP3CA,PRKAR1A |
| Gαs Signaling | 0.488 | 0.0455 | #NUM! | GNB4,ADD2,GNG2,RACK1,PRKAR1A |
| Cell Cycle: G2/M DNA Damage Checkpoint Regulation | 0.477 | 0.06 | #NUM! | YWHAQ,YWHAE,YWHAZ |
| Superpathway of D-myo-inositol (1,4,5)-trisphosphate Metabolism | 0.477 | 0.0833 | #NUM! | PMPCA,PTEN |
| GPCR-Mediated Nutrient Sensing in Enteroendocrine Cells | 0.475 | 0.0446 | 1.342 | CACNB1,GNG2,GNAI1,PLCG1,PRKAR1A |
| Cyclins and Cell Cycle Regulation | 0.467 | 0.0494 | #NUM! | PPP2R1A,CCND2,HDAC2,CCND1 |
| BMP signaling pathway | 0.463 | 0.0488 | #NUM! | MAGED1,JUN,BMPR1A,PRKAR1A |
| Creatine-phosphate Biosynthesis | 0.463 | 0.2 | #NUM! | CKB |
| Tetrahydrofolate Salvage from 5,10-methenyltetrahydrofolate | 0.463 | 0.2 | #NUM! | GART |
| Serine Biosynthesis | 0.463 | 0.2 | #NUM! | PSAT1 |
| T Cell Receptor Signaling | 0.463 | 0.0435 | #NUM! | CALM1 (includes others),JUN,MAP3K1,PLCG1,PPP3CA |
| CD27 Signaling in Lymphocytes | 0.462 | 0.0566 | #NUM! | JUN,CASP3,MAP3K1 |
| Semaphorin Signaling in Neurons | 0.462 | 0.0566 | #NUM! | PTK2,CRMP1,DPYSL5 |
| Hereditary Breast Cancer Signaling | 0.462 | 0.04 | #NUM! | NPM1,HDAC2,SLC19A1,CCND1,SMARCA4,PTEN |
| Small Cell Lung Cancer Signaling | 0.446 | 0.0471 | #NUM! | PTK2,TRAF4,CCND1,PTEN |
| D-myo-inositol (1,4,5)-Trisphosphate Biosynthesis | 0.438 | 0.0741 | #NUM! | PIP5K1A,PLCG1 |
| RAR Activation | 0.432 | 0.0368 | #NUM! | CSNK2A1,JUN,MAP3K1,RPL7A,SMARCA4,PRKAR1A,PTEN |
| Glioma Signaling | 0.432 | 0.0417 | 1 | CALM1 (includes others),PDGFRA,PLCG1,CCND1,PTEN |
| Pyruvate Fermentation to Lactate | 0.427 | 0.167 | #NUM! | LDHB |
| Glycine Cleavage Complex | 0.427 | 0.167 | #NUM! | GCSH |
| Glycogen Biosynthesis II (from UDP-D-Glucose) | 0.427 | 0.167 | #NUM! | UGP2 |
| Role of CHK Proteins in Cell Cycle Checkpoint Control | 0.423 | 0.0526 | #NUM! | PPP2R1A,SLC19A1,TLK1 |
| Gαi Signaling | 0.421 | 0.0407 | #NUM! | GNB4,GNG2,RACK1,GNAI1,PRKAR1A |
| Relaxin Signaling | 0.421 | 0.038 | #NUM! | GNB4,JUN,GNG2,RACK1,GNAI1,PRKAR1A |
| Regulation of Actin-based Motility by Rho | 0.416 | 0.0444 | 2 | PIP5K1A,ACTR3,PFN2,WASF1 |
| FGF Signaling | 0.41 | 0.044 | 2 | CRKL,FGF14,MAP3K1,PLCG1 |
| ILK Signaling | 0.41 | 0.0355 | 0.378 | PTK2,MYH10,PPP2R1A,JUN,CASP3,CCND1,PTEN |
| Gαq Signaling | 0.41 | 0.0373 | 2 | CALM1 (includes others),GNB4,GNG2,RACK1,PLCG1,PPP3CA |
| TNFR2 Signaling | 0.41 | 0.0667 | #NUM! | JUN,MAP3K1 |
| Sperm Motility | 0.403 | 0.0394 | 2.236 | PTK2,PAFAH1B2,CALM1 (includes others),PLCG1,PRKAR1A |
| Superpathway of Serine and Glycine Biosynthesis I | 0.403 | 0.143 | #NUM! | PSAT1 |
| NAD Salvage Pathway III | 0.403 | 0.143 | #NUM! | NMRK1 |
| Renin-Angiotensin Signaling | 0.4 | 0.0391 | 2.236 | PTK2,JUN,MAP3K1,PLCG1,PRKAR1A |
| Neuregulin Signaling | 0.397 | 0.0426 | #NUM! | CRKL,PLCG1,TMEFF2,PTEN |
| Retinoic acid Mediated Apoptosis Signaling | 0.389 | 0.0484 | #NUM! | CASP3,TNFRSF10B,IFNAR1 |
| Apoptosis Signaling | 0.384 | 0.0417 | 1 | CAPN5,CASP3,CASP2,PLCG1 |
| Cellular Effects of Sildenafil (Viagra) | 0.384 | 0.0382 | #NUM! | MYH10,CALM1 (includes others),KCNQ2,PLCG1,PRKAR1A |
| Regulation of Cellular Mechanics by Calpain Protease | 0.384 | 0.0476 | #NUM! | PTK2,CAPN5,CCND1 |
| Glioblastoma Multiforme Signaling | 0.382 | 0.0357 | 0.447 | TSC1,PDGFRA,NF2,PLCG1,CCND1,PTEN |
| Phospholipase C Signaling | 0.378 | 0.0328 | 2.449 | CALM1 (includes others),GNB4,HDAC2,ARHGEF7,GNG2,RACK1,PLCG1,PPP3CA |
| Superoxide Radicals Degradation | 0.378 | 0.125 | #NUM! | SOD1 |
| Adipogenesis pathway | 0.374 | 0.0373 | #NUM! | KAT7,HDAC2,BMPR1A,SOX9,RBBP4 |
| Mitochondrial Dysfunction | 0.374 | 0.0351 | #NUM! | ATP5PF,CASP3,NDUFV3,LRRK2,SDHC,APP |
| CXCR4 Signaling | 0.374 | 0.0351 | 2 | PTK2,GNB4,JUN,GNG2,RACK1,GNAI1 |
| Inhibition of Angiogenesis by TSP1 | 0.374 | 0.0588 | #NUM! | JUN,CASP3 |
| Mitotic Roles of Polo-Like Kinase | 0.37 | 0.0455 | #NUM! | SMC3,PPP2R1A,CDC26 |
| CCR3 Signaling in Eosinophils | 0.37 | 0.0368 | #NUM! | CALM1 (includes others),GNB4,GNG2,RACK1,GNAI1 |
| PI3K Signaling in B Lymphocytes | 0.37 | 0.0368 | 1.342 | CALM1 (includes others),JUN,PLCG1,PPP3CA,PTEN |
| Phosphatidylethanolamine Biosynthesis II | 0.358 | 0.111 | #NUM! | ETNK1 |
| RANK Signaling in Osteoclasts | 0.358 | 0.0392 | 2 | CALM1 (includes others),JUN,MAP3K1,PPP3CA |
| STAT3 Pathway | 0.351 | 0.0388 | 2 | NTRK3,BMPR1A,PDGFRA,IFNAR1 |
| AMPK Signaling | 0.344 | 0.0324 | 0.447 | TSC1,PPP2R1A,EEF2,RAB11A,CCND1,SMARCA4,PRKAR1A |
| Gα12/13 Signaling | 0.343 | 0.0355 | 0.447 | PTK2,CDH2,JUN,MAP3K1,CDH13 |
| Cell Cycle Regulation by BTG Family Proteins | 0.343 | 0.0541 | #NUM! | PPP2R1A,CCND1 |
| Germ Cell-Sertoli Cell Junction Signaling | 0.343 | 0.0335 | #NUM! | PTK2,TUBB3,CDH2,MAP3K1,TUBB4A,TUBB |
| PAK Signaling | 0.325 | 0.0374 | 2 | PTK2,CASP3,ARHGEF7,PDGFRA |
| ERK5 Signaling | 0.322 | 0.0417 | #NUM! | YWHAQ,YWHAE,YWHAZ |
| April Mediated Signaling | 0.32 | 0.0513 | #NUM! | JUN,MAP3K1 |
| GPCR-Mediated Integration of Enteroendocrine Signaling Exemplified by an L Cell | 0.317 | 0.0411 | #NUM! | GNAI1,PLCG1,PRKAR1A |
| VEGF Signaling | 0.317 | 0.0367 | #NUM! | PTK2,YWHAE,PLCG1,EIF2S3 |
| SAPK/JNK Signaling | 0.314 | 0.0364 | 2 | JUN,CRKL,GNG2,MAP3K1 |
| Phagosome Maturation | 0.314 | 0.0338 | #NUM! | TUBB3,PRDX1,TUBB4A,GOSR1,TUBB |
| NF-κB Signaling | 0.314 | 0.0321 | 1.633 | CSNK2A1,HDAC2,NTRK3,BMPR1A,MAP3K1,PDGFRA |
| ErbB2-ErbB3 Signaling | 0.314 | 0.04 | #NUM! | JUN,CCND1,PTEN |
| Hypoxia Signaling in the Cardiovascular System | 0.314 | 0.04 | #NUM! | UBE2G2,JUN,PTEN |
| B Cell Activating Factor Signaling | 0.314 | 0.0488 | #NUM! | JUN,MAP3K1 |
| Mechanisms of Viral Exit from Host Cells | 0.314 | 0.0488 | #NUM! | LMNB1,CHMP3 |
| nNOS Signaling in Skeletal Muscle Cells | 0.314 | 0.0488 | #NUM! | CALM1 (includes others),CACNB1 |
| Toll-like Receptor Signaling | 0.308 | 0.0395 | #NUM! | JUN,TRAF4,MAP3K1 |
| Acyl-CoA Hydrolysis | 0.303 | 0.0833 | #NUM! | GNPAT |
| Role of Wnt/GSK-3β Signaling in the Pathogenesis of Influenza | 0.303 | 0.039 | #NUM! | TCF4,CSNK1A1,IFNAR1 |
| Virus Entry via Endocytic Pathways | 0.297 | 0.0345 | #NUM! | AP3M1,ITSN1,AP2B1,PLCG1 |
| B Cell Receptor Signaling | 0.297 | 0.0309 | 1.633 | PTK2,CALM1 (includes others),JUN,MAP3K1,PPP3CA,PTEN |
| Role of PI3K/AKT Signaling in the Pathogenesis of Influenza | 0.297 | 0.038 | #NUM! | CRKL,GNAI1,IFNAR1 |
| GM-CSF Signaling | 0.297 | 0.038 | #NUM! | RACK1,CCND1,PPP3CA |
| Assembly of RNA Polymerase III Complex | 0.297 | 0.0769 | #NUM! | GTF3C5 |
| Oleate Biosynthesis II (Animals) | 0.297 | 0.0769 | #NUM! | SCD5 |
| Cholesterol Biosynthesis I | 0.297 | 0.0769 | #NUM! | LSS |
| Cholesterol Biosynthesis II (via 24,25-dihydrolanosterol) | 0.297 | 0.0769 | #NUM! | LSS |
| Cholesterol Biosynthesis III (via Desmosterol) | 0.297 | 0.0769 | #NUM! | LSS |
| Superpathway of Inositol Phosphate Compounds | 0.297 | 0.0297 | 2.646 | SET,PIP5K1A,PDGFRA,PLCG1,PMPCA,PPP3CA,PTEN |
| Endothelin-1 Signaling | 0.297 | 0.0306 | 2.449 | PAFAH1B2,JUN,CASP3,CASP2,GNAI1,PLCG1 |
| iNOS Signaling | 0.293 | 0.0444 | #NUM! | CALM1 (includes others),JUN |
| Antiproliferative Role of Somatostatin Receptor 2 | 0.293 | 0.037 | #NUM! | GNB4,GNG2,RACK1 |
| NRF2-mediated Oxidative Stress Response | 0.288 | 0.0302 | #NUM! | JUN,PRDX1,GSTA4,MAP3K1,SOD1,DNAJC11 |
| Neuroprotective Role of THOP1 in Alzheimer's Disease | 0.288 | 0.0333 | #NUM! | YWHAE,MAPT,PRKAR1A,APP |
| Colanic Acid Building Blocks Biosynthesis | 0.287 | 0.0714 | #NUM! | UGP2 |
| Adrenomedullin signaling pathway | 0.287 | 0.03 | 2.236 | PTK2,CALM1 (includes others),KCNQ2,CASP3,PLCG1,PRKAR1A |
| Neurotrophin/TRK Signaling | 0.286 | 0.0361 | #NUM! | JUN,NTRK3,PLCG1 |
| iCOS-iCOSL Signaling in T Helper Cells | 0.274 | 0.0325 | 1 | CALM1 (includes others),PLCG1,PPP3CA,PTEN |
| Telomere Extension by Telomerase | 0.27 | 0.0667 | #NUM! | HNRNPA2B1 |
| NGF Signaling | 0.266 | 0.032 | #NUM! | TRAF4,MAP3K1,PLCG1,TRIO |
| PKCθ Signaling in T Lymphocytes | 0.266 | 0.0303 | 2 | CACNB1,JUN,MAP3K1,PLCG1,PPP3CA |
| Extrinsic Prothrombin Activation Pathway | 0.257 | 0.0625 | #NUM! | TFPI |
| Mismatch Repair in Eukaryotes | 0.257 | 0.0625 | #NUM! | SLC19A1 |
| Chondroitin Sulfate Degradation (Metazoa) | 0.257 | 0.0625 | #NUM! | MGEA5 |
| Thrombin Signaling | 0.252 | 0.0286 | #NUM! | PTK2,GNB4,GNG2,RACK1,GNAI1,PLCG1 |
| IL-3 Signaling | 0.252 | 0.0337 | #NUM! | JUN,CRKL,PPP3CA |
| Osteoarthritis Pathway | 0.249 | 0.0283 | 1.633 | TCF4,CASP3,BMPR1A,CASP2,SOX9,JAG1 |
| Docosahexaenoic Acid (DHA) Signaling | 0.249 | 0.0385 | #NUM! | CASP3,APP |
| RAN Signaling | 0.249 | 0.0588 | #NUM! | KPNA6 |
| Dermatan Sulfate Degradation (Metazoa) | 0.249 | 0.0588 | #NUM! | MGEA5 |
| IL-7 Signaling Pathway | 0.246 | 0.033 | #NUM! | PTK2,JUN,CCND1 |
| D-myo-inositol (1,4,5)-trisphosphate Degradation | 0.236 | 0.0556 | #NUM! | PMPCA |
| Death Receptor Signaling | 0.236 | 0.0323 | #NUM! | CASP3,CASP2,TNFRSF10B |
| Transcriptional Regulatory Network in Embryonic Stem Cells | 0.236 | 0.037 | #NUM! | SET,H3F3A/H3F3B |
| Estrogen Receptor Signaling | 0.235 | 0.0299 | #NUM! | H3F3A/H3F3B,NR0B1,HNRNPD,SMARCA4 |
| Bladder Cancer Signaling | 0.235 | 0.0319 | #NUM! | MMP16,FGF14,CCND1 |
| HER-2 Signaling in Breast Cancer | 0.235 | 0.0319 | #NUM! | TSC1,PLCG1,CCND1 |
| GABA Receptor Signaling | 0.229 | 0.0316 | #NUM! | CACNB1,KCNQ2,AP2B1 |
| 1D-myo-inositol Hexakisphosphate Biosynthesis II (Mammalian) | 0.228 | 0.0526 | #NUM! | PMPCA |
| Molecular Mechanisms of Cancer | 0.222 | 0.0254 | #NUM! | PTK2,TCF4,CCND2,JUN,CASP3,BMPR1A,ARHGEF7,GNAI1,CCND1,PRKAR1A |
| Protein Ubiquitination Pathway | 0.222 | 0.0264 | #NUM! | PSMB4,UBE2G2,UCHL1,USP22,USP10,UBE4A,DNAJC11 |
| Cardiomyocyte Differentiation via BMP Receptors | 0.216 | 0.05 | #NUM! | BMPR1A |
| Ceramide Signaling | 0.212 | 0.0303 | #NUM! | PPP2R1A,JUN,MAP3K1 |
| CTLA4 Signaling in Cytotoxic T Lymphocytes | 0.212 | 0.0303 | #NUM! | PPP2R1A,AP2B1,PLCG1 |
| Endoplasmic Reticulum Stress Pathway | 0.206 | 0.0476 | #NUM! | CASP3 |
| Human Embryonic Stem Cell Pluripotency | 0.203 | 0.028 | #NUM! | TCF4,NTRK3,BMPR1A,PDGFRA |
| Induction of Apoptosis by HIV1 | 0.201 | 0.0328 | #NUM! | CASP3,SLC25A3 |
| Melanoma Signaling | 0.201 | 0.0328 | #NUM! | CCND1,PTEN |
| PCP pathway | 0.201 | 0.0328 | #NUM! | JUN,PFN2 |
| Polyamine Regulation in Colon Cancer | 0.201 | 0.0455 | #NUM! | TCF4 |
| Role of Macrophages, Fibroblasts and Endothelial Cells in Rheumatoid Arthritis | 0.201 | 0.025 | #NUM! | CALM1 (includes others),TCF4,JUN,TRAF4,CSNK1A1,PLCG1,CCND1,PPP3CA |
| Actin Nucleation by ARP-WASP Complex | 0.201 | 0.0323 | #NUM! | ACTR3,WASF1 |
| Phospholipases | 0.201 | 0.0323 | #NUM! | PAFAH1B2,PLCG1 |
| Role of Osteoblasts, Osteoclasts and Chondrocytes in Rheumatoid Arthritis | 0.201 | 0.0258 | #NUM! | CALM1 (includes others),TCF4,JUN,BMPR1A,CSNK1A1,PPP3CA |
| Insulin Receptor Signaling | 0.201 | 0.0272 | #NUM! | TSC1,CRKL,PRKAR1A,PTEN |
| Role of Lipids/Lipid Rafts in the Pathogenesis of Influenza | 0.201 | 0.0435 | #NUM! | IFNAR1 |
| Activation of IRF by Cytosolic Pattern Recognition Receptors | 0.201 | 0.0317 | #NUM! | JUN,IFNAR1 |
| Wnt/Ca+ pathway | 0.201 | 0.0317 | #NUM! | PLCG1,PPP3CA |
| CDK5 Signaling | 0.201 | 0.0286 | #NUM! | PPP2R1A,MAPT,PRKAR1A |
| Agranulocyte Adhesion and Diapedesis | 0.197 | 0.0259 | #NUM! | MYH10,PODXL2,JAM3,MMP16,GNAI1 |
| Ovarian Cancer Signaling | 0.197 | 0.0267 | #NUM! | TCF4,CCND1,PRKAR1A,PTEN |
| Tumoricidal Function of Hepatic Natural Killer Cells | 0.197 | 0.0417 | #NUM! | CASP3 |
| Role of JAK1, JAK2 and TYK2 in Interferon Signaling | 0.197 | 0.0417 | #NUM! | IFNAR1 |
| TCA Cycle II (Eukaryotic) | 0.197 | 0.0417 | #NUM! | SDHC |
| Regulation of the Epithelial-Mesenchymal Transition Pathway | 0.195 | 0.0256 | #NUM! | TCF4,CDH2,FGF14,ZEB1,JAG1 |
| UVB-Induced MAPK Signaling | 0.191 | 0.0303 | #NUM! | H3F3A/H3F3B,JUN |
| Oxidative Phosphorylation | 0.191 | 0.0275 | #NUM! | ATP5PF,NDUFV3,SDHC |
| IL-17A Signaling in Gastric Cells | 0.191 | 0.04 | #NUM! | JUN |
| Lymphotoxin β Receptor Signaling | 0.19 | 0.0299 | #NUM! | CASP3,TRAF4 |
| Lipid Antigen Presentation by CD1 | 0.19 | 0.0385 | #NUM! | AP2B1 |
| Estrogen-mediated S-phase Entry | 0.19 | 0.0385 | #NUM! | CCND1 |
| NAD Salvage Pathway II | 0.19 | 0.0385 | #NUM! | NMRK1 |
| Glycolysis I | 0.19 | 0.0385 | #NUM! | ENO2 |
| Gluconeogenesis I | 0.19 | 0.0385 | #NUM! | ENO2 |
| Chronic Myeloid Leukemia Signaling | 0.19 | 0.0268 | #NUM! | HDAC2,CRKL,CCND1 |
| UVA-Induced MAPK Signaling | 0.19 | 0.0268 | #NUM! | JUN,CASP3,PLCG1 |
| 3-phosphoinositide Biosynthesis | 0.19 | 0.0249 | 2.236 | SET,PIP5K1A,PDGFRA,PPP3CA,PTEN |
| Endometrial Cancer Signaling | 0.184 | 0.0286 | #NUM! | CCND1,PTEN |
| IL-2 Signaling | 0.184 | 0.0286 | #NUM! | CSNK2A1,JUN |
| Thrombopoietin Signaling | 0.18 | 0.0282 | #NUM! | JUN,PLCG1 |
| IL-15 Production | 0.18 | 0.0357 | #NUM! | PTK2 |
| Superpathway of Cholesterol Biosynthesis | 0.18 | 0.0357 | #NUM! | LSS |
| D-myo-inositol-5-phosphate Metabolism | 0.175 | 0.0247 | 2 | SET,PLCG1,PPP3CA,PTEN |
| Sonic Hedgehog Signaling | 0.161 | 0.0333 | #NUM! | PRKAR1A |
| Glutathione-mediated Detoxification | 0.153 | 0.0323 | #NUM! | GSTA4 |
| Dopamine Receptor Signaling | 0.151 | 0.026 | #NUM! | PPP2R1A,PRKAR1A |
| HIF1α Signaling | 0.151 | 0.0242 | #NUM! | JUN,MMP16,LDHB |
| Cytotoxic T Lymphocyte-mediated Apoptosis of Target Cells | 0.151 | 0.0312 | #NUM! | CASP3 |
| 4-1BB Signaling in T Lymphocytes | 0.151 | 0.0312 | #NUM! | JUN |
| Role of MAPK Signaling in the Pathogenesis of Influenza | 0.151 | 0.0256 | #NUM! | PAFAH1B2,CASP3 |
| Neuroinflammation Signaling Pathway | 0.149 | 0.0225 | 1.89 | JUN,CASP3,MAPT,S100B,PLCG1,PPP3CA,APP |
| eNOS Signaling | 0.149 | 0.0233 | 2 | CALM1 (includes others),CASP3,PLCG1,PRKAR1A |
| Role of BRCA1 in DNA Damage Response | 0.144 | 0.025 | #NUM! | SLC19A1,SMARCA4 |
| IL-15 Signaling | 0.14 | 0.0244 | #NUM! | PTK2,PLCG1 |
| GDNF Family Ligand-Receptor Interactions | 0.14 | 0.0244 | #NUM! | JUN,PLCG1 |
| Coagulation System | 0.14 | 0.0286 | #NUM! | TFPI |
| TWEAK Signaling | 0.14 | 0.0286 | #NUM! | CASP3 |
| IL-17A Signaling in Fibroblasts | 0.14 | 0.0286 | #NUM! | JUN |
| Role of Tissue Factor in Cancer | 0.14 | 0.0231 | #NUM! | YES1,CASP3,PTEN |
| Non-Small Cell Lung Cancer Signaling | 0.139 | 0.0241 | #NUM! | PLCG1,CCND1 |
| Interferon Signaling | 0.135 | 0.0278 | #NUM! | IFNAR1 |
| cAMP-mediated signaling | 0.134 | 0.0219 | 1 | CALM1 (includes others),GNAI1,AKAP7,PPP3CA,PRKAR1A |
| Granulocyte Adhesion and Diapedesis | 0.134 | 0.0221 | #NUM! | JAM3,MMP16,GNAI1,THY1 |
| Dopamine Degradation | 0.134 | 0.027 | #NUM! | LRTOMT |
| Estrogen-Dependent Breast Cancer Signaling | 0.134 | 0.0233 | #NUM! | JUN,CCND1 |
| Leptin Signaling in Obesity | 0.134 | 0.0233 | #NUM! | PLCG1,PRKAR1A |
| Erythropoietin Signaling | 0.13 | 0.023 | #NUM! | JUN,PLCG1 |
| tRNA Charging | 0.125 | 0.0256 | #NUM! | LARS |
| Inhibition of Matrix Metalloproteases | 0.125 | 0.0256 | #NUM! | MMP16 |
| Prolactin Signaling | 0.125 | 0.0225 | #NUM! | JUN,PLCG1 |
| Noradrenaline and Adrenaline Degradation | 0.122 | 0.025 | #NUM! | LRTOMT |
| Role of PKR in Interferon Induction and Antiviral Response | 0.117 | 0.0244 | #NUM! | CASP3 |
| Factors Promoting Cardiogenesis in Vertebrates | 0.117 | 0.0217 | #NUM! | TCF4,BMPR1A |
| MIF Regulation of Innate Immunity | 0.108 | 0.0233 | #NUM! | JUN |
| Role of p14/p19ARF in Tumor Suppression | 0.108 | 0.0233 | #NUM! | NPM1 |
| Stearate Biosynthesis I (Animals) | 0.104 | 0.0227 | #NUM! | GNPAT |
| Role of Oct4 in Mammalian Embryonic Stem Cell Pluripotency | 0.0956 | 0.0217 | #NUM! | KDM5B |
| Dermatan Sulfate Biosynthesis (Late Stages) | 0.0956 | 0.0217 | #NUM! | DSEL |
| Glucocorticoid Receptor Signaling | 0 | 0.0116 | #NUM! | JUN,MAP3K1,PPP3CA,SMARCA4 |
| Natural Killer Cell Signaling | 0 | 0.00781 | #NUM! | PLCG1 |
| IL-10 Signaling | 0 | 0.0145 | #NUM! | JUN |
| Fc Epsilon RI Signaling | 0 | 0.008 | #NUM! | PLCG1 |
| PPARα/RXRα Activation | 0 | 0.0161 | #NUM! | JUN,PLCG1,PRKAR1A |
| LPS/IL-1 Mediated Inhibition of RXR Function | 0 | 0.0135 | #NUM! | JUN,GSTA4,MAP3K1 |
| Acute Phase Response Signaling | 0 | 0.017 | #NUM! | TCF4,JUN,MAP3K1 |
| Hepatic Cholestasis | 0 | 0.0125 | #NUM! | JUN,PRKAR1A |
| VDR/RXR Activation | 0 | 0.0128 | #NUM! | CSNK2A1 |
| PXR/RXR Activation | 0 | 0.0154 | #NUM! | PRKAR1A |
| TR/RXR Activation | 0 | 0.0102 | #NUM! | SREBF2 |
| Caveolar-mediated Endocytosis Signaling | 0 | 0.0141 | #NUM! | ITSN1 |
| IL-12 Signaling and Production in Macrophages | 0 | 0.00685 | #NUM! | JUN |
| TREM1 Signaling | 0 | 0.0133 | #NUM! | PLCG1 |
| FcγRIIB Signaling in B Lymphocytes | 0 | 0.0118 | #NUM! | CACNB1 |
| LPS-stimulated MAPK Signaling | 0 | 0.0108 | #NUM! | JUN |
| NF-κB Activation by Viruses | 0 | 0.0108 | #NUM! | MAP3K1 |
| CD40 Signaling | 0 | 0.0127 | #NUM! | JUN |
| IL-17 Signaling | 0 | 0.011 | #NUM! | JUN |
| Dendritic Cell Maturation | 0 | 0.0103 | #NUM! | PLCG1,IFNAR1 |
| Angiopoietin Signaling | 0 | 0.0119 | #NUM! | PTK2 |
| HMGB1 Signaling | 0 | 0.0144 | #NUM! | KAT7,JUN |
| Cholecystokinin/Gastrin-mediated Signaling | 0 | 0.0187 | #NUM! | PTK2,JUN |
| Melanocyte Development and Pigmentation Signaling | 0 | 0.0192 | #NUM! | PLCG1,PRKAR1A |
| Aldosterone Signaling in Epithelial Cells | 0 | 0.0179 | #NUM! | PIP5K1A,PLCG1,DNAJC11 |
| Role of NANOG in Mammalian Embryonic Stem Cell Pluripotency | 0 | 0.00781 | #NUM! | BMPR1A |
| Growth Hormone Signaling | 0 | 0.0118 | #NUM! | PLCG1 |
| Prostate Cancer Signaling | 0 | 0.0194 | #NUM! | CCND1,PTEN |
| Renal Cell Carcinoma Signaling | 0 | 0.0114 | #NUM! | JUN |
| Type I Diabetes Mellitus Signaling | 0 | 0.00901 | #NUM! | CASP3 |
| Basal Cell Carcinoma Signaling | 0 | 0.0139 | #NUM! | TCF4 |
| Acute Myeloid Leukemia Signaling | 0 | 0.0202 | #NUM! | TCF4,CCND1 |
| Type II Diabetes Mellitus Signaling | 0 | 0.013 | #NUM! | CACNB1,MAP3K1 |
| Production of Nitric Oxide and Reactive Oxygen Species in Macrophages | 0 | 0.0206 | 1 | PPP2R1A,JUN,MAP3K1,PLCG1 |
| Pancreatic Adenocarcinoma Signaling | 0 | 0.00833 | #NUM! | CCND1 |
| Systemic Lupus Erythematosus Signaling | 0 | 0.0172 | #NUM! | JUN,HNRNPA2B1,SNRPB2,PLCG1 |
| Cdc42 Signaling | 0 | 0.018 | #NUM! | ACTR3,JUN,CDC42SE1 |
| Atherosclerosis Signaling | 0 | 0.00787 | #NUM! | PAFAH1B2 |
| Glioma Invasiveness Signaling | 0 | 0.0132 | #NUM! | PTK2 |
| OX40 Signaling Pathway | 0 | 0.011 | #NUM! | JUN |
| Cell Cycle Control of Chromosomal Replication | 0 | 0.0179 | #NUM! | ORC4 |
| IL-17A Signaling in Airway Cells | 0 | 0.0128 | #NUM! | PTEN |
| Paxillin Signaling | 0 | 0.0168 | #NUM! | PTK2,ARHGEF7 |
| Telomerase Signaling | 0 | 0.0171 | #NUM! | PPP2R1A,HDAC2 |
| Mouse Embryonic Stem Cell Pluripotency | 0 | 0.0179 | #NUM! | TCF4,BMPR1A |
| VEGF Family Ligand-Receptor Interactions | 0 | 0.0106 | #NUM! | PLCG1 |
| Ephrin A Signaling | 0 | 0.0167 | #NUM! | PTK2 |
| ErbB Signaling | 0 | 0.0192 | #NUM! | JUN,PLCG1 |
| ErbB4 Signaling | 0 | 0.0128 | #NUM! | PLCG1 |
| Pyridoxal 5'-phosphate Salvage Pathway | 0 | 0.0154 | #NUM! | CSNK1A1 |
| Dermatan Sulfate Biosynthesis | 0 | 0.0169 | #NUM! | DSEL |
| D-myo-inositol (1,4,5,6)-Tetrakisphosphate Biosynthesis | 0 | 0.0208 | #NUM! | SET,PPP3CA,PTEN |
| D-myo-inositol (3,4,5,6)-tetrakisphosphate Biosynthesis | 0 | 0.0208 | #NUM! | SET,PPP3CA,PTEN |
| 3-phosphoinositide Degradation | 0 | 0.019 | #NUM! | SET,PPP3CA,PTEN |
| Salvage Pathways of Pyrimidine Ribonucleotides | 0 | 0.0103 | #NUM! | CSNK1A1 |
| Antioxidant Action of Vitamin C | 0 | 0.0185 | #NUM! | PAFAH1B2,PLCG1 |
| PEDF Signaling | 0 | 0.0215 | #NUM! | TCF4,ZEB1 |
| UVC-Induced MAPK Signaling | 0 | 0.0204 | #NUM! | JUN |
| Unfolded protein response | 0 | 0.0179 | #NUM! | SREBF2 |
| Nitric Oxide Signaling in the Cardiovascular System | 0 | 0.0177 | #NUM! | CALM1 (includes others),PRKAR1A |
| JAK/Stat Signaling | 0 | 0.0112 | #NUM! | JUN |
| Xenobiotic Metabolism Signaling | 0 | 0.0101 | #NUM! | PPP2R1A,GSTA4,MAP3K1 |
| Phototransduction Pathway | 0 | 0.0189 | #NUM! | PRKAR1A |
| PPAR Signaling | 0 | 0.0198 | #NUM! | JUN,PDGFRA |
| p38 MAPK Signaling | 0 | 0.0167 | #NUM! | H3F3A/H3F3B,MAPT |
| TGF-β Signaling | 0 | 0.0215 | #NUM! | JUN,BMPR1A |
| G-Protein Coupled Receptor Signaling | 0 | 0.00709 | #NUM! | GNAI1,PRKAR1A |
| IL-6 Signaling | 0 | 0.0149 | #NUM! | CSNK2A1,JUN |
| Gustation Pathway | 0 | 0.0195 | #NUM! | CACNB1,GNG2,PRKAR1A |
| Phagosome Formation | 0 | 0.00763 | #NUM! | PLCG1 |
| Macropinocytosis Signaling | 0 | 0.0115 | #NUM! | PLCG1 |
| Cancer Drug Resistance By Drug Efflux | 0 | 0.0182 | #NUM! | PTEN |
| Th1 and Th2 Activation Pathway | 0 | 0.0162 | #NUM! | JUN,JAG1,IFNAR1 |
| Th1 Pathway | 0 | 0.00741 | #NUM! | IFNAR1 |
| Th2 Pathway | 0 | 0.0133 | #NUM! | JUN,JAG1 |
| Iron homeostasis signaling pathway | 0 | 0.0146 | #NUM! | BMPR1A,PDGFRA |
| Eicosanoid Signaling | 0 | 0.0149 | #NUM! | PAFAH1B2 |
